# High-Dose Gestational Irradiation Disrupts Fetal Mouse Brain Structure and Region-Specific Transcriptomic Profiles

**DOI:** 10.3390/genes17101267

**Published:** 2026-10-12

**Authors:** Cameron Hourtovenko, Shayenthiran Sreetharan, Neelam Khaper, Simon J. Lees, Christopher Thome, Douglas Boreham, Christine Lalonde, Sujeenthar Tharmalingam, T.C. Tai

**Affiliations:** 1 Medical Sciences Division, NOSM University, Sudbury, ON P3E 2C6, Canada; 2 School of Natural Sciences, Laurentian University, Sudbury, ON P3E 2C6, Canada; 3 Medical Sciences Division, NOSM University, Thunder Bay, ON P7B 5E1, Canada; 4 Department of Physics and Astronomy, McMaster University, Hamilton, ON L8S 4M1, Canada; 5 Department of Biology, Lakehead University, Thunder Bay, ON P7B 5E1, Canada; 6 Health Sciences North Research Institute, Sudbury, ON P3E 2H3, Canada; 7 Department of Psychology, Nipissing University, North Bay, ON P1B 8L7, Canada; 8 Department of Child and Family Studies, Nipissing University, North Bay, ON P1B 8L7, Canada

**Keywords:** gestational radiation exposure, fetal brain development, ionizing radiation, neuroinflammation, cell migration, neurotransmitters, region-specific sensitivity, BALB/c mouse model

## Abstract

**Background/Objectives:** Prenatal exposure to ionizing radiation can disrupt neurodevelopment, yet the region-specific structural and molecular responses of the developing brain remain incompletely understood. In particular, how gestational irradiation alters transcriptional programs across distinct fetal brain regions and whether these responses differ by sex has not been fully characterized. This study examined the structural and transcriptomic responses in the fetal mouse brain following high-dose gestational irradiation. **Methods:** Pregnant BALB/c mice were exposed to 1.8 Gy of X-ray irradiation on gestational day (GD) 14.5, and fetal brains were collected on GD 18.5. **Results:** Using a previously characterized model of radiation-induced fetal growth restriction, H&E-stained sagittal fetal brain sections revealed reduced hippocampal volume and enlargement of the lateral ventricle, while cerebellar volume and optical density were not significantly altered. RNA-seq analysis of the telencephalon, diencephalon, midbrain, and hindbrain revealed region-specific transcriptional dysregulation enriched for genes associated with immune and inflammatory signalling, neurogenesis, gliogenesis, cell migration, and neurotransmitter signalling, with the strongest response observed in the telencephalon and differing DEG profiles between males and females in sex-stratified analyses. **Conclusions:** These findings indicate that high-dose gestational irradiation is associated with region-specific structural alterations and transcriptional changes in the fetal brain, generating hypotheses regarding the mechanisms of radiation-induced neurodevelopmental disruption.

## 1. Introduction

Ionizing radiation exposure during pregnancy can disrupt fetal development through DNA damage, oxidative stress, altered cell survival, and changes in gene expression [1,2,3]. Although high-dose gestational exposure is uncommon, it remains relevant in the context of accidental radiation scenarios, where exposure severity depends on dose, exposure duration, and the tissues affected [4,5,6,7]. For context, fetal doses from diagnostic imaging are typically below 50 mGy, and deterministic effects are generally considered unlikely below approximately 100 mGy [8]. The 1.8 Gy maternal dose used here is therefore well above medical diagnostic exposures and is representative of accidental or radiotherapy-level exposures. The developing central nervous system is particularly susceptible because neurodevelopment requires tightly regulated cell proliferation, migration, differentiation, gliogenesis, and synaptic organization [9,10]. Disruption of these processes during critical gestational windows may lead to persistent structural and functional abnormalities [11,12,13,14].

Fetal brain regions mature according to distinct developmental timelines, potentially creating region-specific windows of susceptibility to radiation-induced injury [9,15,16,17]. During mid-to-late gestation in mice, major brain regions undergo distinct developmental processes, including expansion of the telencephalon, hippocampal formation, ventricular zone activity, neuronal migration, glial development, and maturation of neurotransmitter systems [9]. Consistent with this, postnatal X-ray irradiation impaired hippocampal neurogenesis and altered neuronal precursor populations [15,16]. However, few studies have investigated the effects of high-dose radiation exposure during fetal brain development.

Human evidence from the atomic bombings of Hiroshima and Nagasaki indicates that prenatal radiation exposure increases the risk of intellectual disability and impaired brain growth, particularly when exposure occurs during sensitive developmental windows [11,12,18,19]. Experimental studies further demonstrate that gestational radiation exposure disrupts fetal brain development through multiple mechanisms, including reduced expression of genes associated with microcephaly, abnormal neuronal positioning, disruption of radial glial structure, and impaired neuronal migration [20,21,22]. More recent animal studies also suggest that prenatal radiation exposure can produce molecular dysregulation in the brain even when overt behavioural effects are limited [13]. Together, these findings suggest that gestational radiation disrupts fetal brain development through coordinated structural and molecular alterations, particularly during critical periods of neurodevelopment.

Despite this evidence, relatively few studies have integrated fetal brain histology with transcriptome-wide profiling across multiple brain regions in the context of high-dose gestational radiation exposure. It remains unclear whether radiation produces uniform effects across the fetal brain or whether specific regions and sexes show greater vulnerability. The current study investigated the effects of 1.8 Gy X-ray irradiation on gestational day (GD) 14.5 in BALB/c mice, with fetal brain collection on GD 18.5. High-dose gestational irradiation was hypothesized in this study to preferentially affect actively developing brain regions, resulting in structural alterations and transcriptional dysregulation of pathways associated with neuroinflammation, neurogenesis, gliogenesis, cell migration, and neurotransmission. By integrating histological assessment with region-specific transcriptomic profiling, this study provides insight into how the fetal brain responds to high-dose gestational radiation exposure.

## 2. Materials and Methods

### 2.1. Animals and Irradiation

Wildtype BALB/cAnNCrl mice, 7–8 weeks of age, were purchased from Charles River Laboratories (Montreal, QC, Canada) and housed at the Lakehead University Animal Care Facility. All protocols used in this study were approved by the Lakehead University Animal Care Committee (AUP#: 1467646). The mice were housed in a 12:12 light-dark cycle with a room humidity of 40–70%. Food and water were provided ad libitum, except during irradiation. Male mice were maintained on standard laboratory chow, while female mice were maintained on ProLab RMH 2000 breeding diet (fiber content of 4%, protein of 18% and fat of 9%). For breeding, one male was placed into a cage with two female mice overnight, to increase the likelihood of mating. The presence of a vaginal plug the following morning was designated GD 0.5. Plug-positive females were housed individually for the remainder of the study. Pregnancy was confirmed by gestational weight gain on GD 13.5, after which dams were assigned to sham or irradiated groups.

Pregnant dams were exposed to either sham irradiation or whole-body 1.8 Gy X-ray irradiation on GD 14.5, as previously described by Sreetharan et al. (2025) [23]. Sham-exposed dams were placed in the irradiation chamber for 5 min without receiving radiation. Irradiated dams received 1.8 Gy using an X-RAD320 Precision X-ray irradiator system (Precision X-Ray; Madison, CT, USA) operating at 320 kVp, with a dose rate of 921 mGy/min for 130 s. Delivered dose and dose rate were verified using thermoluminescent dosimeters (Mirion Technologies GDS Inc.; Oak Ridge, TN, USA) [23]. The beam was filtered to attenuate low-energy photons, improving dose uniformity with depth through the maternal abdomen. Because exposure was whole-body to the dam, the absorbed dose to the fetal brain was not directly measured and may therefore differ modestly from the delivered maternal dose. On GD 18.5, dams were anesthetized with isoflurane and euthanized by exsanguination. Fetuses were collected and euthanized by CO_2_. Fetal tissues were either flash frozen and stored at −80 °C or fixed overnight in 4% paraformaldehyde, followed by storage in 70% ethanol, and then shipped to NOSM University (Sudbury, ON, Canada) for downstream histological or transcriptomic analysis of the brain. Sample selection prioritized the inclusion of one male and one female fetus per dam where possible. Because irradiation was administered to the pregnant dam, the dam was treated as the experimental unit for treatment allocation. In both the histological and transcriptomic cohorts, no dam contributed more than one fetus to any sex × treatment group. Therefore, within each sex-specific comparison, every fetus originated from a different dam.

### 2.2. Fetal Sex Determination

To determine the sex of each fetus, a 5 mm fetal tail sample was collected and digested in an SDS-based tail buffer with 20 mg/mL Proteinase K (ThermoFisher Scientific, Mississauga, ON, Canada; cat. no. 25530049) following the StarrLab protocol (StarrLab, Minneapolis, MN, USA). DNA was extracted from the digested tail samples via neutralized phenol/chloroform/iso-amyl alcohol (25:24:1 ratio) based on the Jackson Laboratory protocol (The Jackson Laboratory, Bar Harbor, ME, USA) [24]. Fetal sex was determined by PCR amplification followed by gel electrophoresis using the following primer pair (Forward: 5′-CTGAAGCTTTTGGCTTTGAG-3′ and Reverse: 5′-CCACTGCCAAATTCTTTGG-3′). These primers allow for amplification of both the X and Y chromosomes through the genes *Kdm5c* and *Kdm5d*, respectively [25].

PCR was performed in 25 µL reactions using the S1000 Thermal Cycler (Bio-Rad Laboratories; Mississauga, ON, Canada). PCR began with an initial denaturation step at 95 °C for 2 min. This was followed by 35 cycles of: 30 s for denaturation at 95 °C, 30 s for annealing at 57 °C, and 1 min for extension at 72 °C. A final extension was performed at 72 °C for 5 min, followed by a hold at 4 °C. The PCR reactions included 12.5 µL of Bi2M PCR 2× Master Mix (containing 0.02 U/µL *Taq* DNA polymerase, reaction buffer, 3 mM MgCl_2_, 0.4 mM of each dNTP; MediRes Corp, Sudbury, ON, Canada; cat. no. Bi2M-2xPCR), 1.25 µL of each forward and reverse primer (final concentration of 500 nM; Integrated DNA Technologies, Coralville, IA, USA), and 10 µL of diluted DNA template (10 ng/µL concentration, totalling 100 ng per reaction). A 2% agarose gel (MediRes Corp, Sudbury, ON, Canada; cat. no. Bi2M-Agar-E) stained with 10 µL of Ser-Red Nucleic Acid Gel Stain (10,000×) (MediRes Corp, Sudbury, ON, Canada; cat. no. Bi2M-SrRed) was used to view the amplified DNA of the tail samples. Positive controls of adult male and female BALB/cAnNCrl mice were run on the same gels, with female DNA exhibiting a single band 331 bp in size (*Kdm5c*), whereas male DNA displayed both the 331 bp band and an additional 302 bp band (*Kdm5d*).

### 2.3. Histological Preparation and Analysis

#### 2.3.1. H&E Staining

Histological analysis was performed on 12 fetal heads (*n* = 3 per sex per treatment group) originating from six dams (3 sham and 3 irradiated dams, each contributing one male and one female fetus). The fetal heads were collected by decapitation, bisected sagittally, and shipped to the Sunnybrook Research Institute (Toronto, ON, Canada), where both halves were embedded together in paraffin. Embedded samples were returned to NOSM University, where the entire brain of each sample was sagittally sectioned at 10 µm and divided into five serial series. The first series was stained with hematoxylin and eosin (H&E), while the remaining four series were stored for future histological analyses. For H&E staining, sections were dewaxed in two changes in xylene (Sigma-Aldrich, St. Louis, MO, USA), rehydrated through graded ethanol (Greenfield Global, Brampton, ON, Canada), and rinsed in 1× PBS (Fisher Scientific, Fair Lawn, NJ, USA). Sections were stained with Harris hematoxylin (prepared in-house from hematoxylin, Fisher Scientific, Fair Lawn, NJ, USA; see Supplementary Table S1), differentiated in 1% acid alcohol (hydrochloric acid, Fisher Scientific, Fair Lawn, NJ, USA), blued in 0.1% sodium bicarbonate, counterstained with eosin-phloxine B (eosin Y, Fisher Scientific, Fair Lawn, NJ, USA; phloxine B, Cayman Chemical Company, Ann Arbor, MI, USA), dehydrated through graded ethanol, cleared in xylene, and coverslipped using Permount mounting medium (Fisher Scientific, Fair Lawn, NJ, USA). Detailed reagent preparation and staining conditions are provided in Supplementary Table S1.

#### 2.3.2. Image Acquisition and Morphometric Analysis

The H&E-stained sections were imaged using the BioTek Cytation 5 Imaging Reader (Agilent Technologies, Santa Clara, CA, USA) at 4× magnification, where images were automatically stitched (12 rows × 5 columns) to create one large image. ImageJ (version 1.54g; National Institutes of Health, Bethesda, MD, USA) was then used to perform morphometric analysis on these images. Using the Cytation 5 scale calibration of 2000 µm, a scale of 1212 pixels/2000 µm (0.6060 pixels/µm) was set on ImageJ and standardized for all images. Using the gestational day 18.5 H&E-stained sagittal sections in the Uta Schambra Prenatal Mouse Brain Atlas (Springer, Berlin/Heidelberg, Germany), the hippocampus, lateral ventricle, and cerebellum were identified. These structures were then traced with the freehand selection tool. Using the *colour deconvolution 2* plugin, the H&E stain was selected, which separated the image into three colours (gray, pink, and purple). The purple-only image, the nuclear stain (hematoxylin), was isolated to identify cell density. The image was then inverted, where darker hematoxylin staining corresponded to greater signal intensity after inversion. Three fixed-size background regions of interest (ROIs) (50 × 50 µm) located around the image with no staining artifacts were used to subtract background staining.

For the hippocampus, cerebellum, and background ROIs, the area, mean gray value, and integrated density were recorded. For the lateral ventricle, only area was measured because the ventricle is a fluid-filled cavity and does not contain stained tissue from which optical density could be reliably quantified. Therefore, optical density was not assessed for this region. The mean optical density was calculated by subtracting the average mean gray value of the background ROIs from the mean gray value of the structure of interest. For each sample, the mean optical density measured across all sections was averaged to create a single value per brain region. Volumetric measurements for each structure were calculated using the Cavalieri principle, where the calculated cross-sectional area was multiplied by section thickness (10 µm), and then all sections across the sample were summed. The area of any missing section (due to section damage) was determined by taking the average of the prior and following sections. To account for the remaining four series (10 µm each), the summed value was multiplied by 5, after which the volumes were converted to mm^3^. Each technical replicate (individual section) was not individually analysed but combined into a single value per animal.

### 2.4. Transcriptomic Analysis

#### 2.4.1. Brain Microdissection and RNA Extraction

RNA-seq was performed on 32 fetal brains (*n* = 8 per sex per treatment group) originating from 17 dams. Sham samples included one male and one female fetus from each of eight dams, whereas irradiated samples were selected from nine dams (7 dams contributing one male and one female fetus and 2 dams contributing a single fetus) based on fetal sex, tissue availability, and RNA quality. The fetal brains were removed on ice, and the telencephalon, diencephalon, midbrain, and hindbrain were identified using the Uta Schambra Prenatal Mouse Brain Atlas (Springer, Berlin/Heidelberg, Germany) and the Allen Developing Mouse Brain Atlas (Allen Institute for Brain Science, Seattle, WA, USA). The telencephalon, diencephalon, midbrain, and hindbrain were dissected using a scalpel, with the choroid plexus retained within its associated brain region. RNA was extracted separately from each brain region for each sample using a spin column-based RNA extraction kit (MediRes Corp, Sudbury, ON, Canada; cat. no. G3640-50T) according to the manufacturer’s instructions. Each brain region was placed into a centrifuge tube with 100 mg RNase-free glass beads (MediRes Corp, Sudbury, ON, Canada; cat. no. G0350) and 1 mL of RNA extraction solution (MediRes Corp, Sudbury, ON, Canada; cat. no. G3013-100ML). The tissues were then homogenized using a TissueLyser (QIAGEN, Toronto, ON, Canada) at 30 Hz for 2 min. The aqueous phase was collected and passed through a series of wash buffers within the filter columns to isolate the RNA. The RNA was finally eluted in 20 µL of nuclease-free water. RNA concentration and purity were assessed using a NanoDrop One spectrophotometer (Thermo Fisher Scientific, Waltham, MA, USA).

#### 2.4.2. RNA-Seq Library Preparation and Sequencing

RNA-seq libraries were prepared from 128 brain-region samples in four batches, each balanced across sex, treatment group, and brain region, using the NEBNext^®^ Ultra™ II Directional RNA Library Kit (New England Biolabs, Ipswich, MA, USA; cat. no. E7765S) according to the manufacturer’s protocol, as previously described by Dickinson et al. (2025) [26]. Samples were then quantified via the NEBNext^®^ Library Quant Kit (New England Biolabs, Ipswich, MA, USA; cat. no. E7630S). Indexed libraries were pooled equimolarly for sequencing while retaining individual sample identity and were sequenced together in a single lane of an S4 flow cell on a NovaSeq 6000 Illumina platform (*The Centre for Applied Genomics*, Toronto, ON, Canada) at a median depth of approximately 28.6 million reads per sample (range: 11.4–88.4 million; Supplementary Table S11).

#### 2.4.3. Differential Expression and Functional Enrichment Analysis

Read quality was assessed using *DRAGEN FASTQ Toolkit* (v1.3.1) (BaseSpace, Illumina Inc., San Diego, CA, USA), and reads were aligned to the mouse reference genome (mm10/GRCm38) using STAR aligner within the *RNA-seq Alignment* application (v2.0.2) (BaseSpace, Illumina Inc.). Per-sample sequencing and alignment metrics, including read number, percentage of reads aligned, coverage uniformity, and strand specificity, are provided in Supplementary Table S11. Differential expression analysis was then conducted using *DRAGEN Differential Expression* (v4.3.7) (BaseSpace, Illumina Inc.) with DESeq2 (*Mus musculus* (UCSC mm10) RefSeq gene annotation). For each sex × brain region subset, DESeq2 was fitted with the design formula ∼ condition (sham vs. 1.8 Gy) using default DESeq2 settings (median-of-ratios normalization and Wald test). Irradiated and sham samples were compared separately for each sex and brain region (eight independent comparisons). Library-preparation batch was not included as a covariate because each of the four library batches was balanced across sex, treatment group, and brain region, so that batch was not confounded with treatment; all libraries were also sequenced together on a single flow-cell lane. Each fetus represented one biological replicate; no technical replicates were sequenced. Adjusted *p*-values were computed using the Benjamini–Hochberg method. Genes excluded during DESeq2 independent filtering or Cook’s distance outlier detection were assigned an adjusted *p*-value of NA. Genes were considered differentially expressed at signed fold-change thresholds of ≥+1.5 or ≤−1.5 (equivalent to a log2 fold change of ≥+0.6 or ≤−0.6) and adjusted *p*-value < 0.05. Because each sex was analysed separately, differences in the number of DEGs between sexes are descriptive and do not constitute a formal statistical test of treatment × sex interaction.

Functional and pathway analyses of the RNA-seq data were conducted using iPathwayGuide (AdvaitaBio, Ann Arbor, MI, USA), applying the statistical framework described by Draghici et al. (2007) [27]. Gene ontology (GO) enrichment was assessed against the annotations from the GO Consortium database (April 2025 release), in which DEGs were organized hierarchically within structured GO categories and evaluated using the software’s ‘impact’ and ‘perturbation’ analysis across three domains: biological processes, cellular components, and molecular functions. Cell-type enrichment was performed using the iPathwayGuide cell-type analysis module, which applies an overrepresentation analysis against cell-type gene sets curated in the Molecular Signatures Database (MSigDB). Pathway enrichment was assessed against the KEGG database using global network enrichment analysis, which accounts for both the proportion of DEGs within a pathway and the magnitude, direction, and topological position of each dysregulated gene. Significant pathway enrichment indicates that genes within a pathway are perturbed more than expected by chance; it does not establish whether the pathway is functionally activated or inhibited. Pathway diagrams were overlaid with gene perturbation values, with a log2 fold change threshold of ≥0.6 or ≤−0.6. All *p*-values were corrected using the false discovery rate (FDR) method, and GO terms, cell-type signatures, and pathways were considered significantly enriched at an adjusted *p*-value < 0.05, with the top 10 terms reported for each analysis, ranked by adjusted *p*-value.

#### 2.4.4. RNA-Seq Validation

Complementary DNA (cDNA) was synthesized using the Bi2M-SSRT3 cDNA SuperMix Synthesis Kit (MediRes Corp, Sudbury, ON, Canada; cat. no. Bi2M-SSRT3) according to the manufacturer’s instructions. Briefly, 800 ng of RNA was diluted in nuclease-free water to a volume of 15 μL and incubated at 65 °C for 5 min. Following this, 1 μL of DNase enzyme and 4 μL of 5× cDNA SuperMix (containing proprietary reverse transcriptase, a thermosensitive DNase, an RNase inhibitor, random and oligo(dT)18 primers, dNTPs, and an optimized buffer system) were added and then incubated at 25 °C for 5 min, 42 °C for 15 min, and 85 °C for 30 s.

A subset of genes of interest was selected to validate the accuracy of the RNA sequencing results using RT-qPCR. Genes were chosen based on predefined criteria to ensure biological relevance and reliable expression. Specifically, transcripts were required to have a minimum abundance of ≥30 transcripts per million (TPM), and priority was given to genes that demonstrated dysregulation across multiple brain regions or in both sexes. In addition, genes were selected to represent a range of expression outcomes (increased, decreased, or unchanged), to confirm that RT-qPCR validation captured the full spectrum of transcriptional responses. Based on these criteria, the following genes were selected: *Lyz2*, *Bst2*, *Icam1*, *Psrc1*, *Dnah7b*, *Ttr*, *Tcf7l2*, and *Kcng4*.

Both forward and reverse primers for each gene were designed via the NCBI Primer Blast Tool, with primer design criteria of 50–150 base pairs, predicted melting temperature between 57–63 °C, a GC content of 50–60%, and spanning exon–exon junctions (Table 1) as described by Tharmalingam et al. (2020) [28]. The designed primers were ordered from Integrated DNA Technologies (IDT; Coralville, IA, USA). Primer specificity was confirmed by melt-curve analysis, and amplification efficiency was determined using standard curves generated from serially diluted cDNA. Reaction efficiency was calculated using the equation [Efficiency = 10^(−1/slope)^ − 1]. Primer sets were considered acceptable if amplification efficiency ranged from 90–110% and R^2^ ≥ 0.99, as previously described [29]. Quantitative PCR was performed in 15 µL reactions containing 5 µL of cDNA (10 ng), 7.5 µL of qPCR Master Mix (2×) (MediRes Corp, Sudbury, ON, Canada; cat. no. Bi2M-2xqPCR), 0.9 µL each of forward and reverse primers (600 nM final concentration), and 0.7 µL of nuclease-free water. Reactions were run on a QuantStudio 5 Real-Time PCR System (Thermo Fisher Scientific, Waltham, MA, USA) using SYBR Green-based detection. Cycling conditions consisted of an initial denaturation at 95 °C for 1 min, followed by 40 cycles of 95 °C for 15 s and 60 °C for 30 s. Melt-curve analysis was performed from 60 °C for 10 s to 95 °C for 15 s. Gene fold change was determined by calculating 2^ΔΔCq^, which accounts for the C_q_ values of the housekeeping genes (*Rer1* and *Actb*) and compares expression in the irradiated group with the sham group.

### 2.5. Statistical Analysis

Analysis of the histological data was performed by two-way repeated-measures ANOVA, in which treatment was a between-dam factor and sex was a within-dam (matched) factor. Because each dam contributed one male and one female fetus, this approach accounts for the non-independence of littermates and treats the dam as the independent experimental unit for the treatment comparison (n = 3 dams per treatment group). Post hoc comparisons of sham and irradiated fetuses within each sex, and of male and female fetuses within each treatment group, were performed using Šidák’s multiple comparisons test in GraphPad PRISM 10 (GraphPad Software; San Diego, CA, USA). Data are presented as mean ± SEM.

RT-qPCR data are presented as mean ± SEM and compared across treatment groups using a two-way ANOVA, followed by Tukey’s multiple pairwise comparisons post hoc test in GraphPad PRISM 10 (GraphPad Software; San Diego, CA, USA) to assess treatment and/or sex differences. Statistical approaches for transcriptomic and RT-qPCR analyses have been described previously by Pirkkanen et al. (2019) and Tharmalingam et al. (2020) [28,30].

## 3. Results

### 3.1. Gestational Irradiation Alters Fetal Brain Structure

**Figure 1:**
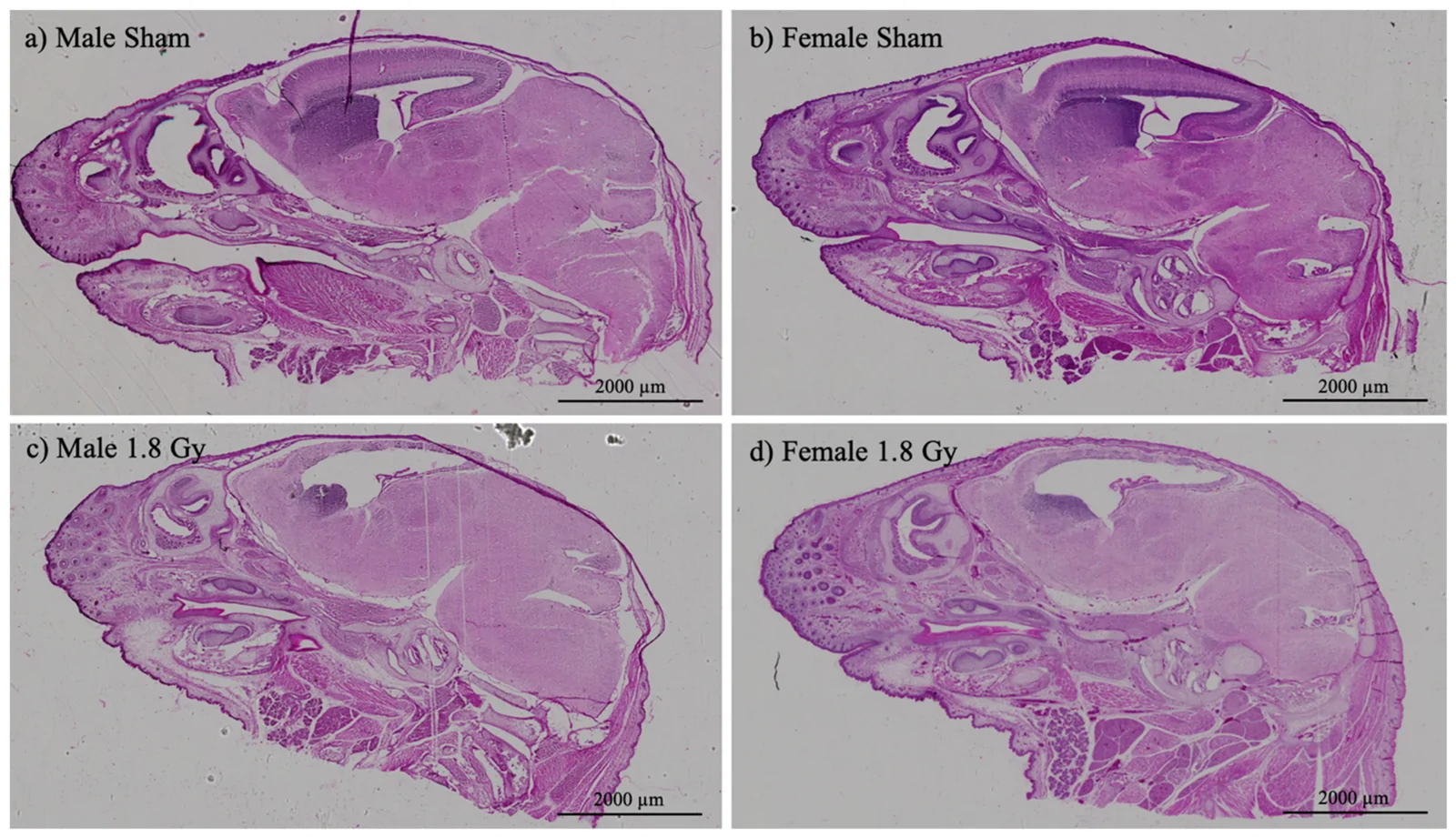
Representative H&E stained BALB/c fetal mouse heads. Panels (**a**) and (**b**) show the sham-exposed fetal head in a male and female mouse, respectively. Panels (**c**) and (**d**) show fetal heads following 1.8 Gy exposure on GD 14.5 in a male and female mouse, respectively.

**Figure 2:**
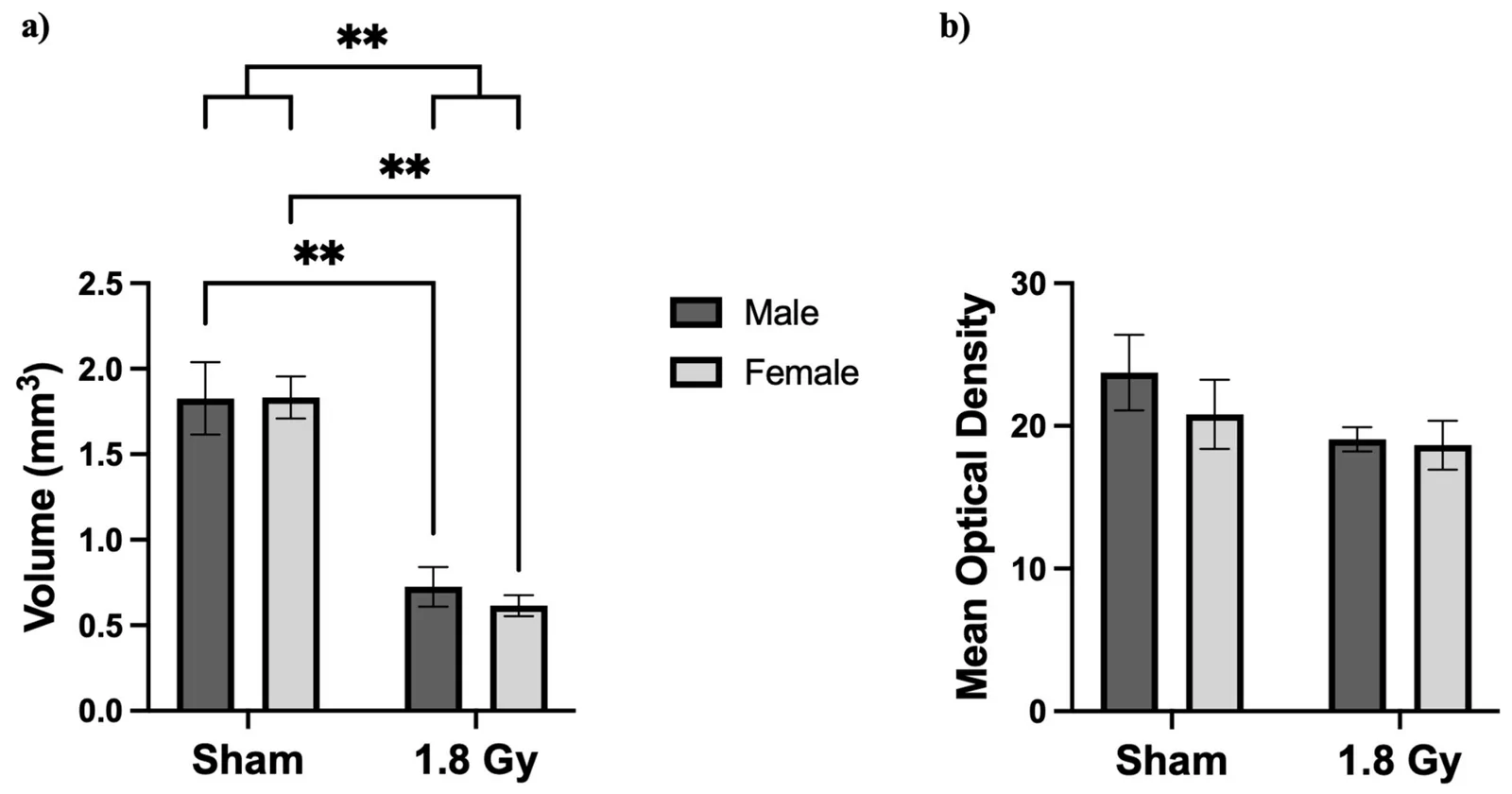
Effects of gestational radiation exposure on BALB/c hippocampal structure. (**a**) Hippocampal volume (mm^3^) measured from H&E-stained sagittal brain sections in male and female sham and 1.8 Gy X-ray exposure on gestational day 14.5 (n = 3 fetuses per sex per treatment group, from 3 sham and 3 irradiated dams). (**b**) Mean optical density of the hippocampus from the same H&E sections. Statistical analysis was performed using two-way repeated-measures ANOVA (sex matched within dam) followed by Šidák’s multiple comparisons test. Data are presented as mean ± SEM (**, *p* < 0.01).

To assess structural changes, the hippocampus, lateral ventricle, and cerebellum were quantified in H&E-stained sagittal sections of fetal brains. Representative images from male and female fetuses for both sham and 1.8 Gy radiation exposure are shown in Figure 1. Hippocampal volume was significantly reduced in the radiation exposure group compared to the control (main effect of treatment, *p*-value = 0.0018), with a mean volume of 1.8 ± 0.1 mm^3^ in the sham group and 0.67 ± 0.06 mm^3^ in the radiation group (Figure 2). Šidák’s post hoc test also identified a significant reduction within each sex. The male sham and radiation groups had mean volumes of 1.8 ± 0.2 mm^3^ and 0.7 ± 0.1 mm^3^, respectively (adjusted *p*-value = 0.0020). The female sham and radiation groups had mean volumes of 1.8 ± 0.1 mm^3^ and 0.61 ± 0.06 mm^3^, respectively (adjusted *p*-value = 0.0010). There was no difference between the sexes (main effect of sex, *p*-value = 0.6785), with mean volumes of 1.3 ± 0.3 mm^3^ for males and 1.2 ± 0.3 mm^3^ for females. The mean optical density was also determined for the hippocampus (Figure 2). The hippocampal mean optical density was not significantly altered following radiation exposure (main effect of treatment, *p*-value = 0.1733), or between sexes (main effect of sex, *p*-value = 0.4509).

**Figure 3:**
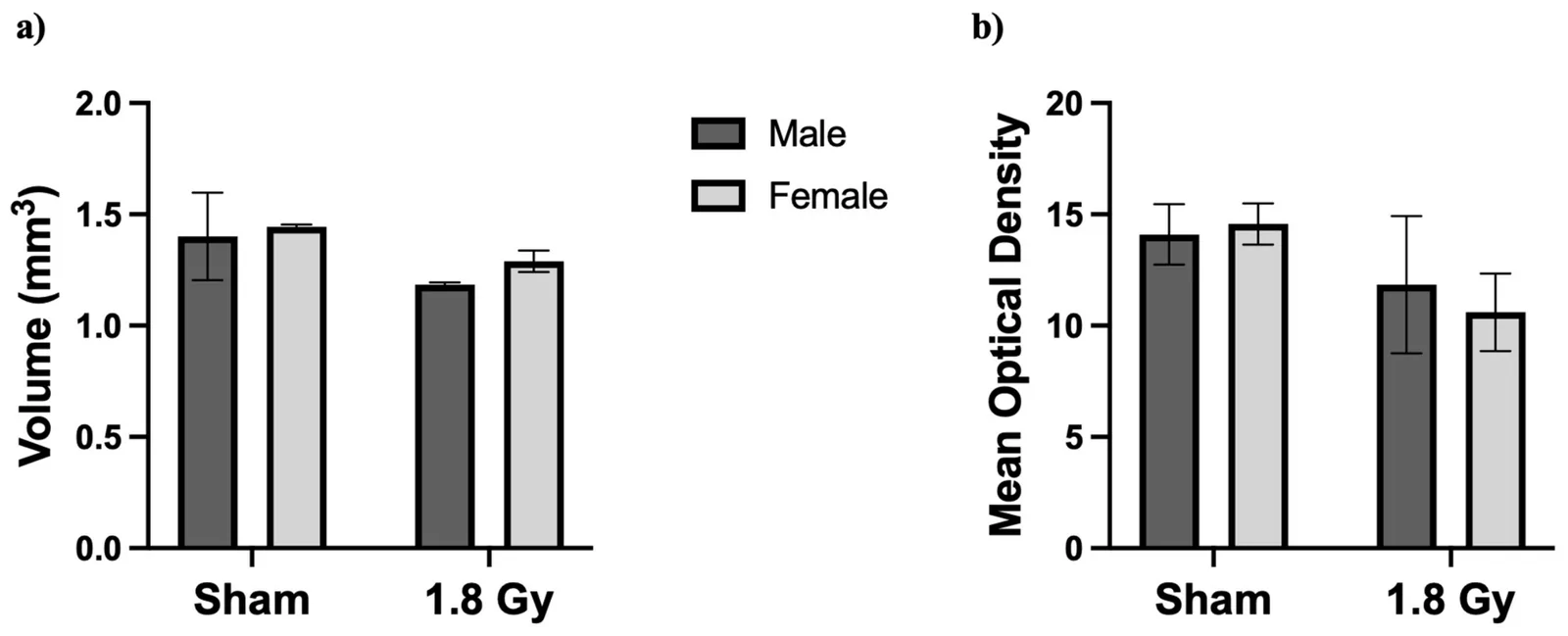
Effects of gestational radiation exposure on BALB/c cerebellar structure. (**a**) Cerebellar volume (mm^3^) measured from H&E-stained sagittal brain sections in male and female sham and 1.8 Gy X-ray exposure on gestational day 14.5 (n = 3 fetuses per sex per treatment group, from 3 sham and 3 irradiated dams). (**b**) Mean optical density of the cerebellum from the same H&E sections. Statistical analysis was performed using two-way repeated-measures ANOVA (sex matched within dam) followed by Šidák’s multiple comparisons test. Data are presented as mean ± SEM.

No significant difference in cerebellar volume was observed between treatment groups (main effect of treatment, *p*-value = 0.1252) or sexes (main effect of sex, *p*-value = 0.5229) (Figure 3). The mean volume was 1.42 ± 0.09 mm^3^ for the sham group and 1.24 ± 0.03 mm^3^ for the radiation group. The mean volumes were 1.3 ± 0.1 mm^3^ for males and 1.37 ± 0.04 mm^3^ for females. Šidák’s post hoc test indicated no significant difference within each sex, with adjusted *p*-values of 0.5241 and 0.7745 in males and females, respectively. Similar to the volume, no significant difference in optical density was observed between the sexes or treatment groups (main effect of treatment, *p*-value = 0.1426; main effect of sex, *p*-value = 0.8689) (Figure 3). The mean optical density within the sham group was 14.3 ± 0.8 and was 11 ± 2 for the radiation group. The mean optical density was 13 ± 2 for males and 13 ± 1 for females.

**Figure 4:**
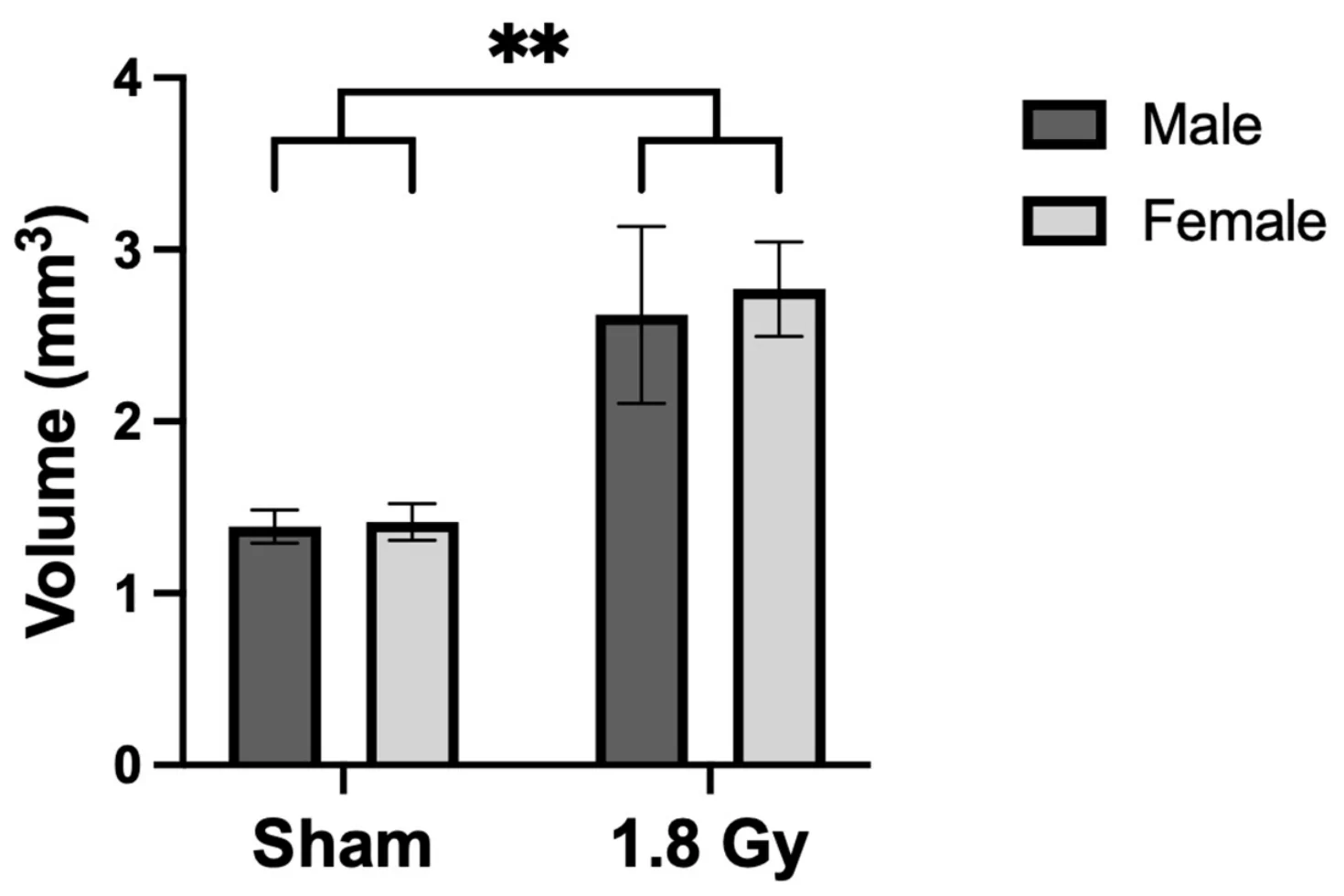
Effects of gestational radiation exposure on BALB/c lateral ventricle volume. Lateral ventricle volume (mm^3^) measured from H&E-stained sagittal brain sections in male and female sham and 1.8 Gy X-ray exposure on gestational day 14.5 (n = 3 fetuses per sex per treatment group, from 3 sham and 3 irradiated dams). Statistical analysis was performed using two-way repeated-measures ANOVA (sex matched within dam) followed by Šidák’s multiple comparisons test. Data are presented as mean ± SEM (**, *p* < 0.01).

The volume of the lateral ventricle was also calculated (Figure 4). A significant increase in volume was observed following radiation exposure (main effect of treatment, *p*-value = 0.0324), with a mean volume of 1.40 ± 0.07 mm^3^ for the sham group and 2.7 ± 0.3 mm^3^ for the radiation group. However, Šidák’s post hoc test did not identify significant within-sex differences, with adjusted *p*-values of 0.0772 and 0.0501 in males and females, respectively. No significant treatment × sex interaction was detected for any histological measure (all *p*-values > 0.56).

### 3.2. Gestational Irradiation Is Associated with Region-Specific Transcriptional Dysregulation

#### 3.2.1. Differential Gene Expression Across Fetal Brain Regions

**Figure 5:**
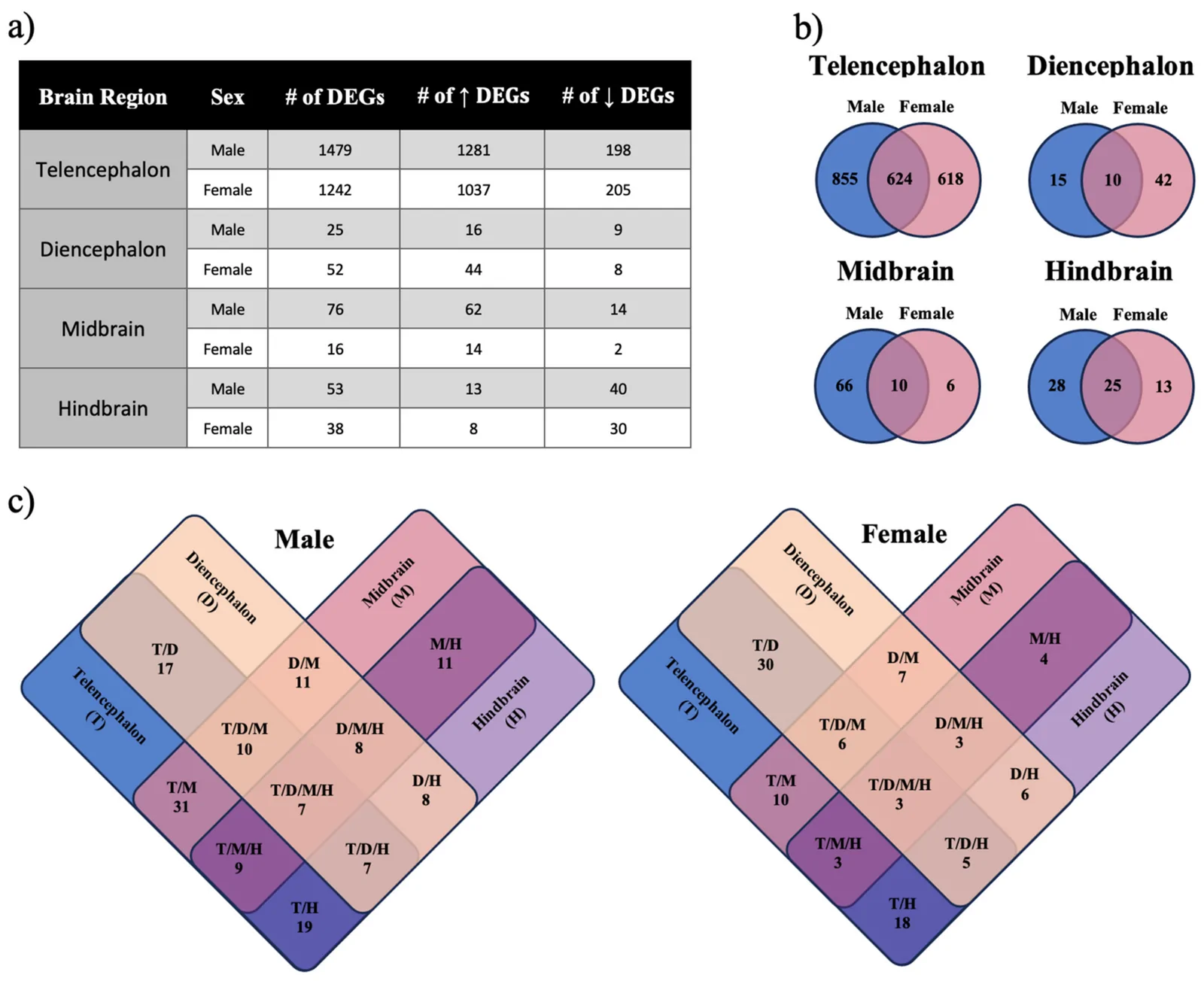
Comparison of differentially expressed genes (DEGs) between males and females following gestational radiation exposure. (**a**) Table summarizes total number of DEGs per brain region and sex, as well as the number of upregulated and downregulated DEGs following RNA-seq transcriptomic analysis (n = 8 per sex per treatment group, one fetus per sex per dam; sham: 8 dams, irradiated: 9 dams). (**b**) Venn diagrams show the number of similar and unique DEGs identified between the sexes in the telencephalon, diencephalon, midbrain, and hindbrain. (**c**) Venn diagrams show the number of similar DEGs between the telencephalon (T), diencephalon (D), midbrain (M), and hindbrain (H). DEGs were identified as significant with a fold change ≥ +1.5 or ≤−1.5 and adjusted *p*-value < 0.05.

RNA-seq of the telencephalon, diencephalon, midbrain, and hindbrain was conducted to identify region-specific transcriptional dysregulation. In the male telencephalon, there were a total of 1479 DEGs, with 1281 upregulated and 198 downregulated (Figure 5). In the female telencephalon, 1242 DEGs were identified, with 1037 upregulated and 205 downregulated (Figure 5). The complete lists of DEGs, ranked by fold change, are provided in Supplementary Table S2 (male) and Supplementary Table S3 (female). In both sexes, the most highly upregulated gene was *Gpnmb* (glycoprotein nmb; male fold change = +15.80, female fold change = +38.39). The most downregulated gene for males was *Gm10719* (fold change = −10.60), whereas in females it was *Msx3* (msh homeobox 3; fold change = −15.01). Because males and females were analysed separately, comparisons of DEG lists between sexes are descriptive and do not test a treatment × sex interaction (Section 2.4.3). Out of the 1479 male DEGs and 1242 female DEGs in the telencephalon, 624 were shared between sexes, while 855 were unique to males and 618 were unique to females (Supplementary Table S10).

Within the male diencephalon, there was a total of 25 DEGs, with 16 upregulated and 9 downregulated (Figure 5). In the female diencephalon, 52 DEGs were identified, with 44 upregulated and 8 downregulated (Figure 5). The complete lists of DEGs, ranked by fold change, are provided in Supplementary Table S4 (male) and Supplementary Table S5 (female). The most highly upregulated gene for males was *Gpnmb* (fold change = +4.30), whereas in females it was *Gm10273*, a predicted gene with negligible expression in sham controls, which yielded a disproportionately large fold change (Supplementary Table S5). The most downregulated gene for males was *Gm9780* (fold change = −5.88), whereas in females it was *Crybb1* (crystallin, beta B1; fold change = −3.99). Out of the 25 DEGs within the male diencephalon and 52 DEGs in the female diencephalon, only 10 genes were found to be dysregulated in both sexes, with 15 being unique to males and 42 to females (Figure 5; Supplementary Table S10).

In the male midbrain, there was a total of 76 DEGs, with 62 upregulated and 14 downregulated (Figure 5). Within the female midbrain, 16 DEGs were identified, with 14 upregulated and 2 downregulated (Figure 5). The complete lists of DEGs, ranked by fold change, are provided in Supplementary Table S6 (male) and Supplementary Table S7 (female). The most upregulated gene for males was *Gm21378* (fold change = +144.58), a predicted gene with negligible expression in sham controls (0.19 CPM; Supplementary Table S6), whereas for females it was *Eda2r* (ectodysplasin A2 receptor; fold change = +4.44). The most highly downregulated gene for males was *Ttr* (transthyretin; fold change = 16.03), whereas in females it was *P2ry12* (purinergic receptor P2Y, G-protein coupled 12; fold change = −2.09). Out of the 76 DEGs within the male midbrain and 16 DEGs in the female midbrain, only 10 genes were found to be dysregulated in both sexes, with 66 being unique to males and 6 to females (Supplementary Table S10).

In the male hindbrain, there was a total of 53 DEGs, with 13 upregulated and 40 downregulated (Figure 5). Within the female hindbrain, 38 DEGs were identified, with 8 upregulated and 30 downregulated (Figure 5). The complete lists of DEGs, ranked by fold change, are provided in Supplementary Table S8 (male) and Supplementary Table S9 (female). The most upregulated gene for males was *Flg* (filaggrin; fold change = +34.05), which was expressed at low levels in sham controls (Supplementary Table S8), whereas in females it was *Gpnmb* (fold change = +4.74). In both sexes, the most highly downregulated gene was *Mobp* (myelin-associated oligodendrocyte basic protein; male fold change = −34.20, female fold change = −20.56). Out of the 53 DEGs within the male hindbrain and 38 DEGs in the female hindbrain, only 25 genes were found to be dysregulated in both sexes, with 28 being unique to males and 13 to females (Supplementary Table S10).

Comparing DEGs across all brain regions, the telencephalon exhibited the greatest degree of overlap. In males, the telencephalon shared 17 DEGs with the diencephalon, 31 with the midbrain, and 19 with the hindbrain, whereas in females it shared 30 DEGs with the diencephalon, 10 with the midbrain, and 18 with the hindbrain (Figure 5). A subset of DEGs was also shared across all four brain regions. Three DEGs, *P2ry12*, *Siglec1* (sialic acid binding Ig like lectin 1, sialoadhesin), and *Eda2r*, were common to all regions in both sexes. In males, four additional DEGs were shared across all brain regions, namely *Crybb1*, *Gpnmb*, *Lyz2* (lysozyme 2), and *Ighm* (immunoglobulin heavy constant mu), resulting in a total of seven whole-brain overlapping DEGs in males.

#### 3.2.2. RNA-Seq Validation

The RNA-seq findings were validated by RT-qPCR in a subset of genes selected as described in Section 2.4.4 (Table 2 and Supplementary Figures S9 and S10). Fold changes from RNA-seq and RT-qPCR are compared in Table 2. RT-qPCR confirmed the direction and significance pattern of the RNA-seq findings for all significantly dysregulated transcripts, and transcripts that were unchanged by RNA-seq likewise showed no significant change by RT-qPCR (Table 2).

#### 3.2.3. Gene Ontology Enrichment Analysis

**Figure 6:**
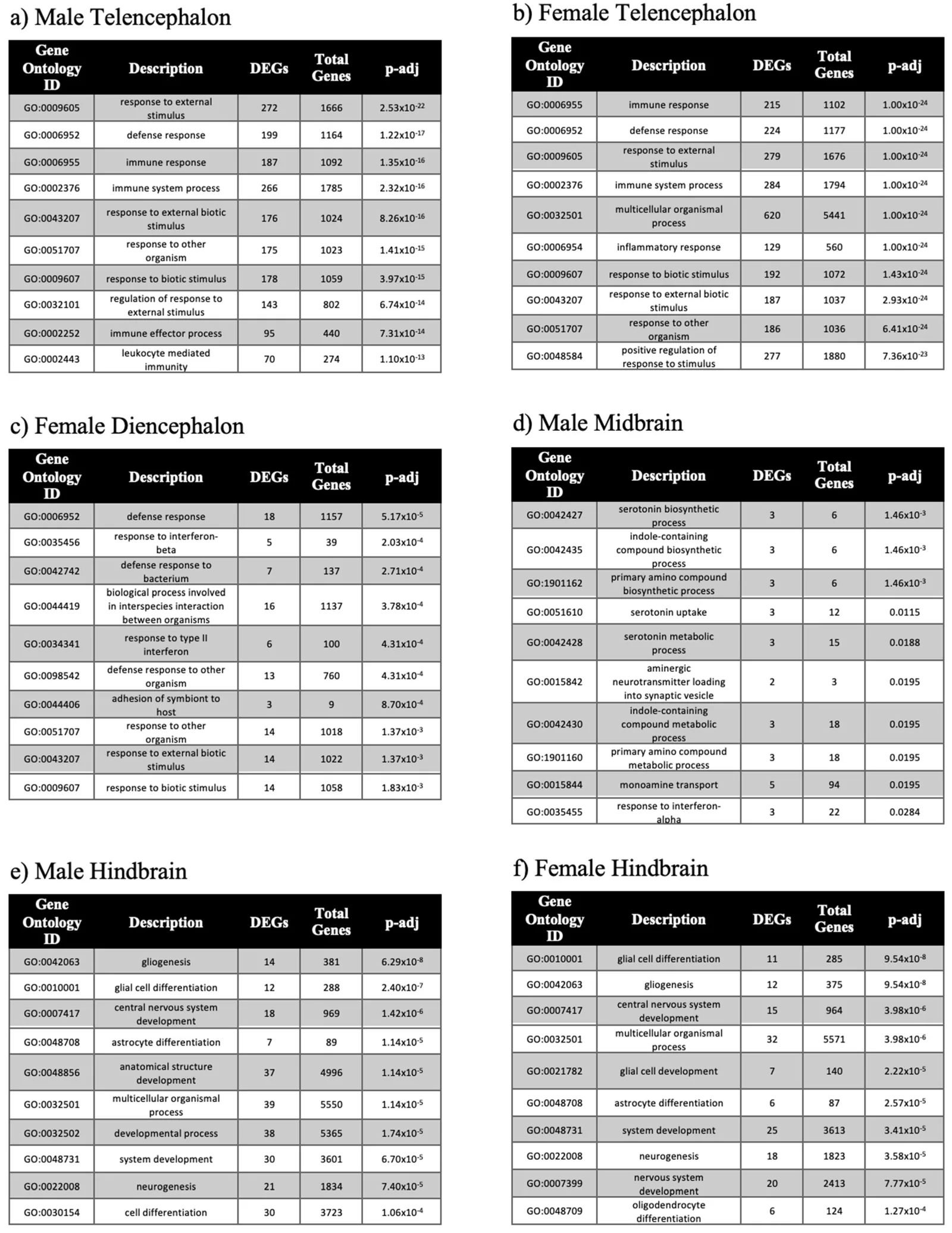
Gene ontology analysis of biological processes following gestational radiation exposure. The top 10 enriched GO terms for (**a**) male telencephalon, (**b**) female telencephalon, (**c**) female diencephalon, (**d**) male midbrain, (**e**) male hindbrain, and (**f**) female hindbrain are shown, ranked by FDR-adjusted *p*-value. Only terms with an FDR-adjusted *p*-value < 0.05 are displayed (n = 8 per sex per treatment group, one fetus per sex per dam; sham: 8 dams, irradiated: 9 dams). The number of DEGs and the total number of genes within each GO term are indicated, where “Total Genes” represents all genes annotated to the ontology category, and “DEGs” represents the subset that was significantly dysregulated.

**Figure 7:**
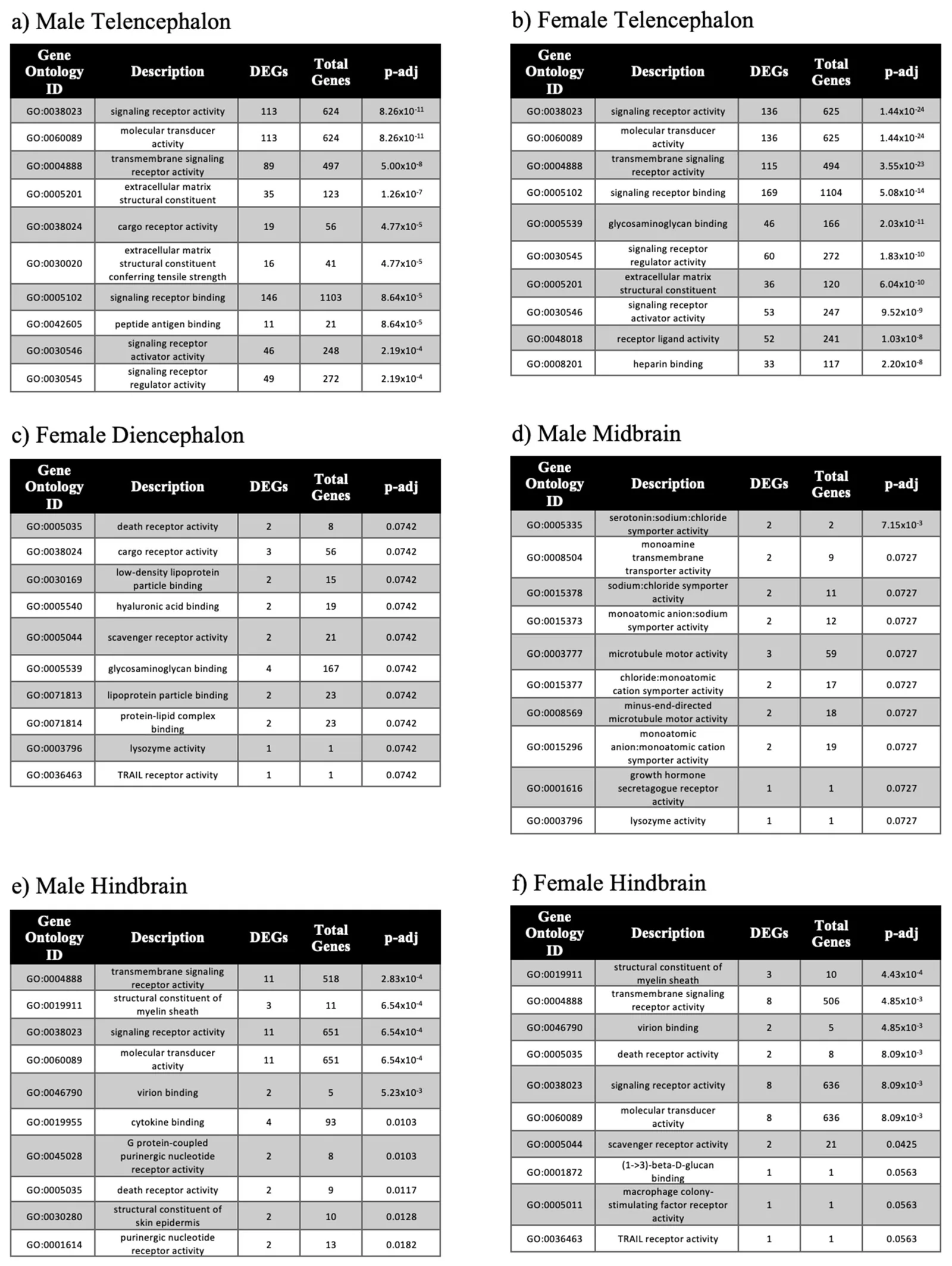
Gene ontology analysis of molecular functions following gestational radiation exposure. The top 10 enriched GO terms for (**a**) male telencephalon, (**b**) female telencephalon, (**c**) female diencephalon, (**d**) male midbrain, (**e**) male hindbrain, and (**f**) female hindbrain are shown, ranked by FDR-adjusted *p*-value. Only terms with an FDR-adjusted *p*-value < 0.05 are displayed (n = 8 per sex per treatment group, one fetus per sex per dam; sham: 8 dams, irradiated: 9 dams). The number of DEGs and the total number of genes within each GO term are indicated, where “Total Genes” represents all genes annotated to the ontology category, and “DEGs” represents the subset that was significantly dysregulated.

Gene ontology (GO) enrichment analysis was performed on DEGs from the telencephalon (male and female), diencephalon (female), midbrain (male), and hindbrain (male and female), identifying the biological processes, cellular components, and molecular functions most affected by gestational radiation exposure. The top 10 enriched GO terms are presented for each category: biological processes (Figure 6), cellular components (Supplementary Figure S1), and molecular functions (Figure 7), ranked by adjusted *p*-value (*p*-adj).

The top enriched GO biological processes in the telencephalon for both sexes were predominantly associated with stimulus and immune responses, including *defence response*, *immune response*, and *response to external biotic stimulus* (Figure 6). In the female diencephalon, enriched GO terms were largely related to interferon-mediated signalling, such as *response to interferon-beta* and *response to type II interferon* (Figure 6). Within the male midbrain, enriched GO biological processes were associated with serotonin, including *serotonin biosynthetic process*, *serotonin uptake*, and *serotonin metabolic process* (Figure 6). The hindbrain, for both sexes, exhibited significant enrichment in GO terms related to glial cell development, such as *gliogenesis*, *glial cell differentiation*, and *astrocyte and oligodendrocyte differentiation* (Figure 6). Additionally, neurogenesis was among the significantly enriched biological processes in both sexes of the hindbrain.

GO cellular component enrichment is summarized in Supplementary Figure S1. In both sexes, telencephalon terms were predominantly associated with cell periphery structures (e.g., *extracellular space*, *extracellular region*, *extracellular matrix*). The female diencephalon was enriched for intracellular components, and the male midbrain for transport and motility-related terms (e.g., *axonemal dynein complex*). In the hindbrain, males showed enrichment in peripheral membrane structures, while females displayed greater enrichment in axonal and myelin-associated components (e.g., *myelin sheath*, *main axon*).

The top enriched GO molecular functions for the telencephalon for both sexes were predominantly associated with receptor-related activity, such as *signal receptor activity*, *signal receptor binding*, and *signal receptor regulator activity* (Figure 7). In the female diencephalon, no molecular functions were significantly affected by radiation exposure (Figure 7). In the male midbrain, the only significantly enriched molecular function was *serotonin/sodium/chloride symporter activity* (Figure 7). The hindbrain, for both sexes, exhibited significance in GO terms related to receptor-mediated processes, including *signalling receptor activity*, *death receptor activity*, and *molecular transducer activity* (Figure 7).

#### 3.2.4. Cell-Type Enrichment Analysis

Cell-type enrichment identified dysregulated transcriptional signatures among the DEGs (Supplementary Figure S2). Within both the male and female telencephalon and hindbrain, dysregulation in various microglia gene sets was observed. In the female diencephalon, one microglia set was dysregulated. Within the male midbrain, radiation exposure was associated with dysregulation of astrocyte-associated genes. In the hindbrain, alongside microglia dysregulation, there was also dysregulation in oligodendrocytes and Schwann cells, both of which are associated with myelin sheath formation.

#### 3.2.5. KEGG Pathway Enrichment Analysis

**Figure 8:**
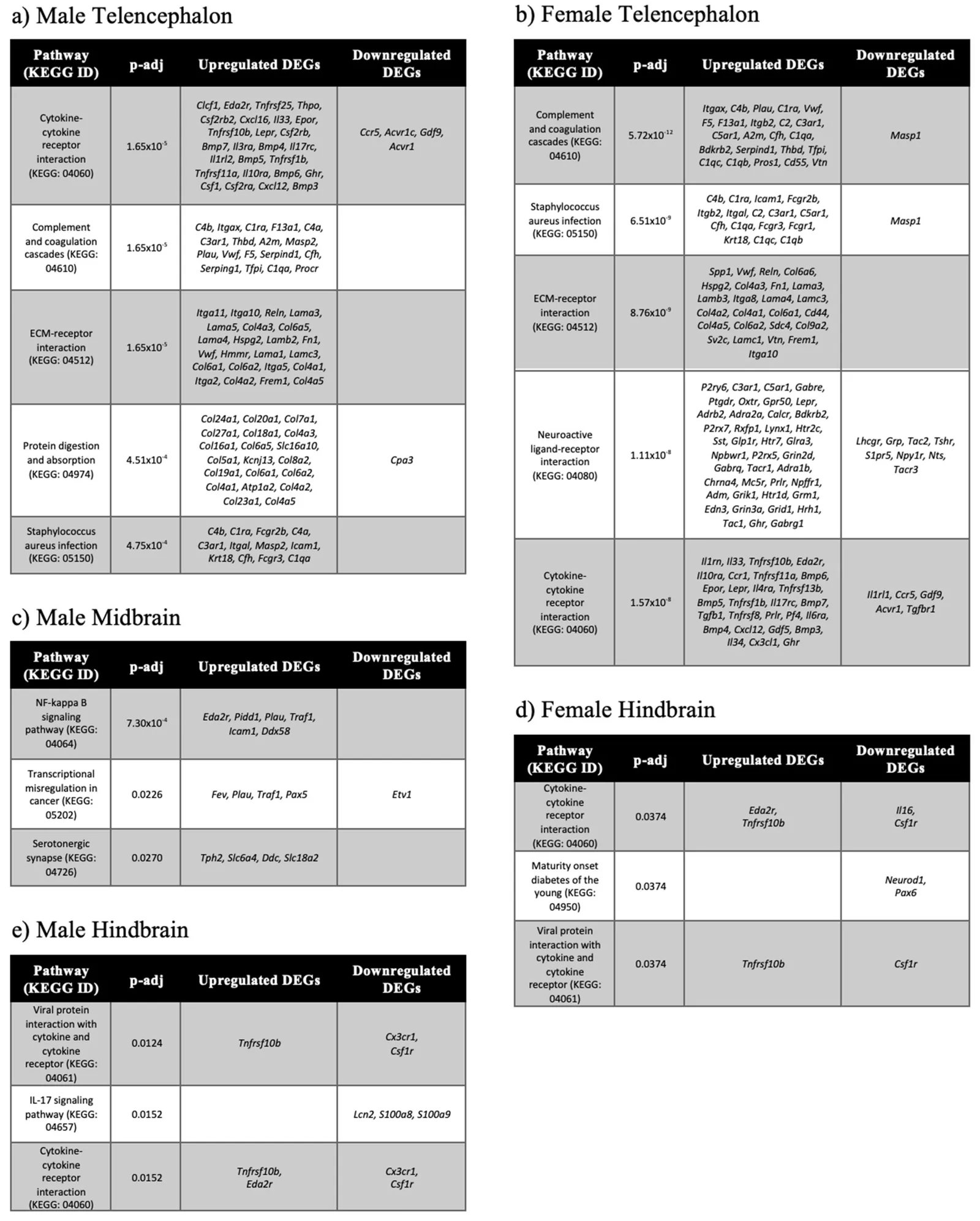
KEGG pathway enrichment analysis of DEGs across different brain regions following gestational radiation exposure. The top enriched signalling pathways, (**a**) male telencephalon, (**b**) female telencephalon, (**c**) male midbrain,(**d**) female hindbrain, (**e**) male hindbrain, are shown, ranked by FDR-adjusted *p*-value. Only pathways with an FDR-adjusted *p*-value < 0.05 are displayed (n = 8 per sex per treatment group, one fetus per sex per dam; sham: 8 dams, irradiated: 9 dams). The upregulated and downregulated genes in each pathway are also indicated.

Pathway enrichment identified dysregulated molecular signalling pathways in the irradiated fetal brains, with significant pathways detected in each brain region. Within the telencephalon, four of the top five dysregulated pathways were shared between males and females (Figure 8). These pathways included *complement and coagulation cascade* (male: FDR-adjusted *p* = 1.65 × 10^−5^, female: FDR-adjusted *p* = 5.72 × 10^−12^) (Supplementary Figure S3), *cytokine–cytokine receptor interaction* (male: FDR-adjusted *p* = 1.65 × 10^−5^, female: FDR-adjusted *p* = 1.57 × 10^−8^) (Supplementary Figure S4), *ECM–receptor interaction* (male: FDR-adjusted *p* = 1.65 × 10^−5^, female: FDR-adjusted *p* = 8.76 × 10^−9^) (Supplementary Figure S5), and *Staphylococcus aureus infection* (male: FDR-adjusted *p* = 4.75 × 10^−4^, female: FDR-adjusted *p* = 6.51 × 10^−5^).

The *complement and coagulation cascade* (KEGG: 04610) was among the top enriched pathways identified in the telencephalon following gestational radiation exposure. In males, the pathway showed widespread upregulation across both the complement and coagulation arms, with a similar pattern in females except for downregulated *Masp1/2* (Supplementary Figure S3). The *cytokine–cytokine receptor interaction* pathway (KEGG: 04060) was also significantly dysregulated, with broad transcriptional activation of cytokine and receptor genes, including *TNF*, *IL1*, *IL6*, and CXCL family members, which were more prominent in males (Supplementary Figure S4). Downregulated genes were also observed in this pathway, especially within the TGF-β family receptors.

The *ECM–receptor interaction* pathway (KEGG: 04512) was also significantly dysregulated. Visualization of the pathway revealed broad upregulation of genes associated with ECM structure and cell–matrix adhesion, including members of the *collagen*, *laminin*, and *fibronectin* families (Supplementary Figure S5). Both sexes exhibited similar transcriptional profiles, although expression levels appeared higher in males. The final dysregulated pathway in the male telencephalon was *protein digestion and absorption* (FDR-adjusted *p* = 4.51 × 10^−4^) (Figure 8). This pathway was also significantly dysregulated within the female telencephalon (FDR-adjusted *p* = 9.80 × 10^−5^) but did not rank within the top five. The *neuroactive ligand–receptor interaction* pathway was significantly dysregulated in the female telencephalon (FDR-adjusted *p* = 1.11 × 10^−8^), whereas no significant effect was observed in males (FDR-adjusted *p* = 0.167) (Figure 8). Most DEGs within these pathways were upregulated.

Within the female diencephalon, no pathways met the significance criteria. Within the male midbrain, only three dysregulated pathways were observed: *NF-κB signalling pathway* (FDR-adjusted *p* = 7.30 × 10^−4^), *transcriptional misregulation in cancer* (FDR-adjusted *p* = 0.0226), and *serotonergic synapse* (FDR-adjusted *p* = 0.0270) (Figure 8). The *NF-κB signalling* pathway (KEGG: 04064) revealed upregulation of the genes *Xedar*, *Pidd*, *uPa*, *Traf1/2*, *Icam*, and *Rig1* (Supplementary Figure S6). The *serotonergic synapse* pathway (KEGG: 04726) was also significantly enriched, with upregulation of genes associated with serotonin production, release, and reuptake, such as *Tph*, *Ddc*, *Vmat*, and *Sert* (Supplementary Figure S7).

Within the hindbrain, three pathways per sex met the significance criteria. Similar to the telencephalon, dysregulation of the *cytokine–cytokine receptor interaction* in both males (FDR-adjusted *p* = 0.0152) and females (FDR-adjusted *p* = 0.0374) was observed (Figure 8). In contrast to the telencephalon, this pathway (KEGG: 04060) showed substantially less dysregulation, with an even balance between upregulated and downregulated transcripts (Supplementary Figure S8). Both sexes shared upregulation of *Dr4*, *Dr5*, and *Xedar* and downregulation of *Csf1r*, with downregulation of *IL16* detected only in females and *Cx3cr1* only in males. Both sexes also shared dysregulation of the *viral protein interaction with cytokine and cytokine receptor* pathway (male: FDR-adjusted *p* = 0.0124, female: FDR-adjusted *p* = 0.0374) (Figure 8). The only other pathway dysregulated in the male hindbrain was the *IL-17 signalling pathway* (FDR-adjusted *p* = 0.0152), while the only other pathway dysregulated in the female hindbrain was *maturity-onset diabetes of the young* (FDR-adjusted *p* = 0.0374).

## 4. Discussion

The goal of the study was to determine whether high-dose gestational radiation exposure during late fetal development induces region-specific structural and transcriptomic alterations in the developing mouse brain. Structural analysis revealed a reduction in hippocampal volume and an increase in lateral ventricle volume, with no significant effects observed in the cerebellum. Transcriptomic profiling demonstrated widespread dysregulation of immune-related genes, along with alterations in pathways associated with glial function, extracellular matrix organization, cell migration, and neurotransmitter signalling. Together, these findings indicate that high-dose gestational irradiation produces region-specific structural disruption accompanied by transcriptional changes consistent with immune/inflammatory activation, altered gliogenesis, migration-associated pathway dysregulation, and neurotransmitter-related transcriptional changes.

### 4.1. Region-Specific Structural Effects of Gestational Irradiation

Histological analysis was conducted to examine region-specific structural alterations in the fetal brain following radiation exposure. The pronounced reduction in hippocampal size observed here aligns with prior reports of radiation-induced hippocampal vulnerability. Earlier postnatal rodent studies showed significant reductions in dentate gyrus volume and proliferative cell populations, along with increased apoptosis, following radiation exposure of 2 Gy or higher [31,32,33]. Humans exposed to 40 Gy irradiation during targeted radiotherapy exhibited a 78% reduction in hippocampal volume, providing further support [34].

Although the present study did not reveal a significant decrease in hippocampal cell density, this does not exclude localized cellular loss. Optical density measurements were derived from hematoxylin staining rather than the cresyl violet used in prior studies, which may account for the discrepancy [31,33]. Moreover, the reduced hippocampal size likely made it difficult to distinguish cell-dense subregions such as CA1, CA2, and the dentate gyrus, so optical density averaged across the whole hippocampus may underestimate localized loss [35,36]. The histological sample size in the present study (n = 3 per sex per treatment group) was smaller than in previous reports, which used group sizes of 4–20 animals. Nonetheless, the reduction in hippocampal volume was significant within each sex analysed separately and likely reflects the vulnerability of neural stem and progenitor populations within the dentate gyrus [37,38].

Although this progenitor-driven sensitivity persists across the lifespan [16], the developmental stage at exposure likely determines its structural consequence. Gestational day 14.5 corresponds to a critical neurodevelopmental window in mice, as neuronal proliferation and migration intensify after GD 10 [39,40]. Disruption of progenitor division or migration within neurogenic regions, including the subgranular zone (SGZ) of the dentate gyrus and the subventricular zone (SVZ) of the lateral ventricle, may therefore underlie the reduced hippocampal volume [20,22,41]. Because the hippocampus is critical for learning and memory, this change may carry functional consequences if it persists postnatally [35,36]. Consistent with this, human studies report hippocampal atrophy with memory and learning impairments following fractionated cranial irradiation [34,42,43].

The enlargement of the lateral ventricles observed in radiation-exposed fetuses further supports the occurrence of neurodevelopmental disruption. Prior studies attribute such ventricular dilation to reduced progenitor proliferation and migration, and increased apoptosis within the SVZ [37,44,45,46]. Although a TUNEL assay was not performed, increased apoptosis in the SVZ and adjacent hippocampal tissue, previously reported following radiation exposure, may have contributed. The enlargement itself also hindered reliable identification of the SVZ [45,47,48]. Impaired cerebrospinal fluid (CSF) circulation may also play a role, as radiation affects the CSF system [44,49]. Consistent with this, *Ttr*, which within the brain is synthesized by the choroid plexus and transports thyroxine into the CSF [50], was upregulated in the telencephalon of both sexes (Table 2), suggesting altered activity of the choroid plexus associated with the enlarged lateral ventricles. In contrast, *Ttr* was strongly downregulated in the male midbrain. However, the midbrain lacks its own choroid plexus, and the baseline *Ttr* expression was an order of magnitude lower. This change more likely reflects variable inclusion of adjacent choroid plexus during microdissection and should be interpreted with caution. Because thyroid hormone is essential for fetal neurodevelopment, future assessment of choroid plexus structure, TTR protein, and CSF composition would clarify whether gestational radiation impairs choroid plexus function. Taken together, the ventricular enlargement may reflect a combination of progenitor loss, disrupted migration, and possibly impaired CSF circulation, disturbances associated in both animals and humans with slower learning, reduced verbal fluency, and visual processing deficits [51,52,53].

In contrast to the hippocampus and lateral ventricles, no significant change in cerebellar volume or optical density was detected at GD 18.5. This differs from reports in which earlier gestational irradiation caused substantial cerebellar volume loss and increased cell death in the external germinal layer [46,54]. The difference likely reflects the mouse neurodevelopmental timeline, as cerebellar neurogenesis begins around GD 12 but peaks postnatally, continuing until roughly 20 days after birth [9,55,56,57,58]. GD 14.5 irradiation may therefore have preceded the period of greatest cerebellar vulnerability, though delayed postnatal effects remain possible as radiation-induced cerebellar changes in adulthood have been linked to deficits in cognition, learning, and social behaviour [59,60].

Overall, these histological findings indicate that high-dose gestational radiation exposure induces region-specific alterations in the fetal brain, with the strongest effects in structures associated with active neurogenesis and ventricular organization, providing an anatomical framework for interpreting the transcriptomic alterations identified across fetal brain regions. Structural changes were assessed on H&E-stained sections, which permit regional volumetric and density measurements but not identification of specific neuronal populations. Cortical layer organization was therefore not evaluated, and confirmation of cell-type-specific changes will require immunohistochemical characterization in future studies.

### 4.2. Region-Specific Transcriptomic Responses in the Fetal Brain

Transcriptomic profiling revealed extensive gene dysregulation across the telencephalon, diencephalon, midbrain, and hindbrain in both sexes. The strongest response was observed in the telencephalon, with smaller but biologically relevant changes in the remaining regions. Dysregulated genes and enriched pathways were primarily associated with immune function, glial activity, and neuronal homeostasis, suggesting that radiation-induced molecular disruption may contribute to the structural vulnerabilities observed.

#### 4.2.1. Immune and Inflammatory Transcriptional Responses

A prominent effect of gestational radiation exposure was the dysregulation of immune-related genes. Upregulation of *Gpnmb* in all four brain regions of males, and in the telencephalon, diencephalon, and hindbrain of females, was consistent with a transcriptional signature of microglial activation, mirroring findings in adult rats exposed to 14 Gy of X-rays, in which increased microglial proliferation accompanied *Gpnmb* upregulation [61,62].

Beyond *Gpnmb*, pathway analysis identified several significantly affected immune-related pathways, including cytokine–cytokine receptor interaction, complement and coagulation cascades, and NF-κB signalling. Dysregulation of the cytokine–cytokine receptor interaction pathway was most pronounced in the telencephalon, likely reflecting its heightened radiosensitivity due to proliferative zones such as the SGZ of the dentate gyrus and the SVZ of the lateral ventricle (Figure 8). Most affected genes were upregulated, while a smaller subset was downregulated, possibly reflecting a compensatory process (Supplementary Figure S4). This pathway was also affected in the hindbrain, though with fewer DEGs. A similar pattern appeared in the *complement and coagulation cascade* pathway, where nearly all dysregulated genes were upregulated with only one downregulated gene in the female telencephalon (Supplementary Figure S3). The coordinated upregulation of genes within these pathways is consistent with an inflammatory transcriptional response in the fetal brain, although inflammatory activation at the cellular or protein level was not measured in this study. In support of these changes, inflammatory and innate immune response pathways were also reported in placentas from the same irradiation model, including *S. aureus* infection and systemic lupus erythematosus [23]. The placental response, however, was predominantly downregulation, in contrast to the upregulation observed in this study. Despite this directional difference, the overlap supports immune dysregulation across the fetoplacental unit following in utero X-ray exposure, with organ-specific magnitude and direction.

Gene ontology (GO) enrichment analysis revealed significant dysregulation in immune-related processes, including defence response, immune response, leucocyte-mediated immunity, and inflammatory response. Consistent with these results, cell-type enrichment indicated dysregulation of microglia and macrophages within the telencephalon and hindbrain, aligning with previous literature in young mice exposed to high-dose radiation, with effects observed up to one month after exposure [15]. Microglial marker dysregulation, including downregulation of *P2ry12* across all four regions in both sexes and downregulation of *Cx3cr1* in the male hindbrain, is likewise consistent with altered *Cx3cr1* and *Tmem119* expression reported in adult C57Bl/6J mice exposed in utero at GD 15 to considerably lower doses (50–300 mGy) [13]. Although strain, dose, and age at analysis differed substantially, the recurrence of microglial marker dysregulation suggests that microglia may represent a persistently sensitive target of prenatal irradiation of the brain. *P2ry12* encodes a purinergic receptor that is selectively expressed by homeostatic microglia and mediates their process extension towards sites of injury, and its expression is markedly reduced upon microglial activation [63]. Conversely, Siglec1 (CD169), a marker of border-associated macrophages of the meninges, perivascular spaces, and choroid plexus [64], was upregulated in all four regions of both sexes, alongside interferon-stimulated genes such as *Bst2*, *Ifit1*, and *Ifitm3*. These opposing changes suggest that fetal brain myeloid cells shift away from a homeostatic microglial state towards an activated, interferon-responsive profile and/or that border-associated macrophages expand, although bulk tissue data cannot distinguish changes in cell state from changes in cell number.

Astrocyte-associated genes were also dysregulated in the male midbrain. Prenatal and adult irradiation increase GFAP expression in rodents, and GFAP upregulation frequently coincides with NF-κB signalling during neuroinflammation [65,66,67,68,69,70,71]. Consistent with this, six genes within the *NF-κB* signalling pathway were upregulated in the male midbrain (FDR-adjusted *p* = 7.30 × 10^−4^). Because pathway enrichment reflects coordinated changes in transcript abundance rather than measured signalling activity, whether *NF-κB* signalling is functionally activated, and whether it represents a pro-survival response to radiation-induced apoptosis [72], remains to be determined.

Collectively, the dysregulation of microglial, astrocytic, and immune-related genes constitutes a pronounced inflammatory transcriptional signature following high-dose radiation exposure on GD 14.5. Because microglial and astrocytic activation were inferred from transcriptomic enrichment rather than directly measured by immunohistochemistry, future validation using cell-specific markers, such as P2Y12 and TMEM119 for homeostatic microglia and CD169 for border-associated macrophages, will be important. Beyond immune activation, gestational radiation exposure also altered transcriptional pathways governing glial development.

#### 4.2.2. Glial Development and Gliogenesis-Associated Dysregulation

Transcriptomic analysis also revealed alterations in genes associated with glial development. Significant GO terms related to gliogenesis, glial cell differentiation, astrocyte differentiation, and oligodendrocyte differentiation were dysregulated within the hindbrain. Consistent with this, cell-type enrichment indicated altered expression of oligodendrocyte-associated genes in the hindbrain of both sexes and astrocyte-associated genes in the male midbrain. GO analysis of cellular components further supported oligodendrocyte dysregulation, with compact myelin, myelin sheath, and main axon affected. At the gene level, *Mobp* was the most strongly downregulated gene in the hindbrain of both sexes (male fold change = −34.20; female fold change = −20.56) and was accompanied by reduced *Mbp* and *Plp1* in both sexes and *Mag* in females. *Mobp* encodes an abundant structural protein of compact CNS myelin expressed by maturing oligodendrocytes [73]. Because myelination in the mouse is only beginning at GD 18.5, as reflected by low *Mobp* expression in the sham hindbrain, this coordinated reduction more likely indicates delayed onset of oligodendrocyte maturation than loss of established myelin. Fetal growth restriction, which occurs in this model [74], is itself associated with impaired oligodendroglial maturation [75]. This effect may therefore reflect direct radiation effects on the oligodendrocyte lineage, indirect effects of growth restriction, or both.

Previous literature examining radiation effects on glial populations is mixed. Rola et al. (2004) reported increased oligodendrocyte progenitor and astrocyte proliferation in the hippocampus of young mice following 5 Gy of X-rays, suggesting a compensatory response to neuroinflammation, whereas Panagiotakos et al. (2007) found reduced oligodendrocytes in the SVZ of rats exposed to 25 Gy, likely reflecting impaired progenitor renewal at higher doses [15,76,77]. Together, these findings suggest that radiation dose and brain region influence glial response, with lower exposures eliciting compensatory proliferation and higher exposures reducing stem cell populations. The present findings are consistent with gestational irradiation disrupting glial developmental programs, although the direction of cellular change cannot be determined from transcriptomic enrichment alone.

Cresyl violet (Nissl) staining was attempted to distinguish and quantify neuronal and glial cell populations, as previously reported in mice and monkeys, but individual glial subtypes could not be confidently identified due to technical limitations [78,79]. Future work employing immunohistochemical markers, such as *GFAP* for astrocytes, *SOX10* for oligodendrocytes, and *IBA1* for microglia, would enable more precise identification of glial cell types [65,80]. MBP and MOBP immunostaining at postnatal time points could determine whether the reduced myelin gene expression represents a transient delay or persistent hypomyelination. Differentiating mature glial cells from their stem cell counterparts is also important for understanding how gestational radiation exposure alters glial development and subsequent neuronal connectivity.

Collectively, the transcriptomic findings indicate that gestational radiation exposure may disrupt glial development in a region-dependent manner, with dysregulation of gliogenesis and cell-type markers throughout the brain suggesting potential impairment in myelination, blood–brain barrier integrity, and neuroinflammation regulation. Beyond glial development, proper cell migration is also essential for brain organization and function [81,82].

#### 4.2.3. ECM and Migration-Associated Pathway Dysregulation

Pathway analysis identified potential effects on cell migration through dysregulation of the ECM–receptor interaction pathway, in which all dysregulated genes were upregulated (23 in males and 25 in females). The ECM plays a critical role in neural stem cell differentiation and migration, and its disruption has been linked to neurodevelopmental disease [83]. Radiation exposure during gestation has likewise been shown to impair cellular migration [20,22]. Although cell migration was not directly analysed, dysregulation of this pathway, together with previous literature, suggests that migration-associated molecular programs may be altered following irradiation and may contribute to the reduced hippocampal volume and enlarged lateral ventricle observed in exposed fetuses. Future investigations employing dual BrdU and EdU labelling could directly track cell migration following gestational radiation exposure [84].

#### 4.2.4. Serotonergic Pathway Dysregulation

Beyond developmental and migration-associated programs, transcriptional changes were also observed in the neurotransmitter systems. Transcriptomic analysis revealed dysregulation of serotonin-system genes in the male midbrain, with enrichment in serotonin biosynthetic, uptake, and metabolic processes. Pathway analysis also identified significant alterations to the serotonergic synapse pathway (Supplementary Figure S7), characterized by increased expression of genes associated with serotonin synthesis (*Tph* and *Ddc*) and vesicle packaging (*Vmat*). Upregulation of *Sert*, which encodes the serotonin reuptake transporter, suggests a potential compensatory response to maintain synaptic homeostasis. Broader neurotransmitter-related dysregulation was also identified within GO biological process terms, including aminergic neurotransmitter loading into synaptic vesicles, monoamine transport, and primary amino compound metabolic process.

Previous literature consistently demonstrates that radiation exposure disrupts multiple neurotransmitter systems. High-dose exposure in rodents reduces levels of GABA, serotonin, dopamine, and other monoamine and amino acid neurotransmitters across several brain regions, including the hypothalamus and hippocampus, often alongside altered glutamate/GABA ratios and receptor imbalances that indicate excitatory-inhibitory dysregulation within neural circuits [85,86].

Serotonin influences higher-order processes such as mood regulation, learning, appetite, and stress responses, so dysregulation of serotonergic signalling may have functional implications if these changes persist as the brain matures [87]. Previous studies have reported increased anxiety-like behaviour, reduced exploratory activity, and short-term memory deficits following radiation exposure of 1 Gy or higher [86]. Together, these findings raise the hypothesis that radiation-induced dysregulation of neurotransmitter systems, particularly the serotonergic pathway, may contribute to long-term alterations in neural signalling and behaviour, which will require postnatal assessment to test.

#### 4.2.5. Sex-Stratified Transcriptional Patterns Following Gestational Irradiation

In the sex-stratified analysis, the DEG profiles of males and females differed in both number and composition. More genes were uniquely dysregulated in each sex than shared. Males had more DEGs in the telencephalon, midbrain, and hindbrain, whereas females had nearly twice as many DEGs in the diencephalon. However, because each sex was analysed separately, these differences are descriptive and do not constitute a formal test of a treatment × sex interaction. Differences in DEG number between sex-specific comparisons can also arise from differences in within-group variability and statistical power, and a gene reaching significance in only one sex does not demonstrate the absence of an effect in the other.

Sex differences in neuroinflammatory responses to radiation have been reported previously. Female mice exhibit greater cytokine and chemokine upregulation in the hippocampus and sustained microglial activity weeks after high-dose radiation exposure, as well as greater loss of granule cell layer volume and immature neuron populations following cranial radiation exposure [88,89]. In contrast, the sex-stratified analysis in this study identified more DEGs in males in three of the four regions, despite no significant morphometric or histological sex differences. This discrepancy may reflect differences in strain, developmental timing, tissue collection time point, the brain region analysed, radiation dose, or the statistical considerations noted above.

Collectively, these results suggest that sex may influence molecular responses to gestational radiation exposure, though the direction and magnitude may vary with strain, dose, and developmental timing. Confirming this will require studies designed and powered to formally test treatment × sex interactions, ideally spanning multiple doses and developmental time points.

#### 4.2.6. Conserved Versus Developmental-Stage-Specific Radiation Responses

A meta-analysis of human transcriptomic datasets exposed to high-dose ionizing radiation identified a conserved response characterized by suppression of cell-cycle genes (e.g., *Mki67*, *Cdk1*, *Plk1*), induction of p53 targets such as *Cdkn1a* and *Mdm2*, and altered DNA repair and apoptotic pathways [90]. Several p53-responsive genes were also dysregulated in the present study, including *Eda2r*, which was differentially expressed in all four brain regions of both sexes, as well as *Psrc1*, *Pidd1*, *Cdkn1a*, *Phlda3*, and *Tnfrsf10b.* This p53-associated signature is consistent with earlier work in the developing brain, in which irradiation at GD 13 induced canonical p53 targets including *Cdkn1a*, *Mdm2*, and *Ccng1* within hours of exposure [91]. *Eda2r* has since been characterized as a sensitive, dose-responsive transcriptional marker of radiation exposure in human blood [92]. *Eda2r* encodes the X-linked ectodysplasin A2 receptor (XEDAR), a TNF receptor superfamily member that is a direct p53 target and signals through NF-κB [93], consistent with its presence among the upregulated genes of the NF-κB signalling pathway in the male midbrain (Supplementary Figure S6). Its uniform upregulation across all fetal brain regions four days after exposure suggests that Eda2r could serve as a tissue-level marker of fetal brain radiation exposure. Future studies could test whether its expression scales with dose and persists postnatally.

A comparable core response has been reported across radiation qualities, including high-LET exposures [94], supporting the interpretation that this program is broadly conserved. Notably, this p53-associated signature remained detectable four days after exposure, indicating that induction of these transcripts persists well beyond the acute DNA damage response. Alongside this conserved component, the dominant signature at GD 18.5 was immune, extracellular matrix, glial, and neurotransmitter-related rather than cell-cycle-related, likely reflecting both the later sampling time, at which the initial proliferative arrest has largely resolved, and features specific to the developing brain, including resident microglia, ongoing gliogenesis and neuronal migration, and regionally distinct maturation. Regional differences in p53-dependent radiation responses have also been reported in the irradiated embryonic brain, where the extent of DNA damage signalling and apoptosis differs between brain regions [95], consistent with the region-specific transcriptional differences observed here. Together, these comparisons indicate that a conserved p53-driven response is retained in the irradiated fetal brain and is accompanied by a brain-, developmental-stage-, and region-specific transcriptional response to gestational radiation.

### 4.3. Study Scope and Future Directions

This study established a focused, biologically relevant model to examine the transcriptional and structural consequences of high-dose gestational radiation exposure on the fetal mouse brain. By concentrating on a single, well-characterized dose and a defined developmental window, it provides clear insight into region-specific vulnerability during late gestation and forms a foundation for future studies examining dose–response relationships, developmental timing, and long-term outcomes.

The single high-dose exposure (1.8 Gy) was selected based on prior evidence of reproducible fetal growth restriction without complete lethality, enabling robust detection of radiation-induced molecular and structural alterations [74]. Future studies may incorporate multiple doses to evaluate dose–response relationships and determine thresholds of neurodevelopmental sensitivity across accidental, occupational, and medically relevant exposure ranges.

Because the dam received whole-body irradiation, the fetal brain response reflects the combined effects of direct fetal exposure and indirect maternal, placental, and systemic responses. Irradiation of this model induces fetal growth restriction and immune-related transcriptional changes in the placenta [23,74], and maternal inflammatory signalling, altered placental function, or growth restriction itself may contribute to the fetal brain transcriptome. The present design cannot separate direct radiation effects on neural tissue from these indirect effects. Approaches such as shielded and fetus-targeted exposure, or comparison with non-radiation models of growth restriction, would be required to distinguish them.

Irradiation at gestational day 14.5 with analysis at gestational day 18.5 allowed assessment of radiation effects during a critical period of neurogenesis and neuronal migration, while avoiding the confounding stress of parturition. Although this design captures acute developmental responses, future work could add gestational and postnatal time points to determine whether the observed transcriptional and structural changes persist, evolve, or resolve, and whether delayed effects emerge in regions such as the cerebellum.

Finally, this study focused on transcriptional and structural outcomes and did not assess epigenetic regulation. In other developmental models, radiation exposure influences DNA methylation, histone modification, and non-coding RNA expression [96,97,98,99,100]. Whether such mechanisms contribute to the gene expression changes observed here remains an open question requiring future study.

## 5. Conclusions

This study demonstrates that high-dose gestational radiation exposure produces region-specific structural and molecular disruption in the fetal mouse brain. The hippocampus exhibited reduced volume, the lateral ventricle was enlarged, and no significant change was detected in the cerebellum. These structural alterations were accompanied by widespread immune-related gene dysregulation, changes in glial and extracellular matrix-associated pathways, altered serotonergic signalling, and differing gene expression profiles between males and females in sex-stratified analyses, although a treatment × sex interaction was not formally tested.

Collectively, these findings underscore the vulnerability of the developing brain to high-dose radiation exposure. Whether these acute fetal changes persist and influence postnatal neurological function remains to be tested. To our knowledge, this study provides one of the first integrated characterizations of transcriptome-wide dysregulation across multiple brain regions in both sexes, accompanied by structural changes, following high-dose gestational radiation exposure in the fetal mouse brain, establishing a foundation for future research aimed at understanding and mitigating radiation-induced neurodevelopmental injury.

## Figures and Tables

**Table 1:** Primers utilized for RT-qPCR.

Gene (Accession #)	Primer Direction	Sequence (5′–3′)	Product Size (bp)
*Rer1* (NM_001362981)	Forward	TGTCCTCTGTGAAATCAAGGTTAC	71
	Reverse	GGCTTCCCATGGACAGAGTC	
*Actb* (NM_007393.5)	Forward	TATAAAACCCGGGCGGCGCA	119
	Reverse	GTCATCCATGGCGAACTGGTG	
*Lyz2* (NM_017372.3)	Forward	TGAACGTTGTGAGTTTGCCA	90
	Reverse	TGAGCTAAACACACCCAGTCG	
*Bst2* (NM_198095)	Forward	CCTGTAGAGACGGGTTGCG	143
	Reverse	CTGAAGGGTCACCACGGTC	
*Icam1* (NM_010493.3)	Forward	CCACTGCCTTGGTAGAGGTG	127
	Reverse	ACCGGAGCTGAAAAGTTGTAGA	
*Psrc1* (NM_001190161.1)	Forward	TCCCTGGACCGACTAGGTAA	112
	Reverse	AGTTAGGGACCACTGGCAGA	
*Dnah7b* (NM_001368717.1)	Forward	CAAGTCCTTGCCGCCATGAG	111
	Reverse	TCTGCTCGATAGTTTATCCATAGGC	
*Ttr* (NM_013697.5)	Forward	CCATGAATTCGCGGATGTGG	140
	Reverse	TCCTGGGCTGAGTCTCTCAA	
*Tcf7l2* (NM_001412706)	Forward	TTCCTCCGATCACAGACCTGA	143
	Reverse	AAAGTCTGTCGGTCAAGCCC	
*Kcng4* (NM_001357210.1)	Forward	GTCAGTGAGACATGGGCTCC	49
	Reverse	GGCTCACCTGTCGCTTGTT	

**Table 2:** RNA-seq fold change validation via RT-qPCR. Differential gene expression of telencephalon, diencephalon, and hindbrain was validated by RT-qPCR where fold changes and adjusted *p*-values for both RNA-seq and RT-qPCR are compared (relative to sham-irradiated controls) (n = 8 per sex per treatment group, one fetus per sex per dam; sham: 8 dams, irradiated: 9 dams). Changes in gene expression were considered significant with a fold change ≥ +1.5 or ≤−1.5 and adjusted *p*-value < 0.05.

Telencephalon					
Gene	Sex	Fold Change (RNA-Seq)	*p*-Adj	Fold Change (RT-qPCR)	*p*-Adj
*Lyz2*	Female	+7.57	<0.0001	+6.75	<0.0001
	Male	+5.88	<0.0001	+5.70	<0.0001
*Bst2*	Female	+2.90	<0.0001	+3.63	<0.0001
	Male	+2.50	<0.0001	+2.85	<0.0001
*Icam1*	Female	+2.94	<0.0001	+2.39	<0.0001
	Male	+1.90	<0.0001	+2.29	<0.0001
*Psrc1*	Female	+1.44	<0.0001	+1.39	0.0174
	Male	+1.84	<0.0001	+2.41	<0.0001
*Dnah7b*	Female	+1.64	4.68 × 10^−3^	+1.77	4.40 × 10^−3^
	Male	+1.78	0.0100	+2.23	<0.0001
*Ttr*	Female	+1.66	<0.0001	+1.96	<0.0001
	Male	+1.69	<0.0001	+2.29	<0.0001
*Kcng4*	Female	−1.28	NA	−1.19	0.893
	Male	−1.29	NA	−1.01	0.852
**Diencephalon**					
*Kcng4*	Female	−2.06	0.0301	−3.82	<0.0001
	Male	−3.51	4.96 × 10^−4^	−2.38	<0.0001
**Hindbrain**					
*Tcf7l2*	Female	+1.08	0.998	−1.04	0.977
	Male	−2.24	1.39 × 10^−3^	−1.67	0.0440

Note: NA indicates that no adjusted *p*-value was generated because the gene was excluded during DESeq2 independent filtering or Cook’s distance outlier detection.

## Data Availability

All data supporting the findings of this study are included in the article and its Supplementary Materials. Complete lists of differentially expressed genes for each brain region and sex are provided in Supplementary Tables S2–S10. Raw sequencing data are available from the corresponding author upon reasonable request.

## Supplementary Materials

The following supporting information can be downloaded at https://www.mdpi.com/article/10.3390/genes17101267/s1, Table S1: Reagent preparation and staining conditions for hematoxylin and eosin staining; Figure S1: Gene ontology analysis of cellular components following gestational radiation exposure; Figure S2: Cell-type analysis using the molecular signatures database following gestational radiation exposure; Figures S3–S8: KEGG pathway diagrams; Figures S9 and S10: RT-qPCR validation of RNA-seq findings; Tables S2–S10: Complete differentially expressed gene lists by brain region and sex; Table S11: Transcriptomic sequencing and alignment metrics for all brain-region samples.

## Abbreviations

The following abbreviations are used in this manuscript:

ANOVA	Analysis of variance
cDNA	Complementary DNA
CPM	Counts per million
CSF	Cerebrospinal fluid
DEG	Differentially expressed gene
ECM	Extracellular matrix
FDR	False discovery rate
GD	Gestational day
GFAP	Glial fibrillary acidic protein
GO	Gene ontology
H&E	Hematoxylin and eosin
KEGG	Kyoto Encyclopedia of Genes and Genomes
NF-κB	Nuclear factor kappa B
RNA-seq	RNA sequencing
RT-qPCR	Reverse transcription quantitative polymerase chain reaction
SEM	Standard error of the mean
SGZ	Subgranular zone
SVZ	Subventricular zone
TPM	Transcripts per million
