# Supplementary material for: High-Dose Gestational Irradiation Disrupts Fetal Mouse Brain Structure and Region-Specific Transcriptomic Profiles
Source: Genes (Basel). 2026 Oct 12;17(10):1267. doi: 10.3390/genes17101267 (PMC13704159; doi:10.3390/genes17101267)
Supplement: Supplementary file 1 [file genes-17-01267-s001.zip › Supplementary Figures.pdf]

Supplementary Figures

a) Male Telencephalon

Gene Ontology ID	Description	DEGs	Total Genes	p-adj
GO:0005615	extracellular space	178	860	$1.00x10^{-24}$
GO:0005576	extracellular region	227	1282	$6.77x10^{-24}$
GO:0071944	cell periphery	507	4081	$1.69x10^{-18}$
GO:0009986	cell surface	128	655	$3.61x10^{-16}$
GO:0009897	external side of plasma membrane	68	246	$6.86x10^{-16}$
GO:0030312	external encapsulating structure	90	394	$1.55x10^{-15}$
GO:0031012	extracellular matrix	90	394	$1.55x10^{-15}$
GO:0005886	plasma membrane	438	3709	$2.31x10^{-11}$
GO:0062023	collagen-containing extracellular matrix	68	301	$2.31x10^{-11}$
GO:0098552	side of membrane	91	493	$5.15x10^{-10}$

b) Female Telencephalon

Gene Ontology ID	Description	DEGs	Total Genes	p-adj
GO:0005576	extracellular region	277	1266	$1.00x10^{-24}$
GO:0005615	extracellular space	213	848	$1.00x10^{-24}$
GO:0071944	cell periphery	570	4078	$1.00x10^{-24}$
GO:0005886	plasma membrane	483	3711	$1.00x10^{-24}$
GO:0030312	external encapsulating structure	110	387	$1.00x10^{-24}$
GO:0031012	extracellular matrix	110	387	$1.00x10^{-24}$
GO:0009986	cell surface	142	661	$1.00x10^{-24}$
GO:0009897	external side of plasma membrane	76	251	$4.32x10^{-23}$
GO:0062023	collagen-containing extracellular matrix	77	296	$6.16x10^{-19}$
GO:0098552	side of membrane	101	498	$1.53x10^{-16}$

c) Female Diencephalon

Gene Ontology ID	Description	DEGs	Total Genes	p-adj
GO:0071944	cell periphery	32	4145	$2.78x10^{-5}$
GO:0005886	plasma membrane	28	3768	$5.06x10^{-4}$
GO:0005576	extracellular region	14	1285	$1.70x10^{-3}$
GO:0020003	symbiont-containing vacuole	2	4	$1.70x10^{-3}$
GO:0020005	symbiont-containing vacuole membrane	2	4	$1.70x10^{-3}$
GO:0030430	host cell cytoplasm	2	4	$1.70x10^{-3}$
GO:0033643	host cell part	2	4	$1.70x10^{-3}$
GO:0033646	host intracellular part	2	4	$1.70x10^{-3}$
GO:0033655	host cell cytoplasm part	2	4	$1.70x10^{-3}$
GO:0043656	host intracellular region	2	4	$1.70x10^{-3}$

d) Male Midbrain

Gene Ontology ID	Description	DEGs	Total Genes	p-adj
GO:0005858	axonemal dynein complex	3	25	0.0524
GO:0098857	membrane microdomain	7	297	0.0524
GO:0005576	extracellular region	15	1291	0.0524
GO:0009986	cell surface	10	673	0.0524
GO:0005930	axoneme	5	169	0.0524
GO:0097014	ciliary plasm	5	170	0.0524
GO:0036156	inner dynein arm	2	12	0.0524
GO:0030286	dynein complex	3	53	0.0647
GO:0005615	extracellular space	11	862	0.0647
GO:0045121	membrane raft	6	296	0.0683

e) Male Hindbrain

Gene Ontology ID	Description	DEGs	Total Genes	p-adj
GO:0005576	extracellular region	17	1312	$1.55x10^{-4}$
GO:0009986	cell surface	12	680	$2.11x10^{-4}$
GO:0071944	cell periphery	30	4163	$2.17x10^{-4}$
GO:1990660	calprotectin complex	2	2	$5.28x10^{-4}$
GO:0005615	extracellular space	12	875	$1.09x10^{-3}$
GO:0001533	cornified envelope	3	22	$1.64x10^{-3}$
GO:0005886	plasma membrane	26	3797	$2.09x10^{-3}$
GO:0043235	receptor complex	6	316	0.0158
GO:0009897	external side of plasma membrane	5	258	0.0368
GO:0042571	immunoglobulin complex, circulating	1	1	0.0378

f) Female Hindbrain

Gene Ontology ID	Description	DEGs	Total Genes	p-adj
GO:0043218	compact myelin	3	20	$9.50x10^{-4}$
GO:0097453	mesaxon	2	3	$9.50x10^{-4}$
GO:1990015	ensheathing process	2	3	$9.50x10^{-4}$
GO:0043209	myelin sheath	5	198	$5.25x10^{-3}$
GO:0009986	cell surface	8	668	$6.90x10^{-3}$
GO:0043220	Schmidt-Lanterman incisure	2	16	0.0185
GO:0071944	cell periphery	20	4158	0.0203
GO:0005576	extracellular region	10	1300	0.0203
GO:0005615	extracellular space	8	868	0.0203
GO:0044304	main axon	3	86	0.0203

Supplementary Figure S1: Gene ontology analysis of cellular components following gestational radiation exposure*.*

The top 10 enriched GO terms for a) male telencephalon, b) female telencephalon, c) female diencephalon, d) male midbrain, e) male hindbrain, and f) female hindbrain are shown, ranked by FDR adjusted p-value. Only terms with an FDR-adjusted p-value < 0.05 are displayed (n = 8 per sex per treatment group, one fetus per sex per dam; sham: 8 dams, irradiated: 9 dams). The number of DEGs and the total number of genes within each GO term are indicated, where "Total Genes" represents all genes annotated to the ontology category, and "DEGs" represents the subset that was significantly dysregulated.

a) Male Telencephalon

Gene Set ID	Gene Set Name	DEGs	Total Genes	p-adj
12194	DESCARTES FETAL CEREBELLUM MICROGLIA	101	465	$1.45x10^{-22}$
11801	MANNO MIDBRAIN NEUROTYPES HMGL	103	532	$2.79x10^{-19}$
11917	GAO LARGE INTESTINE ADULT CJ IMMUNE CELLS	91	451	$2.97x10^{-18}$
12010	HU FETAL RETINA FIBROBLAST	76	357	$1.31x10^{-16}$
12051	CUI DEVELOPING HEART C8 MACROPHAGE	61	245	$1.31x10^{-16}$
12205	DESCARTES FETAL CEREBRUM MICROGLIA	74	343	$1.40x10^{-16}$
11772	FAN EMBRYONIC CTX BIG GROUPS MICROGLIA	73	338	$1.95x10^{-16}$
11860	AIZARANI LIVER C6 KUPFFER CELLS 2	51	197	$1.08x10^{-14}$
11955	HAY BONE MARROW STROMAL	106	676	$1.10x10^{-13}$
11827	ZHONG PFC MAJOR TYPES MICROGLIA	75	397	$1.10x10^{-13}$

b) Female Telencephalon

Gene Set ID	Gene Set Name	DEGs	Total Genes	p-adj
12194	DESCARTES FETAL CEREBELLUM MICROGLIA	109	465	$3.26x10^{-29}$
11910	GAO LARGE INTESTINE 24W C11 PANETH LIKE CELL	80	277	$1.03x10^{-27}$
11801	MANNO MIDBRAIN NEUROTYPES HMGL	108	532	$9.59x10^{-24}$
12205	DESCARTES FETAL CEREBRUM MICROGLIA	82	343	$1.35x10^{-22}$
12051	CUI DEVELOPING HEART C8 MACROPHAGE	65	245	$2.35x10^{-20}$
11827	ZHONG PFC MAJOR TYPES MICROGLIA	85	397	$3.37x10^{-20}$
11955	HAY BONE MARROW STROMAL	117	676	$4.95x10^{-20}$
11772	FAN EMBRYONIC CTX BIG GROUPS MICROGLIA	76	338	$2.11x10^{-19}$
12185	DESCARTES FETAL ADRENAL MYELOID CELLS	44	124	$3.82x10^{-19}$
11800	MANNO MIDBRAIN NEUROTYPES HPERIC	121	734	$4.35x10^{-19}$

c) Female Diencephalon

Gene Set ID	Gene Set Name	DEGs	Total Genes	p-adj
11923	MURARO PANCREAS DUCTAL CELL	15	1165	$3.11x10^{-5}$
11800	MANNO MIDBRAIN NEUROTYPES HPERIC	12	734	$3.55x10^{-5}$
12010	HU FETAL RETINA FIBROBLAST	8	357	$36.4x10^{-4}$
11799	MANNO MIDBRAIN NEUROTYPES HENDO	11	821	$4.50x10^{-4}$
11863	AIZARANI LIVER C9 LSECS 1	7	287	$5.62x10^{-4}$
11881	AIZARANI LIVER C32 MVECS 3	5	118	$7.53x10^{-4}$
12417	FAN OVARY CL9 PUTATIVE APOPTOTIC ENDOTHELIAL CELL	7	336	$9.85x10^{-4}$
11772	FAN EMBRYONIC CTX BIG GROUPS MICROGLIA	7	338	$9.85x10^{-4}$
11768	FAN EMBRYONIC CTX BIG GROUPS BRAIN ENDOTHELIAL	7	346	$9.85x10^{-4}$
12070	BUSSLINGER ESOPHAGEAL PROLIFERATING BASAL CELLS	4	68	$9.85x10^{-4}$

d) Male Midbrain

Gene Set ID	Gene Set Name	DEGs	Total Genes	p-adj
12335	DESCARTES FETAL STOMACH CILIATED EPITHELIAL CELLS	8	249	$2.96x10^{-3}$
11808	MANNO MIDBRAIN NEUROTYPES HPROGM	8	287	$4.15x10^{-3}$
11910	GAO LARGE INTESTINE 24W C11 PANETH LIKE CELL	7	277	0.0132
12278	DESCARTES FETAL LUNG CILIATED EPITHELIAL CELLS	8	380	0.0132
12300	DESCARTES FETAL PANCREAS ISLET ENDOCRINE CELLS	5	126	0.0132
12105	BUSSLINGER DUODENAL EC CELLS	3	29	0.0152
12277	DESCARTES FETAL LUNG NEUROENDOCRINE CELLS	4	76	0.0153
12208	DESCARTES FETAL CEREBRUM ASTROCYTES	5	153	0.0203
12161	DESCARTES MAIN FETAL CILIATED EPITHELIAL CELLS	8	479	0.0315
12049	CUI DEVELOPING HEART C6 EPICARDIAL CELL	5	179	0.0332

e) Male Hindbrain

Gene Set ID	Gene Set Name	DEGs	Total Genes	p-adj
12258	DESCARTES FETAL KIDNEY MYELOID CELLS	7	138	$8.81x10^{-5}$
11801	MANNO MIDBRAIN NEUROTYPES HMGL	11	532	$8.81x10^{-5}$
12301	DESCARTES FETAL PANCREAS MYELOID CELLS	7	162	$8.81x10^{-5}$
12205	DESCARTES FETAL CEREBRUM MICROGLIA	9	343	$9.93x10^{-5}$
11849	ZHONG PFC C4 PTGDS POS OPC	6	132	$2.63x10^{-4}$
12247	DESCARTES FETAL INTESTINE MYELOID CELLS	6	156	$5.14x10^{-4}$
12233	DESCARTES FETAL HEART MYELOID CELLS	5	91	$5.14x10^{-4}$
12051	CUI DEVELOPING HEART C8 MACROPHAGE	7	245	$5.14x10^{-4}$
12206	DESCARTES FETAL CEREBRUM OLIGODENDROCYTES	4	47	$5.48x10^{-4}$
12296	DESCARTES FETAL MUSCLE MYELOID CELLS	5	99	$5.48x10^{-4}$

f) Female Hindbrain

Gene Set ID	Gene Set Name	DEGs	Total Genes	p-adj
12206	DESCARTES FETAL CEREBRUM OLIGODENDROCYTES	4	47	$7.58x10^{-4}$
12231	DESCARTES FETAL HEART SCHWANN CELLS	4	49	$7.58x10^{-4}$
12197	DESCARTES FETAL CEREBELLUM OLIGODENDROCYTES	4	61	$8.22x10^{-4}$
12205	DESCARTES FETAL CEREBRUM MICROGLIA	7	343	$8.22x10^{-4}$
11849	ZHONG PFC C4 PTGDS POS OPC	5	132	$8.22x10^{-4}$
11801	MANNO MIDBRAIN NEUROTYPES HMGL	8	532	$1.39x10^{-3}$
12296	DESCARTES FETAL MUSCLE MYELOID CELLS	4	99	$3.53x10^{-3}$
11825	ZHONG PFC MAJOR TYPES EXCITATORY NEURON	2	8	$5.33x10^{-3}$
12185	DESCARTES_FETAL_ADRENAL _MYELOID_CELLS	4	124	0.006563
12258	DESCARTES_FETAL_KIDNEY_ MYELOID_CELLS	4	138	0.008891

Supplementary Figure S2: Cell-type analysis using the molecular signatures database following gestational radiation exposure*.*

The top 10 dysregulated cell-types for a) male telencephalon, b) female telencephalon, c) female diencephalon, d) male midbrain, e) male hindbrain, and f) female hindbrain are shown, ranked by FDR-adjusted p-value. Only cell-type signatures with an FDR-adjusted p-value < 0.05 are displayed (n = 8 per sex per treatment group, one fetus per sex per dam; sham: 8 dams, irradiated: 9 dams). The number of DEGs and the total number of genes within each cell-type signature are indicated, where "Total Genes" represents all genes annotated to the ontology category, and "DEGs" represents the subset that was significantly dysregulated.

a) Male Telencephalon

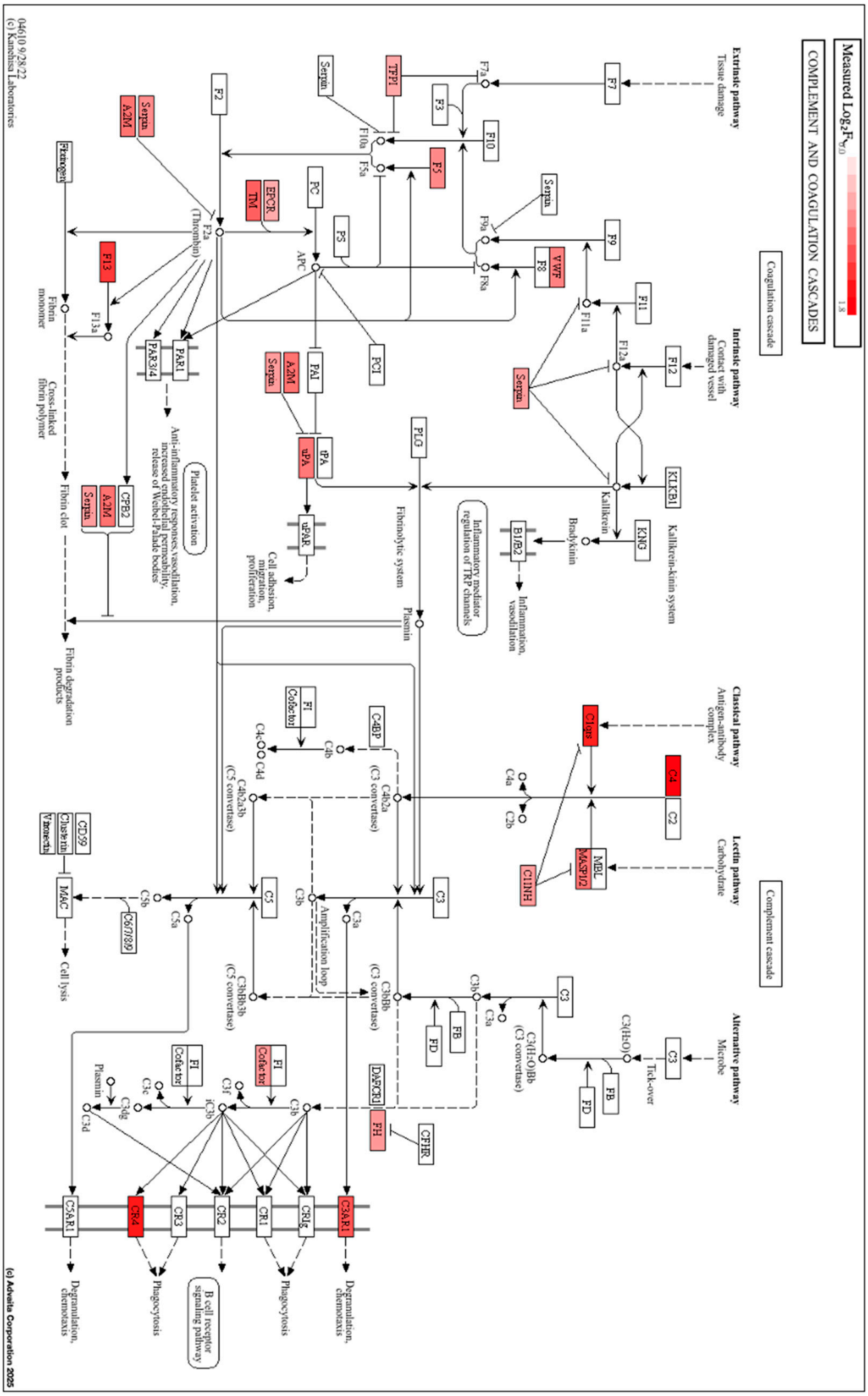

b) Female Telencephalon

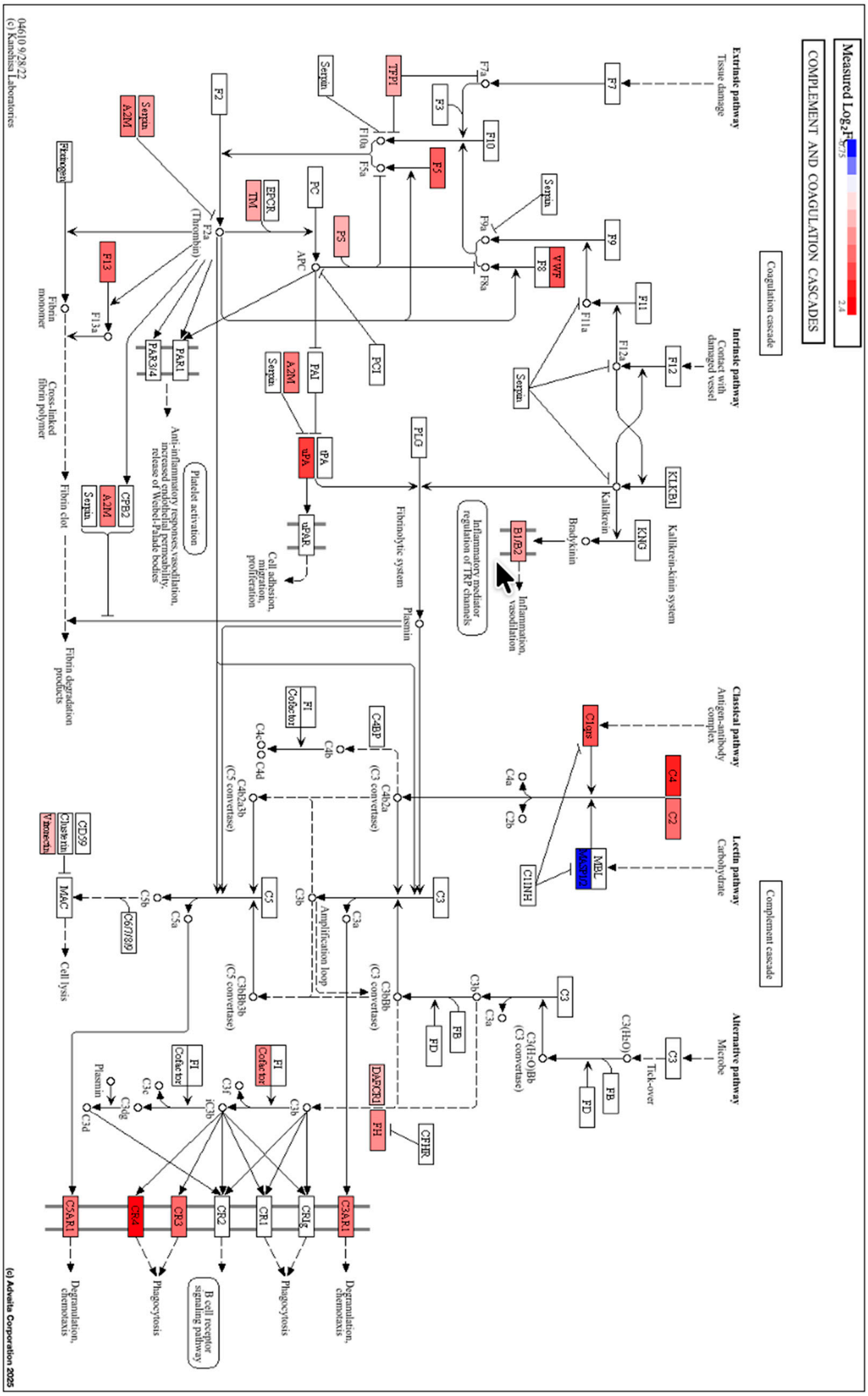

Supplementary Figure S3: Complement and Coagulation Cascade (KEGG: 04610).
The KEGG (Kyoto Encyclopedia of Genes and Genomes) pathway diagrams for (a) male and (b) female telencephalon are overlaid with gene perturbation values, accounting for both fold change and accumulated perturbation propagated from upstream genes (n = 8 per sex per treatment group, one fetus per sex per dam; sham: 8 dams, irradiated: 9 dams). Positive perturbation is indicated in red (darker red being the highest positive perturbation), while negative perturbation is indicated in blue (darker blue being the highest negative perturbation). The legend indicates the log_2 fold change by colour gradient with > 0.6 or < -0.6 being significant. The pathway visualized was developed in iPathwayGuide (Advaita Bioinformatics).

a) Male Telencephalon

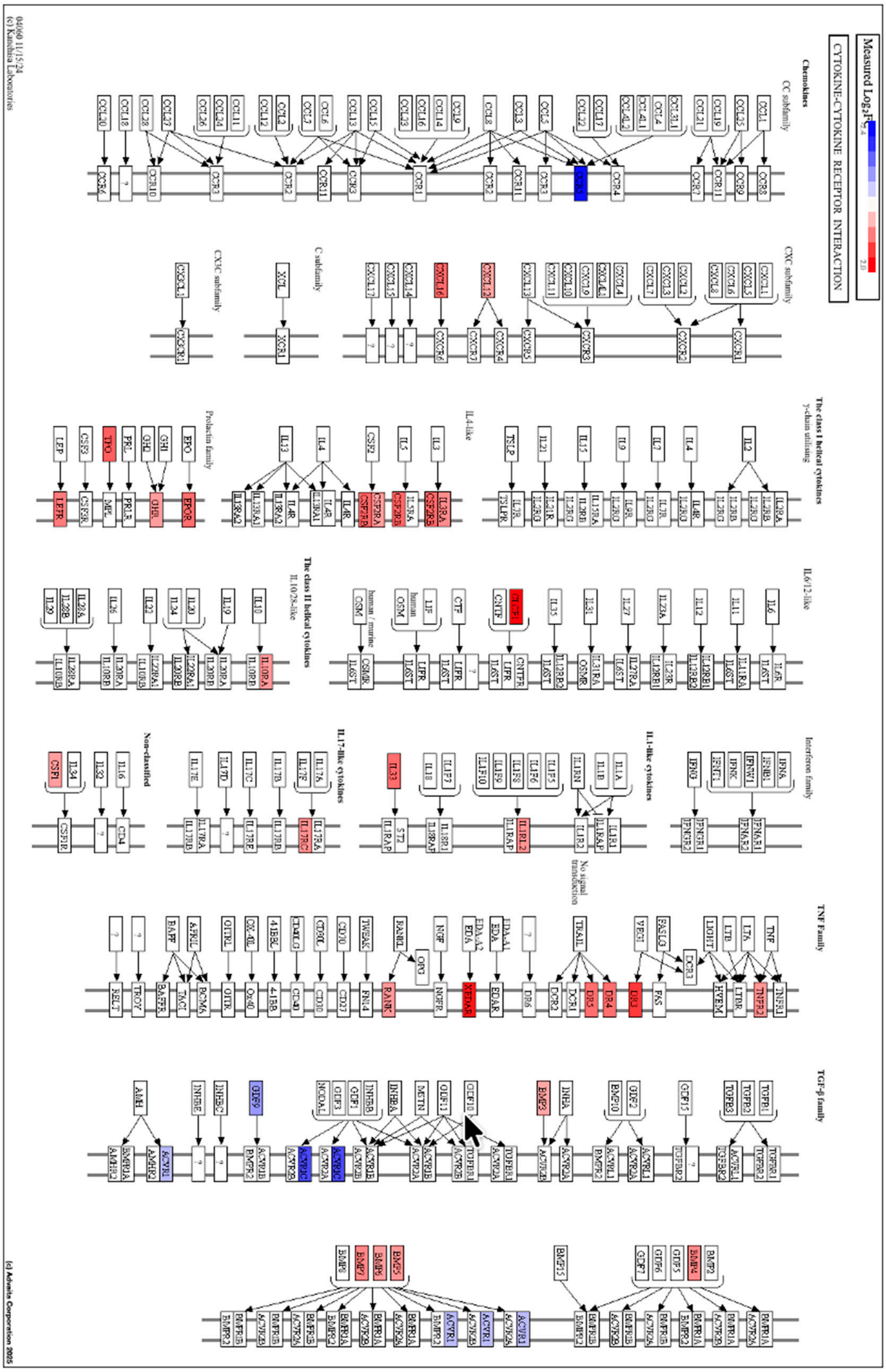

b) Female Telencephalon

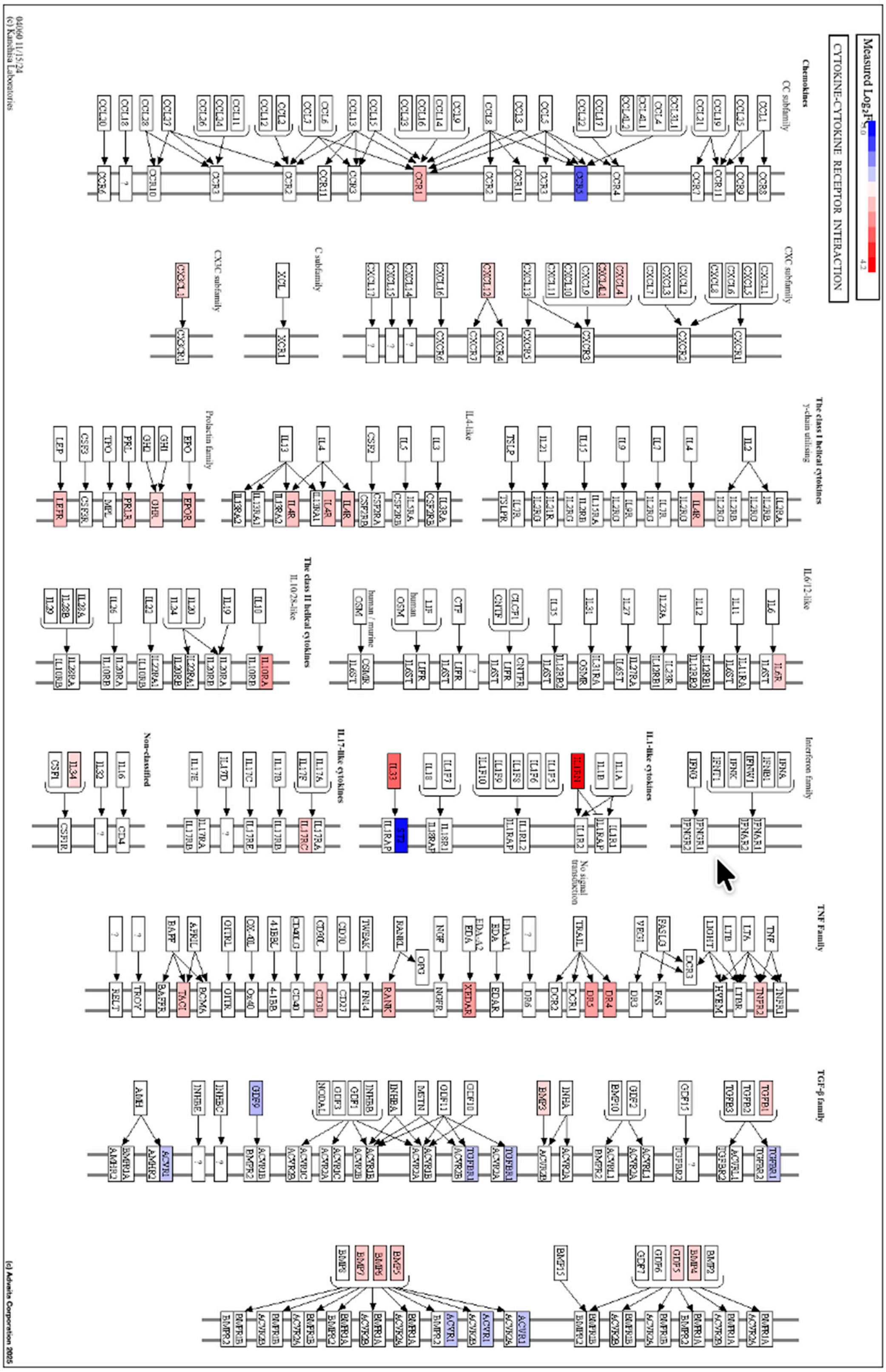

Supplementary Figure S4: Cytokine-Cytokine Receptor Interaction (KEGG: 04060)*.*
The KEGG (Kyoto Encyclopedia of Genes and Genomes) pathway diagrams for (a) male and (b) female telencephalon are overlaid with gene perturbation values, accounting for both fold change and accumulated perturbation propagated from upstream genes (n = 8 per sex per treatment group, one fetus per sex per dam; sham: 8 dams, irradiated: 9 dams). Positive perturbation is indicated in red (darker red being the highest positive perturbation), while negative perturbation is indicated in blue (darker blue being the highest negative perturbation). The legend indicates the log_2 fold change by colour gradient with > 0.6 or < -0.6 being significant. The pathway visualized was developed in iPathwayGuide (Advaita Bioinformatics).

a) Male Telencephalon

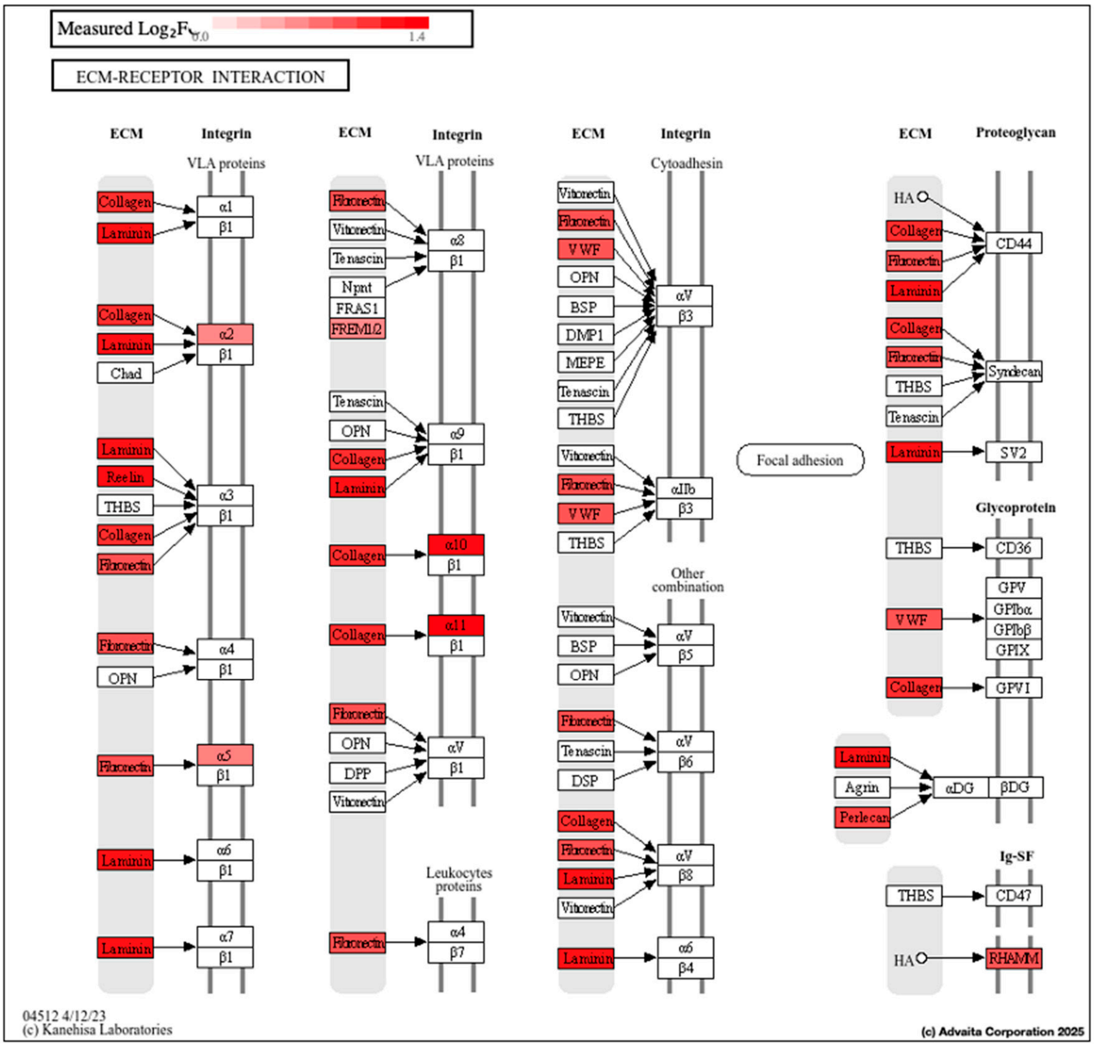

b) Female Telencephalon

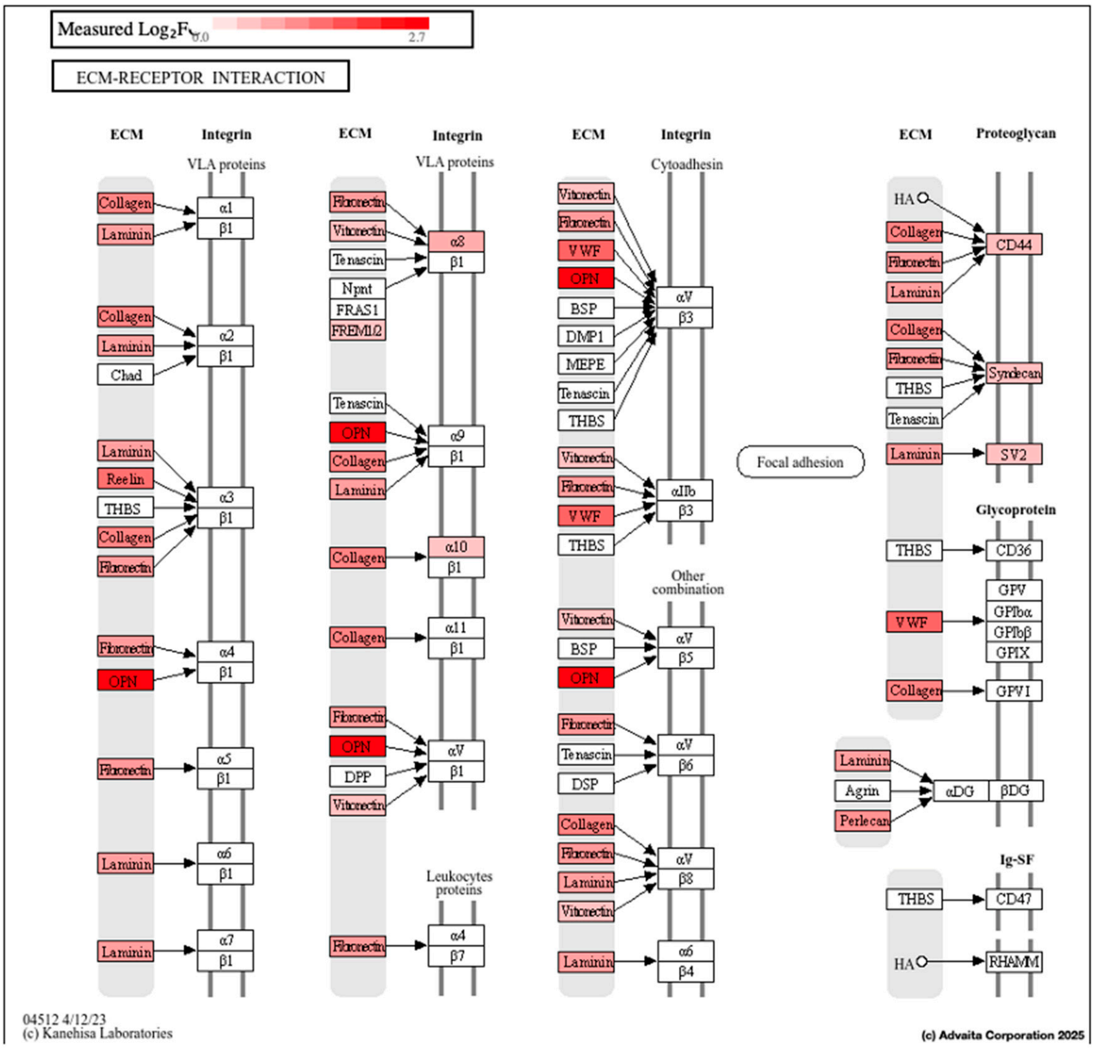

Supplementary Figure S5: ECM-Receptor Interaction (KEGG: 04512).
The KEGG (Kyoto Encyclopedia of Genes and Genomes) pathway diagrams for (a) male and (b) female telencephalon are overlaid with gene perturbation values, accounting for both fold change and accumulated perturbation propagated from upstream genes (n = 8 per sex per treatment group, one fetus per sex per dam; sham: 8 dams, irradiated: 9 dams). Positive perturbation is indicated in red (darker red being the highest positive perturbation), while negative perturbation is indicated in blue (darker blue being the highest negative perturbation). The legend indicates the log$_2$ fold change by colour gradient with > 0.6 or < -0.6 being significant. The pathway visualized was developed in iPathwayGuide (Advaita Bioinformatics).

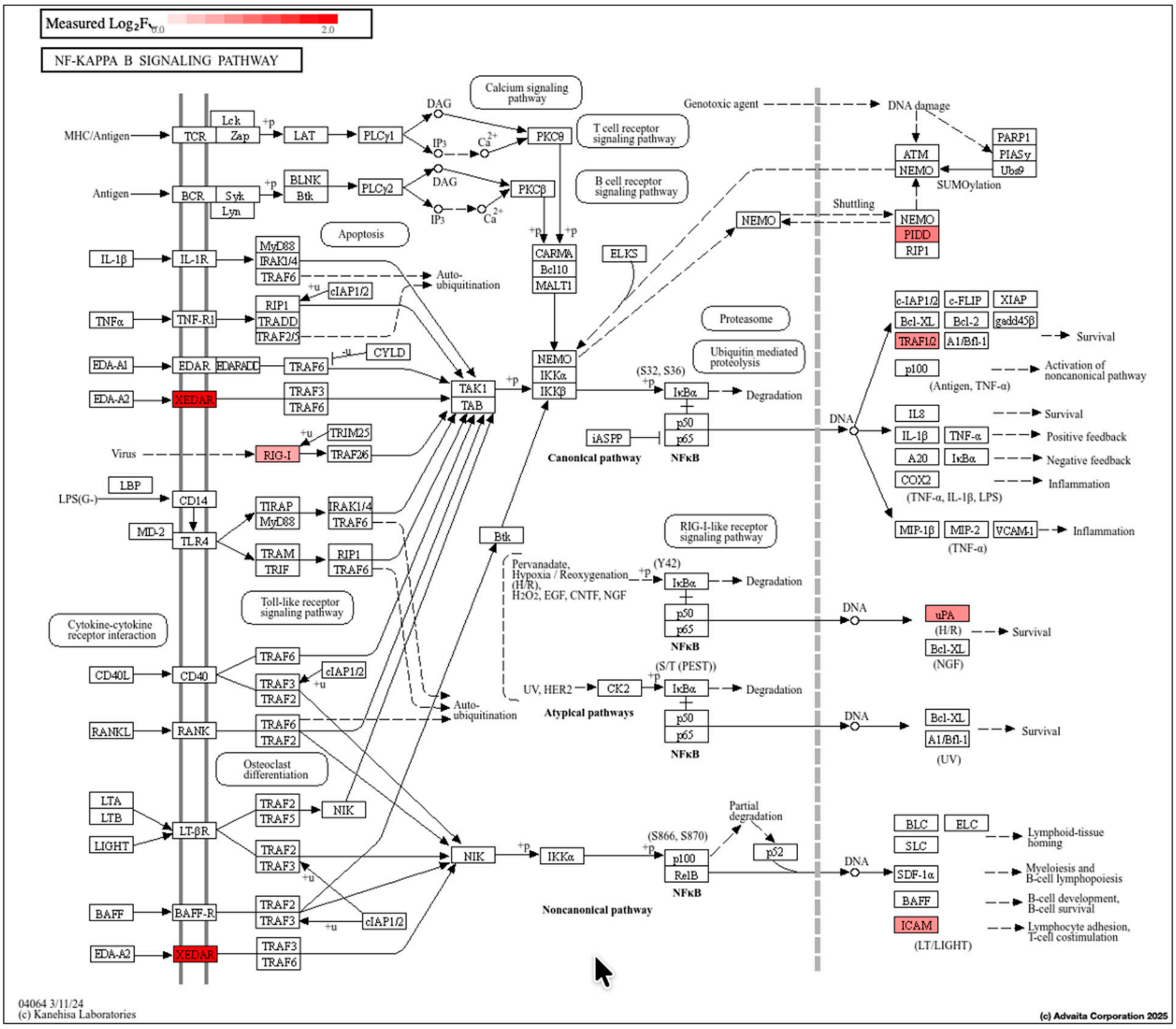

Supplementary Figure S6: NF-κB signalling pathway (KEGG: 04064).

The KEGG (Kyoto Encyclopedia of Genes and Genomes) pathway for the male midbrain is overlaid with gene perturbation values, accounting for both fold change and accumulated perturbation propagated from upstream genes (n = 8 per sex per treatment group, one fetus per sex per dam; sham: 8 dams, irradiated: 9 dams). Positive perturbation is indicated in red (darker red being the highest positive perturbation), while negative perturbation is indicated in blue (darker blue being the highest negative perturbation). The legend indicates the log_2 fold change by colour gradient with > 0.6 or < -0.6 being significant. The pathway visualized was developed in iPathwayGuide (Advaita Bioinformatics).

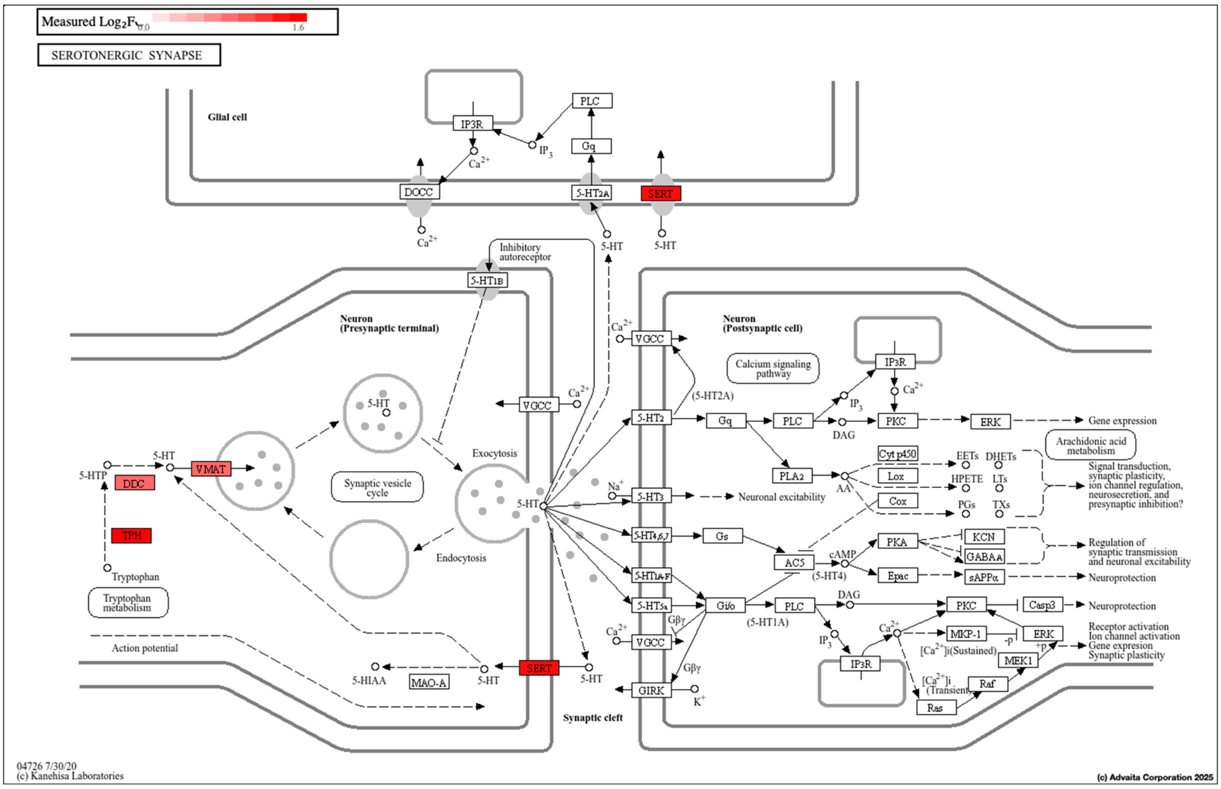

Supplementary Figure S7: Serotonergic Synapse (KEGG: 04726).

The KEGG (Kyoto Encyclopedia of Genes and Genomes) pathway for the male midbrain is overlaid with gene perturbation values, accounting for both fold change and accumulated perturbation propagated from upstream genes (n = 8 per sex per treatment group, one fetus per sex per dam; sham: 8 dams, irradiated: 9 dams). Positive perturbation is indicated in red (darker red being the highest positive perturbation), while negative perturbation is indicated in blue (darker blue being the highest negative perturbation). The legend indicates the log2 fold change by colour gradient with > 0.6 or < -0.6 being significant. The pathway visualized was developed in iPathwayGuide (Advaita Bioinformatics).

a) Male Hindbrain

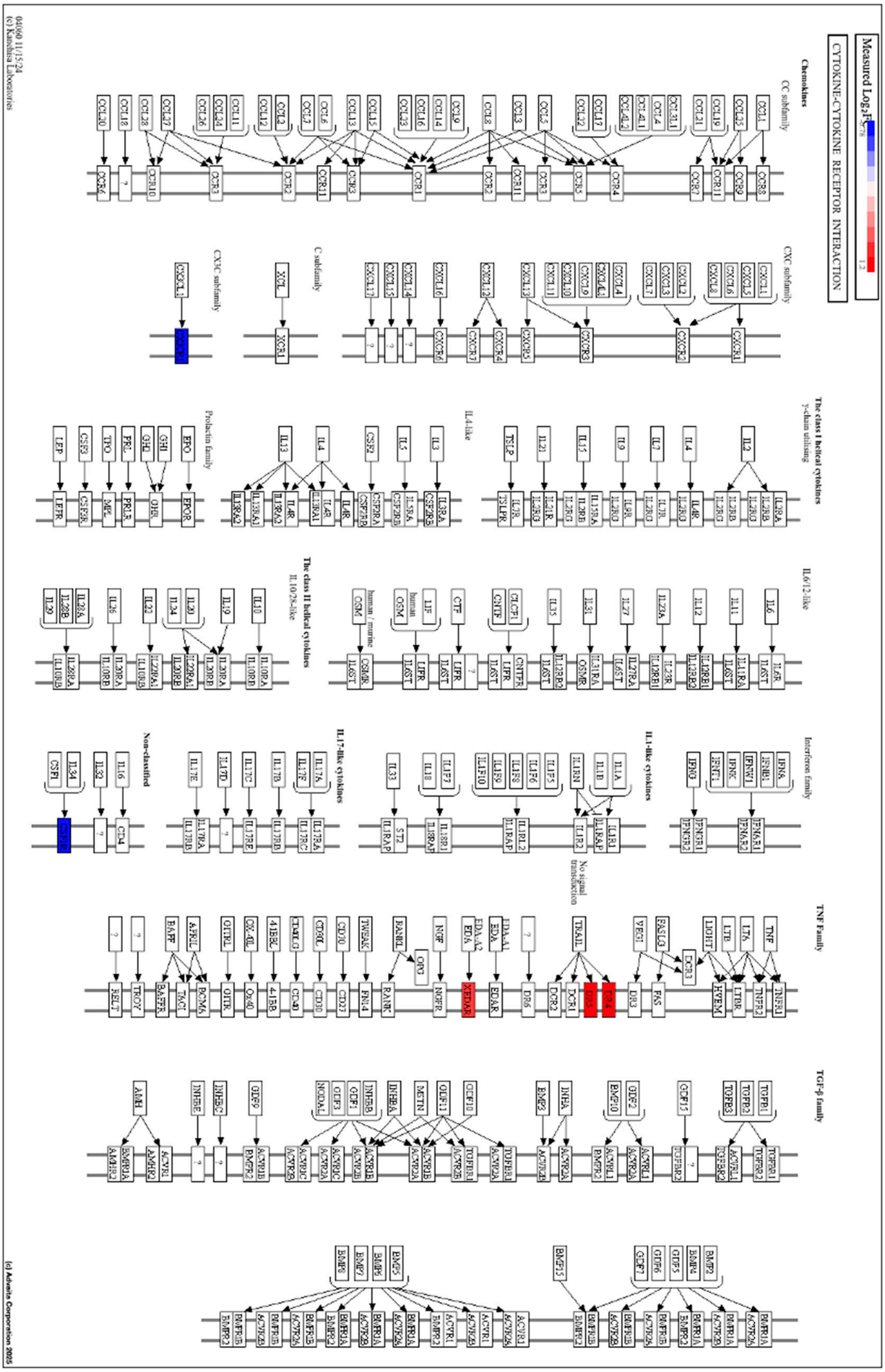

b) Female Hindbrain

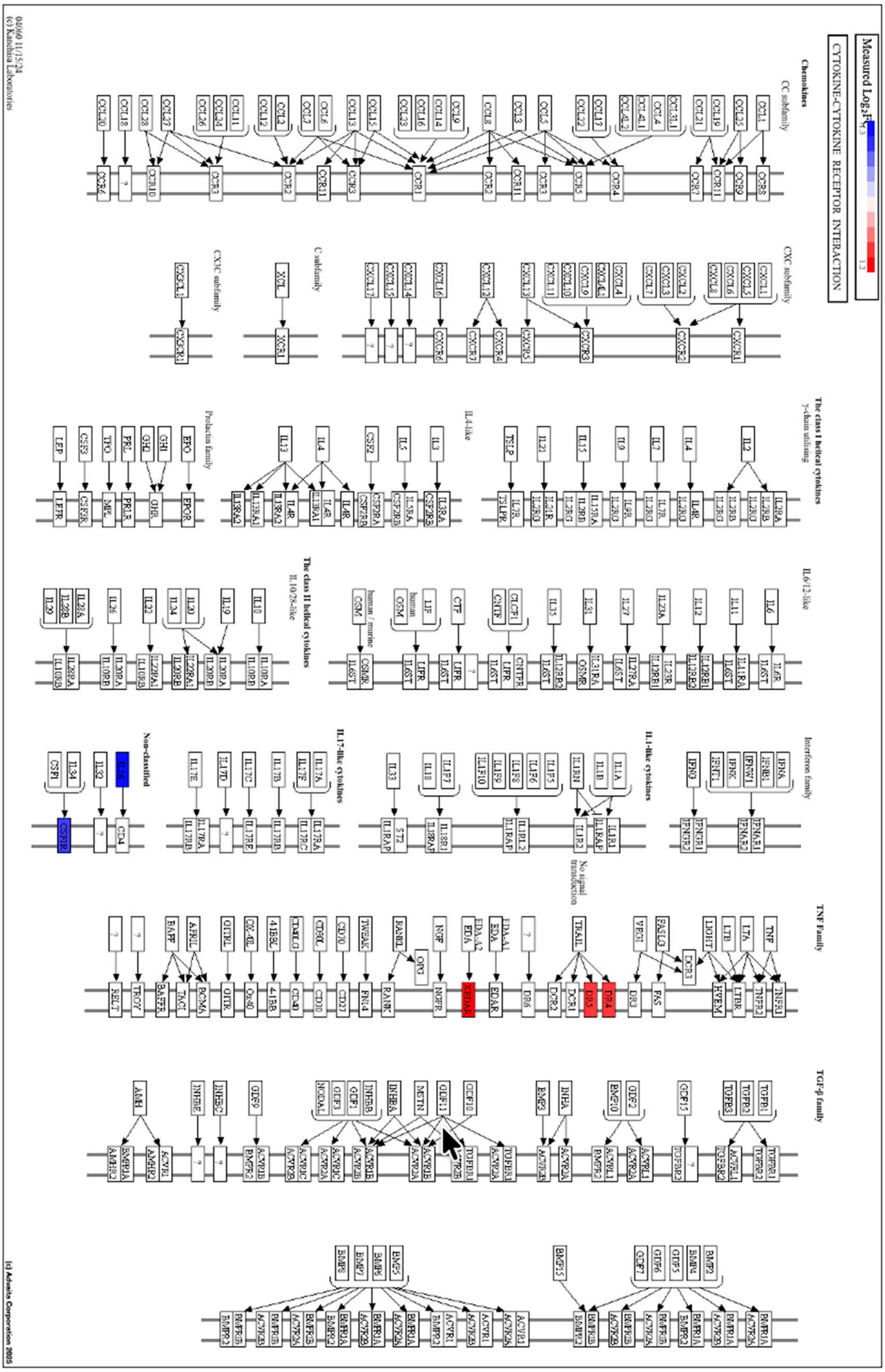

Supplementary Figure S8: Cytokine-Cytokine Receptor Interaction (KEGG: 04060).
The KEGG (Kyoto Encyclopedia of Genes and Genomes) pathway diagrams for (a) male and (b) female hindbrain are overlaid with gene perturbation values, accounting for both fold change and accumulated perturbation propagated from upstream genes (n = 8 per sex per treatment group, one fetus per sex per dam; sham: 8 dams, irradiated: 9 dams). Positive perturbation is indicated in red (darker red being the highest positive perturbation), while negative perturbation is indicated in blue (darker blue being the highest negative perturbation). The legend indicates the log_2 fold change by colour gradient with > 0.6 or < -0.6 being significant. The pathway visualized was developed in iPathwayGuide (Advaita Bioinformatics).

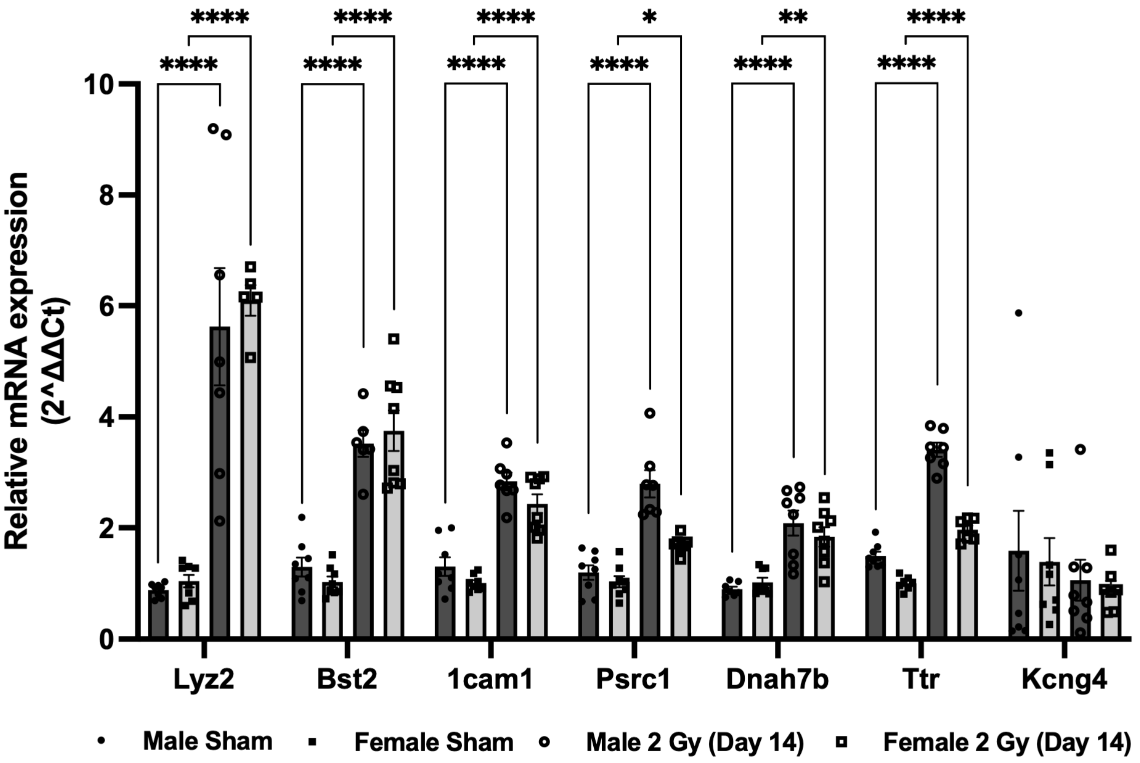

Supplementary Figure S9: RT-qPCR validation of RNA-seq findings in the telencephalon.
Relative mRNA expression (2^ΔΔCq) of Lyz2, Bst2, Icam1, Psrc1, Dnah7b, Ttr, and Kcng4 in male and female fetuses following sham or 1.8 Gy X-ray exposure on gestational day 14.5 (n = 8 per sex per treatment group, one fetus per sex per dam; sham: 8 dams, irradiated: 9 dams). Expression was normalized to housekeeping genes Rer1 and Actb and is presented relative to the sham group of the corresponding sex. Individual points represent biological replicates. Statistical analysis was performed using two-way ANOVA followed by Tukey's multiple comparison test. Data are presented as mean ± SEM.

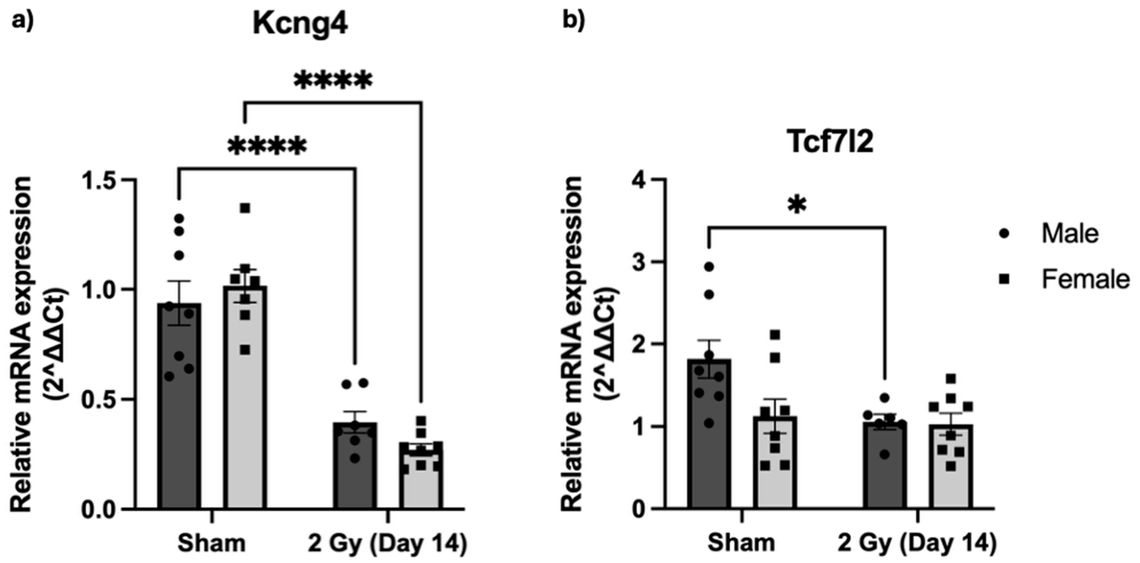

Supplementary Figure S10: RT-qPCR validation of RNA-seq findings in the diencephalon and hindbrain.

Relative mRNA expression (2^ΔΔCq) of (a) Kcng4 in the diencephalon and (b) Tcf7l2 in the hindbrain in male and female fetuses following sham or 1.8 Gy X-ray exposure on gestational day 14.5 (n = 8 per sex per treatment group, one fetus per sex per dam; sham: 8 dams, irradiated: 9 dams). Expression was normalized to housekeeping genes Rer1 and Actb and is presented relative to the sham group of the corresponding sex. Individual points represent biological replicates. Statistical analysis was performed using two-way ANOVA followed by Tukey's multiple comparison test. Data are presented as mean ± SEM.
