# Supplementary material for: High-Dose Gestational Irradiation Disrupts Fetal Mouse Brain Structure and Region-Specific Transcriptomic Profiles
Source: Genes (Basel). 2026 Oct 12;17(10):1267. doi: 10.3390/genes17101267 (PMC13704159; doi:10.3390/genes17101267)
Supplement: Supplementary file 1 [file genes-17-01267-s001.zip › Supplementary Tables.pdf]

Supplementary Tables

Supplementary Table S1: Hematoxylin and Eosin Staining Technique.

**Preparation of solutions and reagents**	
10% Alcoholic hematoxylin solution	- Dissolve 20 g of hematoxylin (Fisher Scientific, New Jersey) in 200 mL of 95% ethanol on heat until dissolved (stir)
Harris hematoxylin solution	- Dissolve 100 g of ammonium alum (Fisher Scientific, Ottawa, ON), in 1 L distilled water on heat to dissolve<br>- Add 50 mL of 10% alcoholic hematoxylin solution and heat to a boil (approx. 1 minute)<br>- Remove heat and add 2.5 g of mercuric oxide (red) (Reagents, Charlotte, NC, USA) and heat solution until purple in colour<br>- Cool solution in cold water bath and add 20 mL of glacial acetic acid (Fisher Scientific, New Jersey)<br>- Run through filter paper
Eosin Y stock solution	- Dissolve 1 g of eosin Y (Fisher Scientific, New Jersey) into 100 mL of distilled water
Phloxine B stock solution	- Dissolve 1 g of phloxine B (Cayman Chemical Company, Ann Arbor, MI) into 100 mL of distilled water
Eosin-phloxine B solution	- Add 100 mL of eosin Y stock solution, 10 mL of phloxine B stock solution, and 4 mL of glacial acetic acid to 780 mL of 95% ethanol
95% Ethanol	- Prepared from 100% ethanol (Greenfield Global, Brampton, ON, Canada) diluted with Milli-Q water

Methodology

- Dewax sections with two rounds of Xylene (Sigma-Aldrich, St. Louis, MO) for 2 minutes
- Rehydrate sections with two rounds of 100% ethanol (Greenfield Global, Brampton, ON) for 2 minutes, then 95% ethanol for 2 minutes, followed by 1X PBS for 2 minutes
- Stain sections with hematoxylin solution for 5 minutes, followed by 1 minute in 1X PBS wash
- Immerse section in 1% acid alcohol (differentiator), followed by 1 minute in 1X PBS wash
- Immerse section in 0.1% sodium bicarbonate (Fisher Scientific, Fair Lawn, NJ, USA) (blueing agent), followed by 1 minute in 1X PBS wash
- Wash section in 1X PBS for 1 minute prior to staining sample in Eosin-phloxine B solution for 3 minutes
- Dehydrate the sample starting with 95% ethanol for 1 minute, followed by two rounds of 100% ethanol for 2 minutes, then ending with two rounds of xylene for 2 minutes
- Attach coverslip with Permount mounting media (Fisher Scientific, New Jersey)

Supplementary Table S2: Differentially expressed genes in the male telencephalon following gestational radiation exposure.

All 1479 genes differentially expressed between control and gestationally irradiated male telencephalon (n = 8 per sex per treatment group, one fetus per sex per dam; sham: 8 dams, irradiated: 9 dams), identified by DESeq2 as log2 fold change ≥ 0.6 or ≤ - 0.6 with a Benjamini-Hochberg false discovery rate (FDR) adjusted p-value (p-adj) <0.05. Genes are ranked by descending log2 fold change, where positive values indicate increased expression relative to control (1281 upregulated, 198 downregulated). Control CPM and Rad CPM are mean normalized counts per million for each group.

Gene ID	Log2 Fold Change	p-adj	Control CPM	Rad CPM
Gpnmb	3.98	9.34×10^{-17}	11.35	179.35
Gm11639	3.26	5.85×10^{-3}	2.54	24.29
Neurog1	2.85	1.10×10^{-6}	2.75	19.88
Siglec1	2.85	7.85×10^{-14}	8.38	60.23
Ifit1	2.82	2.36×10^{-6}	2.53	17.88
Gm4117	2.69	3.96×10^{-2}	3.22	20.85
Lyz2	2.56	3.76×10^{-29}	83.54	491.15
Ms4a6d	2.13	1.12×10^{-15}	14.24	62.50
Oasl2	2.12	1.34×10^{-9}	13.46	58.53
Gm45110	2.12	2.58×10^{-5}	5.73	24.87
Gm47798	2.09	1.04×10^{-5}	5.35	22.72
Col24a1	2.07	1.99×10^{-4}	9.50	39.87
Nlrc5	2.06	2.88×10^{-3}	5.09	21.23
Ccdc88b	2.04	2.80×10^{-4}	7.68	31.49
Gm37606	2.03	7.50×10^{-5}	10.15	41.46
Ms4a7	2.02	1.91×10^{-7}	8.29	33.63
Oas1a	2.01	6.98×10^{-7}	5.84	23.55
Gm14827	2.01	9.33×10^{-10}	12.88	51.96
Clcf1	2.01	1.24×10^{-3}	4.08	16.46
Gm21982	2.01	1.74×10^{-3}	6.76	27.20
Clec4n	2.01	1.71×10^{-4}	4.77	19.17
Ifi44l	1.98	2.35×10^{-4}	4.12	16.27
Adam33	1.98	1.03×10^{-3}	4.21	16.62
Ripor3	1.98	2.33×10^{-5}	7.66	30.15
Atp13a4	1.96	2.09×10^{-4}	9.74	37.89
AC154542.1	1.96	4.80×10^{-3}	5.49	21.36
Ifi207	1.95	1.44×10^{-5}	6.17	23.90
A530041M06Rik	1.94	1.73×10^{-3}	4.44	17.04
Wdfy4	1.93	3.26×10^{-6}	13.85	52.94
Gm20605	1.92	1.08×10^{-13}	75.71	285.51
Tmc4	1.90	3.24×10^{-5}	8.30	31.10

Gene ID	Log2 Fold Change	p-adj	Control CPM	Rad CPM
Cybb	1.89	2.01×10^{-6}	8.43	31.22
Lilrb4a	1.89	5.20×10^{-3}	5.01	18.54
Gm42748	1.88	8.44×10^{-4}	6.97	25.70
Gm37309	1.86	3.06×10^{-4}	5.53	20.02
Xaf1	1.85	1.35×10^{-3}	5.87	21.19
Hmox1	1.85	4.65×10^{-11}	70.23	253.44
AC132288.1	1.84	2.93×10^{-4}	5.72	20.50
C4b	1.84	8.13×10^{-5}	11.45	41.05
Ifi44	1.84	1.97×10^{-8}	12.66	45.30
Gbp2	1.84	1.83×10^{-5}	6.53	23.33
Snhg11	1.84	4.25×10^{-21}	844.23	3,015.34
Ptgdr	1.83	9.78×10^{-6}	6.56	23.32
Rac2	1.82	9.97×10^{-6}	22.98	81.29
Gm42798	1.82	5.94×10^{-4}	4.70	16.56
Ifit3	1.79	5.72×10^{-6}	8.96	31.05
Gm42971	1.79	1.47×10^{-3}	7.91	27.38
Eda2r	1.77	2.72×10^{-11}	16.59	56.46
Gm43813	1.76	5.51×10^{-3}	7.74	26.19
Gm20559	1.75	8.24×10^{-6}	8.09	27.31
Gm37608	1.75	1.15×10^{-4}	21.95	73.97
Pla2g4b	1.75	1.21×10^{-4}	13.98	46.94
C130089K02Rik	1.73	7.05×10^{-3}	9.38	31.12
Ptpn18	1.72	4.55×10^{-3}	7.49	24.72
Aspg	1.71	1.07×10^{-3}	5.22	17.06
Parp14	1.70	1.91×10^{-10}	16.79	54.71
Ager	1.69	6.44×10^{-6}	7.47	24.19
Sned1	1.69	3.64×10^{-13}	164.82	531.86
Rbm47	1.69	7.65×10^{-7}	21.12	68.13
Ms4a6c	1.69	6.92×10^{-10}	16.29	52.48
Gm27000	1.68	6.58×10^{-4}	10.00	32.15
Gm35339	1.68	5.97×10^{-8}	14.01	44.78
Gm49493	1.67	1.33×10^{-4}	5.80	18.49
Scn10a	1.67	6.11×10^{-4}	5.25	16.73
Ptprc	1.67	1.90×10^{-6}	11.08	35.23
Gm28035	1.65	9.36×10^{-3}	42.12	132.34
B930086L07Rik	1.65	1.34×10^{-2}	5.28	16.56
Gm48700	1.65	1.08×10^{-2}	5.51	17.28
Gm33869	1.65	5.05×10^{-4}	12.82	40.15
Gm47087	1.65	2.49×10^{-2}	6.50	20.32

Gene ID	Log2 Fold Change	p-adj	Control CPM	Rad CPM
A230103L15Rik	1.64	7.21×10^{-6}	31.72	98.64
Gm43099	1.63	1.54×10^{-2}	7.94	24.61
Catsperg1	1.63	1.02×10^{-6}	18.90	58.51
Gm47761	1.63	1.92×10^{-7}	50.91	157.42
Coro6	1.62	5.48×10^{-3}	7.23	22.27
Dlx6os2	1.61	2.41×10^{-3}	7.98	24.43
8430422M14Rik	1.61	5.94×10^{-5}	7.79	23.74
Itgax	1.60	2.39×10^{-3}	8.75	26.56
Flt3l	1.60	9.61×10^{-3}	5.56	16.84
Themis2	1.59	2.42×10^{-4}	6.15	18.48
Lpo	1.58	3.36×10^{-3}	10.58	31.70
Gm12905	1.58	2.74×10^{-4}	10.97	32.74
Gm43654	1.58	1.68×10^{-2}	7.17	21.41
Gm42463	1.57	7.05×10^{-3}	8.35	24.77
Gm36963	1.56	4.51×10^{-3}	7.31	21.63
Abcg5	1.56	2.11×10^{-3}	6.44	19.04
Gm43188	1.56	2.13×10^{-2}	8.97	26.47
Slc2a4rg-ps	1.56	1.30×10^{-11}	45.88	135.32
Col20a1	1.55	1.05×10^{-5}	12.22	35.89
Pla2g4c	1.55	8.80×10^{-3}	6.90	20.19
Gm42047	1.55	1.24×10^{-6}	620.98	1,812.49
Agbl2	1.54	8.65×10^{-5}	7.24	21.12
C1ra	1.54	5.48×10^{-4}	6.95	20.28
Ubap1l	1.54	1.19×10^{-2}	5.34	15.53
Adam8	1.54	4.08×10^{-10}	36.30	105.38
Insrr	1.54	6.00×10^{-6}	13.11	38.03
Cd38	1.53	1.52×10^{-7}	11.66	33.67
AC151602.1	1.53	9.46×10^{-4}	7.54	21.73
Msr1	1.53	3.64×10^{-3}	9.47	27.27
Tnfrsf25	1.52	3.64×10^{-3}	5.49	15.77
Gm37018	1.52	3.30×10^{-2}	7.31	20.99
Dmpk	1.52	1.32×10^{-12}	132.81	380.64
Gm43481	1.51	2.57×10^{-4}	29.14	83.01
2900027M19Rik	1.50	2.26×10^{-2}	10.96	31.10
Gm49194	1.50	1.50×10^{-3}	6.97	19.78
Adamts19	1.50	9.78×10^{-8}	19.84	56.27
Gm37274	1.50	1.27×10^{-2}	6.11	17.24
Gm36936	1.50	1.68×10^{-5}	27.25	76.86
Plekhg4	1.49	3.15×10^{-9}	30.85	86.80

Gene ID	Log2 Fold Change	p-adj	Control CPM	Rad CPM
Ankle1	1.49	6.05×10^{-9}	23.99	67.26
Gm45074	1.48	1.97×10^{-2}	6.44	17.98
Gm45652	1.48	4.34×10^{-2}	6.20	17.28
Gm37893	1.47	3.34×10^{-3}	9.97	27.68
Gm37084	1.47	3.09×10^{-2}	8.07	22.31
Cfap43	1.46	4.79×10^{-4}	17.74	48.97
6430590A07Rik	1.46	2.22×10^{-3}	7.32	20.18
Gm43389	1.46	1.43×10^{-3}	6.72	18.49
Ifi203	1.46	8.14×10^{-8}	14.04	38.60
Igf2os	1.46	3.27×10^{-6}	19.49	53.45
Gm49759	1.45	7.20×10^{-12}	104.92	287.23
Gm43302	1.45	1.57×10^{-3}	12.63	34.47
Gm43759	1.45	1.08×10^{-3}	8.81	24.03
Gm44597	1.44	3.79×10^{-3}	42.08	114.45
Gm38057	1.44	8.09×10^{-3}	7.26	19.67
Pmfbp1	1.44	2.98×10^{-2}	5.81	15.73
AI480526	1.44	1.19×10^{-11}	188.13	508.88
Scube2	1.44	2.02×10^{-5}	12.13	32.79
Eldr	1.43	1.79×10^{-3}	13.97	37.69
Tlr13	1.43	1.49×10^{-4}	11.50	30.94
Gm45222	1.43	1.49×10^{-2}	7.75	20.81
Gm48796	1.42	2.00×10^{-2}	6.01	16.06
Gm29514	1.42	1.22×10^{-2}	6.58	17.57
9330162G02Rik	1.42	3.95×10^{-9}	184.69	492.81
Gm47939	1.42	2.18×10^{-5}	13.70	36.54
Hpse	1.42	2.26×10^{-3}	8.04	21.45
AI662270	1.41	6.29×10^{-3}	7.66	20.41
Gm37249	1.41	1.64×10^{-4}	16.80	44.77
Gm49502	1.41	8.79×10^{-3}	11.31	30.10
Tpcn2	1.41	9.62×10^{-5}	20.96	55.78
Rsad2	1.41	2.81×10^{-10}	33.77	89.83
Folr2	1.41	2.75×10^{-4}	8.02	21.30
Cd84	1.41	1.82×10^{-5}	14.88	39.44
P2rx5	1.41	1.65×10^{-4}	10.26	27.20
Gm44168	1.40	1.51×10^{-5}	26.84	71.07
Gm19817	1.40	1.40×10^{-2}	7.45	19.72
F13a1	1.40	8.38×10^{-19}	188.44	497.93
Prdm6	1.40	4.12×10^{-4}	19.87	52.42
Dmrta1	1.40	3.77×10^{-4}	10.46	27.57

Gene ID	Log2 Fold Change	p-adj	Control CPM	Rad CPM
Gm49027	1.40	9.93×10^{-10}	31.90	84.03
Tcf7l2	1.40	2.53×10^{-2}	239.20	629.21
Gm6548	1.39	3.21×10^{-5}	10.24	26.92
Gypa	1.39	6.60×10^{-3}	7.99	21.01
Slc39a2	1.39	1.29×10^{-5}	14.95	39.28
Cd68	1.39	5.64×10^{-9}	39.52	103.83
Pkn3	1.39	9.35×10^{-6}	17.29	45.39
Rnf213	1.39	9.23×10^{-16}	108.75	284.74
C130023A14Rik	1.39	3.14×10^{-3}	14.39	37.62
Gm43080	1.38	2.58×10^{-3}	7.10	18.49
Susd2	1.38	7.24×10^{-8}	20.49	53.26
Gm43336	1.38	3.74×10^{-2}	6.81	17.68
Gm42979	1.38	3.55×10^{-6}	25.35	65.77
Gm38043	1.38	4.19×10^{-2}	8.71	22.60
Stab1	1.37	6.10×10^{-12}	377.88	978.52
Col7a1	1.37	1.10×10^{-3}	66.65	172.30
A430110C17Rik	1.37	2.37×10^{-2}	6.77	17.47
Rtbdn	1.37	4.00×10^{-4}	13.66	35.24
C130051F05Rik	1.36	1.20×10^{-2}	6.01	15.46
Gm42991	1.36	1.72×10^{-3}	27.59	70.97
A230057D06Rik	1.36	7.08×10^{-8}	58.41	150.10
Itga11	1.36	3.79×10^{-15}	39.94	102.54
Ugt1a7c	1.36	6.70×10^{-5}	12.37	31.73
Ugt1a6b	1.36	3.52×10^{-4}	18.84	48.32
Ankrd23	1.36	6.95×10^{-5}	22.78	58.33
Miat	1.36	3.19×10^{-7}	2,679.46	6,856.60
Firre	1.36	1.49×10^{-11}	275.67	705.32
Per3	1.35	1.25×10^{-12}	51.84	132.60
C78859	1.35	4.99×10^{-9}	51.94	132.81
Gm26901	1.35	1.26×10^{-3}	8.38	21.42
A330023F24Rik	1.35	1.33×10^{-5}	42.61	108.74
Arhgap6	1.35	2.75×10^{-5}	11.51	29.36
Gm11611	1.35	1.10×10^{-2}	12.43	31.69
9930017N22Rik	1.35	5.18×10^{-3}	8.40	21.41
Osr1	1.35	1.10×10^{-12}	47.50	121.09
Prox2os	1.35	1.74×10^{-3}	9.20	23.43
Slfn2	1.35	1.09×10^{-4}	10.29	26.20
Prr32	1.35	5.79×10^{-6}	18.79	47.84
Ssh3	1.35	7.47×10^{-12}	134.15	340.92

Gene ID	Log2 Fold Change	p-adj	Control CPM	Rad CPM
Aebp1	1.34	6.62×10^{-26}	242.79	616.65
Gm44949	1.34	2.01×10^{-2}	6.13	15.51
Tgfbr3l	1.34	7.11×10^{-4}	25.59	64.75
Frrs1	1.34	1.52×10^{-5}	25.70	64.92
Gm20045	1.33	1.95×10^{-7}	32.52	82.04
Gm42992	1.33	3.19×10^{-2}	33.21	83.77
Nlrc3	1.33	1.75×10^{-5}	15.05	37.91
Lyve1	1.33	1.59×10^{-10}	70.73	177.80
Dnah11	1.33	4.12×10^{-3}	21.69	54.42
Bst2	1.32	4.08×10^{-10}	40.18	100.55
Slc25a37	1.32	6.22×10^{-9}	123.31	308.26
Rhbdf2	1.32	6.78×10^{-8}	17.44	43.50
Gm43483	1.32	4.14×10^{-2}	7.92	19.72
Neat1	1.31	7.67×10^{-9}	75.54	187.56
Fcgr2b	1.31	1.86×10^{-5}	24.21	60.09
Gm37310	1.31	9.65×10^{-3}	10.84	26.87
Usp18	1.30	2.80×10^{-3}	7.47	18.38
Igtp	1.30	4.58×10^{-5}	12.40	30.52
Gm43858	1.30	3.57×10^{-3}	11.24	27.63
Ptchd4	1.30	1.81×10^{-5}	20.59	50.54
Fmod	1.29	1.80×10^{-20}	263.79	647.19
Plekhd1	1.29	5.26×10^{-3}	11.31	27.71
Gm44286	1.29	3.37×10^{-2}	6.16	15.08
Gjb6	1.29	7.83×10^{-12}	92.34	225.42
Meg3	1.28	7.04×10^{-12}	8,805.13	21,434.60
Gm19938	1.28	1.10×10^{-3}	24.27	58.94
Gm19409	1.28	3.23×10^{-2}	7.17	17.40
Ttc39a	1.28	1.85×10^{-3}	7.93	19.22
C4a	1.28	2.73×10^{-2}	15.15	36.71
Irf7	1.28	3.85×10^{-5}	14.26	34.53
5330431K02Rik	1.27	6.34×10^{-4}	29.88	72.28
Gm44831	1.27	1.65×10^{-2}	6.52	15.78
Cfap44	1.27	2.07×10^{-5}	48.31	116.82
Cyp27a1	1.27	1.60×10^{-3}	7.22	17.46
Greb1	1.27	1.30×10^{-2}	24.27	58.59
Rgs11	1.27	1.53×10^{-5}	43.33	104.40
Mroh7	1.27	4.64×10^{-2}	13.61	32.76
D030055H07Rik	1.27	3.97×10^{-5}	24.44	58.83
H2-T24	1.27	2.15×10^{-5}	10.83	26.02

Gene ID	Log2 Fold Change	p-adj	Control CPM	Rad CPM
Gbp9	1.26	1.03×10^{-5}	21.31	51.20
Gm47260	1.26	1.20×10^{-4}	19.45	46.70
Il18bp	1.26	6.56×10^{-6}	32.23	77.25
Klhdc7a	1.26	3.72×10^{-14}	62.36	149.47
Gm17491	1.26	1.46×10^{-5}	33.34	79.85
Cntnap1	1.26	3.86×10^{-11}	64.76	155.01
Itga10	1.26	3.60×10^{-7}	28.50	68.15
Myof	1.26	4.87×10^{-9}	59.60	142.30
Vnn1	1.25	2.19×10^{-3}	9.62	22.97
Reln	1.25	1.11×10^{-29}	3,543.93	8,454.02
Cdc14b	1.25	5.65×10^{-7}	29.50	70.30
AB124611	1.25	7.87×10^{-4}	7.87	18.73
9530056E24Rik	1.25	2.32×10^{-2}	7.44	17.70
Lgals4	1.25	5.33×10^{-4}	11.20	26.66
Ccdc187	1.25	4.50×10^{-9}	33.11	78.64
Tgfbr3	1.25	1.80×10^{-17}	80.67	191.33
Gm44437	1.25	1.32×10^{-2}	12.49	29.62
Mirg	1.25	7.98×10^{-8}	83.37	197.70
Thpo	1.25	1.09×10^{-2}	6.47	15.33
Gm49599	1.24	3.80×10^{-2}	12.01	28.42
Elmo3	1.24	9.97×10^{-4}	8.64	20.44
Col27a1	1.24	6.28×10^{-7}	58.88	139.00
Bhlhe41	1.24	7.16×10^{-9}	44.71	105.53
Calhm2	1.24	3.83×10^{-4}	10.16	23.97
Lime1	1.24	3.60×10^{-8}	167.36	394.76
Krt8	1.24	2.86×10^{-5}	16.35	38.53
Gm50253	1.24	2.38×10^{-3}	27.84	65.53
Mir99ahg	1.23	1.60×10^{-4}	79.03	185.79
mt-Nd4	1.23	3.06×10^{-5}	8,510.72	20,000.20
Mrc1	1.23	2.76×10^{-20}	217.11	510.15
Gm6145	1.23	3.21×10^{-5}	22.35	52.49
A330076H08Rik	1.23	3.64×10^{-3}	20.39	47.89
Gm44758	1.23	2.23×10^{-2}	8.90	20.90
9430064I24Rik	1.23	3.74×10^{-4}	18.17	42.63
Pdlim1	1.23	6.67×10^{-5}	23.44	54.94
2700029L08Rik	1.23	4.40×10^{-2}	7.69	18.03
Hkdc1	1.23	1.64×10^{-2}	6.25	14.64
Prox2	1.23	1.36×10^{-2}	6.87	16.07
Npas4	1.23	9.07×10^{-6}	29.53	69.05

Gene ID	Log2 Fold Change	p-adj	Control CPM	Rad CPM
Gm44901	1.23	1.88×10^{-2}	8.65	20.22
Gm40309	1.22	4.53×10^{-2}	6.41	14.97
A930004J17Rik	1.22	8.85×10^{-4}	33.29	77.73
Gm3764	1.22	6.17×10^{-11}	700.67	1,635.27
Lama3	1.22	3.12×10^{-6}	41.04	95.72
4930518I15Rik	1.22	2.48×10^{-2}	8.44	19.65
Spta1	1.22	9.31×10^{-3}	12.40	28.88
Catsperg2	1.22	2.12×10^{-3}	11.71	27.26
Gm12940	1.22	2.60×10^{-2}	66.48	154.65
Mst1r	1.22	1.01×10^{-4}	28.28	65.77
H2-M5	1.21	8.60×10^{-6}	37.28	86.53
Gm37139	1.21	3.37×10^{-2}	10.67	24.70
Snai2	1.21	3.23×10^{-3}	9.13	21.14
Fcna	1.21	6.34×10^{-6}	25.34	58.62
Gm43843	1.21	6.28×10^{-4}	22.41	51.72
C3ar1	1.21	1.20×10^{-6}	26.41	60.91
Parp3	1.20	1.89×10^{-2}	8.84	20.37
Dock5	1.20	2.37×10^{-8}	45.67	104.97
Foxc2	1.20	4.40×10^{-6}	46.35	106.50
Foxb1	1.20	4.53×10^{-2}	6.55	15.05
Kcnq1ot1	1.20	1.42×10^{-3}	536.63	1,231.69
Onecut3	1.20	1.21×10^{-2}	15.07	34.51
Fat2	1.19	3.78×10^{-3}	12.04	27.56
Rtel1	1.19	9.23×10^{-16}	244.71	559.34
2600014E21Rik	1.19	1.02×10^{-3}	25.00	57.11
Casr	1.19	2.69×10^{-2}	6.93	15.79
Muc1	1.19	8.68×10^{-4}	15.12	34.45
Gucy2e	1.19	1.82×10^{-3}	8.32	18.96
Ssc5d	1.18	6.61×10^{-7}	27.60	62.73
Cntrl	1.18	1.12×10^{-9}	217.28	493.36
Cfap65	1.18	4.16×10^{-4}	21.51	48.84
Gm43305	1.18	9.26×10^{-4}	12.19	27.65
Gm42853	1.18	1.47×10^{-3}	29.47	66.83
Slc11a1	1.18	4.39×10^{-3}	15.02	34.05
Pidd1	1.18	4.04×10^{-11}	71.75	162.65
AC107792.1	1.18	1.36×10^{-2}	6.26	14.17
Pqlc3	1.18	1.24×10^{-5}	14.16	32.01
Cp	1.17	7.35×10^{-10}	111.91	252.42
Csf2rb2	1.17	3.94×10^{-2}	7.31	16.47

Gene ID	Log2 Fold Change	p-adj	Control CPM	Rad CPM
Lama5	1.17	2.73×10^{-3}	151.58	341.59
Foxm1	1.17	6.31×10^{-15}	261.15	588.21
Mfsd2b	1.17	6.95×10^{-5}	15.27	34.37
Slc22a2	1.17	5.69×10^{-8}	25.72	57.83
Gm44433	1.17	1.75×10^{-2}	27.44	61.67
Ifitm3	1.17	4.42×10^{-9}	81.22	182.52
Gm45457	1.17	4.61×10^{-2}	11.62	26.06
Tmprss5	1.17	1.49×10^{-2}	6.19	13.88
Itgal	1.16	8.64×10^{-3}	12.92	28.97
Ifi202b	1.16	1.75×10^{-2}	6.97	15.60
Gm37090	1.16	1.20×10^{-2}	9.27	20.73
E030022I16Rik	1.16	3.87×10^{-2}	14.06	31.43
Svep1	1.16	1.17×10^{-8}	67.24	150.25
Cxcl16	1.16	2.97×10^{-3}	15.93	35.58
Tcirg1	1.16	8.95×10^{-8}	95.78	213.80
Gm2824	1.16	4.11×10^{-4}	14.20	31.68
Cd300a	1.16	4.08×10^{-6}	18.54	41.34
Gbp4	1.16	1.70×10^{-4}	23.72	52.90
Spaca6	1.16	1.44×10^{-5}	113.01	251.87
Irgm2	1.15	5.77×10^{-4}	13.60	30.24
Onecut1	1.15	4.25×10^{-2}	11.59	25.77
Lin28b	1.15	6.15×10^{-8}	60.57	134.68
Rasa4	1.15	3.21×10^{-8}	67.71	150.56
Dsp	1.15	1.27×10^{-9}	106.06	235.80
Slc15a3	1.15	7.55×10^{-4}	15.86	35.25
Six4	1.15	4.49×10^{-3}	7.69	17.09
Podn	1.15	3.49×10^{-7}	38.54	85.58
5031434O11Rik	1.15	3.42×10^{-8}	29.91	66.40
Slc13a4	1.15	7.70×10^{-16}	112.08	248.65
Proca1	1.15	8.93×10^{-3}	8.57	19.00
Vwa5b2	1.15	1.44×10^{-3}	153.95	341.49
Mapkapk3	1.15	4.11×10^{-4}	14.68	32.55
Cpeb1	1.15	1.98×10^{-3}	24.40	54.04
Cdh1	1.15	3.08×10^{-4}	37.86	83.83
Il33	1.15	2.59×10^{-2}	6.50	14.40
Txlnb	1.14	1.51×10^{-2}	6.81	15.06
Gbp7	1.14	7.33×10^{-7}	33.61	74.26
Elf4	1.14	1.28×10^{-6}	31.57	69.73
Ccdc162	1.14	1.02×10^{-2}	10.90	24.02

Gene ID	Log2 Fold Change	p-adj	Control CPM	Rad CPM
Ikbke	1.14	1.62×10^{-3}	14.58	32.10
Slc4a11	1.14	1.63×10^{-2}	6.64	14.63
Tomm6os	1.14	4.77×10^{-4}	12.13	26.71
Thbd	1.14	1.15×10^{-7}	160.13	352.19
9930111J21Rik2	1.14	1.21×10^{-2}	12.39	27.22
Gm37019	1.13	2.63×10^{-2}	6.56	14.41
Gli1	1.13	4.86×10^{-8}	102.74	225.41
Hapln4	1.13	2.61×10^{-2}	10.31	22.51
Car13	1.13	7.74×10^{-3}	15.87	34.65
Ccdc194	1.13	1.81×10^{-2}	6.92	15.10
Gbp11	1.12	5.05×10^{-3}	6.68	14.56
Epor	1.12	1.21×10^{-2}	8.01	17.47
Col18a1	1.12	1.25×10^{-23}	369.61	805.19
5830487J09Rik	1.12	4.83×10^{-2}	8.61	18.74
Fam20a	1.12	1.28×10^{-7}	27.61	60.04
Adamts12	1.12	3.04×10^{-12}	133.75	290.73
Gjb2	1.12	1.22×10^{-13}	81.63	177.07
1700109H08Rik	1.12	1.56×10^{-2}	12.70	27.53
Tnfrsf10b	1.12	1.45×10^{-4}	15.52	33.65
Trabd2b	1.12	4.14×10^{-6}	45.18	97.93
Gm49396	1.12	2.36×10^{-5}	73.43	159.12
Slc16a11	1.11	5.31×10^{-3}	33.52	72.55
Gm10134	1.11	8.64×10^{-4}	13.55	29.32
AU020206	1.11	2.51×10^{-7}	47.28	102.29
Robo3	1.11	4.13×10^{-3}	20.42	44.12
Ackr2	1.11	3.97×10^{-3}	34.76	74.98
Cyp1b1	1.11	7.20×10^{-12}	100.55	216.78
Gm45501	1.11	1.41×10^{-2}	10.41	22.42
Tmem140	1.11	2.12×10^{-3}	11.84	25.50
Ebf3	1.11	1.49×10^{-11}	124.95	269.05
1700020D05Rik	1.11	4.44×10^{-2}	6.73	14.49
Lef1	1.11	1.00×10^{-10}	95.80	206.07
Ptpn14	1.10	3.58×10^{-9}	101.25	217.76
Pnisr	1.10	9.84×10^{-14}	969.93	2,085.01
A530058N18Rik	1.10	2.43×10^{-2}	7.97	17.14
Fmn1	1.10	3.33×10^{-2}	17.49	37.59
Etl4	1.10	1.43×10^{-10}	215.87	463.75
Tmem72	1.10	5.73×10^{-4}	74.62	160.25
Gm38253	1.10	2.62×10^{-2}	7.56	16.24

Gene ID	Log2 Fold Change	p-adj	Control CPM	Rad CPM
Adamts9	1.10	1.31×10^{-13}	153.40	328.80
Tnfrsf23	1.10	3.65×10^{-2}	12.36	26.49
Slc22a21	1.10	3.44×10^{-3}	18.12	38.84
Gmnc	1.10	3.13×10^{-2}	7.38	15.80
Dab2	1.10	1.07×10^{-14}	275.02	589.16
Eml5	1.10	1.03×10^{-10}	418.34	894.47
Rtp4	1.10	2.63×10^{-3}	21.07	45.03
Sidt1	1.10	8.83×10^{-18}	152.62	326.12
Crip3	1.09	1.53×10^{-2}	8.75	18.68
Foxd1	1.09	1.44×10^{-4}	57.12	121.83
Gm37206	1.09	1.99×10^{-4}	36.60	77.98
Onecut2	1.09	8.50×10^{-6}	99.04	210.95
Zc3h7a	1.09	1.81×10^{-16}	328.78	700.12
Gm37333	1.09	3.37×10^{-2}	14.30	30.37
Ano5	1.09	1.57×10^{-3}	14.15	30.04
Adamts10	1.09	2.01×10^{-15}	390.43	828.63
Ppp1r3b	1.09	1.23×10^{-6}	67.36	142.89
Col4a3	1.08	1.07×10^{-2}	8.29	17.57
Malat1	1.08	3.46×10^{-2}	8,695.76	18,430.21
Lgals3bp	1.08	6.69×10^{-6}	58.18	123.28
Pfkp	1.08	2.07×10^{-10}	1,711.84	3,625.68
Adrb2	1.08	3.54×10^{-3}	9.56	20.21
Ms4a6b	1.08	5.17×10^{-4}	14.18	29.96
Gm37694	1.08	6.55×10^{-3}	10.65	22.47
Trp73	1.08	3.46×10^{-23}	246.71	520.40
Cacna1g	1.08	3.32×10^{-10}	972.84	2,051.76
Polq	1.08	1.87×10^{-5}	69.32	146.16
Gtse1	1.07	1.72×10^{-7}	71.41	150.35
Mki67	1.07	1.09×10^{-9}	983.09	2,069.45
Cd274	1.07	7.31×10^{-6}	25.41	53.42
Itih2	1.07	8.01×10^{-8}	47.97	100.81
Arhgap19	1.07	1.31×10^{-5}	30.28	63.62
A730011C13Rik	1.07	4.11×10^{-3}	10.81	22.66
Stac3	1.07	1.67×10^{-2}	9.65	20.21
Unc45b	1.07	2.32×10^{-4}	22.25	46.57
Plxna3	1.07	4.03×10^{-5}	811.50	1,698.50
Mmp23	1.07	1.09×10^{-3}	15.43	32.28
Nid2	1.07	1.10×10^{-11}	493.45	1,032.43
Mir17hg	1.06	1.27×10^{-3}	30.85	64.54

Gene ID	Log2 Fold Change	p-adj	Control CPM	Rad CPM
Ubxn11	1.06	3.16×10^{-9}	54.76	114.26
D430036J16Rik	1.06	1.02×10^{-3}	15.69	32.73
Sh3d21	1.06	1.15×10^{-2}	8.03	16.73
Gm48062	1.06	1.32×10^{-2}	8.31	17.32
Leng8	1.06	2.51×10^{-11}	1,334.89	2,775.92
Mrgprf	1.06	4.17×10^{-10}	51.26	106.55
Cpz	1.05	3.99×10^{-2}	18.97	39.30
Gm29666	1.05	1.94×10^{-3}	21.34	44.15
mt-Co2	1.05	1.33×10^{-3}	3,296.74	6,817.13
Wnt11	1.04	2.23×10^{-2}	9.79	20.19
Alas2	1.04	2.26×10^{-3}	477.95	985.58
Hif3a	1.04	2.41×10^{-9}	188.15	387.65
Gm44769	1.04	3.77×10^{-3}	15.83	32.62
Ncf1	1.04	7.61×10^{-7}	35.04	72.10
Carmil3	1.04	1.14×10^{-13}	1,178.44	2,424.68
Gm21781	1.04	9.77×10^{-6}	87.84	180.50
Ushbp1	1.04	3.27×10^{-9}	59.29	121.83
Dcxr	1.04	1.19×10^{-5}	31.71	65.10
Aifm3	1.04	3.80×10^{-10}	100.37	205.92
Gm47573	1.04	4.05×10^{-3}	16.39	33.61
Ybx2	1.03	1.71×10^{-4}	47.78	97.90
Unc80	1.03	2.82×10^{-6}	226.90	464.70
Spag5	1.03	4.00×10^{-7}	165.53	338.63
Tmem106a	1.03	1.49×10^{-4}	16.83	34.43
Lepr	1.03	2.12×10^{-8}	91.87	187.92
Pik3ap1	1.03	4.07×10^{-6}	32.57	66.62
Gm44250	1.03	1.71×10^{-3}	22.11	45.19
Gm38190	1.03	6.09×10^{-3}	16.94	34.58
Dennd1c	1.03	3.11×10^{-3}	37.25	76.03
Aspdh	1.03	3.97×10^{-3}	16.45	33.55
Nrbp2	1.03	3.93×10^{-11}	829.53	1,692.20
Enpp1	1.03	3.37×10^{-6}	46.97	95.82
Gm26672	1.03	4.86×10^{-3}	13.76	28.08
Snai1	1.03	1.39×10^{-4}	27.69	56.46
Kcnj5	1.03	1.13×10^{-4}	24.27	49.45
Gm5577	1.03	1.25×10^{-3}	23.73	48.31
Mertk	1.03	2.42×10^{-7}	30.66	62.41
Col16a1	1.03	2.27×10^{-5}	204.00	415.20
Espl1	1.02	8.04×10^{-8}	142.84	290.46

Gene ID	Log2 Fold Change	p-adj	Control CPM	Rad CPM
mt-Cytb	1.02	1.65×10^{-4}	15,612.79	31,741.87
Cdk18	1.02	6.60×10^{-4}	23.96	48.60
Sim1	1.02	5.25×10^{-3}	21.59	43.71
6430511E19Rik	1.02	6.67×10^{-3}	12.26	24.80
Hk3	1.02	2.27×10^{-3}	12.07	24.43
D930036K23Rik	1.02	2.51×10^{-2}	7.97	16.13
Cenpf	1.02	6.77×10^{-6}	436.55	882.61
Gm28042	1.01	3.00×10^{-4}	51.22	103.41
Nbeal2	1.01	2.01×10^{-8}	152.91	308.63
Angptl2	1.01	4.59×10^{-12}	163.69	330.26
Eif2ak2	1.01	8.85×10^{-6}	61.61	124.24
Kif15	1.01	1.95×10^{-5}	135.42	273.07
Prss53	1.01	4.17×10^{-2}	10.16	20.47
Pou2f2	1.01	6.39×10^{-7}	260.67	524.86
Col6a5	1.01	4.03×10^{-2}	15.56	31.33
Gm43549	1.01	3.99×10^{-2}	6.97	14.02
Sfi1	1.01	4.88×10^{-11}	552.03	1,109.78
Lhx1	1.01	2.50×10^{-4}	46.10	92.58
Aox3	1.00	1.85×10^{-2}	11.16	22.38
Lama4	1.00	1.26×10^{-7}	393.71	789.79
Uba7	1.00	3.22×10^{-2}	19.00	38.07
Rnf207	1.00	2.43×10^{-2}	13.52	27.08
Gm20342	1.00	2.24×10^{-3}	49.09	98.31
A2m	1.00	8.62×10^{-13}	146.41	292.96
Map4k2	1.00	4.86×10^{-7}	328.62	657.02
mt-Co1	1.00	1.73×10^{-4}	31,238.36	62,435.98
Cubn	1.00	9.06×10^{-3}	13.36	26.67
Plekha2	1.00	6.15×10^{-6}	42.78	85.38
Mir124a-1hg	1.00	6.31×10^{-4}	817.31	1,629.69
Vwa3a	1.00	4.11×10^{-3}	30.76	61.33
Srrm2	0.99	8.55×10^{-12}	5,029.62	10,021.13
Lrriq1	0.99	3.34×10^{-3}	23.29	46.32
Gm43162	0.99	3.18×10^{-2}	7.85	15.59
Nhlh1	0.99	5.98×10^{-4}	68.60	136.15
Kntc1	0.99	3.41×10^{-4}	119.70	237.22
Tent5c	0.99	6.25×10^{-6}	77.04	152.63
Cdh3	0.98	6.37×10^{-6}	30.31	59.98
Hspg2	0.98	4.03×10^{-6}	661.65	1,309.05
Foxr2	0.98	1.13×10^{-2}	10.09	19.97

Gene ID	Log2 Fold Change	p-adj	Control CPM	Rad CPM
Masp2	0.98	2.30×10^{-4}	31.59	62.41
mt-Nd1	0.98	1.31×10^{-3}	9,137.70	18,047.49
Scarf2	0.98	4.22×10^{-6}	80.07	157.83
Pld4	0.98	3.57×10^{-6}	41.00	80.76
Aph1c	0.98	3.30×10^{-3}	45.92	90.44
Slc2a12	0.98	7.49×10^{-17}	107.39	211.39
Dnase1l2	0.98	2.24×10^{-4}	32.24	63.42
Mmp19	0.97	9.03×10^{-3}	9.26	18.19
Stard9	0.97	1.71×10^{-8}	165.65	325.12
Pyroxd2	0.97	2.59×10^{-5}	25.38	49.77
AI661453	0.97	6.20×10^{-5}	21.10	41.37
Ece2	0.97	6.95×10^{-7}	146.65	287.16
Arhgef39	0.97	4.64×10^{-4}	40.79	79.81
Hebp2	0.97	1.15×10^{-3}	19.11	37.38
Gm5628	0.97	1.61×10^{-2}	8.42	16.46
L3mbtl1	0.97	9.46×10^{-5}	46.13	90.17
Plau	0.97	1.13×10^{-4}	40.91	79.95
Iqgap3	0.97	1.43×10^{-6}	108.33	211.66
Card14	0.97	3.30×10^{-2}	10.18	19.90
Cln6	0.97	2.03×10^{-8}	72.30	141.16
Clic6	0.97	3.18×10^{-14}	233.61	456.07
Icam1	0.97	7.00×10^{-6}	40.34	78.75
2610035F20Rik	0.96	8.53×10^{-3}	9.20	17.95
Csf2rb	0.96	2.29×10^{-3}	16.62	32.41
Zfp967	0.96	4.41×10^{-2}	9.13	17.79
Gm29695	0.96	7.16×10^{-6}	83.32	162.40
Helz2	0.96	2.16×10^{-6}	54.19	105.58
Gm49405	0.96	2.41×10^{-4}	64.32	125.29
Gm50107	0.96	7.57×10^{-6}	42.61	82.85
Dok3	0.96	6.35×10^{-5}	35.01	68.07
Dgkq	0.96	1.10×10^{-11}	260.36	505.93
Obscn	0.96	1.30×10^{-4}	116.26	225.91
Irgm1	0.96	1.63×10^{-5}	30.84	59.80
BC022960	0.95	5.29×10^{-3}	26.05	50.48
Acss3	0.95	2.08×10^{-3}	12.71	24.63
Trank1	0.95	2.39×10^{-9}	1,102.73	2,135.79
Abca9	0.95	1.50×10^{-9}	158.28	306.55
Gask1b	0.95	8.81×10^{-3}	45.86	88.81
Otof	0.95	4.70×10^{-10}	120.02	232.05

Gene ID	Log2 Fold Change	p-adj	Control CPM	Rad CPM
Fanca	0.95	1.55×10^{-3}	49.71	96.10
Lrrc45	0.95	2.81×10^{-10}	449.87	869.08
Efemp1	0.95	2.06×10^{-2}	24.20	46.72
Rgl3	0.95	1.20×10^{-2}	15.47	29.87
Gm43300	0.95	6.81×10^{-3}	21.16	40.83
Clcn1	0.95	3.12×10^{-3}	30.95	59.70
Ism2	0.95	2.49×10^{-2}	12.76	24.61
Ccdc114	0.95	3.00×10^{-6}	28.63	55.13
Rmst	0.94	2.93×10^{-2}	41.83	80.44
Slc22a6	0.94	9.22×10^{-16}	178.35	342.92
Neurod4	0.94	2.84×10^{-2}	20.14	38.64
Slc7a11	0.94	9.23×10^{-10}	251.31	482.15
Ranbp3l	0.94	1.33×10^{-6}	73.67	141.27
Arhgap30	0.94	1.01×10^{-3}	25.69	49.25
E330034L11Rik	0.94	3.05×10^{-5}	29.26	56.08
Bmp7	0.94	1.91×10^{-17}	315.38	604.32
Kcnt1	0.94	1.50×10^{-6}	353.52	676.11
Mitf	0.94	1.20×10^{-5}	32.06	61.31
Stxbp2	0.93	3.91×10^{-9}	220.80	422.12
Cspp1	0.93	5.61×10^{-8}	319.63	610.85
Wdr90	0.93	3.10×10^{-6}	287.86	549.39
Igf2	0.93	8.02×10^{-20}	4,702.18	8,968.96
Cacna2d2	0.93	6.90×10^{-12}	860.15	1,639.66
Wdr62	0.93	4.91×10^{-2}	72.02	137.23
4731419I09Rik	0.93	4.76×10^{-2}	7.34	13.99
Fcer1g	0.93	1.11×10^{-6}	39.13	74.49
Lcp2	0.93	1.14×10^{-2}	31.81	60.53
2500002B13Rik	0.93	4.12×10^{-2}	13.89	26.44
Lhx9	0.93	2.36×10^{-3}	127.82	243.24
Gm50322	0.93	2.86×10^{-3}	15.93	30.29
Hcar1	0.93	2.83×10^{-3}	14.78	28.11
9630028B13Rik	0.93	2.32×10^{-4}	29.82	56.66
Mov10	0.93	8.86×10^{-12}	88.62	168.29
Amy1	0.93	1.42×10^{-4}	106.69	202.57
2610037D02Rik	0.93	3.16×10^{-3}	26.44	50.19
Gm38077	0.92	4.66×10^{-2}	13.65	25.92
Cenpe	0.92	2.47×10^{-4}	233.75	443.66
Angpt2	0.92	3.27×10^{-6}	62.93	119.41
Rgl2	0.92	3.53×10^{-7}	422.05	800.14

Gene ID	Log2 Fold Change	p-adj	Control CPM	Rad CPM
Cfap46	0.92	1.17×10^{-2}	34.00	64.45
Mrvi1	0.92	5.20×10^{-3}	28.14	53.29
4930539E08Rik	0.92	8.26×10^{-4}	41.26	78.10
Prodh	0.92	2.58×10^{-4}	31.41	59.46
Apod	0.92	1.12×10^{-15}	428.53	809.64
Parp12	0.92	6.17×10^{-9}	52.86	99.75
A4galt	0.92	1.09×10^{-2}	23.20	43.77
Clspn	0.91	3.03×10^{-4}	104.79	197.41
Rreb1	0.91	1.29×10^{-5}	119.64	225.29
Car10	0.91	7.49×10^{-10}	245.45	461.74
Lamb2	0.91	1.29×10^{-12}	412.70	775.45
Rab7-ps1	0.91	8.48×10^{-3}	11.79	22.14
Arap1	0.91	2.42×10^{-4}	208.30	391.37
Fn1	0.91	3.01×10^{-9}	1,441.91	2,707.48
Il3ra	0.91	3.97×10^{-3}	20.96	39.35
Ap1g2	0.91	3.96×10^{-4}	64.18	120.48
Pla2g6	0.91	3.41×10^{-10}	214.74	403.02
Bmp4	0.91	2.14×10^{-3}	38.46	72.17
Hspb8	0.91	2.94×10^{-6}	83.43	156.55
Mrc2	0.91	1.55×10^{-6}	228.52	428.69
Tchh	0.91	1.57×10^{-3}	23.18	43.44
Man2b2	0.91	6.58×10^{-13}	152.34	285.45
Car12	0.91	3.36×10^{-13}	183.43	343.52
Ptgds	0.90	1.00×10^{-6}	1,317.33	2,463.32
Piezo1	0.90	1.21×10^{-15}	433.52	810.58
Tex9	0.90	2.69×10^{-8}	142.94	267.23
Shfl	0.90	6.99×10^{-9}	111.31	207.90
Mamdc4	0.90	1.43×10^{-2}	16.81	31.40
Gm47033	0.90	8.85×10^{-3}	17.12	31.97
Ccn4	0.90	1.19×10^{-2}	25.26	47.17
Phykpl	0.90	6.80×10^{-4}	23.49	43.85
Alx4	0.90	2.86×10^{-5}	69.95	130.53
Mir100hg	0.90	8.42×10^{-6}	1,276.54	2,381.65
Crocc	0.90	1.54×10^{-8}	445.39	830.72
Isg15	0.90	4.05×10^{-2}	14.85	27.68
Itih5	0.90	1.84×10^{-14}	280.73	523.19
Adra2b	0.90	3.29×10^{-2}	11.44	21.33
Magel2	0.90	2.88×10^{-8}	221.44	412.37
2900040C04Rik	0.90	1.48×10^{-3}	13.82	25.72

Gene ID	Log2 Fold Change	p-adj	Control CPM	Rad CPM
Tfap2c	0.90	1.30×10^{-7}	150.95	280.97
Sigirr	0.89	1.32×10^{-2}	12.96	24.09
mt-Nd5	0.89	1.51×10^{-2}	4,593.09	8,529.82
Vwf	0.89	3.11×10^{-3}	259.28	481.50
Gm3448	0.89	7.51×10^{-3}	23.78	44.14
Stat6	0.89	3.30×10^{-8}	80.90	150.19
Gm49593	0.89	4.95×10^{-2}	8.73	16.20
Fgfbp1	0.89	1.22×10^{-5}	65.57	121.66
Pabpc4l	0.89	4.09×10^{-2}	14.63	27.14
Slc16a12	0.89	6.95×10^{-5}	47.52	88.14
Pnpla7	0.89	1.01×10^{-6}	60.91	112.95
Lgals9	0.89	2.14×10^{-6}	83.73	155.08
Lox	0.89	7.43×10^{-9}	303.44	561.51
Arhgef1	0.89	2.59×10^{-10}	729.95	1,350.19
Cep164	0.89	3.21×10^{-9}	289.26	534.96
Gm48485	0.89	3.84×10^{-2}	8.27	15.30
Baiap3	0.89	8.16×10^{-6}	198.79	367.49
Grk3	0.89	3.70×10^{-12}	466.91	862.86
Vegfa	0.89	2.40×10^{-13}	582.85	1,076.67
Irak4	0.89	3.64×10^{-3}	15.10	27.89
Slc16a10	0.89	3.97×10^{-4}	37.84	69.90
Hmmr	0.88	4.76×10^{-6}	100.47	185.39
Dock6	0.88	2.37×10^{-5}	330.17	609.12
Bicc1	0.88	4.58×10^{-10}	142.20	262.26
Zfhx2	0.88	6.64×10^{-7}	547.43	1,009.60
Tfcp2l1	0.88	2.02×10^{-3}	39.00	71.85
E2f2	0.88	2.26×10^{-5}	66.39	122.28
Samd3	0.88	1.38×10^{-2}	33.99	62.57
Man2c1	0.88	1.51×10^{-8}	518.87	955.06
Fam228a	0.88	2.64×10^{-2}	10.64	19.57
Ints6l	0.88	7.77×10^{-9}	187.74	345.39
Psrc1	0.88	4.95×10^{-7}	117.79	216.68
Gm12992	0.88	9.87×10^{-3}	14.53	26.71
Rad54l	0.88	2.47×10^{-4}	74.97	137.84
Mpeg1	0.88	6.88×10^{-11}	135.55	249.15
Trem2	0.88	1.89×10^{-3}	39.74	72.97
H2-K1	0.88	2.14×10^{-9}	179.32	329.21
D930016D06Rik	0.88	8.23×10^{-9}	139.44	255.97
Tifab	0.88	1.00×10^{-2}	20.10	36.89

Gene ID	Log2 Fold Change	p-adj	Control CPM	Rad CPM
Fign	0.88	4.68×10^{-5}	126.47	232.07
Gm8909	0.87	1.87×10^{-2}	11.88	21.78
Emp3	0.87	1.40×10^{-4}	32.99	60.48
Hba-a2	0.87	5.04×10^{-3}	17,442.45	31,965.46
B2m	0.87	3.45×10^{-7}	159.94	293.05
4632427E13Rik	0.87	3.47×10^{-2}	50.69	92.84
Syne1	0.87	6.57×10^{-12}	731.10	1,338.66
Ogn	0.87	3.41×10^{-9}	187.77	343.79
Plec	0.87	5.50×10^{-7}	591.57	1,082.95
Myo19	0.87	1.28×10^{-6}	111.95	204.92
Slc6a13	0.87	2.85×10^{-14}	250.86	459.07
Fes	0.87	8.06×10^{-6}	48.25	88.25
Gpt	0.87	1.73×10^{-6}	83.23	152.21
Col5a1	0.87	6.09×10^{-3}	514.91	940.85
Gm38317	0.87	2.79×10^{-3}	13.80	25.21
Syt15	0.87	1.65×10^{-2}	13.29	24.26
Kank2	0.87	1.12×10^{-9}	188.78	344.35
Jag1	0.87	3.78×10^{-8}	228.82	417.31
Lrrk1	0.87	5.74×10^{-4}	85.63	156.13
Cep250	0.87	6.28×10^{-10}	462.29	842.60
Adcy4	0.87	1.55×10^{-3}	39.46	71.90
Gm13563	0.87	5.11×10^{-3}	26.89	48.98
Rin1	0.86	6.34×10^{-4}	51.62	94.00
Six5	0.86	6.61×10^{-7}	62.31	113.43
Myh11	0.86	1.36×10^{-3}	25.48	46.37
Cfp	0.86	5.87×10^{-4}	59.75	108.60
Grk4	0.86	9.62×10^{-3}	17.01	30.91
Tap2	0.86	1.19×10^{-4}	29.03	52.65
Zgrf1	0.86	2.30×10^{-4}	88.68	160.78
Cdca2	0.86	9.24×10^{-5}	120.91	219.21
Tspoap1	0.86	2.40×10^{-14}	1,268.69	2,298.70
Scn5a	0.86	8.29×10^{-12}	171.56	310.82
Fxyd5	0.86	9.74×10^{-5}	71.82	130.11
Efcc1	0.86	4.88×10^{-2}	26.12	47.30
Snx20	0.86	2.91×10^{-2}	7.49	13.56
Adgre1	0.86	1.69×10^{-4}	51.42	93.07
Ebf2	0.86	1.53×10^{-5}	44.38	80.28
Gm45250	0.85	2.37×10^{-3}	21.12	38.15
Hydin	0.85	3.65×10^{-2}	20.29	36.63

Gene ID	Log2 Fold Change	p-adj	Control CPM	Rad CPM
Igsf6	0.85	2.87×10^{-2}	8.60	15.52
Trpv4	0.85	4.34×10^{-4}	22.55	40.69
Rmi2	0.85	9.02×10^{-3}	23.06	41.59
Tonsl	0.85	2.64×10^{-3}	153.07	275.99
Lrrc25	0.85	3.39×10^{-2}	12.18	21.94
Ano1	0.85	9.05×10^{-4}	89.30	160.79
Smad6	0.85	9.24×10^{-5}	47.12	84.84
Alpk1	0.85	8.54×10^{-4}	44.11	79.41
Itpr2	0.85	6.06×10^{-4}	154.84	278.57
Gm19040	0.85	1.86×10^{-2}	10.59	19.06
Serpinb1b	0.85	1.89×10^{-2}	11.50	20.69
Kcnj13	0.85	2.69×10^{-3}	20.87	37.53
Gm4316	0.85	6.02×10^{-4}	41.25	74.18
Alox5ap	0.85	2.53×10^{-2}	20.43	36.72
Mecom	0.85	6.61×10^{-3}	45.78	82.29
Il17rc	0.85	1.11×10^{-2}	23.35	41.96
Adam12	0.85	7.49×10^{-8}	222.76	400.20
Hpgds	0.85	2.39×10^{-2}	36.92	66.33
mt-Co3	0.84	1.18×10^{-2}	6,590.53	11,835.83
Bub1b	0.84	7.80×10^{-7}	259.42	465.72
Zfyve26	0.84	3.63×10^{-5}	278.37	499.72
F5	0.84	3.19×10^{-2}	9.53	17.09
Col8a2	0.84	2.19×10^{-6}	180.54	323.48
Unc93b1	0.84	1.30×10^{-10}	89.19	159.68
Nek10	0.84	4.73×10^{-3}	16.81	30.07
Tmem191c	0.84	1.72×10^{-7}	173.66	310.61
Colec12	0.84	2.94×10^{-20}	419.93	750.85
Ttf2	0.84	5.94×10^{-7}	65.32	116.78
Hba-a1	0.84	4.90×10^{-3}	11,897.81	21,267.70
Ndnf	0.84	2.18×10^{-7}	486.69	869.89
Fermt3	0.84	5.56×10^{-5}	43.91	78.43
Gm42664	0.84	3.29×10^{-2}	54.98	98.19
Il1rl2	0.84	2.23×10^{-2}	10.44	18.64
Tmem35b	0.84	5.09×10^{-3}	23.45	41.85
Fancd2	0.84	1.28×10^{-4}	63.85	113.95
Cep192	0.84	6.78×10^{-4}	303.13	540.75
Dnah7b	0.83	1.00×10^{-2}	82.17	146.53
Sulf1	0.83	4.98×10^{-11}	355.08	632.88
Ddx11	0.83	8.77×10^{-5}	84.58	150.65

Gene ID	Log2 Fold Change	p-adj	Control CPM	Rad CPM
4930556M19Rik	0.83	3.55×10^{-2}	9.71	17.29
Clca3a1	0.83	2.03×10^{-2}	13.05	23.23
Cpne7	0.83	2.97×10^{-6}	177.78	316.41
Sgms2	0.83	3.98×10^{-2}	32.56	57.90
Aldh1a2	0.83	4.09×10^{-14}	222.74	396.05
Gm37584	0.83	1.35×10^{-2}	19.18	34.07
Lama1	0.83	1.10×10^{-2}	259.34	460.65
Ttc25	0.83	3.06×10^{-2}	14.71	26.13
Arhgap9	0.83	4.05×10^{-2}	8.44	14.98
Fhod1	0.83	2.74×10^{-5}	68.18	120.93
Gca	0.83	1.60×10^{-3}	54.14	95.95
Mfrp	0.83	2.17×10^{-3}	48.45	85.85
Ttll3	0.83	3.64×10^{-3}	58.81	104.21
Rbm20	0.82	1.23×10^{-7}	86.19	152.62
Bmp5	0.82	3.38×10^{-6}	51.57	91.28
Gabpb2	0.82	2.39×10^{-6}	248.34	439.01
Msx1	0.82	3.46×10^{-10}	107.61	190.14
Nktr	0.82	1.53×10^{-6}	1,160.78	2,050.38
Gm3052	0.82	4.25×10^{-2}	11.02	19.47
H2-Q4	0.82	2.79×10^{-6}	49.57	87.53
Lacc1	0.82	1.10×10^{-2}	15.81	27.91
Tnfrsf1b	0.82	6.30×10^{-6}	54.81	96.73
Tfap2b	0.82	2.99×10^{-2}	40.94	72.25
P2ry6	0.82	5.39×10^{-3}	25.94	45.76
Atoh8	0.82	7.49×10^{-3}	22.73	40.08
Ahnak2	0.82	1.55×10^{-3}	64.02	112.87
Cit	0.82	2.54×10^{-8}	377.72	665.42
Edn3	0.82	8.84×10^{-7}	69.99	123.30
Nupr1	0.82	1.02×10^{-2}	28.53	50.21
Apobec1	0.81	8.53×10^{-3}	26.97	47.43
Serpind1	0.81	5.09×10^{-3}	27.49	48.30
Ulk3	0.81	1.39×10^{-6}	138.01	242.43
Vwa5b1	0.81	9.09×10^{-4}	101.66	178.54
Cacna1a	0.81	7.60×10^{-11}	659.54	1,158.10
Slc6a20a	0.81	1.79×10^{-6}	105.88	185.91
Lrp4	0.81	2.66×10^{-11}	381.74	670.15
4933439C10Rik	0.81	6.74×10^{-5}	216.03	379.13
Slfn5	0.81	6.14×10^{-7}	62.94	110.32
Akr1b10	0.81	4.30×10^{-5}	31.24	54.77

Gene ID	Log2 Fold Change	p-adj	Control CPM	Rad CPM
Reck	0.81	9.69×10^{-10}	109.67	192.17
Wnk4	0.81	4.72×10^{-4}	67.86	118.86
Slc38a6	0.81	3.45×10^{-3}	43.31	75.84
Arhgap25	0.81	8.51×10^{-3}	25.08	43.91
Nadsyn1	0.81	3.92×10^{-3}	37.68	65.96
Gsdmd	0.81	3.14×10^{-3}	25.48	44.55
Tead3	0.81	2.68×10^{-6}	95.78	167.44
Tirap	0.81	7.02×10^{-3}	46.84	81.87
Col19a1	0.80	1.30×10^{-5}	73.23	127.89
Slc12a7	0.80	4.50×10^{-10}	266.50	465.43
Arhgap45	0.80	1.71×10^{-4}	34.22	59.77
Samd11	0.80	2.68×10^{-2}	16.67	29.10
Ddx58	0.80	9.31×10^{-5}	58.21	101.60
1700001L05Rik	0.80	9.95×10^{-3}	28.55	49.81
Bag3	0.80	9.61×10^{-8}	63.33	110.41
Sema3g	0.80	1.54×10^{-7}	57.75	100.67
Gm49322	0.80	2.54×10^{-2}	40.46	70.52
Tbx18	0.80	6.94×10^{-6}	99.35	173.13
Tram2	0.80	1.02×10^{-7}	101.89	177.53
Lmx1a	0.80	8.85×10^{-4}	22.80	39.71
Appl2	0.80	2.56×10^{-5}	254.52	443.33
Kcne2	0.80	3.37×10^{-8}	100.60	175.21
Pheta2	0.80	5.88×10^{-3}	16.98	29.55
Gli2	0.80	1.25×10^{-8}	163.72	284.51
Cgn	0.80	4.50×10^{-10}	149.17	259.21
Tmem86b	0.80	8.11×10^{-4}	52.99	92.04
Folr1	0.80	9.41×10^{-6}	134.62	233.68
Aox1	0.80	3.60×10^{-3}	17.18	29.82
Rhbdl3	0.80	5.60×10^{-7}	211.61	367.22
Cfap126	0.80	2.85×10^{-4}	30.43	52.80
Slc35g1	0.79	1.81×10^{-2}	21.93	38.05
Dtx3l	0.79	6.74×10^{-5}	37.70	65.39
Cyp4f13	0.79	1.05×10^{-3}	38.43	66.65
Tgm2	0.79	2.96×10^{-10}	146.69	254.40
Nfkbiz	0.79	2.17×10^{-2}	17.14	29.71
Ugt1a6a	0.79	2.72×10^{-2}	29.27	50.74
Kndc1	0.79	5.19×10^{-13}	614.74	1,065.17
Tpp1	0.79	2.34×10^{-9}	291.50	504.96
Pif1	0.79	5.42×10^{-3}	60.80	105.11

Gene ID	Log2 Fold Change	p-adj	Control CPM	Rad CPM
Vmn2r1	0.79	2.54×10^{-2}	11.09	19.17
Wnt4	0.79	6.32×10^{-11}	165.40	285.29
Mab21l1	0.79	2.17×10^{-7}	138.37	238.47
Tnfrsf11a	0.79	7.34×10^{-3}	25.20	43.42
Crispld2	0.79	2.83×10^{-4}	46.41	79.97
Gstt2	0.78	7.56×10^{-3}	19.24	33.13
Gm5089	0.78	1.22×10^{-3}	22.07	38.00
Rcsd1	0.78	6.01×10^{-9}	112.75	194.14
C030037D09Rik	0.78	5.02×10^{-3}	45.58	78.48
Jade2	0.78	9.02×10^{-6}	49.95	85.98
Tbxas1	0.78	3.84×10^{-2}	11.71	20.16
Cdkn1a	0.78	9.33×10^{-10}	455.42	783.82
Slc8b1	0.78	1.14×10^{-3}	32.70	56.27
Il10ra	0.78	9.62×10^{-3}	19.68	33.84
Zwilch	0.78	6.38×10^{-3}	70.05	120.33
Plxnb3	0.78	4.54×10^{-2}	10.28	17.66
Arhgef5	0.78	2.30×10^{-4}	68.83	118.22
Rgs14	0.78	8.07×10^{-3}	26.16	44.93
Ifitm1	0.78	4.88×10^{-3}	34.98	60.07
Arhgef40	0.78	3.71×10^{-8}	667.56	1,145.49
E330013P04Rik	0.78	3.49×10^{-2}	20.23	34.66
Pdzd7	0.78	3.98×10^{-4}	45.49	77.94
Zmynd15	0.78	4.35×10^{-2}	12.09	20.72
Axin2	0.78	6.42×10^{-11}	454.15	777.90
Aurkb	0.78	1.06×10^{-6}	174.21	298.37
Ppargc1b	0.78	1.62×10^{-3}	29.82	51.07
Cmbl	0.78	3.31×10^{-6}	60.65	103.87
Adgra2	0.78	3.64×10^{-10}	328.49	562.42
Ccdc142	0.78	3.73×10^{-2}	23.76	40.67
Ccdc173	0.78	2.07×10^{-2}	15.99	27.37
Synj2	0.78	6.02×10^{-4}	67.29	115.19
Gm49083	0.78	4.46×10^{-2}	10.92	18.69
Ttc21a	0.78	2.34×10^{-2}	22.08	37.79
Cldn11	0.77	1.94×10^{-5}	282.99	483.80
Pitpnm3	0.77	1.23×10^{-6}	224.08	383.05
Flna	0.77	2.21×10^{-7}	2,916.16	4,984.25
Pantr2	0.77	1.98×10^{-2}	22.80	38.96
Gm15328	0.77	2.01×10^{-2}	24.23	41.39
Kcnc2	0.77	8.55×10^{-8}	139.62	238.23

Gene ID	Log2 Fold Change	p-adj	Control CPM	Rad CPM
Dna2	0.77	3.11×10^{-3}	68.43	116.75
Tgfbi	0.77	2.04×10^{-4}	53.56	91.35
Neurog2	0.77	1.16×10^{-2}	283.59	483.60
Bora	0.77	1.68×10^{-3}	71.23	121.34
Szt2	0.77	1.95×10^{-6}	696.28	1,185.95
Creb3l1	0.77	1.54×10^{-3}	70.35	119.67
Bmp6	0.77	3.14×10^{-19}	290.68	494.26
Syne3	0.77	3.23×10^{-2}	18.96	32.23
Ifi27	0.77	1.39×10^{-3}	64.63	109.86
Fezf1	0.77	4.72×10^{-3}	99.51	169.15
Rxfp1	0.77	3.25×10^{-2}	31.00	52.69
BC067074	0.77	3.51×10^{-3}	52.96	90.00
Ghr	0.76	9.37×10^{-3}	61.48	104.45
Irf1	0.76	1.03×10^{-3}	27.60	46.87
2900052L18Rik	0.76	1.16×10^{-2}	31.30	53.12
Neil3	0.76	2.56×10^{-3}	67.02	113.69
Phldb2	0.76	6.51×10^{-4}	150.22	254.82
Arhgap4	0.76	7.14×10^{-3}	55.47	94.08
Ppfibp2	0.76	1.11×10^{-4}	38.26	64.89
Sesn2	0.76	4.81×10^{-5}	124.62	211.26
Adamts15	0.76	1.29×10^{-9}	161.46	273.64
Tlr2	0.76	2.46×10^{-3}	20.20	34.21
Cdc14a	0.76	2.57×10^{-4}	63.11	106.88
Ephx1	0.76	2.16×10^{-5}	84.89	143.74
Rnaset2b	0.76	1.25×10^{-2}	102.32	173.16
Tha1	0.76	1.47×10^{-3}	23.91	40.46
Knl1	0.76	2.24×10^{-4}	109.10	184.53
Phyhd1	0.76	2.88×10^{-3}	38.22	64.59
Paxbp1	0.76	1.28×10^{-5}	529.82	895.28
Lamc3	0.76	5.40×10^{-9}	156.02	263.61
Gli3	0.76	2.48×10^{-5}	259.96	438.99
Ttr	0.76	5.17×10^{-11}	9,336.81	15,766.19
P3h3	0.76	3.21×10^{-5}	303.76	512.90
Arid3a	0.76	7.15×10^{-9}	113.54	191.70
Csf1	0.75	2.85×10^{-11}	177.51	299.54
Glb1l	0.75	5.58×10^{-6}	119.18	201.08
Tcf7l1	0.75	2.27×10^{-6}	101.29	170.83
Spag1	0.75	4.94×10^{-5}	59.93	101.07
Sult1a1	0.75	1.76×10^{-4}	54.63	92.11

Gene ID	Log2 Fold Change	p-adj	Control CPM	Rad CPM
Gm45441	0.75	2.50×10^{-2}	17.23	29.06
Lrp2	0.75	2.80×10^{-3}	36.49	61.52
Pax6	0.75	1.53×10^{-5}	343.19	578.50
Ankrd16	0.75	7.46×10^{-5}	275.66	464.54
Scd3	0.75	1.58×10^{-2}	29.18	49.17
Aspm	0.75	4.13×10^{-2}	271.91	457.99
Tap1	0.75	5.37×10^{-4}	51.85	87.32
Slc26a11	0.75	6.06×10^{-4}	27.95	47.02
Fyco1	0.75	4.67×10^{-10}	229.33	385.79
Wnt6	0.75	2.04×10^{-3}	41.25	69.38
Zic5	0.75	2.40×10^{-8}	168.65	283.48
A830073O21Rik	0.75	3.59×10^{-2}	14.31	24.03
Olfml2a	0.75	1.33×10^{-5}	144.73	243.05
Gm37226	0.75	1.93×10^{-2}	23.12	38.81
Reep4	0.75	7.71×10^{-3}	50.68	85.05
Uhrf1	0.75	3.72×10^{-8}	409.80	687.37
AC099699.1	0.75	9.16×10^{-3}	76.36	128.07
Fgd6	0.74	1.15×10^{-5}	109.47	183.30
Cdkn3	0.74	2.02×10^{-2}	15.11	25.31
Oip5	0.74	6.52×10^{-3}	29.84	49.95
Trf	0.74	4.85×10^{-7}	218.07	365.03
Hspa1a	0.74	2.55×10^{-3}	55.70	93.20
Slc4a5	0.74	9.93×10^{-3}	54.28	90.82
Clasrp	0.74	5.31×10^{-6}	523.88	875.89
Anxa1	0.74	1.11×10^{-3}	56.92	95.16
Gm17396	0.74	1.53×10^{-2}	15.67	26.20
Car14	0.74	1.14×10^{-7}	78.39	130.98
Fam227a	0.74	4.55×10^{-3}	123.06	205.61
Lpp	0.74	3.19×10^{-4}	260.20	434.71
Tcaf2	0.74	2.96×10^{-2}	23.87	39.86
Ehd2	0.74	5.86×10^{-7}	154.36	257.67
Dse	0.74	1.48×10^{-7}	126.50	211.12
Med12	0.74	3.78×10^{-6}	624.45	1,041.53
Gm4890	0.74	4.20×10^{-2}	12.43	20.73
Rrbp1	0.74	3.27×10^{-6}	649.29	1,082.84
Lcp1	0.74	1.23×10^{-5}	174.92	291.70
Csf2ra	0.74	3.89×10^{-6}	222.55	371.11
Zfp469	0.74	1.79×10^{-3}	65.99	110.03
Celsr1	0.74	5.90×10^{-6}	845.47	1,409.34

Gene ID	Log2 Fold Change	p-adj	Control CPM	Rad CPM
Snapc4	0.74	7.50×10^{-7}	372.43	620.68
Dnah9	0.74	1.62×10^{-3}	72.15	120.23
Parp9	0.74	2.38×10^{-4}	43.34	72.22
Syt12	0.74	5.43×10^{-5}	52.69	87.78
Wdr86	0.74	2.55×10^{-6}	90.93	151.39
Oxtr	0.74	5.94×10^{-5}	73.62	122.54
Trim66	0.73	5.08×10^{-4}	52.41	87.22
Kank4	0.73	7.99×10^{-5}	41.69	69.37
Abcd4	0.73	5.58×10^{-6}	106.74	177.61
Adra1b	0.73	2.80×10^{-4}	70.90	117.95
Gm37928	0.73	4.89×10^{-2}	64.06	106.50
Slc6a7	0.73	2.31×10^{-6}	355.02	589.76
Tle2	0.73	3.71×10^{-5}	257.12	427.09
Laptm5	0.73	8.89×10^{-7}	230.20	382.35
Zfhx4	0.73	1.44×10^{-2}	452.31	751.11
Palb2	0.73	2.08×10^{-2}	37.59	62.42
Ankrd24	0.73	1.84×10^{-8}	228.58	379.48
Parp10	0.73	1.88×10^{-3}	33.05	54.86
Ace	0.73	3.97×10^{-7}	282.29	468.56
Islr	0.73	3.70×10^{-17}	312.22	518.21
Krt18	0.73	1.13×10^{-3}	30.75	51.03
Cfh	0.73	3.23×10^{-7}	302.57	502.11
Rin3	0.73	3.62×10^{-5}	79.43	131.77
Slc25a45	0.73	2.31×10^{-2}	22.22	36.85
Ryr2	0.73	2.17×10^{-5}	356.11	590.54
Tanc1	0.73	3.76×10^{-7}	176.01	291.80
Ticrr	0.73	7.78×10^{-4}	90.29	149.64
Recql4	0.73	6.81×10^{-3}	47.29	78.35
Cfap69	0.73	2.74×10^{-4}	96.92	160.57
Aldh1a1	0.73	4.96×10^{-4}	45.98	76.15
Cgas	0.73	3.51×10^{-2}	16.92	28.01
Copz2	0.73	1.76×10^{-3}	47.33	78.30
Ovgp1	0.73	2.02×10^{-2}	31.99	52.90
Arhgap24	0.72	1.85×10^{-6}	77.64	128.33
Foxc1	0.72	9.98×10^{-8}	155.90	257.68
Tns2	0.72	7.21×10^{-3}	162.82	269.09
H19	0.72	8.62×10^{-3}	3,777.12	6,239.84
Htr2c	0.72	1.53×10^{-7}	179.28	296.15
Gm46378	0.72	4.20×10^{-2}	14.96	24.71

Gene ID	Log2 Fold Change	p-adj	Control CPM	Rad CPM
Irf8	0.72	1.14×10^{-3}	35.89	59.25
Slc13a3	0.72	3.28×10^{-8}	142.66	235.53
Gm49883	0.72	3.10×10^{-3}	58.05	95.83
Nid1	0.72	6.35×10^{-11}	764.93	1,262.70
Gja1	0.72	1.05×10^{-8}	1,117.71	1,844.19
Incenp	0.72	3.82×10^{-7}	390.10	643.13
Mink1	0.72	5.96×10^{-9}	822.05	1,354.78
Mbd6	0.72	2.92×10^{-6}	611.45	1,006.49
Nhs	0.72	8.70×10^{-6}	170.94	281.21
Chek2	0.72	4.12×10^{-4}	38.20	62.81
Col6a1	0.72	1.45×10^{-3}	441.63	726.09
BC005561	0.72	2.14×10^{-4}	162.04	266.37
Kif18b	0.72	1.09×10^{-4}	115.84	190.41
Mdm4	0.72	2.23×10^{-5}	1,078.47	1,772.16
Pear1	0.72	7.04×10^{-7}	122.35	200.98
Ccdc3	0.72	1.78×10^{-5}	116.56	191.46
Clcn2	0.72	7.28×10^{-6}	294.88	484.27
Cdc25b	0.72	3.07×10^{-8}	276.65	454.33
Gm28151	0.72	3.53×10^{-2}	15.70	25.77
Slfn9	0.72	1.34×10^{-2}	106.55	174.98
Nat8f4	0.71	1.82×10^{-2}	27.10	44.48
Ier3	0.71	2.25×10^{-3}	34.70	56.94
Cdh5	0.71	7.42×10^{-10}	443.58	727.29
Gm28512	0.71	3.13×10^{-2}	12.79	20.97
Cox6b2	0.71	1.89×10^{-3}	32.36	53.04
Scrib	0.71	5.16×10^{-10}	1,137.96	1,864.61
Scara5	0.71	2.26×10^{-2}	27.54	45.06
Gm11266	0.71	1.82×10^{-2}	55.67	91.07
Rfx2	0.71	7.24×10^{-4}	57.29	93.72
Trpm3	0.71	2.14×10^{-6}	839.65	1,373.41
Gm32444	0.71	2.64×10^{-2}	30.67	50.16
Naa80	0.71	1.51×10^{-7}	122.61	200.48
Stap2	0.71	6.13×10^{-3}	26.59	43.47
Jak3	0.71	7.14×10^{-7}	105.50	172.43
Aoc3	0.71	4.89×10^{-2}	18.36	30.01
Elovl1	0.71	6.69×10^{-6}	102.83	168.01
Slc38a4	0.71	3.97×10^{-5}	49.50	80.85
Mmp2	0.71	3.60×10^{-4}	228.13	372.59
Gm47283	0.71	4.31×10^{-2}	1,503.32	2,454.35

Gene ID	Log2 Fold Change	p-adj	Control CPM	Rad CPM
Tmem88	0.71	2.29×10^{-3}	37.58	61.32
Gm10643	0.71	2.55×10^{-2}	18.64	30.39
Rad54b	0.70	4.72×10^{-2}	41.14	67.06
Whrn	0.70	2.85×10^{-11}	298.11	485.76
Hopx	0.70	4.00×10^{-7}	146.59	238.79
Barhl2	0.70	7.70×10^{-7}	75.37	122.66
Slc16a6	0.70	2.23×10^{-4}	120.48	196.01
Tmem181c-ps	0.70	4.85×10^{-4}	85.06	138.38
Grb10	0.70	7.79×10^{-7}	1,433.13	2,331.14
Cecr2	0.70	5.90×10^{-3}	159.71	259.76
Myh9	0.70	4.16×10^{-11}	1,110.01	1,804.47
Nfe2l2	0.70	7.45×10^{-8}	137.96	224.20
Fbn2	0.70	9.03×10^{-9}	730.73	1,187.04
Nhlh2	0.70	3.55×10^{-7}	162.45	263.86
Slitrk6	0.70	3.65×10^{-2}	17.02	27.64
Serping1	0.70	2.35×10^{-7}	99.88	162.13
Ubr4	0.70	6.54×10^{-5}	3,298.65	5,353.80
Abhd14b	0.70	3.13×10^{-3}	54.54	88.47
Plekhh1	0.70	4.98×10^{-7}	75.85	122.98
Myo9b	0.70	1.12×10^{-5}	763.12	1,237.18
H2-D1	0.70	2.01×10^{-7}	205.10	332.49
Ncf2	0.70	4.07×10^{-2}	33.58	54.44
Igdcc3	0.70	1.55×10^{-5}	101.00	163.73
Cxcl12	0.70	1.10×10^{-12}	777.82	1,260.54
Alpl	0.70	1.79×10^{-2}	96.67	156.65
Lgals3	0.70	4.03×10^{-2}	28.72	46.52
Zbtb7b	0.70	2.37×10^{-2}	44.59	72.23
Bmp3	0.70	1.73×10^{-6}	87.54	141.73
Tubgcp6	0.69	1.94×10^{-4}	492.41	797.04
Cdc25c	0.69	3.97×10^{-2}	66.41	107.45
AW822252	0.69	3.94×10^{-2}	15.15	24.50
Baz1a	0.69	3.72×10^{-3}	130.57	211.10
Ddit4l	0.69	9.62×10^{-3}	36.23	58.55
Fcgr3	0.69	7.27×10^{-7}	84.55	136.62
Mapk15	0.69	1.70×10^{-2}	53.81	86.91
Ifih1	0.69	6.77×10^{-3}	24.32	39.29
Samd9l	0.69	4.28×10^{-3}	26.77	43.23
Nfat5	0.69	7.65×10^{-4}	605.77	978.04
Hells	0.69	8.81×10^{-3}	142.36	229.79

Gene ID	Log2 Fold Change	p-adj	Control CPM	Rad CPM
Gm47113	0.69	1.87×10^{-2}	29.02	46.82
Ccdc159	0.69	1.03×10^{-2}	42.27	68.17
Ska1	0.69	3.90×10^{-3}	31.53	50.83
Svil	0.69	4.65×10^{-11}	291.39	469.70
Egfem1	0.69	2.57×10^{-2}	35.22	56.76
Aifm2	0.69	1.05×10^{-3}	80.68	129.97
Gch1	0.69	2.76×10^{-2}	15.68	25.25
Ddr2	0.69	1.56×10^{-3}	234.33	377.32
Zmym6	0.69	1.64×10^{-4}	325.75	524.35
Sfta3-ps	0.69	4.29×10^{-2}	43.90	70.64
Cdca3	0.69	4.84×10^{-4}	151.22	243.25
Stard8	0.69	1.54×10^{-3}	110.41	177.57
Ror1	0.69	1.03×10^{-3}	34.14	54.89
Ahnak	0.68	4.47×10^{-5}	583.13	937.36
Gm11127	0.68	3.13×10^{-2}	14.19	22.81
Gm43681	0.68	2.16×10^{-2}	20.64	33.16
Slc16a3	0.68	2.84×10^{-2}	69.51	111.66
Slc9a5	0.68	3.27×10^{-6}	325.57	522.82
Itpr3	0.68	1.31×10^{-5}	74.90	120.19
Pigl	0.68	2.09×10^{-3}	38.89	62.39
Adcy7	0.68	1.64×10^{-4}	108.79	174.46
Exoc3l	0.68	4.11×10^{-3}	38.36	61.50
Zfp92	0.68	1.40×10^{-2}	38.99	62.50
Prkd2	0.68	1.68×10^{-6}	108.48	173.84
Arsg	0.68	9.24×10^{-3}	65.56	105.06
Sox7	0.68	1.24×10^{-3}	64.18	102.83
Kmt2d	0.68	6.49×10^{-5}	2,347.78	3,761.44
Grin2d	0.68	3.22×10^{-9}	594.92	953.06
D330041H03Rik	0.68	2.89×10^{-3}	51.64	82.72
Irf5	0.68	3.51×10^{-2}	14.25	22.82
Tk1	0.68	4.39×10^{-4}	129.39	207.15
Gm36995	0.68	1.32×10^{-2}	59.98	96.00
Safb2	0.68	5.64×10^{-5}	767.41	1,227.56
Dot1l	0.68	8.60×10^{-7}	991.77	1,586.25
Kcp	0.68	3.05×10^{-3}	196.33	314.01
Eya1	0.68	4.54×10^{-7}	92.07	147.22
Gm21887	0.68	2.97×10^{-2}	346.45	553.64
Epha10	0.68	2.24×10^{-4}	118.59	189.48
Col6a2	0.68	4.33×10^{-2}	435.42	695.65

Gene ID	Log2 Fold Change	p-adj	Control CPM	Rad CPM
Csrnp1	0.68	1.34×10^{-2}	34.92	55.77
Igf2bp1	0.68	2.75×10^{-3}	228.83	365.39
Kifc2	0.67	5.39×10^{-5}	749.38	1,196.16
Dusp11	0.67	2.29×10^{-5}	960.37	1,532.83
Gbx1	0.67	7.92×10^{-4}	88.85	141.79
Prnd	0.67	1.75×10^{-4}	67.54	107.75
Lrrc32	0.67	2.26×10^{-6}	81.27	129.58
Dpp7	0.67	7.16×10^{-4}	99.31	158.31
Eml6	0.67	1.73×10^{-7}	232.50	370.63
Lhx5	0.67	1.07×10^{-4}	85.79	136.74
Zfp366	0.67	3.53×10^{-6}	82.53	131.54
Crb1	0.67	3.36×10^{-3}	65.61	104.57
Abca8b	0.67	2.25×10^{-6}	70.42	112.23
Notch1	0.67	1.98×10^{-7}	1,178.72	1,878.40
Sp5	0.67	7.41×10^{-3}	37.63	59.93
Itga5	0.67	2.43×10^{-4}	123.74	197.05
Fcgrt	0.67	1.20×10^{-8}	211.94	337.43
Sgo2a	0.67	3.97×10^{-5}	103.08	164.06
Atad2	0.67	1.88×10^{-3}	216.32	343.97
Hcls1	0.67	7.69×10^{-3}	31.45	49.99
Pxdn	0.67	1.64×10^{-8}	1,078.29	1,713.53
Coro7	0.67	1.11×10^{-4}	610.75	970.04
Hacd4	0.67	3.50×10^{-4}	68.46	108.67
Trim56	0.67	4.67×10^{-3}	134.04	212.72
Srpx	0.67	3.56×10^{-2}	27.68	43.91
Gpc3	0.66	3.25×10^{-8}	487.95	773.43
Evc	0.66	4.80×10^{-4}	133.41	211.43
Kif2c	0.66	4.47×10^{-4}	158.82	251.70
Epb41l4b	0.66	1.85×10^{-6}	91.46	144.94
Abcc10	0.66	6.10×10^{-5}	142.22	225.12
Tyrobp	0.66	1.88×10^{-3}	49.78	78.79
Nfkb2	0.66	2.30×10^{-4}	55.82	88.33
Sphk1	0.66	6.99×10^{-3}	53.45	84.55
Maskbp3	0.66	3.15×10^{-3}	116.57	184.39
Hbb-bs	0.66	4.22×10^{-2}	26,044.30	41,176.82
Olfml1	0.66	6.64×10^{-4}	31.90	50.41
Rfx5	0.66	4.64×10^{-5}	157.09	248.19
Rbp4	0.66	4.08×10^{-3}	25.91	40.92
Id3	0.66	3.39×10^{-11}	435.59	687.93

Gene ID	Log2 Fold Change	p-adj	Control CPM	Rad CPM
Pard3b	0.66	6.95×10^{-7}	131.69	207.85
Slc19a2	0.66	3.88×10^{-7}	102.01	160.96
Rora	0.66	2.52×10^{-4}	136.17	214.79
Prune2	0.66	8.84×10^{-3}	153.24	241.69
Tbc1d2	0.66	3.61×10^{-3}	24.60	38.79
Ccnl2	0.66	2.29×10^{-6}	1,116.93	1,761.08
Dhtkd1	0.66	1.00×10^{-3}	73.91	116.52
Unc5b	0.66	3.20×10^{-10}	310.14	488.95
Mvp	0.66	1.46×10^{-3}	65.95	103.96
Doc2g	0.66	4.05×10^{-3}	66.67	105.06
Col4a1	0.66	3.51×10^{-8}	3,395.64	5,350.75
Ccdc74a	0.65	3.87×10^{-2}	24.75	38.95
Dock2	0.65	1.21×10^{-2}	27.04	42.56
Rtl9	0.65	8.18×10^{-3}	27.79	43.72
Phlda3	0.65	7.93×10^{-6}	160.05	251.71
Vamp1	0.65	1.72×10^{-3}	97.81	153.82
Ncapd2	0.65	1.44×10^{-4}	644.95	1,014.29
Clec14a	0.65	5.39×10^{-5}	101.95	160.24
Minar1	0.65	1.79×10^{-8}	161.20	253.20
Ube2l6	0.65	1.98×10^{-4}	140.55	220.70
Zdhhc1	0.65	1.19×10^{-8}	205.84	323.19
Noxo1	0.65	3.01×10^{-2}	33.99	53.36
Ucp2	0.65	7.34×10^{-5}	388.35	609.27
Ecel1	0.65	1.31×10^{-4}	345.19	541.52
Abcc5	0.65	5.12×10^{-5}	1,200.83	1,883.70
Fkbp10	0.65	1.49×10^{-4}	233.35	365.95
Dhrs11	0.65	9.34×10^{-3}	28.14	44.09
Ptprs	0.65	8.99×10^{-6}	9,093.44	14,231.00
Cped1	0.65	3.95×10^{-3}	87.83	137.43
Ebf4	0.65	1.84×10^{-7}	217.21	339.91
Myo1f	0.65	4.50×10^{-2}	30.63	47.92
Chrna4	0.64	3.64×10^{-7}	281.65	440.37
Gigyf1	0.64	1.95×10^{-8}	759.06	1,186.73
Arhgap17	0.64	1.23×10^{-3}	155.52	243.14
Plekha7	0.64	1.50×10^{-6}	209.18	327.02
Plekhg1	0.64	7.19×10^{-5}	198.95	310.98
Lrp1	0.64	7.35×10^{-10}	5,914.64	9,245.30
Ect2	0.64	1.11×10^{-4}	177.90	278.06
Tor3a	0.64	1.51×10^{-2}	69.89	109.24

Gene ID	Log2 Fold Change	p-adj	Control CPM	Rad CPM
P3h2	0.64	2.42×10^{-2}	17.67	27.61
Cyth4	0.64	1.46×10^{-3}	63.41	99.00
Sox13	0.64	3.05×10^{-5}	162.43	253.58
Ctsc	0.64	1.95×10^{-4}	97.96	152.82
Rhbdf1	0.64	2.06×10^{-13}	204.90	319.56
Gm37254	0.64	2.21×10^{-2}	22.71	35.42
Trpm2	0.64	1.02×10^{-2}	109.75	171.15
Ppfia4	0.64	2.36×10^{-7}	783.08	1,221.01
Apoe	0.64	2.54×10^{-6}	3,946.62	6,152.78
Kif14	0.64	3.76×10^{-3}	78.85	122.92
Pml	0.64	1.34×10^{-5}	173.20	269.92
Esyt1	0.64	2.68×10^{-9}	200.14	311.84
Tert	0.64	2.80×10^{-4}	111.26	173.28
Rhpn1	0.64	1.53×10^{-5}	105.52	164.35
Irf9	0.64	2.21×10^{-6}	160.83	250.44
Itga2	0.64	1.99×10^{-2}	40.01	62.29
Gdpd2	0.64	5.62×10^{-6}	145.63	226.68
Bgn	0.64	6.04×10^{-5}	549.19	854.80
Cspg4	0.64	1.64×10^{-6}	256.51	399.03
Nfatc1	0.64	4.78×10^{-4}	58.94	91.68
H2-Q6	0.64	1.26×10^{-2}	29.76	46.24
Pld1	0.64	9.97×10^{-4}	104.43	162.17
Mybl2	0.63	3.06×10^{-4}	114.72	178.09
Aff1	0.63	3.17×10^{-4}	114.80	178.20
E2f8	0.63	6.61×10^{-4}	89.67	139.16
Efhc1	0.63	3.44×10^{-2}	18.48	28.68
Kifc5b	0.63	9.14×10^{-4}	93.28	144.69
Gpr182	0.63	2.17×10^{-2}	20.41	31.65
Tfpi	0.63	3.19×10^{-4}	104.66	162.20
Peg3	0.63	5.58×10^{-11}	2,345.72	3,634.80
Rrp1b	0.63	3.15×10^{-3}	293.64	454.89
Tmem37	0.63	4.26×10^{-2}	19.04	29.50
Shroom4	0.63	6.80×10^{-4}	102.83	159.25
Slc25a42	0.63	9.04×10^{-7}	152.43	235.99
Hlf	0.63	5.33×10^{-7}	211.96	327.87
Cks2	0.63	2.43×10^{-3}	82.56	127.63
Calcr	0.63	4.17×10^{-2}	51.82	80.08
Fam114a1	0.63	7.08×10^{-3}	98.70	152.50
Atp1a2	0.63	9.74×10^{-12}	2,968.86	4,586.65

Gene ID	Log2 Fold Change	p-adj	Control CPM	Rad CPM
Slc12a5	0.63	1.15×10^{-11}	2,030.10	3,135.52
Vps13d	0.63	6.77×10^{-6}	1,080.63	1,668.60
Stc2	0.63	6.99×10^{-3}	47.12	72.73
Dlgap5	0.63	4.24×10^{-2}	105.48	162.76
Ikzf4	0.63	6.40×10^{-7}	397.75	613.68
Map7	0.63	1.47×10^{-2}	81.36	125.48
Plch2	0.63	1.42×10^{-7}	532.70	821.61
Ntng1	0.62	6.50×10^{-4}	245.71	378.78
Steap3	0.62	7.27×10^{-3}	59.28	91.31
Thsd1	0.62	8.84×10^{-4}	106.78	164.36
5330434G04Rik	0.62	1.04×10^{-2}	53.75	82.70
C1qa	0.62	2.00×10^{-4}	180.67	277.95
Arglu1	0.62	6.34×10^{-7}	1,208.97	1,859.64
Rflna	0.62	4.42×10^{-2}	19.04	29.29
Aoc2	0.62	3.27×10^{-3}	44.72	68.76
Ptch2	0.62	5.18×10^{-4}	104.47	160.64
Mavs	0.62	5.87×10^{-4}	79.93	122.80
Crispld1	0.62	2.80×10^{-2}	45.78	70.28
Zc3hav1	0.62	1.07×10^{-2}	38.45	59.02
Chrd	0.62	1.15×10^{-4}	132.82	203.76
Col4a2	0.62	5.54×10^{-12}	2,317.48	3,555.07
Akap8l	0.62	2.45×10^{-6}	745.79	1,143.42
Stx3	0.62	1.14×10^{-3}	82.45	126.41
Plekha6	0.62	6.75×10^{-9}	721.22	1,105.69
Gypc	0.62	2.26×10^{-4}	90.33	138.48
A930024E05Rik	0.62	1.88×10^{-2}	44.05	67.53
Mis18bp1	0.62	3.96×10^{-3}	94.09	144.22
Cmtm7	0.62	4.03×10^{-3}	38.11	58.40
Ermard	0.62	8.73×10^{-4}	174.08	266.72
P4ha2	0.62	2.31×10^{-3}	91.79	140.61
Gm4739	0.62	3.79×10^{-4}	396.28	607.04
Ift172	0.62	1.96×10^{-4}	461.45	706.79
Frem1	0.62	4.00×10^{-3}	105.92	162.24
C130074G19Rik	0.62	1.72×10^{-7}	215.34	329.81
Lyl1	0.61	2.06×10^{-3}	34.91	53.45
Ube2c	0.61	2.65×10^{-4}	248.60	380.63
Cdt1	0.61	3.00×10^{-5}	125.88	192.68
Gm43951	0.61	4.29×10^{-2}	379.13	580.28
A930033H14Rik	0.61	2.87×10^{-2}	21.75	33.28

Gene ID	Log2 Fold Change	p-adj	Control CPM	Rad CPM
Zfhx3	0.61	3.63×10^{-2}	849.34	1,299.34
Ckap2l	0.61	1.03×10^{-3}	221.31	338.55
Sntb1	0.61	2.49×10^{-2}	31.83	48.68
Rassf4	0.61	4.16×10^{-3}	160.15	244.93
Mtmr11	0.61	1.30×10^{-3}	68.13	104.14
BC024139	0.61	8.04×10^{-4}	137.57	210.22
P2rx4	0.61	8.61×10^{-3}	75.91	115.98
Gm7694	0.61	3.94×10^{-2}	52.80	80.66
Shisa7	0.61	6.45×10^{-7}	1,301.76	1,987.48
Acad10	0.61	2.78×10^{-2}	24.26	37.04
Catsper2	0.61	1.72×10^{-2}	32.69	49.88
Procr	0.61	4.31×10^{-2}	17.78	27.12
Cenpi	0.61	1.61×10^{-2}	43.29	66.02
Acsf2	0.61	5.40×10^{-6}	119.89	182.79
Creg1	0.61	5.35×10^{-3}	141.68	215.85
Mrap2	0.61	3.33×10^{-2}	21.05	32.07
Fgfr3	0.61	9.39×10^{-7}	497.46	757.76
Cnn2	0.61	7.17×10^{-7}	201.38	306.68
Prr11	0.61	1.38×10^{-3}	104.53	159.18
Flt1	0.61	2.68×10^{-4}	330.74	503.61
Sfxn2	0.61	1.05×10^{-3}	162.54	247.33
2700038G22Rik	0.60	3.87×10^{-2}	29.73	45.22
Col23a1	0.60	9.74×10^{-6}	230.62	350.72
Nhsl2	0.60	1.62×10^{-2}	268.06	407.58
Krt222	0.60	9.93×10^{-3}	52.07	79.17
4930570G19Rik	0.60	2.43×10^{-2}	29.78	45.27
Col4a5	0.60	2.76×10^{-8}	268.06	407.46
Snrnp48	0.60	1.18×10^{-3}	179.05	272.15
Rock1	0.60	2.74×10^{-4}	442.68	672.46
Mbnl2	0.60	4.99×10^{-8}	272.13	413.35
Sept1	0.60	2.94×10^{-3}	46.89	71.22
Xylb	0.60	1.49×10^{-3}	50.33	76.42
Emilin1	0.60	6.38×10^{-3}	207.48	315.03
Rad51ap1	0.60	1.95×10^{-2}	59.41	90.20
Slc37a2	0.60	2.00×10^{-2}	45.75	69.44
Ildr2	0.60	1.85×10^{-6}	652.28	989.54
Impa2	0.60	1.87×10^{-2}	56.34	85.45
Cyb561d1	0.60	2.08×10^{-3}	109.14	165.51
Syde1	0.60	4.91×10^{-6}	101.65	154.13

Gene ID	Log2 Fold Change	p-adj	Control CPM	Rad CPM
Nsun7	0.60	4.48×10^{-2}	21.87	33.15
Cep95	0.60	3.79×10^{-3}	108.43	164.35
Smim26	-0.60	1.42×10^{-4}	107.81	70.99
Chchd1	-0.61	1.29×10^{-9}	446.09	292.81
Gm42778	-0.61	7.37×10^{-3}	116.19	76.26
Gm9774	-0.61	2.13×10^{-4}	197.25	129.45
Psma6	-0.61	7.68×10^{-31}	1,756.49	1,152.68
Gpr21	-0.61	6.07×10^{-6}	523.59	343.51
Rgs6	-0.61	1.95×10^{-5}	664.51	435.50
Maf1	-0.61	2.84×10^{-8}	1,980.28	1,297.78
Rnf182	-0.61	3.80×10^{-8}	869.37	569.70
Gm6658	-0.61	1.95×10^{-2}	29.50	19.29
Gm14391	-0.61	3.62×10^{-3}	182.79	119.46
Prxl2b	-0.61	7.04×10^{-4}	326.79	213.50
Dio2	-0.62	8.33×10^{-4}	172.25	112.32
Gm14292	-0.62	7.11×10^{-4}	66.81	43.56
Rpl9-ps6	-0.62	1.01×10^{-6}	667.52	435.08
BC024063	-0.62	1.61×10^{-2}	83.40	54.35
Gm14150	-0.62	3.13×10^{-2}	32.16	20.95
Chrna7	-0.62	1.95×10^{-2}	97.15	63.28
Gm20390	-0.62	2.75×10^{-3}	1,320.06	859.83
Tbcc	-0.62	3.35×10^{-7}	355.62	231.58
Sla	-0.62	1.43×10^{-6}	1,223.16	796.37
Mkln1	-0.62	9.81×10^{-4}	1,493.37	971.16
Lix1	-0.62	5.25×10^{-8}	350.17	227.71
S1pr5	-0.62	9.40×10^{-3}	49.88	32.44
Rpl10a-ps1	-0.62	3.54×10^{-11}	2,584.29	1,678.89
Mc4r	-0.62	4.70×10^{-4}	91.58	59.48
Arsk	-0.62	2.38×10^{-2}	91.79	59.60
Id2	-0.63	1.54×10^{-13}	2,121.49	1,372.21
Mien1	-0.63	5.63×10^{-9}	890.61	575.28
Zfp558	-0.63	3.22×10^{-4}	343.09	221.60
Cdc26	-0.63	1.35×10^{-6}	758.74	490.05
Mcub	-0.63	2.49×10^{-2}	43.70	28.22
Ssbp2	-0.63	6.21×10^{-21}	2,517.88	1,625.37
Tmem200c	-0.63	4.43×10^{-7}	173.49	111.89
BC002059	-0.63	2.86×10^{-3}	103.69	66.84
Adora2a	-0.63	1.02×10^{-4}	387.29	249.52
Nip7	-0.63	6.69×10^{-9}	684.98	441.20

Gene ID	Log2 Fold Change	p-adj	Control CPM	Rad CPM
Snx7	-0.64	6.88×10^{-6}	227.14	146.00
Baiap2l2	-0.64	3.72×10^{-2}	53.33	34.27
Fbxo32	-0.64	4.94×10^{-6}	197.73	127.04
H3f3aos	-0.64	1.83×10^{-2}	48.71	31.29
Tmsb15l	-0.64	2.08×10^{-2}	59.19	38.00
Cops9	-0.64	1.12×10^{-5}	975.19	624.88
Slc25a53	-0.64	4.56×10^{-4}	221.76	141.98
3010001F23Rik	-0.65	2.07×10^{-2}	34.65	22.15
Gm17383	-0.65	3.21×10^{-9}	2,797.56	1,784.69
Trappc4	-0.65	8.39×10^{-12}	1,351.58	862.20
Tradd	-0.65	1.19×10^{-6}	126.18	80.48
Acvr1	-0.65	3.44×10^{-14}	737.42	470.21
Rps14	-0.65	1.09×10^{-12}	6,661.08	4,245.78
Mrpl47	-0.65	1.65×10^{-4}	282.99	180.35
Eloc	-0.65	4.47×10^{-5}	1,909.92	1,216.43
Atp5e	-0.65	2.52×10^{-7}	1,616.71	1,029.66
Fst	-0.65	6.02×10^{-3}	109.95	70.02
Nts	-0.65	6.25×10^{-4}	518.63	330.14
Gm1673	-0.65	3.56×10^{-7}	1,041.44	662.55
Acot9	-0.66	1.95×10^{-6}	386.83	245.47
Gm13436	-0.66	2.99×10^{-2}	21.28	13.50
Cct6a	-0.66	2.97×10^{-6}	5,028.74	3,184.86
Pcdh20	-0.67	2.32×10^{-5}	218.74	137.92
2900009J06Rik	-0.67	1.57×10^{-2}	31.42	19.74
Gm13910	-0.67	3.92×10^{-2}	54.34	34.12
Kcnu1	-0.67	7.57×10^{-3}	68.89	43.25
Cldn3	-0.68	5.43×10^{-4}	73.45	45.98
Has2	-0.68	1.48×10^{-3}	47.84	29.93
Lipg	-0.68	1.12×10^{-7}	178.03	111.39
C230057M02Rik	-0.68	1.33×10^{-11}	380.22	237.51
Supt4a	-0.68	8.30×10^{-14}	634.71	396.37
Dnajb3	-0.68	5.65×10^{-3}	61.82	38.60
Rarb	-0.68	6.08×10^{-8}	448.11	279.66
Gm45716	-0.68	1.51×10^{-8}	204.68	127.69
Mindy4	-0.68	3.16×10^{-3}	190.11	118.55
Hspe1	-0.69	5.71×10^{-9}	991.48	614.93
Htr3a	-0.69	1.28×10^{-6}	122.75	76.07
Tcea1-ps1	-0.69	2.03×10^{-2}	44.82	27.73
Gm10240	-0.70	1.69×10^{-2}	2,023.48	1,248.93

Gene ID	Log2 Fold Change	p-adj	Control CPM	Rad CPM
Mrps23	-0.70	1.88×10^{-4}	740.32	456.57
Fbxl12os	-0.70	1.64×10^{-2}	62.41	38.45
Ldb2	-0.71	1.45×10^{-8}	1,004.33	615.70
Gm40841	-0.71	9.47×10^{-4}	65.17	39.88
Gm17750	-0.73	2.18×10^{-6}	306.59	185.30
Rpl18-ps2	-0.73	2.87×10^{-2}	17.70	10.70
Zfp560	-0.73	2.38×10^{-5}	246.12	148.16
Gm9954	-0.73	3.93×10^{-3}	104.93	63.09
Tac2	-0.74	3.98×10^{-2}	23.18	13.92
Mef2c	-0.74	4.00×10^{-10}	5,284.24	3,172.81
Car4	-0.74	6.55×10^{-3}	39.27	23.56
Trmt9b	-0.74	4.36×10^{-5}	111.30	66.76
Gpr6	-0.74	1.84×10^{-2}	39.03	23.38
Prokr2	-0.74	5.79×10^{-4}	292.12	174.84
Zfp759	-0.74	3.47×10^{-2}	133.88	80.13
Syt6	-0.75	8.69×10^{-9}	821.35	489.74
Sfrp5	-0.75	1.90×10^{-3}	47.48	28.28
Syt4	-0.75	6.37×10^{-9}	5,093.22	3,030.19
Gm2423	-0.75	3.71×10^{-5}	815.26	484.48
Rpl9-ps4	-0.75	2.99×10^{-8}	231.96	137.66
Mpped1	-0.75	2.73×10^{-14}	8,968.96	5,318.45
Mrpl23	-0.76	1.76×10^{-3}	69.04	40.91
Cck	-0.76	2.52×10^{-5}	328.44	194.61
Gucy1b1	-0.76	2.45×10^{-12}	1,769.93	1,043.92
Gm10778	-0.77	7.69×10^{-3}	128.10	75.34
Saysd1	-0.77	1.86×10^{-6}	148.38	87.07
Fgfbp3	-0.77	7.09×10^{-10}	664.28	389.11
Bhlhe22	-0.77	5.08×10^{-14}	3,035.72	1,775.70
2900079G21Rik	-0.78	1.86×10^{-6}	204.45	119.44
Gm8206	-0.78	1.90×10^{-2}	46.78	27.30
Zbtb18	-0.78	7.06×10^{-15}	9,376.16	5,467.22
Cd24a	-0.78	1.86×10^{-10}	11,701.15	6,822.69
Msra	-0.78	3.38×10^{-17}	665.15	386.98
Gpr34	-0.78	2.89×10^{-2}	24.06	13.98
Lmo4	-0.78	1.19×10^{-36}	3,181.77	1,846.81
9130024F11Rik	-0.79	3.85×10^{-9}	675.65	391.82
2610307P16Rik	-0.79	1.63×10^{-2}	66.44	38.39
Tmem74	-0.79	1.40×10^{-10}	406.90	234.58
Neurod6	-0.80	5.00×10^{-13}	5,033.74	2,900.63

Gene ID	Log2 Fold Change	p-adj	Control CPM	Rad CPM
Aox4	-0.80	1.08×10^{-10}	230.99	132.88
Ntf3	-0.80	1.19×10^{-4}	85.43	49.07
Hapln3	-0.81	1.68×10^{-4}	109.22	62.27
Gm17455	-0.81	3.85×10^{-2}	17.28	9.85
Snrpe	-0.81	1.11×10^{-4}	855.89	486.62
Atp5l	-0.82	6.63×10^{-19}	534.54	303.71
Gm16464	-0.82	9.59×10^{-3}	31.43	17.81
Zfp708	-0.83	1.88×10^{-5}	75.67	42.54
Gm1818	-0.83	2.50×10^{-4}	73.99	41.55
Vmp1	-0.84	3.60×10^{-7}	1,025.51	574.73
Satb2	-0.84	3.37×10^{-10}	3,767.56	2,108.66
Hgf	-0.84	3.03×10^{-3}	35.34	19.70
Hdc	-0.84	1.42×10^{-2}	60.53	33.72
Gpx4-ps2	-0.85	5.47×10^{-5}	86.29	47.99
Gm11578	-0.85	5.37×10^{-3}	31.97	17.78
Gm10421	-0.85	1.49×10^{-2}	18.26	10.10
Gm14325	-0.87	1.57×10^{-2}	88.27	48.45
Gm12966	-0.87	2.42×10^{-2}	36.70	20.06
Lpl	-0.87	1.36×10^{-6}	776.97	424.08
Sema7a	-0.88	9.04×10^{-29}	1,411.63	767.63
1600023N17Rik	-0.89	2.54×10^{-2}	14.53	7.84
Pdlim3	-0.89	4.97×10^{-2}	32.43	17.48
Glt28d2	-0.90	1.75×10^{-2}	28.46	15.26
Gm14270	-0.90	3.88×10^{-2}	17.58	9.42
Ikzf1	-0.90	1.02×10^{-4}	233.23	124.62
Syndig1	-0.91	2.68×10^{-6}	252.19	134.42
Gm45169	-0.91	3.97×10^{-4}	46.91	25.01
Sec61g	-0.92	2.09×10^{-6}	127.95	67.58
Dok5	-0.92	8.35×10^{-18}	995.24	525.63
Npy	-0.93	2.08×10^{-12}	801.09	421.40
Gm4081	-0.94	9.03×10^{-3}	18.83	9.78
Efcab7	-0.95	2.92×10^{-3}	75.05	38.72
Kcnj2	-0.96	1.30×10^{-10}	173.55	89.15
Crym	-0.97	3.09×10^{-6}	642.94	327.85
9930014A18Rik	-0.97	3.53×10^{-11}	171.22	87.12
Gdf9	-0.98	2.57×10^{-3}	32.18	16.37
Ccbe1	-0.99	5.48×10^{-13}	310.29	156.63
Usp43	-1.01	2.57×10^{-9}	251.36	125.13
Pou3f1	-1.01	2.58×10^{-55}	1,170.05	581.01

Gene ID	Log2 Fold Change	p-adj	Control CPM	Rad CPM
Ermn	-1.01	2.88×10^{-3}	24.42	12.10
Nlrp6	-1.01	8.35×10^{-3}	29.17	14.45
Ly6g6e	-1.02	1.59×10^{-3}	28.43	13.99
Gm3531	-1.03	8.62×10^{-8}	77.20	37.89
Pomc	-1.04	1.41×10^{-2}	38.66	18.86
Gm2999	-1.04	2.97×10^{-3}	23.33	11.37
Kcns3	-1.05	2.94×10^{-2}	17.10	8.28
Gm26881	-1.05	2.16×10^{-4}	27.53	13.31
Gm4544	-1.05	1.89×10^{-2}	14.58	7.04
Gm40847	-1.07	1.01×10^{-2}	14.29	6.81
Nr4a3	-1.11	1.46×10^{-21}	970.77	449.36
1110002E22Rik	-1.11	1.53×10^{-2}	46.48	21.50
Cacng1	-1.14	3.68×10^{-3}	30.72	13.98
Gucy1a1	-1.15	3.34×10^{-21}	2,964.42	1,335.70
Dsc3	-1.15	4.11×10^{-3}	37.60	16.89
Gm49708	-1.17	1.68×10^{-5}	51.72	22.99
Rpl10-ps1	-1.19	4.43×10^{-2}	686.57	300.68
Pabpc1l	-1.19	6.95×10^{-3}	15.01	6.56
Lhcgr	-1.20	1.34×10^{-2}	40.29	17.53
Casq1	-1.21	1.71×10^{-3}	76.32	33.05
Gm17102	-1.26	4.08×10^{-3}	17.35	7.27
Ighm	-1.29	1.08×10^{-4}	29.46	12.09
Gm47210	-1.31	1.74×10^{-2}	18.84	7.61
1700014D04Rik	-1.31	6.19×10^{-3}	14.45	5.83
Gm21972	-1.33	1.55×10^{-2}	27.24	10.87
9130017K11Rik	-1.34	5.93×10^{-4}	15.63	6.16
Gm8430	-1.38	3.99×10^{-3}	228.94	87.70
Crlf1	-1.39	6.43×10^{-11}	194.14	73.85
Hspb3	-1.51	4.12×10^{-4}	19.70	6.91
A830009L08Rik	-1.54	5.21×10^{-4}	15.73	5.40
Ccno	-1.58	3.89×10^{-6}	19.67	6.57
C1qtnf3	-1.64	1.60×10^{-2}	20.13	6.47
Grp	-1.66	7.31×10^{-9}	95.46	30.26
Acvr1c	-1.70	8.95×10^{-4}	22.24	6.85
Crybb1	-2.04	3.07×10^{-11}	44.16	10.72
Cma1	-2.07	5.52×10^{-4}	85.22	20.25
P2ry12	-2.08	4.19×10^{-30}	87.69	20.71
Tpsab1	-2.10	2.80×10^{-4}	22.67	5.29
Cpa3	-2.35	3.25×10^{-7}	67.59	13.29

Gene ID	Log2 Fold Change	p-adj	Control CPM	Rad CPM
Ccr5	-2.43	1.59×10^{-4}	30.80	5.72
Tpsb2	-2.58	1.30×10^{-10}	78.68	13.14
Msx3	-3.18	7.20×10^{-12}	33.48	3.71
Gm10718	-3.22	3.59×10^{-2}	38.68	4.16
Gm10719	-3.41	1.05×10^{-2}	33.08	3.12

Supplementary Table S3: Differentially expressed genes in the female telencephalon following gestational radiation exposure.

All 1242 genes differentially expressed between control and gestationally irradiated female telencephalon (n = 8 per sex per treatment group, one fetus per sex per dam; sham: 8 dams, irradiated: 9 dams), identified by DESeq2 as log2 fold change ≥ 0.6 or ≤ - 0.6 with a Benjamini-Hochberg false discovery rate (FDR) adjusted p-value (p-adj) <0.05. Genes are ranked by descending log2 fold change, where positive values indicate increased expression relative to control (1037 upregulated, 205 downregulated). Control CPM and Rad CPM are mean normalized counts per million for each group.

Gene ID	Log2 Fold Change	p-adj	Control CPM	Rad CPM
Gpnmb	5.26	2.31×10^{-19}	8.92	342.54
Gm11639	4.55	1.43×10^{-2}	0.89	20.88
H2-Q5	4.34	9.74×10^{-4}	1.37	27.69
Il1rn	4.20	6.17×10^{-4}	1.30	23.78
Atp6v0d2	3.68	9.58×10^{-6}	1.75	22.45
Ifit1	3.65	2.51×10^{-12}	2.39	30.06
Lilrb4a	3.16	3.26×10^{-8}	2.32	20.76
Lyz2	2.92	9.06×10^{-21}	97.23	736.30
Siglec1	2.79	3.37×10^{-16}	10.48	72.27
Spp1	2.68	4.38×10^{-6}	164.21	1,052.10
Il33	2.46	6.77×10^{-6}	3.69	20.32
Msr1	2.43	3.79×10^{-10}	7.49	40.43
Itgax	2.43	1.74×10^{-6}	9.16	49.29
Hmox1	2.41	1.65×10^{-7}	84.15	448.30
Ms4a7	2.34	1.93×10^{-9}	5.70	28.79
Clec4n	2.26	3.46×10^{-5}	5.28	25.37
4930594M22Rik	2.22	1.25×10^{-5}	3.57	16.68
Clec7a	2.15	1.36×10^{-3}	5.00	22.17
Isg15	2.11	1.24×10^{-7}	10.01	43.19
C4b	2.06	1.70×10^{-5}	18.34	76.41
Sp100	2.04	1.76×10^{-4}	5.51	22.64
Ifi27l2a	2.00	2.23×10^{-6}	4.68	18.74
Ifit3	1.93	9.07×10^{-9}	11.31	43.01
Gbp10	1.92	1.53×10^{-2}	4.24	16.09
Oasl2	1.90	1.58×10^{-9}	19.73	73.90
Ms4a6c	1.90	4.22×10^{-9}	19.07	71.40
Cybb	1.89	8.10×10^{-5}	9.74	36.18
Tmem106a	1.89	4.09×10^{-8}	13.57	50.28
Sp110	1.87	3.16×10^{-4}	5.24	19.14
Dnm3os	1.85	3.72×10^{-2}	4.50	16.17
Lgals3bp	1.84	8.70×10^{-15}	50.50	180.82

Gene ID	Log2 Fold Change	p-adj	Control CPM	Rad CPM
Gbp2b	1.82	6.24×10^{-5}	6.41	22.65
Plau	1.81	6.02×10^{-12}	36.02	125.92
Folr2	1.79	9.56×10^{-4}	6.73	23.22
Ifi44l	1.77	1.30×10^{-5}	5.68	19.37
Oas1a	1.72	3.43×10^{-5}	8.88	29.21
Slc22a2	1.71	7.10×10^{-8}	22.20	72.60
Olfr1372-ps1	1.68	7.15×10^{-3}	5.93	19.00
Slc15a3	1.65	2.44×10^{-5}	16.45	51.79
Lrrc25	1.62	2.44×10^{-4}	8.68	26.68
C1ra	1.62	3.63×10^{-4}	7.67	23.57
Clec10a	1.61	3.06×10^{-3}	5.47	16.68
Vwf	1.61	1.38×10^{-8}	187.42	570.82
Pqlc3	1.60	3.29×10^{-6}	12.91	39.20
Ifi207	1.59	1.16×10^{-3}	8.13	24.49
Gm44675	1.58	2.69×10^{-2}	6.12	18.32
Mx2	1.58	2.04×10^{-4}	5.19	15.53
Ifi202b	1.57	1.42×10^{-2}	6.01	17.81
AB124611	1.56	1.95×10^{-4}	5.82	17.20
Lyve1	1.56	8.52×10^{-10}	70.69	208.84
Icam1	1.56	2.04×10^{-8}	31.40	92.47
Tnfrsf10b	1.55	3.25×10^{-8}	14.34	41.98
Stab1	1.55	9.16×10^{-19}	308.96	902.52
Cd38	1.55	1.10×10^{-4}	12.93	37.74
Eda2r	1.54	2.92×10^{-6}	19.03	55.47
Irf7	1.54	1.40×10^{-6}	20.96	60.88
Bst2	1.53	2.07×10^{-14}	47.58	137.75
Gcnt4	1.53	1.11×10^{-4}	7.45	21.50
Fzd10os	1.53	2.74×10^{-3}	6.85	19.71
AI662270	1.52	2.63×10^{-3}	6.45	18.49
F5	1.52	1.73×10^{-3}	5.69	16.26
Il10ra	1.51	6.27×10^{-7}	11.98	34.23
Ifi44	1.51	4.72×10^{-5}	18.01	51.37
Myo7a	1.51	1.87×10^{-7}	34.47	98.28
Gm44562	1.51	2.34×10^{-2}	5.46	15.57
Ugt1a7c	1.51	7.51×10^{-5}	12.15	34.63
Vnn1	1.51	1.65×10^{-5}	9.35	26.58
Gm10134	1.51	1.82×10^{-6}	10.68	30.33
Trpm5	1.50	8.93×10^{-6}	7.42	21.03
Xaf1	1.50	5.84×10^{-3}	7.70	21.81

Gene ID	Log2 Fold Change	p-adj	Control CPM	Rad CPM
Obscn	1.50	1.51×10^{-7}	61.43	173.42
Ms4a6b	1.48	4.18×10^{-5}	16.95	47.34
Gm14176	1.48	3.98×10^{-2}	6.91	19.29
Reln	1.48	2.50×10^{-26}	2,688.24	7,494.64
Tlr13	1.47	6.01×10^{-5}	12.92	35.86
P2ry6	1.47	3.13×10^{-8}	20.36	56.28
Ccdc88b	1.46	1.29×10^{-2}	12.78	35.26
Fcgr2b	1.46	1.21×10^{-8}	22.47	61.97
Sned1	1.45	2.65×10^{-7}	167.18	457.33
Cdk18	1.45	1.03×10^{-4}	22.22	60.50
Cd300a	1.44	2.09×10^{-9}	15.80	42.96
Eps8l2	1.44	3.93×10^{-3}	7.91	21.47
Gja5	1.44	1.03×10^{-4}	15.11	40.86
Lhx1os	1.43	8.55×10^{-4}	6.31	17.05
Rbm47	1.43	1.32×10^{-5}	26.79	72.09
F13a1	1.43	5.70×10^{-15}	211.09	566.95
Trem2	1.41	6.99×10^{-8}	34.06	90.82
Gm42715	1.41	4.06×10^{-2}	217.39	579.10
A230103L15Rik	1.41	9.19×10^{-4}	21.24	56.40
Itgb2	1.40	2.78×10^{-3}	10.13	26.69
Myo1f	1.39	7.53×10^{-7}	22.55	59.12
Cp	1.39	1.69×10^{-19}	95.40	249.60
Cyp1b1	1.38	2.12×10^{-14}	92.64	241.06
Itgal	1.38	2.99×10^{-4}	13.13	34.13
Proca1	1.38	1.71×10^{-4}	9.39	24.37
Dlx6os2	1.37	4.12×10^{-3}	6.82	17.63
Dhx58	1.37	8.97×10^{-5}	9.07	23.42
Gm49027	1.37	1.07×10^{-4}	26.98	69.63
Snhg11	1.37	2.10×10^{-19}	862.91	2,223.79
Myof	1.36	4.99×10^{-6}	55.84	143.79
C2	1.36	3.50×10^{-3}	17.49	44.87
Ifitm3	1.35	1.24×10^{-18}	94.92	242.24
D630023F18Rik	1.35	7.52×10^{-8}	23.54	59.88
Slc4a5	1.34	1.30×10^{-7}	45.70	115.98
Rgs11	1.34	7.43×10^{-5}	40.67	103.08
Gab3	1.34	4.83×10^{-3}	6.04	15.31
Apobec1	1.33	4.33×10^{-7}	21.70	54.59
A2ml1	1.33	1.23×10^{-6}	18.34	46.13
Insrr	1.33	6.06×10^{-4}	12.01	30.21

Gene ID	Log2 Fold Change	p-adj	Control CPM	Rad CPM
Abi3bp	1.33	1.91×10^{-3}	11.18	28.03
Ccdc158	1.32	2.18×10^{-3}	7.59	18.95
Fat2	1.32	4.51×10^{-4}	11.13	27.77
Trp73	1.32	6.09×10^{-27}	202.48	504.45
Gm43302	1.31	7.60×10^{-3}	17.60	43.74
Frrs1	1.31	1.13×10^{-6}	27.73	68.90
Hpse	1.31	1.97×10^{-3}	10.57	26.24
Igsf6	1.31	3.35×10^{-3}	8.12	20.11
Foxd2	1.30	5.55×10^{-4}	11.35	28.01
Slc7a11	1.30	7.28×10^{-13}	225.34	555.69
Tifab	1.30	2.51×10^{-7}	19.46	47.98
Tnfaip2	1.30	7.48×10^{-3}	10.92	26.83
Prodh	1.30	6.68×10^{-4}	26.61	65.38
H2-Q7	1.30	2.06×10^{-2}	6.27	15.42
Plekha4	1.29	6.16×10^{-3}	10.85	26.62
Rtp4	1.29	1.83×10^{-5}	24.22	59.30
Cfap70	1.29	1.38×10^{-2}	9.87	24.06
C3ar1	1.28	4.43×10^{-7}	28.14	68.55
Dok2	1.28	3.32×10^{-3}	12.03	29.28
Usp18	1.28	3.63×10^{-4}	14.64	35.63
Col6a6	1.28	1.54×10^{-2}	16.86	41.01
Gm37090	1.28	5.29×10^{-3}	7.25	17.57
Bnc2	1.28	6.56×10^{-3}	18.96	45.92
Ms4a6d	1.27	5.15×10^{-4}	24.96	60.40
Col24a1	1.27	4.62×10^{-3}	13.33	32.11
Gbp4	1.26	1.68×10^{-6}	28.93	69.35
Ptprc	1.25	2.99×10^{-6}	15.44	36.82
C5ar1	1.25	1.97×10^{-2}	7.50	17.89
Rnf213	1.25	2.87×10^{-7}	111.01	263.74
Gbgt1	1.25	1.45×10^{-2}	6.09	14.47
Svep1	1.25	1.42×10^{-8}	50.62	120.03
Slc37a2	1.25	2.34×10^{-5}	39.15	92.81
Aebp1	1.24	9.45×10^{-12}	253.80	600.90
Cdh23	1.24	6.49×10^{-3}	34.86	82.24
Cd84	1.24	6.14×10^{-4}	23.05	54.33
Slc13a4	1.23	9.88×10^{-19}	112.10	263.73
Trim21	1.23	1.19×10^{-4}	16.70	39.30
H2-K1	1.23	4.06×10^{-10}	177.90	418.31
Gbp7	1.23	6.83×10^{-6}	33.28	78.24

Gene ID	Log2 Fold Change	p-adj	Control CPM	Rad CPM
Sidt1	1.23	4.40×10^{-22}	141.13	331.42
Apod	1.23	4.73×10^{-12}	461.95	1,083.34
Gabre	1.23	2.79×10^{-3}	11.45	26.80
Fmod	1.23	2.33×10^{-9}	273.42	639.14
Cpne7	1.22	5.59×10^{-27}	126.30	294.71
9930111J21Rik2	1.22	8.64×10^{-4}	10.62	24.76
Fcna	1.22	3.04×10^{-6}	34.61	80.60
Parp9	1.22	1.39×10^{-11}	35.64	82.86
A2m	1.22	2.82×10^{-16}	148.37	344.52
Psmb8	1.21	1.04×10^{-4}	16.42	38.05
Kcnh6	1.21	7.13×10^{-6}	18.34	42.36
Mpeg1	1.21	2.23×10^{-12}	144.09	332.42
Parp14	1.20	5.14×10^{-7}	23.99	55.23
Gbp2	1.20	1.09×10^{-3}	12.73	29.31
Zan	1.20	3.07×10^{-2}	6.46	14.83
Ppp1r3b	1.20	5.82×10^{-11}	66.52	152.58
Gbp9	1.20	3.71×10^{-4}	22.14	50.74
Ripor3	1.20	2.24×10^{-2}	7.16	16.39
Gjb2	1.19	2.27×10^{-9}	82.84	189.65
Ptgdr	1.19	3.54×10^{-3}	14.01	32.07
Irgm2	1.19	3.25×10^{-7}	16.56	37.82
Rmst	1.19	8.69×10^{-4}	25.72	58.73
Cd274	1.19	3.20×10^{-5}	20.02	45.64
Foxc1	1.19	1.61×10^{-15}	119.35	271.90
Rasal1	1.19	1.11×10^{-8}	179.02	407.80
Lrp8os2	1.19	2.64×10^{-2}	8.96	20.39
Sfta3-ps	1.19	2.51×10^{-4}	31.53	71.71
A330023F24Rik	1.18	6.72×10^{-4}	37.83	85.98
Acss3	1.18	3.04×10^{-3}	11.27	25.57
H2-T24	1.18	5.36×10^{-3}	11.52	26.11
Baiap3	1.18	1.15×10^{-9}	157.66	357.39
Dlec1	1.18	2.72×10^{-3}	16.69	37.79
Gjb6	1.18	2.61×10^{-6}	115.06	260.47
Slfn2	1.17	1.85×10^{-3}	14.57	32.88
Ankrd35	1.17	9.95×10^{-14}	59.47	133.95
Nid2	1.17	8.26×10^{-23}	448.63	1,010.22
Greb1	1.17	6.36×10^{-3}	17.28	38.91
Ccr1	1.17	1.10×10^{-3}	8.57	19.27
Ifitm1	1.17	4.20×10^{-9}	35.42	79.62

Gene ID	Log2 Fold Change	p-adj	Control CPM	Rad CPM
Irgm1	1.17	5.99×10^{-5}	30.51	68.57
Zfp469	1.17	1.76×10^{-7}	48.89	109.74
Scn5a	1.16	1.62×10^{-16}	137.18	307.55
Klhdc7a	1.16	1.52×10^{-18}	64.77	145.17
Spag17	1.16	3.39×10^{-2}	7.77	17.41
Cntnap1	1.16	8.79×10^{-12}	60.50	135.58
8430422M14Rik	1.16	2.32×10^{-3}	7.19	16.11
Ncf2	1.16	1.17×10^{-3}	29.68	66.47
4930447C04Rik	1.16	4.90×10^{-2}	11.44	25.60
Erap1	1.16	1.07×10^{-3}	59.53	133.24
Oxtr	1.16	6.61×10^{-16}	64.43	144.05
Asah2	1.16	3.02×10^{-4}	20.82	46.42
Ahnak2	1.16	1.52×10^{-7}	48.22	107.45
Plk5	1.16	3.66×10^{-6}	42.43	94.54
Tmem140	1.15	9.65×10^{-3}	14.63	32.50
Piezo1	1.15	3.65×10^{-14}	333.64	740.87
Gpr50	1.15	2.12×10^{-2}	10.42	23.10
Rac2	1.15	4.49×10^{-4}	31.39	69.56
Gm8909	1.15	2.43×10^{-4}	12.23	27.10
Mfrp	1.15	2.75×10^{-8}	42.84	94.84
Rsad2	1.14	4.88×10^{-5}	42.56	93.79
Iigp1	1.14	3.61×10^{-2}	6.79	14.96
Sesn2	1.13	6.15×10^{-21}	97.02	213.03
Slc16a11	1.13	3.63×10^{-4}	28.86	63.22
Mertk	1.13	2.13×10^{-5}	33.02	72.33
Cdh3	1.13	1.11×10^{-4}	31.32	68.48
Mrap2	1.13	7.01×10^{-4}	14.75	32.18
Tnfrsf11a	1.13	2.46×10^{-5}	19.08	41.63
BC067074	1.12	2.43×10^{-8}	40.25	87.73
Hspg2	1.12	2.69×10^{-5}	515.00	1,120.41
Mroh7	1.12	2.77×10^{-2}	13.19	28.68
Psmb9	1.12	9.04×10^{-3}	8.01	17.37
Pik3ap1	1.11	1.03×10^{-6}	31.56	68.14
Aldh1a1	1.11	1.49×10^{-8}	39.75	85.79
Tm4sf5	1.11	2.62×10^{-2}	7.32	15.79
Adamts12	1.11	3.09×10^{-8}	133.91	288.78
Asb2	1.10	5.19×10^{-3}	16.24	34.87
Col4a3	1.10	3.42×10^{-2}	8.90	19.10
Arhgef16	1.10	2.56×10^{-3}	15.11	32.37

Gene ID	Log2 Fold Change	p-adj	Control CPM	Rad CPM
Serpinb9	1.10	1.36×10^{-4}	21.16	45.31
Uba7	1.10	3.16×10^{-2}	19.56	41.85
Gm16485	1.10	1.51×10^{-2}	10.48	22.42
Col27a1	1.10	8.39×10^{-12}	57.56	123.04
Cd68	1.09	1.25×10^{-5}	55.91	119.42
Car12	1.09	5.78×10^{-25}	197.89	422.63
Wnk4	1.09	7.57×10^{-5}	59.68	127.44
Cfh	1.09	2.45×10^{-9}	264.15	563.47
Gm49083	1.09	1.03×10^{-3}	10.81	23.06
Atoh8	1.09	1.10×10^{-5}	23.04	49.13
Ebf2	1.09	1.96×10^{-7}	38.17	81.26
Cpz	1.09	2.23×10^{-2}	15.63	33.26
Tgfbi	1.09	1.20×10^{-5}	53.67	114.18
Tlr2	1.09	3.37×10^{-4}	19.16	40.73
Parp10	1.09	1.10×10^{-3}	27.75	58.89
Exoc3l4	1.08	1.90×10^{-2}	8.99	19.06
Hapln4	1.08	1.95×10^{-2}	10.22	21.65
Rasa4	1.08	1.59×10^{-6}	71.31	150.95
Hck	1.08	4.92×10^{-5}	19.06	40.32
Tnfrsf23	1.08	1.70×10^{-2}	9.89	20.90
Plekhd1	1.08	4.00×10^{-2}	9.57	20.22
Tmem63a	1.08	3.11×10^{-5}	29.86	63.05
Mrc1	1.08	8.70×10^{-10}	232.44	490.54
Ikbke	1.08	3.79×10^{-2}	13.86	29.24
Colec12	1.08	8.26×10^{-16}	398.22	839.62
Aox3	1.08	2.92×10^{-2}	17.60	37.09
Trpv4	1.07	2.38×10^{-3}	21.26	44.74
Slc16a8	1.07	1.13×10^{-2}	7.88	16.57
Sh3d21	1.07	4.74×10^{-3}	10.83	22.77
Eif2ak2	1.07	6.65×10^{-14}	63.33	132.92
Mab21l1	1.07	1.02×10^{-15}	126.10	264.53
Gm27199	1.07	1.46×10^{-2}	7.68	16.09
Cldn11	1.07	2.47×10^{-7}	311.22	651.52
Arhgap9	1.06	1.30×10^{-2}	8.06	16.86
Lgals9	1.06	5.33×10^{-11}	89.70	187.59
Arhgap22	1.06	2.63×10^{-3}	17.58	36.75
Foxc2	1.06	2.24×10^{-4}	61.27	127.93
Tll2	1.06	1.60×10^{-3}	12.20	25.43
Sult1a1	1.06	1.06×10^{-8}	57.04	118.88

Gene ID	Log2 Fold Change	p-adj	Control CPM	Rad CPM
Trabd2b	1.06	1.09×10^{-8}	48.49	100.98
Gbx1	1.06	5.80×10^{-7}	71.81	149.53
Bhlhe41	1.06	1.54×10^{-7}	42.25	87.95
Uncx	1.06	1.42×10^{-4}	26.68	55.50
Bicc1	1.06	1.75×10^{-10}	133.56	277.87
Tcirg1	1.06	3.98×10^{-6}	96.77	201.26
Themis2	1.06	9.58×10^{-3}	8.12	16.89
Srpx2	1.06	2.01×10^{-2}	12.18	25.33
Nbeal2	1.06	3.31×10^{-5}	141.58	294.26
Arap1	1.05	3.65×10^{-11}	184.79	383.91
Dab2	1.05	8.59×10^{-11}	280.12	580.64
Ptpn14	1.05	9.64×10^{-15}	92.36	191.40
Spta1	1.05	8.68×10^{-3}	12.36	25.60
Gpx3	1.05	5.32×10^{-15}	149.01	308.55
Igf2os	1.05	3.79×10^{-3}	20.51	42.34
Tbx18	1.04	1.63×10^{-7}	79.41	163.84
Osr1	1.04	3.34×10^{-6}	60.29	124.35
Ifi203	1.04	6.43×10^{-3}	17.30	35.67
Ctss	1.04	7.63×10^{-11}	85.83	176.95
9430064I24Rik	1.04	4.48×10^{-3}	14.80	30.48
Gm10499	1.04	7.32×10^{-5}	31.45	64.63
Col18a1	1.04	1.34×10^{-13}	388.86	798.64
Tns2	1.04	1.56×10^{-7}	134.25	275.67
Fn1	1.04	1.08×10^{-10}	1,325.58	2,719.48
Lhx1	1.03	1.96×10^{-5}	34.02	69.71
Bmp6	1.03	8.70×10^{-15}	259.86	531.99
Ranbp3l	1.03	5.10×10^{-5}	75.80	154.42
Itih2	1.03	1.68×10^{-5}	66.21	134.90
Epor	1.03	2.39×10^{-2}	8.62	17.55
Gm38198	1.03	2.89×10^{-2}	6.89	14.02
Piezo2	1.02	3.58×10^{-6}	44.83	91.09
Adamts9	1.02	9.38×10^{-16}	164.29	333.37
Kcnc4	1.02	9.02×10^{-18}	191.50	388.48
Mmp19	1.02	3.59×10^{-2}	8.83	17.91
B2m	1.02	3.26×10^{-10}	170.32	344.56
Odf3b	1.01	3.76×10^{-2}	7.29	14.71
Atp10b	1.01	5.54×10^{-3}	10.04	20.24
Phlda3	1.01	5.65×10^{-12}	147.27	296.05
Lepr	1.01	4.28×10^{-8}	99.29	199.60

Gene ID	Log2 Fold Change	p-adj	Control CPM	Rad CPM
Mrgprf	1.01	1.98×10^{-5}	48.64	97.76
Adrb2	1.01	1.12×10^{-2}	11.04	22.20
Ifi27	1.01	1.16×10^{-5}	66.61	133.85
Slc6a7	1.01	2.98×10^{-24}	289.01	580.14
Ptgds	1.00	2.69×10^{-9}	1,458.31	2,925.83
Parp12	1.00	6.22×10^{-10}	62.17	124.48
Slc22a6	1.00	6.96×10^{-10}	169.81	339.95
5031434O11Rik	1.00	1.14×10^{-3}	30.70	61.32
Lama3	1.00	5.74×10^{-6}	39.13	78.05
Barhl2	1.00	1.90×10^{-9}	62.32	124.23
Gucy2e	0.99	1.32×10^{-2}	8.55	17.03
Slc41a3	0.99	3.51×10^{-6}	34.10	67.93
Epha10	0.99	4.59×10^{-12}	89.15	177.60
Brca2	0.99	8.03×10^{-8}	120.69	240.35
Hspb8	0.99	8.67×10^{-9}	83.36	165.98
Tmem132e	0.99	6.86×10^{-9}	142.00	282.37
Trim30a	0.99	1.50×10^{-2}	12.30	24.45
Fezf1	0.99	3.68×10^{-6}	80.76	160.35
Gm38317	0.99	7.95×10^{-3}	13.59	26.98
Ugt1a6b	0.99	2.49×10^{-2}	22.52	44.69
Gm26672	0.99	1.72×10^{-3}	11.95	23.71
Apoe	0.99	9.21×10^{-23}	3,742.31	7,425.71
Xpnpep2	0.99	9.73×10^{-3}	12.98	25.75
Ace	0.99	6.50×10^{-23}	245.97	487.75
Neat1	0.99	1.72×10^{-3}	83.94	166.27
Ecel1	0.99	6.97×10^{-13}	279.71	553.97
Aldh1a2	0.98	1.64×10^{-12}	218.63	432.25
Timp1	0.98	3.98×10^{-2}	11.39	22.51
Flt3l	0.98	2.67×10^{-2}	8.46	16.72
Adcy7	0.98	4.89×10^{-9}	90.89	179.60
Tap2	0.98	1.29×10^{-3}	33.43	66.03
Lrrk2	0.98	3.88×10^{-3}	71.05	140.34
Fignl2	0.98	4.94×10^{-4}	17.17	33.89
Ddx58	0.98	1.05×10^{-4}	55.48	109.41
Iqca	0.98	3.06×10^{-2}	7.04	13.88
Nxnl2	0.98	2.89×10^{-3}	13.81	27.21
Mvp	0.98	4.17×10^{-5}	48.18	94.72
Cfap100	0.97	2.57×10^{-2}	25.10	49.31
H2-D1	0.97	1.03×10^{-13}	206.80	405.93

Gene ID	Log2 Fold Change	p-adj	Control CPM	Rad CPM
Sfmbt2	0.97	3.71×10^{-6}	43.53	85.37
Cacna2d2	0.97	2.35×10^{-9}	767.69	1,504.22
Lgals3	0.97	2.39×10^{-2}	34.63	67.84
Mapkapk3	0.97	2.82×10^{-3}	17.09	33.44
Prdm6	0.97	4.36×10^{-2}	28.75	56.25
Il4ra	0.97	4.34×10^{-7}	56.72	110.85
Ctsc	0.97	1.51×10^{-8}	104.47	204.12
Nupr1	0.96	4.38×10^{-3}	24.83	48.47
Igf2	0.96	2.80×10^{-9}	4,664.45	9,103.10
Slc17a8	0.96	6.43×10^{-3}	18.41	35.91
Prr32	0.96	2.39×10^{-4}	25.17	48.95
Syt17	0.96	1.58×10^{-15}	138.57	269.26
Plxnb3	0.96	1.43×10^{-2}	9.78	19.00
Jade2	0.96	9.02×10^{-8}	41.12	79.78
Lox	0.95	1.21×10^{-7}	293.89	569.46
Cd14	0.95	2.08×10^{-2}	13.19	25.53
Slc25a37	0.95	4.69×10^{-5}	138.80	268.50
C1qa	0.95	1.68×10^{-8}	170.18	329.18
Gm49593	0.95	8.78×10^{-3}	10.71	20.73
Irf5	0.95	9.63×10^{-3}	13.65	26.40
Cdh17	0.95	4.69×10^{-2}	8.57	16.56
Gm6145	0.95	3.09×10^{-3}	25.61	49.47
Col8a2	0.95	1.09×10^{-7}	192.71	372.10
Tnfrsf13b	0.95	3.87×10^{-2}	10.59	20.44
Adra2a	0.95	4.12×10^{-25}	222.43	429.22
Tap1	0.95	1.82×10^{-4}	55.28	106.63
Mst1r	0.95	7.59×10^{-3}	31.45	60.65
Prune2	0.94	1.68×10^{-6}	141.66	272.52
B130024G19Rik	0.94	2.17×10^{-2}	17.46	33.56
Slc2a12	0.94	8.97×10^{-14}	125.34	240.64
Npr1	0.94	3.00×10^{-6}	48.04	92.12
Adam12	0.94	1.29×10^{-8}	206.93	396.61
Calcr	0.94	5.29×10^{-3}	42.98	82.36
Plekha7	0.94	1.19×10^{-9}	174.55	334.24
Rinl	0.94	7.23×10^{-3}	14.64	28.02
Arhgap19	0.94	3.21×10^{-4}	25.97	49.68
Ankle1	0.94	4.21×10^{-3}	23.79	45.50
Aifm3	0.93	9.75×10^{-7}	108.90	208.13
Igtp	0.93	1.53×10^{-3}	15.28	29.19

Gene ID	Log2 Fold Change	p-adj	Control CPM	Rad CPM
Tep1	0.93	1.42×10^{-2}	61.99	118.41
Optc	0.93	8.35×10^{-4}	21.33	40.75
Hif3a	0.93	2.21×10^{-5}	195.68	373.17
S100b	0.93	5.13×10^{-3}	14.84	28.31
2900040C04Rik	0.93	1.42×10^{-3}	15.88	30.25
Slc12a5	0.93	2.21×10^{-48}	1,648.55	3,141.11
Bdkrb2	0.93	9.41×10^{-4}	15.11	28.77
Tmem252	0.93	3.44×10^{-4}	23.90	45.52
Gm43719	0.93	2.57×10^{-3}	14.49	27.59
Slco1a4	0.93	1.65×10^{-2}	28.44	54.13
Krt8	0.93	3.16×10^{-3}	22.64	43.07
Col16a1	0.93	9.85×10^{-5}	204.74	389.47
Ddn	0.93	1.21×10^{-19}	427.69	813.44
Gm20559	0.93	1.45×10^{-3}	14.28	27.15
Zfp853	0.93	1.90×10^{-5}	36.74	69.86
Clic6	0.93	3.19×10^{-16}	255.65	486.06
P2rx7	0.93	1.80×10^{-3}	59.65	113.39
Smpdl3b	0.92	2.56×10^{-2}	9.54	18.10
Llgl2	0.92	1.11×10^{-2}	15.17	28.76
Slc6a13	0.92	7.53×10^{-7}	259.06	490.68
Gm42979	0.92	4.00×10^{-2}	20.74	39.26
Creg2	0.92	1.45×10^{-8}	124.33	235.01
Egr4	0.92	1.58×10^{-4}	25.20	47.56
Pld6	0.91	3.68×10^{-6}	49.69	93.67
Crispld2	0.91	3.15×10^{-5}	45.39	85.50
Gm38124	0.91	1.69×10^{-2}	12.44	23.43
Kcnj13	0.91	2.95×10^{-3}	24.54	46.20
Alx3	0.91	1.93×10^{-2}	25.74	48.45
Emilin1	0.91	3.14×10^{-5}	171.74	323.22
Kl	0.91	2.96×10^{-5}	26.20	49.25
Adgrg6	0.91	1.49×10^{-4}	41.05	77.18
Pon3	0.91	3.92×10^{-3}	17.11	32.16
Rxfp1	0.91	1.64×10^{-2}	23.49	44.14
Kif19a	0.91	1.29×10^{-2}	18.21	34.21
Gpr183	0.91	3.31×10^{-2}	10.78	20.22
Cdkn1a	0.91	1.31×10^{-32}	441.44	827.40
Kndc1	0.91	1.99×10^{-16}	532.73	998.03
Gpr61	0.91	2.69×10^{-8}	73.06	136.86
Slc6a20a	0.91	1.68×10^{-6}	100.41	188.06

Gene ID	Log2 Fold Change	p-adj	Control CPM	Rad CPM
Bmp5	0.90	3.43×10^{-6}	50.59	94.64
Tmem72	0.90	6.42×10^{-3}	69.64	130.06
Samd9l	0.90	6.48×10^{-4}	27.50	51.31
Gm28151	0.90	1.23×10^{-2}	11.66	21.75
Snai2	0.90	3.38×10^{-2}	12.32	22.97
Thsd7b	0.90	1.20×10^{-10}	204.43	380.99
Car5b	0.90	2.73×10^{-2}	17.91	33.37
Tmem91	0.90	9.64×10^{-7}	58.61	109.12
Ifi35	0.90	7.58×10^{-4}	19.02	35.41
Arhgap30	0.90	7.35×10^{-4}	25.27	47.02
AU020206	0.90	2.30×10^{-5}	51.40	95.58
Lynx1	0.89	2.75×10^{-17}	141.26	262.15
Gm28050	0.89	1.18×10^{-2}	12.28	22.78
Lrp1b	0.89	8.73×10^{-5}	179.13	332.27
Tmem151a	0.89	3.83×10^{-31}	425.08	787.85
Lgi3	0.89	1.89×10^{-3}	32.97	61.11
Cyp4v3	0.89	8.86×10^{-4}	28.67	53.13
Ggt5	0.89	1.72×10^{-4}	30.47	56.47
Angptl2	0.89	7.23×10^{-6}	171.89	318.35
Muc1	0.89	2.58×10^{-2}	14.59	27.02
Cox6b2	0.89	1.28×10^{-4}	31.42	58.17
Fcgr3	0.88	2.02×10^{-4}	76.12	140.57
Htr2c	0.88	5.00×10^{-12}	181.14	334.34
Fxyd7	0.88	5.77×10^{-25}	328.75	606.24
Sst	0.88	1.34×10^{-14}	1,513.83	2,791.04
Lamb3	0.88	4.05×10^{-3}	24.72	45.54
Adamts16	0.88	3.56×10^{-4}	22.60	41.63
Steap3	0.88	1.30×10^{-8}	59.15	108.94
Parp4	0.88	3.46×10^{-5}	36.29	66.82
Lbp	0.88	4.14×10^{-4}	46.42	85.45
Fcer1g	0.88	1.93×10^{-4}	43.28	79.66
Samd3	0.88	2.97×10^{-2}	38.15	70.20
Dok3	0.88	6.22×10^{-4}	30.83	56.72
H2-Q4	0.88	1.76×10^{-6}	55.37	101.81
Slc5a5	0.88	9.57×10^{-5}	21.97	40.38
Plscr2	0.88	3.46×10^{-2}	8.35	15.35
Tgfbr3	0.88	1.23×10^{-5}	109.35	200.83
Ccdc187	0.88	1.82×10^{-4}	31.60	58.01
Lhx5	0.88	2.30×10^{-7}	79.70	146.26

Gene ID	Log2 Fold Change	p-adj	Control CPM	Rad CPM
Alx4	0.87	9.99×10^{-4}	84.44	154.82
L3mbtl1	0.87	7.71×10^{-5}	41.28	75.64
Serpind1	0.87	6.58×10^{-4}	29.64	54.26
Plec	0.87	6.42×10^{-14}	475.40	870.12
Dcxr	0.87	1.52×10^{-5}	38.72	70.83
Dsp	0.87	4.21×10^{-2}	108.30	198.01
Itih5	0.87	1.93×10^{-9}	285.88	522.56
Rin3	0.87	2.07×10^{-8}	67.98	124.13
Irf8	0.87	8.46×10^{-5}	35.88	65.52
9330162G02Rik	0.87	6.47×10^{-4}	176.30	321.81
Car10	0.87	9.15×10^{-6}	229.34	418.36
Wdr86	0.87	2.45×10^{-9}	85.95	156.62
Icam5	0.86	4.67×10^{-12}	142.17	258.88
Calhm2	0.86	1.89×10^{-2}	12.83	23.36
Gm41662	0.86	2.84×10^{-2}	9.14	16.62
Akr1b10	0.86	3.60×10^{-6}	34.55	62.84
Inpp5j	0.86	7.97×10^{-9}	117.46	213.64
Fcgr1	0.86	3.34×10^{-4}	25.16	45.75
Ssc5d	0.86	2.00×10^{-3}	30.07	54.68
Gm5089	0.86	1.47×10^{-2}	19.99	36.31
Thbd	0.86	6.41×10^{-3}	203.07	368.59
Gpr101	0.86	4.68×10^{-2}	12.66	22.98
Zc3hav1	0.86	8.52×10^{-4}	38.04	68.97
Pkp2	0.86	9.46×10^{-3}	13.73	24.90
Ntrk1	0.86	1.45×10^{-2}	23.15	41.96
Glp1r	0.86	8.72×10^{-3}	12.43	22.51
Dnaic1	0.86	3.70×10^{-2}	12.95	23.46
Tnfaip8l2	0.86	1.45×10^{-2}	8.86	16.03
Rab32	0.86	1.21×10^{-2}	21.95	39.73
Shh	0.86	6.27×10^{-4}	25.74	46.56
Zap70	0.85	1.56×10^{-2}	18.77	33.94
Gm37249	0.85	1.14×10^{-2}	15.65	28.29
Itga8	0.85	2.36×10^{-4}	47.21	85.34
Plekhf1	0.85	4.88×10^{-2}	11.48	20.76
Htr7	0.85	1.25×10^{-5}	103.16	186.41
Tfr2	0.85	4.20×10^{-3}	30.91	55.82
Plek2	0.85	2.67×10^{-2}	10.14	18.29
Tril	0.85	5.04×10^{-17}	340.72	613.85
Amz1	0.85	7.94×10^{-10}	99.86	179.88

Gene ID	Log2 Fold Change	p-adj	Control CPM	Rad CPM
4930539E08Rik	0.85	2.34×10^{-3}	38.42	69.21
Trpc6	0.85	1.89×10^{-2}	10.62	19.13
Slc4a10	0.85	2.92×10^{-6}	394.56	709.05
Dtx3l	0.85	5.23×10^{-4}	38.20	68.63
Renbp	0.85	1.25×10^{-4}	43.30	77.80
Cspg4	0.85	4.07×10^{-9}	207.91	373.53
Pla2g3	0.85	1.59×10^{-8}	72.96	131.06
Klc3	0.84	1.02×10^{-2}	16.70	30.00
Insm2	0.84	5.47×10^{-3}	21.00	37.72
Gm11127	0.84	1.57×10^{-2}	14.51	26.05
Foxd1	0.84	1.37×10^{-3}	68.98	123.82
Adamtsl4	0.84	1.88×10^{-2}	17.63	31.65
Tnfrsf1b	0.84	6.30×10^{-6}	55.15	98.89
Fut4	0.84	7.86×10^{-3}	15.57	27.90
Spi1	0.84	4.68×10^{-4}	31.55	56.55
Ryr2	0.84	5.98×10^{-6}	291.73	522.68
Car14	0.84	3.70×10^{-7}	83.15	148.97
Nphs2	0.84	2.72×10^{-2}	11.93	21.35
Gpc3	0.84	6.64×10^{-8}	473.17	845.99
Prima1	0.84	3.84×10^{-2}	11.39	20.36
Fign	0.84	6.74×10^{-4}	118.66	211.98
Chrdl1	0.84	4.14×10^{-3}	41.58	74.28
Col23a1	0.84	4.34×10^{-9}	192.79	344.33
Wnt4	0.84	9.84×10^{-11}	160.70	286.93
Evc	0.83	5.53×10^{-9}	115.26	205.52
Ndnf	0.83	1.44×10^{-5}	449.94	802.10
A4galt	0.83	4.89×10^{-4}	26.40	47.04
Irak4	0.83	2.86×10^{-2}	13.54	24.11
Serhl	0.83	1.37×10^{-4}	40.41	71.89
Ephx1	0.83	2.78×10^{-8}	90.51	160.95
Atp6ap1l	0.83	7.11×10^{-5}	41.62	73.96
Glra3	0.83	1.29×10^{-2}	31.71	56.30
Cpeb1	0.83	2.31×10^{-2}	30.86	54.79
Rgs14	0.83	2.81×10^{-4}	25.98	46.12
Il17rc	0.83	5.07×10^{-3}	23.91	42.45
Susd2	0.83	7.38×10^{-3}	26.80	47.56
Kcng2	0.83	2.16×10^{-2}	29.93	53.10
Svil	0.83	3.87×10^{-9}	261.27	463.39
Tgm2	0.83	4.51×10^{-7}	150.03	266.04

Gene ID	Log2 Fold Change	p-adj	Control CPM	Rad CPM
Sash3	0.83	1.56×10^{-2}	14.22	25.19
AI606473	0.82	7.35×10^{-4}	58.55	103.49
Aox1	0.82	9.67×10^{-3}	13.10	23.16
Bmp7	0.82	1.23×10^{-11}	341.09	602.86
Anpep	0.82	6.68×10^{-4}	124.85	220.57
Igsf10	0.82	9.85×10^{-4}	148.36	261.99
1700001L05Rik	0.82	1.38×10^{-3}	28.02	49.45
Card10	0.82	1.07×10^{-10}	80.74	142.46
Nhsl2	0.82	1.76×10^{-4}	212.69	375.25
Dock6	0.82	3.42×10^{-4}	299.09	527.69
Mov10	0.82	1.41×10^{-6}	93.55	165.05
Gm19410	0.82	1.28×10^{-2}	27.89	49.18
Ncf1	0.82	1.10×10^{-3}	39.73	70.02
Ninj1	0.82	2.12×10^{-14}	114.56	201.90
C78859	0.82	1.76×10^{-4}	50.92	89.73
Gm6158	0.82	4.18×10^{-2}	27.56	48.55
Rassf5	0.82	1.06×10^{-5}	40.58	71.47
Tfap2b	0.82	1.08×10^{-2}	48.55	85.48
Slc24a4	0.82	9.04×10^{-4}	29.78	52.41
Gm44168	0.82	1.51×10^{-2}	26.17	46.05
Lrp1	0.81	3.52×10^{-10}	4,487.26	7,890.14
Syne1	0.81	2.19×10^{-8}	592.75	1,041.90
Sp5	0.81	3.96×10^{-6}	33.77	59.35
Tfpi	0.81	1.62×10^{-10}	99.67	175.15
Ror1	0.81	5.27×10^{-3}	31.23	54.86
Fhit	0.81	1.25×10^{-2}	22.38	39.28
Pitpnm3	0.81	5.47×10^{-6}	196.02	344.08
Tex15	0.81	2.90×10^{-5}	61.57	108.08
Pld2	0.81	1.33×10^{-9}	191.29	335.69
Moxd1	0.81	2.04×10^{-5}	45.62	80.03
Npbwr1	0.81	3.29×10^{-2}	11.67	20.47
P2rx5	0.81	2.54×10^{-2}	9.32	16.34
Rhpn1	0.81	1.70×10^{-5}	102.16	179.00
Adamts15	0.81	7.02×10^{-11}	171.04	299.61
Kank2	0.81	4.20×10^{-9}	179.19	313.84
Grin2d	0.81	3.86×10^{-16}	480.82	841.80
Megf6	0.81	1.49×10^{-7}	263.09	460.42
Gabrq	0.81	1.81×10^{-3}	35.39	61.86
Resp18	0.80	1.26×10^{-5}	165.38	288.70

Gene ID	Log2 Fold Change	p-adj	Control CPM	Rad CPM
Gm37607	0.80	3.30×10^{-2}	10.01	17.46
Adgra2	0.80	2.12×10^{-9}	305.92	533.49
Krt18	0.80	1.82×10^{-4}	32.43	56.51
Grem2	0.80	4.22×10^{-8}	118.37	206.26
Minar1	0.80	1.42×10^{-7}	144.85	252.36
Ier3	0.80	3.26×10^{-4}	37.59	65.47
C1ql2	0.80	3.84×10^{-3}	27.22	47.40
Sardh	0.80	3.62×10^{-3}	129.48	225.45
Tgfb1	0.80	7.04×10^{-6}	82.64	143.87
Tacr1	0.80	3.60×10^{-5}	73.72	128.28
Adra1b	0.80	6.53×10^{-6}	61.76	107.46
C1qc	0.80	1.41×10^{-6}	168.81	293.45
Fgfbp1	0.80	2.98×10^{-4}	79.27	137.76
Rcsd1	0.80	5.04×10^{-6}	115.00	199.77
Nlrc3	0.80	7.63×10^{-3}	18.84	32.70
Iqcd	0.80	1.90×10^{-2}	10.45	18.13
Erbb3	0.79	3.82×10^{-2}	33.51	58.09
Mxra8	0.79	5.08×10^{-5}	155.45	269.41
Acad10	0.79	4.10×10^{-4}	23.78	41.21
Slc16a3	0.79	1.96×10^{-5}	70.83	122.65
Lacc1	0.79	7.66×10^{-3}	17.25	29.82
Dnase2a	0.79	1.98×10^{-4}	37.64	64.97
Slc2a4rg-ps	0.79	2.68×10^{-3}	50.35	86.92
Chrna4	0.79	1.88×10^{-8}	237.92	410.58
C1qb	0.79	1.17×10^{-8}	161.53	278.69
Nkapl	0.79	4.12×10^{-2}	9.33	16.09
Lama4	0.79	2.03×10^{-6}	408.97	704.82
Nckap1l	0.78	3.93×10^{-4}	61.52	105.96
Smad9	0.78	1.46×10^{-2}	25.93	44.65
Camk2d	0.78	2.91×10^{-15}	708.58	1,219.16
Skor1	0.78	2.40×10^{-2}	19.27	33.14
Enpp1	0.78	4.03×10^{-4}	47.84	82.23
Hsd3b7	0.78	1.88×10^{-2}	48.03	82.55
Lamc3	0.78	4.09×10^{-6}	126.74	217.83
Mc5r	0.78	2.69×10^{-5}	38.72	66.53
Gpc5	0.78	3.99×10^{-3}	39.88	68.47
Kcnk9	0.78	2.70×10^{-9}	228.10	391.63
Igsf1	0.78	3.74×10^{-4}	112.39	192.77
Sema3b	0.78	4.93×10^{-5}	56.71	97.22

Gene ID	Log2 Fold Change	p-adj	Control CPM	Rad CPM
Tmprss7	0.78	3.79×10^{-2}	15.66	26.84
Tpcn2	0.78	8.68×10^{-3}	25.74	44.10
Unc93b1	0.78	5.69×10^{-5}	89.89	153.92
Gm6548	0.78	1.10×10^{-2}	14.88	25.48
Tmc8	0.78	4.56×10^{-2}	13.29	22.74
Gfra2	0.77	3.54×10^{-11}	242.37	414.59
Tnfrsf8	0.77	3.21×10^{-2}	16.00	27.36
Dnase1l1	0.77	8.55×10^{-3}	17.52	29.96
Islr	0.77	1.95×10^{-6}	352.79	603.10
Prlr	0.77	4.80×10^{-2}	15.40	26.32
Kcnt1	0.77	2.10×10^{-12}	327.11	558.87
Col4a2	0.77	3.17×10^{-10}	1,989.14	3,396.73
Cmbl	0.77	2.66×10^{-5}	67.61	115.44
Fes	0.77	9.28×10^{-5}	46.22	78.87
Fkbp10	0.77	3.43×10^{-8}	216.00	368.28
Gadd45b	0.77	5.03×10^{-4}	41.84	71.32
Pamr1	0.77	2.06×10^{-3}	36.04	61.42
Man2b2	0.77	8.69×10^{-6}	169.11	288.20
Agtrap	0.77	2.34×10^{-6}	99.23	169.02
Ache	0.77	8.00×10^{-9}	312.70	532.45
Hmgcs2	0.77	1.85×10^{-5}	300.55	511.25
Hacd4	0.77	6.01×10^{-5}	66.57	113.13
9530082P21Rik	0.76	2.22×10^{-2}	16.43	27.91
Rrbp1	0.76	2.84×10^{-7}	566.72	962.26
E330034L11Rik	0.76	2.23×10^{-3}	25.11	42.54
Abca9	0.76	5.67×10^{-5}	183.79	311.38
Abcc3	0.76	2.80×10^{-2}	29.36	49.74
Npffr1	0.76	5.78×10^{-3}	23.24	39.36
Unc5b	0.76	4.95×10^{-9}	275.28	466.02
Ugt1a6a	0.76	3.78×10^{-3}	35.62	60.28
Fam20a	0.76	1.62×10^{-4}	28.64	48.45
Tfcp2l1	0.76	3.94×10^{-2}	41.27	69.72
Adm	0.76	8.01×10^{-3}	34.68	58.57
Ifitm10	0.76	7.06×10^{-4}	33.09	55.88
Lime1	0.75	4.53×10^{-3}	169.37	285.71
Grik1	0.75	1.31×10^{-20}	309.36	521.52
Bin2	0.75	3.97×10^{-2}	13.74	23.17
Gm20045	0.75	2.86×10^{-3}	33.81	56.99
Arhgap27	0.75	8.18×10^{-4}	63.44	106.92

Gene ID	Log2 Fold Change	p-adj	Control CPM	Rad CPM
Htr1d	0.75	4.78×10^{-2}	23.99	40.38
Hap1	0.75	1.97×10^{-10}	2,459.66	4,134.15
Pros1	0.75	8.64×10^{-7}	98.79	165.93
Slc7a3	0.75	3.02×10^{-4}	78.85	132.32
Spsb2	0.75	1.46×10^{-4}	34.51	57.92
Wdr63	0.75	3.05×10^{-2}	21.38	35.87
Sulf1	0.74	6.23×10^{-10}	390.28	654.00
Sec14l2	0.74	5.27×10^{-4}	55.77	93.39
Hlf	0.74	3.72×10^{-6}	210.87	353.12
Ssh3	0.74	5.24×10^{-6}	144.86	242.42
Panx2	0.74	2.49×10^{-8}	318.33	532.63
Pir	0.74	1.94×10^{-2}	16.58	27.74
Cdh22	0.74	2.76×10^{-11}	225.78	377.17
Rasd2	0.74	4.26×10^{-9}	76.35	127.53
Cdc42ep5	0.74	3.07×10^{-2}	19.13	31.95
Paox	0.74	9.41×10^{-4}	99.78	166.64
Smad6	0.74	2.24×10^{-4}	48.38	80.78
Pf4	0.74	6.61×10^{-3}	51.09	85.30
Gm14827	0.74	2.21×10^{-2}	18.44	30.78
Lrrc32	0.74	4.47×10^{-5}	75.83	126.59
Hspa1a	0.74	3.35×10^{-5}	59.31	98.97
Kcne2	0.74	3.05×10^{-6}	101.67	169.58
Trank1	0.74	1.60×10^{-8}	1,042.99	1,739.64
Tor3a	0.74	2.51×10^{-4}	73.23	122.04
Vamp5	0.74	5.84×10^{-5}	44.88	74.78
Grm1	0.74	1.91×10^{-10}	324.01	539.82
Adam15	0.74	1.58×10^{-9}	483.23	804.37
Helz2	0.74	4.94×10^{-3}	46.56	77.50
Rsph10b	0.74	2.67×10^{-2}	26.11	43.46
Angptl4	0.73	1.38×10^{-3}	137.06	227.97
Tbx2	0.73	2.86×10^{-5}	50.52	83.98
Matn2	0.73	7.50×10^{-4}	104.77	174.15
Slc12a7	0.73	7.04×10^{-11}	238.13	395.65
Gm10419	0.73	1.61×10^{-3}	58.65	97.43
Arsg	0.73	1.22×10^{-5}	73.91	122.76
Fam184b	0.73	6.12×10^{-8}	161.49	268.21
Slc1a6	0.73	6.11×10^{-8}	104.53	173.60
Ttr	0.73	1.59×10^{-10}	10,474.62	17,392.65
Gbp3	0.73	7.23×10^{-3}	32.03	53.18

Gene ID	Log2 Fold Change	p-adj	Control CPM	Rad CPM
Cyp26b1	0.73	4.30×10^{-13}	413.01	685.65
Pdzph1	0.73	1.88×10^{-3}	32.93	54.66
Eya2	0.73	7.11×10^{-6}	103.83	172.35
Gda	0.73	8.12×10^{-7}	626.46	1,039.52
Pappa	0.73	9.64×10^{-3}	25.78	42.77
Syt2	0.73	1.58×10^{-2}	56.62	93.87
Fcgrt	0.73	1.68×10^{-6}	223.48	370.38
Rbm20	0.73	1.94×10^{-5}	78.06	129.36
Gypc	0.73	7.68×10^{-7}	87.11	144.23
Tekt5	0.73	3.62×10^{-4}	41.56	68.81
Axin2	0.73	2.89×10^{-9}	416.79	689.46
Ccn2	0.73	1.10×10^{-6}	125.57	207.70
Nell1	0.73	3.84×10^{-13}	349.15	577.35
Ackr1	0.73	8.13×10^{-7}	195.74	323.57
Slc18a3	0.72	2.06×10^{-2}	28.43	46.96
Lhx8	0.72	7.03×10^{-4}	181.61	299.97
Tmem191c	0.72	9.64×10^{-6}	164.22	271.14
Calml4	0.72	1.03×10^{-2}	36.66	60.52
Pdgfd	0.72	3.75×10^{-3}	23.13	38.16
Hebp1	0.72	2.25×10^{-4}	73.85	121.81
Ifi30	0.72	1.28×10^{-4}	98.64	162.68
Myh9	0.72	2.93×10^{-11}	976.75	1,610.63
Naprt	0.72	9.30×10^{-3}	29.37	48.43
Hic1	0.72	1.80×10^{-6}	110.86	182.77
Cdkn3	0.72	3.75×10^{-2}	14.64	24.13
Mmp2	0.72	3.09×10^{-4}	212.56	349.67
Mgll	0.72	1.34×10^{-9}	475.32	781.81
Wnt6	0.72	2.42×10^{-3}	52.59	86.50
Phospho1	0.72	2.23×10^{-2}	53.89	88.58
Ogn	0.72	5.74×10^{-4}	218.45	358.99
Cfap126	0.72	5.02×10^{-4}	33.91	55.70
Irf1	0.72	4.06×10^{-3}	29.92	49.13
Pla2g7	0.72	1.37×10^{-4}	282.17	463.26
Tonsl	0.72	8.53×10^{-4}	134.24	220.37
Gpld1	0.71	3.92×10^{-3}	57.80	94.87
Capg	0.71	9.41×10^{-3}	39.65	65.02
Hnmt	0.71	7.03×10^{-4}	54.17	88.84
Drc7	0.71	1.12×10^{-2}	23.49	38.50
B3gnt9	0.71	1.21×10^{-3}	61.84	101.32

Gene ID	Log2 Fold Change	p-adj	Control CPM	Rad CPM
Rhbdf2	0.71	1.24×10^{-2}	25.71	42.12
Abca4	0.71	6.36×10^{-3}	40.06	65.62
Gas6	0.71	6.90×10^{-13}	667.94	1,094.00
Cd33	0.71	1.87×10^{-2}	24.90	40.77
Fam43a	0.71	4.11×10^{-10}	183.56	300.51
Zfp92	0.71	9.41×10^{-4}	31.96	52.32
Plcg2	0.71	1.03×10^{-2}	26.67	43.66
Dnah7b	0.71	4.68×10^{-3}	71.48	116.96
Dera	0.71	6.49×10^{-5}	44.93	73.50
Slc30a3	0.71	1.96×10^{-2}	19.08	31.21
Sntb1	0.71	4.25×10^{-3}	36.04	58.93
Vsir	0.71	2.64×10^{-3}	93.92	153.56
Samd11	0.71	2.61×10^{-2}	14.40	23.54
Myh14	0.71	1.04×10^{-2}	92.40	151.03
Igfbp5	0.71	2.41×10^{-9}	2,700.23	4,413.42
Cgnl1	0.71	1.04×10^{-4}	224.75	367.32
Ctxn2	0.71	5.23×10^{-3}	39.84	65.11
Tspan17	0.71	1.31×10^{-3}	154.97	253.21
Ehd4	0.71	8.23×10^{-8}	128.21	209.47
Dcn	0.71	1.36×10^{-4}	427.79	698.46
Cpxm1	0.71	3.90×10^{-8}	231.92	378.63
Fgd6	0.71	2.38×10^{-4}	110.40	180.24
Aspm	0.71	1.15×10^{-4}	237.30	387.22
Runx1	0.71	2.20×10^{-2}	17.37	28.34
Elfn1	0.71	2.39×10^{-8}	354.01	577.15
Gli1	0.70	6.72×10^{-5}	108.76	177.29
Nqo2	0.70	3.26×10^{-3}	62.72	102.24
Pear1	0.70	1.25×10^{-4}	118.94	193.83
Laptm5	0.70	9.40×10^{-6}	260.38	424.17
Nid1	0.70	9.92×10^{-10}	770.81	1,255.47
Amy1	0.70	1.85×10^{-2}	102.22	166.47
Sgsh	0.70	2.78×10^{-4}	64.35	104.75
Zfp941	0.70	3.58×10^{-6}	143.06	232.75
Slc7a10	0.70	2.56×10^{-4}	65.40	106.39
Lin28b	0.70	1.29×10^{-3}	69.80	113.51
Il6ra	0.70	5.25×10^{-3}	26.74	43.49
Mmrn2	0.70	6.27×10^{-6}	63.04	102.47
Irs4	0.70	6.17×10^{-3}	89.06	144.76
Mgmt	0.70	3.24×10^{-2}	15.56	25.29

Gene ID	Log2 Fold Change	p-adj	Control CPM	Rad CPM
Lnx1	0.70	2.33×10^{-6}	87.51	142.17
Fmnl1	0.70	2.32×10^{-10}	234.93	381.60
Bcan	0.70	1.13×10^{-9}	1,033.44	1,678.48
Alas2	0.70	1.74×10^{-2}	606.33	984.53
Gm50322	0.70	4.67×10^{-2}	14.02	22.75
Bmp4	0.70	6.88×10^{-3}	47.05	76.35
Cxcl12	0.70	6.51×10^{-10}	767.04	1,244.60
Alk	0.70	8.86×10^{-5}	86.24	139.94
Lrtm2	0.70	8.18×10^{-11}	245.05	397.28
Ildr2	0.70	9.82×10^{-10}	582.35	943.60
Gusb	0.70	1.01×10^{-7}	190.98	309.44
Egfl7	0.70	3.66×10^{-8}	200.06	324.12
Amn	0.70	1.16×10^{-3}	71.75	116.23
Rtkn	0.69	3.65×10^{-4}	86.19	139.50
Mapk15	0.69	5.02×10^{-3}	47.52	76.89
Col4a1	0.69	7.97×10^{-9}	3,129.57	5,063.55
Hpcal1	0.69	3.43×10^{-8}	395.26	639.44
Edn3	0.69	2.08×10^{-3}	72.72	117.63
Ebf3	0.69	5.53×10^{-5}	122.37	197.91
Kifc2	0.69	2.00×10^{-15}	687.77	1,112.12
Slc17a7	0.69	8.96×10^{-16}	1,399.76	2,261.27
Olfml1	0.69	1.57×10^{-3}	36.58	59.08
Ovgp1	0.69	2.23×10^{-2}	29.61	47.80
Slc38a4	0.69	6.03×10^{-5}	57.05	92.09
Magel2	0.69	1.27×10^{-4}	201.44	325.14
Pcdhgc4	0.69	5.04×10^{-3}	2,276.19	3,672.87
Lin7b	0.69	5.13×10^{-5}	62.19	100.34
Gm45311	0.69	3.56×10^{-2}	17.47	28.18
Gja1	0.69	4.23×10^{-6}	1,271.90	2,050.96
Rreb1	0.69	3.29×10^{-3}	114.94	185.26
Tuba4a	0.69	4.48×10^{-5}	324.82	523.52
Rasl10a	0.69	4.76×10^{-2}	11.81	19.02
Slc6a9	0.69	4.65×10^{-10}	260.28	419.22
Klhl33	0.69	3.24×10^{-2}	24.99	40.25
Ppfia4	0.69	4.33×10^{-6}	691.87	1,114.13
Rassf4	0.69	6.17×10^{-4}	142.39	229.12
Stac	0.69	5.40×10^{-8}	91.58	147.36
Kcna2	0.69	5.61×10^{-11}	314.04	505.27
Klhdc1	0.69	5.51×10^{-4}	127.35	204.89

Gene ID	Log2 Fold Change	p-adj	Control CPM	Rad CPM
Gm17396	0.69	4.23×10^{-2}	15.26	24.55
Tmbim1	0.69	2.97×10^{-2}	67.18	108.08
2010300C02Rik	0.69	1.42×10^{-13}	232.97	374.65
Vasn	0.69	3.91×10^{-4}	64.34	103.47
Ptprn	0.68	1.31×10^{-10}	550.81	885.48
Col6a1	0.68	1.09×10^{-2}	430.46	691.62
C2cd2	0.68	9.90×10^{-4}	166.11	266.72
Col26a1	0.68	6.53×10^{-14}	597.29	959.02
Kcnc2	0.68	9.66×10^{-9}	130.80	210.01
Nhs	0.68	4.20×10^{-5}	158.58	254.57
Shfl	0.68	3.75×10^{-6}	115.45	185.31
Crabp2	0.68	1.24×10^{-3}	86.32	138.54
Dkkl1	0.68	1.97×10^{-2}	26.13	41.93
Lmntd1	0.68	1.69×10^{-2}	20.79	33.36
Clec14a	0.68	6.20×10^{-4}	103.83	166.60
H2-Q6	0.68	8.82×10^{-3}	31.88	51.13
Cfap46	0.68	1.36×10^{-2}	35.76	57.35
Dnm1	0.68	7.28×10^{-13}	2,494.99	4,001.13
Tnip2	0.68	1.91×10^{-4}	47.26	75.78
Mir99ahg	0.68	1.09×10^{-2}	77.12	123.60
Rasgef1a	0.68	2.68×10^{-12}	249.28	399.48
AC151602.1	0.68	2.88×10^{-2}	13.80	22.10
Enpep	0.68	1.22×10^{-3}	52.15	83.44
Ddr2	0.68	3.92×10^{-3}	228.25	365.11
Kctd11	0.68	2.62×10^{-3}	30.04	48.05
Zwilch	0.68	3.29×10^{-2}	65.19	104.23
Gulp1	0.68	3.99×10^{-4}	62.00	99.13
Grin3a	0.68	1.44×10^{-9}	1,060.13	1,694.55
Cd44	0.68	1.06×10^{-2}	73.48	117.44
Msx1	0.68	4.70×10^{-4}	131.35	209.77
Ptchd4	0.68	2.87×10^{-2}	22.08	35.25
Spag4	0.67	4.79×10^{-3}	39.39	62.88
Plek	0.67	7.67×10^{-4}	113.93	181.86
Gal3st1	0.67	3.57×10^{-3}	41.10	65.60
Adamts19	0.67	8.08×10^{-3}	24.29	38.77
Rhbdl3	0.67	6.13×10^{-7}	187.59	299.21
Lbhd2	0.67	2.85×10^{-2}	13.73	21.89
Gm42894	0.67	4.73×10^{-2}	11.92	18.99
Nmi	0.67	1.77×10^{-2}	22.52	35.86

Gene ID	Log2 Fold Change	p-adj	Control CPM	Rad CPM
Lrrk1	0.67	4.57×10^{-4}	86.34	137.44
Scg2	0.67	9.39×10^{-10}	1,196.01	1,903.49
Vwc2l	0.67	3.43×10^{-3}	109.55	174.35
Sostdc1	0.67	1.47×10^{-9}	173.57	276.18
Tmem37	0.67	1.51×10^{-2}	20.22	32.18
Kank1	0.67	7.29×10^{-6}	103.89	165.27
Stap2	0.67	4.80×10^{-3}	21.93	34.86
Cfp	0.67	5.19×10^{-3}	54.05	85.93
Adcy8	0.67	3.62×10^{-8}	184.32	293.05
Adgra1	0.67	1.74×10^{-4}	184.21	292.76
Galns	0.67	1.77×10^{-4}	64.31	102.19
Enpp2	0.67	4.59×10^{-12}	608.86	967.36
Myrip	0.67	2.22×10^{-3}	127.08	201.89
Wfs1	0.67	1.36×10^{-6}	195.61	310.75
Pacsin1	0.67	5.34×10^{-13}	638.23	1,012.48
Pfkp	0.67	5.16×10^{-8}	1,710.82	2,713.70
Tmem117	0.67	6.98×10^{-5}	77.91	123.55
Pde8a	0.67	1.97×10^{-3}	43.92	69.64
Gdf5	0.66	4.87×10^{-3}	29.54	46.83
Mrc2	0.66	1.09×10^{-3}	251.94	399.40
Cmklr1	0.66	9.24×10^{-3}	31.56	50.00
Col4a5	0.66	5.07×10^{-9}	263.56	417.36
Rasgrp1	0.66	6.83×10^{-6}	328.42	519.98
Arhgap45	0.66	1.68×10^{-2}	31.40	49.70
Col6a2	0.66	3.47×10^{-2}	422.53	668.68
Slc9a9	0.66	6.28×10^{-5}	65.60	103.79
Cd55	0.66	7.11×10^{-3}	61.86	97.84
Rarres2	0.66	5.52×10^{-3}	39.74	62.83
Dpp4	0.66	1.43×10^{-2}	31.09	49.14
Gxylt2	0.66	1.43×10^{-2}	51.23	80.94
Thy1	0.66	6.01×10^{-8}	530.75	838.51
Nhlh2	0.66	1.07×10^{-4}	161.56	255.24
Nipal3	0.66	1.09×10^{-9}	492.27	777.23
Sdc4	0.66	1.55×10^{-5}	85.41	134.64
Vwa5b2	0.66	1.28×10^{-2}	151.66	239.03
Sphk1	0.66	3.89×10^{-2}	62.73	98.79
Gm4316	0.65	1.82×10^{-4}	41.50	65.34
Gprc5c	0.65	5.04×10^{-4}	68.42	107.71
Smoc2	0.65	4.43×10^{-2}	54.99	86.56

Gene ID	Log2 Fold Change	p-adj	Control CPM	Rad CPM
Pidd1	0.65	5.20×10^{-6}	78.36	123.35
Cacna1a	0.65	4.49×10^{-10}	604.12	950.68
Creb3l1	0.65	1.59×10^{-3}	85.79	134.98
Grid1	0.65	6.88×10^{-8}	490.61	771.63
Gm28661	0.65	2.57×10^{-2}	14,881.40	23,391.78
Heph	0.65	9.76×10^{-5}	63.82	100.29
H19	0.65	1.58×10^{-2}	3,798.92	5,969.01
Vat1l	0.65	3.03×10^{-16}	1,362.21	2,139.77
Ankrd63	0.65	1.33×10^{-3}	51.68	81.14
Lpp	0.65	7.76×10^{-4}	231.64	363.56
B3galt5	0.65	6.22×10^{-4}	75.23	118.06
Bmp3	0.65	1.71×10^{-3}	83.57	131.11
Itpr3	0.65	2.79×10^{-3}	72.35	113.51
Aqp4	0.65	6.24×10^{-4}	169.72	266.24
Synj2	0.65	1.66×10^{-3}	68.19	106.95
Hbb-bt	0.65	2.48×10^{-2}	4,464.42	7,001.21
Clmn	0.65	3.62×10^{-3}	121.84	191.02
Tmem268	0.65	1.48×10^{-3}	71.55	112.08
Gm44250	0.65	3.37×10^{-2}	22.55	35.32
Nfkb2	0.65	2.57×10^{-3}	56.88	89.08
Mirg	0.65	5.71×10^{-4}	86.97	136.18
Manba	0.65	5.51×10^{-4}	63.51	99.40
Gm36995	0.65	4.41×10^{-2}	49.25	77.07
Man2a1	0.65	3.21×10^{-7}	323.43	506.06
Nbl1	0.65	2.23×10^{-7}	447.48	700.01
Nrsn2	0.65	3.96×10^{-9}	206.44	322.95
Pdgfra	0.65	1.19×10^{-8}	731.93	1,144.82
Trim66	0.64	5.64×10^{-3}	41.86	65.45
Nfe2l2	0.64	3.42×10^{-7}	150.71	235.46
Lcp1	0.64	8.15×10^{-4}	190.61	297.76
Camk2a	0.64	4.30×10^{-10}	1,383.40	2,160.84
Entpd1	0.64	1.17×10^{-3}	127.94	199.83
Zim1	0.64	5.02×10^{-3}	277.05	432.26
A230057D06Rik	0.64	1.09×10^{-2}	61.25	95.57
Me3	0.64	7.32×10^{-3}	120.01	187.24
Tmem132d	0.64	9.20×10^{-6}	153.29	239.15
Prkcb	0.64	3.97×10^{-11}	910.65	1,420.36
Efhc1	0.64	9.40×10^{-3}	18.68	29.10
Stat6	0.64	1.16×10^{-3}	89.22	138.96

Gene ID	Log2 Fold Change	p-adj	Control CPM	Rad CPM
Plaat1	0.64	3.04×10^{-3}	44.15	68.76
Cyth4	0.64	2.44×10^{-3}	67.31	104.80
Pcolce	0.64	1.49×10^{-2}	144.80	225.30
Col9a2	0.64	5.62×10^{-6}	205.78	320.10
Il34	0.64	1.45×10^{-2}	56.46	87.82
Tmc6	0.64	1.49×10^{-2}	31.37	48.79
Cx3cl1	0.64	3.60×10^{-11}	807.88	1,256.27
Sv2c	0.64	3.11×10^{-7}	323.74	503.29
Nptxr	0.64	2.93×10^{-16}	2,446.71	3,803.60
Ddit4l	0.64	1.49×10^{-2}	42.16	65.50
Map7d2	0.64	4.43×10^{-7}	372.42	578.65
Hlx	0.64	4.50×10^{-2}	19.34	30.05
Slc22a3	0.64	5.19×10^{-3}	32.02	49.75
Rcan2	0.63	4.69×10^{-5}	548.42	851.57
Grk3	0.63	3.36×10^{-7}	445.93	692.34
Mrs2	0.63	2.00×10^{-5}	511.02	793.29
Retsat	0.63	3.90×10^{-2}	56.23	87.27
Ramp1	0.63	3.84×10^{-4}	109.49	169.92
Efcc1	0.63	3.97×10^{-2}	30.79	47.78
Cdh18	0.63	9.38×10^{-7}	100.76	156.32
Firre	0.63	1.06×10^{-10}	315.76	489.78
Smim1	0.63	2.15×10^{-2}	48.80	75.69
Trpm2	0.63	1.07×10^{-2}	90.72	140.51
Krt222	0.63	2.72×10^{-3}	49.27	76.30
Tor4a	0.63	1.62×10^{-2}	35.94	55.66
Parm1	0.63	1.05×10^{-6}	175.00	270.95
Thsd4	0.63	2.16×10^{-2}	190.24	294.41
Smco3	0.63	1.57×10^{-2}	23.45	36.29
Ndst4	0.63	4.29×10^{-2}	116.18	179.77
Onecut2	0.63	1.19×10^{-2}	83.53	129.25
Ppp1r3g	0.63	4.83×10^{-2}	14.11	21.82
mt-Nd1	0.63	1.63×10^{-2}	10,202.96	15,783.00
Whrn	0.63	5.21×10^{-6}	266.53	412.11
Clec3b	0.63	3.15×10^{-3}	62.35	96.38
Arc	0.63	7.67×10^{-6}	78.22	120.89
AW551984	0.63	1.06×10^{-4}	1,055.92	1,631.22
Lamc1	0.63	1.08×10^{-8}	963.06	1,486.88
Vtn	0.63	2.43×10^{-6}	574.86	887.50
Il1rapl2	0.63	2.21×10^{-2}	27.68	42.73

Gene ID	Log2 Fold Change	p-adj	Control CPM	Rad CPM
Calb1	0.63	7.64×10^{-8}	404.27	623.80
Sowaha	0.63	4.13×10^{-7}	139.18	214.69
Hrh1	0.62	1.25×10^{-2}	33.18	51.17
Pced1b	0.62	3.09×10^{-3}	51.20	78.93
Cped1	0.62	3.06×10^{-2}	96.34	148.50
H6pd	0.62	3.31×10^{-5}	98.84	152.36
Adamts2	0.62	1.35×10^{-4}	202.36	311.89
Gm20605	0.62	2.68×10^{-2}	116.33	179.27
Lrrc3b	0.62	2.33×10^{-5}	86.15	132.75
Peg3	0.62	3.33×10^{-10}	2,007.27	3,092.56
Cyba	0.62	2.83×10^{-4}	102.47	157.86
Gpr157	0.62	4.13×10^{-2}	25.63	39.48
Slc24a2	0.62	2.87×10^{-3}	92.80	142.93
Slc8b1	0.62	1.08×10^{-2}	31.52	48.53
Spata33	0.62	1.98×10^{-2}	51.48	79.24
Cyb561	0.62	1.05×10^{-8}	285.20	439.04
Pdxk	0.62	8.63×10^{-30}	743.69	1,144.41
Sorcs3	0.62	6.28×10^{-8}	254.97	392.06
Ly6e	0.62	6.54×10^{-16}	712.95	1,095.93
Uhmk1	0.62	6.67×10^{-12}	1,056.02	1,622.89
Tac1	0.62	1.29×10^{-8}	341.61	524.92
Necab2	0.62	8.38×10^{-7}	353.71	543.49
Ghr	0.62	1.17×10^{-4}	61.71	94.78
Ecm1	0.62	3.52×10^{-2}	56.08	86.12
Gpsm3	0.62	3.61×10^{-2}	29.18	44.81
Wnt9a	0.62	7.55×10^{-5}	50.40	77.34
Col13a1	0.62	3.35×10^{-2}	31.25	47.96
Fam129a	0.62	4.85×10^{-2}	45.26	69.45
Gm2136	0.62	6.69×10^{-3}	38.40	58.88
Ahnak	0.62	8.09×10^{-5}	531.64	815.29
Cep164	0.62	1.38×10^{-3}	278.68	427.24
Sdk1	0.62	2.98×10^{-6}	263.47	403.87
Pdzd2	0.62	1.15×10^{-5}	449.35	688.74
Ccdc74a	0.62	3.33×10^{-2}	24.11	36.95
Creg1	0.62	1.51×10^{-5}	157.48	241.34
Dock2	0.62	1.90×10^{-2}	28.78	44.10
Tmco4	0.62	4.11×10^{-2}	18.90	28.96
Fam163b	0.61	2.00×10^{-5}	355.23	543.92
Phldb2	0.61	2.76×10^{-3}	149.19	228.32

Gene ID	Log2 Fold Change	p-adj	Control CPM	Rad CPM
Frem1	0.61	3.57×10^{-3}	103.11	157.74
Pdlim2	0.61	1.30×10^{-2}	51.29	78.45
Kcnj16	0.61	5.59×10^{-3}	31.27	47.77
Sh3bp1	0.61	9.30×10^{-3}	71.99	109.94
Dact2	0.61	2.90×10^{-2}	89.56	136.77
Cpq	0.61	9.01×10^{-4}	80.76	123.30
Tmie	0.61	1.91×10^{-3}	37.82	57.73
Itga10	0.61	3.18×10^{-2}	31.92	48.71
Gabrg1	0.61	3.12×10^{-5}	191.73	292.55
Fgf2	0.61	1.96×10^{-2}	32.32	49.28
Cntnap5a	0.61	6.43×10^{-5}	266.71	406.46
Nrip3	0.61	1.18×10^{-6}	190.19	289.82
Plxnd1	0.61	3.03×10^{-6}	1,907.31	2,906.32
Dgkk	0.61	5.71×10^{-3}	158.59	241.45
Fbxo2	0.61	2.00×10^{-3}	71.20	108.39
Selenbp1	0.61	2.44×10^{-3}	78.22	119.06
BC022960	0.61	4.41×10^{-2}	27.73	42.21
Neurl1a	0.61	8.63×10^{-15}	629.69	957.83
Lef1	0.61	1.10×10^{-2}	120.45	183.21
Pde8b	0.61	5.64×10^{-4}	107.56	163.61
Tbc1d2	0.60	1.31×10^{-2}	25.22	38.35
Fam114a1	0.60	1.12×10^{-2}	102.63	156.09
Ehbp1l1	0.60	1.24×10^{-3}	386.04	586.90
Plcxd1	0.60	3.97×10^{-2}	57.10	86.78
Spa17	0.60	2.95×10^{-2}	20.14	30.60
Nr1h3	0.60	4.52×10^{-2}	19.12	29.03
Fads6	0.60	1.14×10^{-4}	75.20	114.11
Six5	0.60	4.35×10^{-4}	63.57	96.45
Filip1	0.60	8.02×10^{-4}	92.24	139.90
Rmi2	0.60	4.10×10^{-2}	25.17	38.17
Atp1a2	0.60	1.01×10^{-11}	3,147.52	4,771.40
Arl13b	-0.60	4.31×10^{-6}	310.04	204.22
Lmo1	-0.60	2.55×10^{-5}	394.35	259.49
Slc16a7	-0.60	1.59×10^{-4}	617.62	406.40
Gm7332	-0.60	3.56×10^{-3}	121.54	79.92
Rabepk	-0.60	9.34×10^{-3}	214.05	140.75
Mycn	-0.61	6.15×10^{-13}	1,712.47	1,125.42
Abracl	-0.61	1.58×10^{-5}	392.47	257.75
Nudcd1	-0.61	5.15×10^{-4}	335.29	219.65

Gene ID	Log2 Fold Change	p-adj	Control CPM	Rad CPM
Tacr3	-0.61	1.92×10^{-2}	51.97	33.97
Gm8399	-0.61	4.93×10^{-2}	24.42	15.95
Srl	-0.61	8.38×10^{-6}	591.85	386.67
Rasgef1b	-0.61	1.16×10^{-14}	799.19	521.85
Mef2c	-0.62	4.65×10^{-10}	4,660.56	3,042.06
Poc5	-0.62	5.89×10^{-7}	661.20	431.55
Gucy1b1	-0.62	7.74×10^{-16}	1,711.77	1,114.37
A930017M01Rik	-0.62	8.01×10^{-3}	130.95	85.21
Nts	-0.62	1.58×10^{-5}	568.78	370.06
Csrp2	-0.62	5.14×10^{-11}	1,285.75	836.18
Gm8822	-0.62	9.50×10^{-4}	73.23	47.54
Ccng2	-0.62	1.62×10^{-10}	1,665.69	1,081.20
Cdkn1b	-0.62	8.67×10^{-15}	2,170.59	1,408.00
Pycr1	-0.63	4.09×10^{-6}	149.77	97.10
Gm10146	-0.63	3.93×10^{-3}	153.28	99.32
A330102I10Rik	-0.63	1.48×10^{-2}	46.48	30.05
2700033N17Rik	-0.63	1.69×10^{-3}	58.38	37.71
Map2k6	-0.63	1.27×10^{-12}	451.96	291.38
BC025920	-0.63	1.73×10^{-4}	93.07	59.94
Mterf1a	-0.64	2.50×10^{-3}	56.54	36.38
Efna4	-0.64	1.27×10^{-5}	150.25	96.47
Gm46409	-0.64	3.16×10^{-2}	37.87	24.30
Slc25a47	-0.64	6.58×10^{-5}	82.05	52.63
Tcea1-ps1	-0.64	1.08×10^{-2}	52.63	33.76
Syt6	-0.64	7.04×10^{-10}	789.97	506.57
Zfp953	-0.64	4.90×10^{-2}	23.10	14.80
Trim13	-0.64	4.93×10^{-8}	358.34	229.31
Mynn	-0.64	1.62×10^{-7}	512.85	328.05
Ssbp2	-0.64	4.99×10^{-23}	2,454.26	1,569.49
Gm41760	-0.65	1.54×10^{-2}	40.27	25.71
Lhx2	-0.65	2.32×10^{-10}	1,883.80	1,202.24
Zfp879	-0.65	1.93×10^{-2}	54.48	34.75
Rab39	-0.65	1.32×10^{-2}	38.53	24.57
Olfml2b	-0.65	3.66×10^{-8}	168.68	107.44
Gm29260	-0.65	1.47×10^{-3}	96.70	61.56
Cttnbp2	-0.65	1.85×10^{-4}	1,459.98	929.19
Klhl35	-0.65	1.06×10^{-2}	65.71	41.77
Kcnh5	-0.65	5.04×10^{-4}	57.52	36.54
Gm42918	-0.65	3.18×10^{-2}	21.43	13.61

Gene ID	Log2 Fold Change	p-adj	Control CPM	Rad CPM
Npy1r	-0.66	1.64×10^{-10}	230.23	146.08
Upp2	-0.66	1.55×10^{-2}	96.87	61.42
Zfp719	-0.66	1.45×10^{-7}	310.19	196.39
Skap1	-0.66	4.07×10^{-2}	24.61	15.56
Cntn2	-0.67	1.14×10^{-6}	6,737.46	4,244.09
Emx1	-0.67	9.71×10^{-10}	429.39	270.02
Fastkd3	-0.67	1.47×10^{-2}	193.35	121.32
Tradd	-0.68	4.60×10^{-7}	132.48	82.85
Syt4	-0.68	8.28×10^{-30}	4,965.59	3,098.03
Fbxo32	-0.68	2.21×10^{-5}	178.85	111.58
Ldb2	-0.69	1.72×10^{-24}	994.72	618.49
Gm6501	-0.69	9.91×10^{-3}	44.86	27.80
S1pr5	-0.69	1.65×10^{-3}	50.68	31.33
Gm5171	-0.69	3.17×10^{-2}	19.90	12.30
Tgfbr1	-0.70	1.54×10^{-14}	1,262.56	779.85
Acvr1	-0.70	1.27×10^{-19}	700.67	432.33
Gm17259	-0.70	4.70×10^{-2}	21.24	13.07
Neurod2	-0.70	7.17×10^{-9}	3,654.63	2,246.57
Cd24a	-0.71	5.01×10^{-18}	11,270.62	6,912.53
Gucy1a2	-0.71	5.53×10^{-3}	108.19	66.21
E430018J23Rik	-0.71	7.28×10^{-3}	93.19	57.00
Rspo3	-0.71	6.11×10^{-6}	155.97	95.33
Gm5468	-0.71	4.34×10^{-3}	53.93	32.86
Gm29562	-0.71	4.21×10^{-2}	46.40	28.27
Pcdh20	-0.72	7.43×10^{-5}	224.01	136.32
2900009J06Rik	-0.72	2.62×10^{-2}	38.24	23.26
Igfbpl1	-0.72	4.49×10^{-10}	4,839.26	2,938.64
Rnf182	-0.72	6.00×10^{-16}	858.96	520.23
Tmsb15l	-0.73	5.13×10^{-3}	57.34	34.66
Fam49a	-0.73	2.06×10^{-14}	4,612.72	2,786.30
Faap24	-0.73	1.34×10^{-5}	145.99	88.13
Tiam2	-0.73	1.18×10^{-11}	2,698.96	1,628.87
Rspo2	-0.73	5.85×10^{-9}	162.73	98.01
Zfp874a	-0.73	2.85×10^{-2}	117.21	70.49
Aste1	-0.74	3.54×10^{-3}	91.01	54.42
A330084C13Rik	-0.74	7.87×10^{-7}	228.40	136.28
Gm48161	-0.75	3.98×10^{-3}	114.31	68.05
Gm43517	-0.75	2.49×10^{-3}	77.76	46.22
Masp1	-0.75	2.00×10^{-20}	1,037.83	616.24

Gene ID	Log2 Fold Change	p-adj	Control CPM	Rad CPM
Rgs6	-0.76	6.15×10^{-19}	626.22	370.46
Gpr21	-0.76	3.32×10^{-13}	528.09	312.35
Foxp1	-0.76	1.01×10^{-22}	2,618.45	1,547.50
Gtdc1	-0.76	1.41×10^{-6}	698.41	412.65
Tshr	-0.76	9.34×10^{-3}	46.03	27.14
Gpr6	-0.76	2.34×10^{-3}	39.42	23.23
Vax1	-0.77	8.58×10^{-4}	39.05	22.97
Fastkd2	-0.77	1.73×10^{-4}	239.06	140.50
Nkx2-3	-0.77	1.34×10^{-2}	32.47	19.06
Gm12063	-0.77	1.60×10^{-2}	37.38	21.92
Atp13a5	-0.77	1.24×10^{-2}	41.60	24.33
Slc18a2	-0.77	1.22×10^{-4}	154.99	90.64
Pou3f1	-0.78	1.39×10^{-9}	1,040.94	606.23
Aff2	-0.78	1.22×10^{-4}	453.75	263.52
Lix1	-0.79	2.76×10^{-20}	371.07	215.23
Neurod1	-0.79	6.17×10^{-12}	537.76	311.66
Nckap5	-0.79	1.17×10^{-5}	604.59	350.08
Tmod1	-0.79	4.09×10^{-2}	37.53	21.70
Crym	-0.81	3.59×10^{-11}	623.90	357.09
Sema7a	-0.81	1.43×10^{-34}	1,301.56	743.70
Mkx	-0.81	1.79×10^{-2}	58.41	33.31
Mlip	-0.81	4.01×10^{-7}	180.37	102.72
Kcnj2	-0.82	1.01×10^{-14}	175.67	99.48
Ikzf1	-0.82	1.13×10^{-6}	208.10	117.84
Gm4724	-0.82	5.29×10^{-3}	176.94	100.00
Thrsp	-0.83	2.26×10^{-2}	132.54	74.79
Gm1818	-0.83	6.20×10^{-5}	82.37	46.34
Impg2	-0.83	1.18×10^{-2}	20.25	11.38
Msra	-0.84	3.03×10^{-16}	650.02	363.59
Bnip3l-ps	-0.84	2.82×10^{-2}	43.78	24.46
Gm10145	-0.85	3.45×10^{-2}	24.52	13.64
Bhlhe22	-0.85	2.11×10^{-27}	2,846.34	1,582.72
Mpped1	-0.85	5.95×10^{-28}	8,309.46	4,618.99
Gdf9	-0.85	8.06×10^{-3}	28.79	15.99
Zeb2	-0.85	1.32×10^{-14}	3,895.77	2,157.69
Id2	-0.86	2.89×10^{-23}	2,242.93	1,239.10
Hgf	-0.86	2.84×10^{-2}	38.28	21.11
Nfix	-0.87	4.57×10^{-19}	9,449.60	5,181.40
Zmym1	-0.87	5.31×10^{-5}	302.23	165.69

Gene ID	Log2 Fold Change	p-adj	Control CPM	Rad CPM
Gm37494	-0.87	3.48×10^{-3}	713.49	391.08
Fezf2	-0.88	2.91×10^{-18}	785.71	427.64
Gm3055	-0.88	3.65×10^{-2}	28.80	15.64
Gm17750	-0.88	3.46×10^{-9}	328.51	178.21
Has2	-0.89	4.77×10^{-5}	50.10	26.96
Lipg	-0.90	1.38×10^{-12}	183.51	98.09
Prokr2	-0.91	3.79×10^{-4}	285.88	152.59
9930014A18Rik	-0.91	2.58×10^{-8}	151.30	80.72
Sema3c	-0.91	1.04×10^{-27}	1,248.77	665.37
Neb	-0.92	1.09×10^{-4}	179.79	95.13
Tfap2d	-0.92	9.91×10^{-5}	82.54	43.62
Usp43	-0.93	3.45×10^{-7}	214.27	112.15
Gm42778	-0.94	2.67×10^{-3}	90.35	47.07
Ighm	-0.94	1.98×10^{-2}	25.48	13.27
9130017K11Rik	-0.95	4.77×10^{-2}	14.44	7.48
Epha4	-0.95	2.41×10^{-47}	2,661.26	1,378.23
E130102H24Rik	-0.95	4.37×10^{-2}	21.13	10.93
Hist1h1e	-0.95	1.85×10^{-2}	33.52	17.32
Cldn3	-0.98	3.29×10^{-5}	73.53	37.31
Slc26a8	-1.00	3.39×10^{-2}	31.96	15.99
Zbtb18	-1.00	5.45×10^{-50}	8,988.67	4,493.89
Tmem74	-1.01	1.01×10^{-16}	402.37	200.24
Lpl	-1.01	6.15×10^{-13}	823.09	407.40
Gm43654	-1.04	1.65×10^{-2}	14.74	7.17
Gm12198	-1.05	9.92×10^{-4}	19.74	9.56
Gm27021	-1.05	1.64×10^{-3}	23.92	11.57
Fsd2	-1.06	4.36×10^{-2}	13.74	6.57
Immp2l	-1.06	4.16×10^{-3}	24.04	11.50
Dct	-1.07	1.17×10^{-3}	67.88	32.33
Rpl9-ps7	-1.08	1.32×10^{-2}	19.57	9.28
Dok5	-1.08	1.53×10^{-39}	1,068.04	504.33
Gm30382	-1.09	4.12×10^{-3}	15.81	7.41
Ntf3	-1.11	9.87×10^{-11}	97.26	45.02
Tac2	-1.14	9.85×10^{-4}	25.65	11.67
Gucy1a1	-1.14	2.21×10^{-48}	2,971.41	1,344.39
Aox4	-1.15	7.80×10^{-24}	232.24	104.67
9430053O09Rik	-1.15	2.00×10^{-9}	58.17	26.13
Ly6g6e	-1.18	4.56×10^{-4}	31.17	13.71
Neurod6	-1.20	4.30×10^{-29}	5,099.76	2,214.58

Gene ID	Log2 Fold Change	p-adj	Control CPM	Rad CPM
Gm40841	-1.21	1.46×10^{-6}	63.87	27.58
Deup1	-1.21	1.56×10^{-4}	24.18	10.42
Nr4a3	-1.24	1.30×10^{-40}	871.18	368.02
Crlf1	-1.26	6.12×10^{-8}	172.24	72.04
Aspn	-1.28	4.59×10^{-3}	52.17	21.51
Cers3	-1.29	3.93×10^{-2}	15.83	6.49
Ermn	-1.29	9.90×10^{-7}	35.95	14.70
Pdlim3	-1.31	9.15×10^{-3}	34.41	13.86
Grp	-1.32	3.87×10^{-9}	73.28	29.37
Ccno	-1.34	2.24×10^{-3}	15.69	6.21
9130024F11Rik	-1.36	3.25×10^{-16}	616.78	240.89
Satb2	-1.37	1.92×10^{-19}	3,465.83	1,339.89
Hspb3	-1.40	2.54×10^{-3}	17.39	6.60
Apobec2	-1.40	4.83×10^{-2}	15.48	5.87
Gm26801	-1.40	1.14×10^{-3}	17.16	6.50
Ccbe1	-1.40	4.50×10^{-22}	288.14	108.94
Kcns3	-1.41	2.18×10^{-3}	16.29	6.13
A830009L08Rik	-1.42	1.39×10^{-3}	18.45	6.89
Itprid1	-1.43	9.13×10^{-6}	46.28	17.21
Slc6a4	-1.47	3.27×10^{-4}	42.27	15.23
Gm10516	-1.48	4.76×10^{-2}	29.08	10.40
1110002E22Rik	-1.56	1.88×10^{-5}	33.02	11.22
Gm4081	-1.57	1.41×10^{-4}	15.10	5.10
Dsc3	-1.63	2.78×10^{-7}	51.80	16.75
3110045C21Rik	-1.64	3.67×10^{-3}	15.46	4.94
Adh7	-1.68	3.33×10^{-2}	16.49	5.13
Casq1	-1.81	1.95×10^{-5}	82.11	23.46
Casq2	-1.89	9.71×10^{-3}	34.88	9.41
Ccr5	-1.89	2.05×10^{-2}	39.15	10.55
Hdc	-1.90	3.49×10^{-4}	106.32	28.52
P2ry12	-1.91	1.51×10^{-15}	89.96	23.96
Hist2h2aa1	-1.93	1.59×10^{-2}	25.95	6.80
Crybb1	-2.04	2.96×10^{-17}	44.54	10.80
Klhl41	-2.36	8.79×10^{-3}	20.44	3.98
Lhcgr	-2.48	7.75×10^{-7}	48.29	8.63
Ltf	-2.61	1.64×10^{-3}	18.02	2.96
Tpsb2	-2.62	2.29×10^{-5}	113.93	18.50
Piwil2	-2.86	5.44×10^{-3}	18.62	2.57
Il1rl1	-2.99	3.80×10^{-5}	19.38	2.44

**Gene ID**	**Log2 Fold Change**	**p-adj**	**Control CPM**	**Rad CPM**
Cma1	-3.13	4.70×10^{-7}	120.05	13.73
Msx3	-3.91	2.00×10^{-20}	29.86	1.99

Supplementary Table S4: Differentially expressed genes in the male diencephalon following gestational radiation exposure.

All 25 genes differentially expressed between control and gestationally irradiated male diencephalon (n = 8 per sex per treatment group, one fetus per sex per dam; sham: 8 dams, irradiated: 9 dams), identified by DESeq2 as log2 fold change ≥ 0.6 or ≤ - 0.6 with a Benjamini-Hochberg false discovery rate (FDR) adjusted p-value (p-adj) <0.05. Genes are ranked by descending log2 fold change, where positive values indicate increased expression relative to control (16 upregulated, 9 downregulated). Control CPM and Rad CPM are mean normalized counts per million for each group.

**Gene ID**	**Log2 Fold Change**	**p-adj**	**Control CPM**	**Rad CPM**
Gpnmb	2.10	3.26×10^{-2}	9.38	40.29
Ms4a6d	1.80	4.59×10^{-4}	8.12	28.20
Lyz2	1.78	8.08×10^{-9}	46.68	160.38
Siglec1	1.66	8.09×10^{-5}	9.32	29.46
Eda2r	1.60	2.43×10^{-5}	19.79	60.12
Ifit3	1.43	4.87×10^{-2}	8.00	21.60
Phospho1	1.14	4.17×10^{-2}	27.85	61.25
Psrc1	1.08	7.96×10^{-4}	38.65	81.64
Rsad2	0.94	2.65×10^{-2}	33.12	63.43
Lgals9	0.83	2.13×10^{-2}	62.15	110.81
Plau	0.82	2.02×10^{-3}	39.54	69.85
Ephx1	0.75	8.09×10^{-5}	84.60	142.16
Tor3a	0.73	4.14×10^{-2}	50.57	84.03
P2rx7	0.65	4.99×10^{-2}	74.21	116.55
Phlda3	0.60	2.02×10^{-5}	197.08	299.50
Parp12	0.60	3.87×10^{-2}	49.30	74.87
Atp13a5	-0.65	8.97×10^{-3}	103.14	65.94
Vsir	-0.86	3.22×10^{-3}	128.57	70.74
Ighm	-1.33	9.67×10^{-5}	33.60	13.37
Siglech	-1.41	4.99×10^{-2}	17.05	6.42
Crybb1	-1.45	4.14×10^{-2}	38.28	14.02
Lncppara	-1.54	4.99×10^{-2}	35.86	12.36
P2ry12	-1.68	1.68×10^{-17}	100.14	31.24
Kcng4	-1.81	4.96×10^{-4}	62.63	17.86
Gm9780	-2.56	9.61×10^{-3}	25.93	4.41

Supplementary Table S5: Differentially expressed genes in the female diencephalon following gestational radiation exposure.

All 52 genes differentially expressed between control and gestationally irradiated female diencephalon (n = 8 per sex per treatment group, one fetus per sex per dam; sham: 8 dams, irradiated: 9 dams), identified by DESeq2 as log2 fold change ≥ 0.6 or ≤ - 0.6 with a Benjamini-Hochberg false discovery rate (FDR) adjusted p-value (p-adj) <0.05. Genes are ranked by descending log2 fold change, where positive values indicate increased expression relative to control (44 upregulated, 8 downregulated). Control CPM and Rad CPM are mean normalized counts per million for each group.

Gene ID	Log2 Fold Change	p-adj	Control CPM	Rad CPM
Gm10273	22.84	8.71×10^{-11}	7.13×10^{-6}	53.54
Ifit1	2.67	8.90×10^{-3}	3.00	19.06
Siglec1	2.58	5.20×10^{-5}	6.34	38.03
Gpnmb	2.22	6.02×10^{-4}	7.47	34.94
Eda2r	1.95	5.57×10^{-12}	14.07	54.53
Synpo2l	1.89	4.34×10^{-2}	6.50	24.13
Lyz2	1.74	5.20×10^{-5}	46.54	155.64
Ms4a6c	1.55	2.13×10^{-2}	9.33	27.38
Msr1	1.55	4.34×10^{-2}	5.69	16.65
Lyve1	1.51	4.21×10^{-3}	37.20	106.14
Ms4a6d	1.25	3.87×10^{-2}	10.86	25.78
Edn3	1.23	3.94×10^{-2}	38.90	91.31
Bst2	1.20	2.13×10^{-2}	33.02	75.62
Gbp9	1.16	3.40×10^{-2}	22.37	49.91
Tnfrsf10b	1.14	3.91×10^{-2}	10.64	23.51
Plekha2	1.06	1.59×10^{-2}	41.21	85.78
Psrc1	1.04	2.50×10^{-2}	36.63	75.31
Irgm1	1.03	5.20×10^{-5}	29.13	59.60
F13a1	1.01	5.46×10^{-3}	142.52	287.76
Parp14	0.98	3.73×10^{-2}	19.09	37.77
Ifitm3	0.98	2.76×10^{-4}	62.84	123.55
Icam1	0.95	2.21×10^{-2}	25.38	49.01
Tmem252	0.93	1.59×10^{-2}	15.86	30.27
Dusp27	0.90	4.32×10^{-2}	21.63	40.45
Adamts12	0.87	3.01×10^{-2}	142.93	261.10
Fam114a1	0.85	8.90×10^{-3}	63.51	114.46
Lgals9	0.82	8.90×10^{-3}	61.40	108.48
Slc16a12	0.80	2.27×10^{-2}	43.10	74.78
Flnc	0.79	1.87×10^{-2}	220.09	380.50
Slfn9	0.79	1.67×10^{-3}	41.64	71.91
Gm47761	0.79	3.62×10^{-2}	50.55	87.28

Gene ID	Log2 Fold Change	p-adj	Control CPM	Rad CPM
Tubb6	0.78	5.20×10^{-5}	85.32	146.67
Kif2c	0.76	2.76×10^{-3}	46.30	78.20
Gbp7	0.74	3.33×10^{-2}	45.27	75.64
Pmp22	0.73	5.20×10^{-5}	131.58	217.96
Calcrl	0.71	1.67×10^{-3}	110.73	181.39
Stab1	0.68	3.50×10^{-2}	339.46	544.04
B2m	0.67	2.50×10^{-2}	124.04	196.91
Arhgap29	0.66	1.87×10^{-2}	350.23	552.13
Ehd2	0.64	3.01×10^{-2}	149.85	232.72
Nfe2l2	0.63	1.03×10^{-2}	95.07	147.62
Slc38a4	0.63	4.14×10^{-2}	55.04	85.28
Prc1	0.62	1.55×10^{-2}	181.48	278.31
Lamb1	0.61	3.01×10^{-2}	429.87	656.33
Cck	-0.64	2.25×10^{-2}	198.90	127.81
Dnah7b	-0.67	2.29×10^{-2}	399.15	251.31
Col11a2	-0.71	2.42×10^{-5}	553.66	338.71
Gpr179	-0.97	1.03×10^{-2}	89.86	45.99
Kcng4	-1.04	3.01×10^{-2}	42.77	20.80
Rax	-1.25	4.72×10^{-2}	42.02	17.70
P2ry12	-1.39	5.57×10^{-12}	81.70	31.25
Crybb1	-2.00	3.91×10^{-9}	43.64	10.94

Supplementary Table S6: Differentially expressed genes in the male midbrain following gestational radiation exposure.

All 76 genes differentially expressed between control and gestationally irradiated male midbrain (n = 8 per sex per treatment group, one fetus per sex per dam; sham: 8 dams, irradiated: 9 dams), identified by DESeq2 as log2 fold change ≥ 0.6 or ≤ - 0.6 with a Benjamini-Hochberg false discovery rate (FDR) adjusted p-value (p-adj) <0.05. Genes are ranked by descending log2 fold change, where positive values indicate increased expression relative to control (62 upregulated, 14 downregulated). Control CPM and Rad CPM are mean normalized counts per million for each group.

Gene ID	Log2 Fold Change	p-adj	Control CPM	Rad CPM
Gm21378	7.18	2.99×10^{-2}	0.19	27.58
Gpnmb	2.40	7.23×10^{-8}	9.28	49.06
Ifit1	2.20	3.42×10^{-3}	4.54	20.81
Mfsd7a	2.08	1.61×10^{-2}	3.95	16.72
Siglec1	2.01	1.05×10^{-6}	8.68	34.89
Eda2r	1.99	4.38×10^{-5}	13.58	53.88
Pkhd1l1	1.95	2.27×10^{-2}	9.75	37.53
Gm37795	1.93	6.31×10^{-3}	4.79	18.23
Dmkn	1.76	3.84×10^{-2}	16.27	55.19
Fev	1.63	1.77×10^{-4}	16.88	52.09
Tph2	1.57	3.30×10^{-6}	71.37	211.46
Lyz2	1.56	3.53×10^{-7}	73.76	217.14
Slc6a4	1.43	7.72×10^{-9}	178.58	480.53
C130021I20Rik	1.34	4.69×10^{-4}	209.91	532.62
Dnah12	1.33	1.15×10^{-2}	19.89	49.89
Lmx1b	1.29	2.97×10^{-6}	124.80	304.96
Fam166b	1.26	1.26×10^{-2}	9.08	21.77
Gm42047	1.21	2.85×10^{-4}	487.36	1,124.18
Dnaic2	1.16	2.00×10^{-3}	29.56	66.26
Pqlc3	1.16	2.05×10^{-2}	9.75	21.79
Crocc2	1.15	4.38×10^{-5}	49.74	110.53
2610028E06Rik	1.10	3.32×10^{-2}	14.25	30.60
Gm47761	1.07	1.09×10^{-2}	44.24	92.69
Bst2	1.06	6.09×10^{-5}	32.56	67.75
Vsx2	1.04	1.57×10^{-2}	23.31	48.03
Map3k19	1.01	2.82×10^{-2}	31.15	62.87
Mapkapk3	1.00	4.18×10^{-2}	20.84	41.74
Pidd1	0.99	9.21×10^{-3}	48.45	96.54
Ghsr	0.98	2.34×10^{-2}	18.83	37.25
Rsad2	0.95	7.40×10^{-3}	45.25	87.59
Ttc21a	0.94	5.65×10^{-3}	38.55	73.97

Gene ID	Log2 Fold Change	p-adj	Control CPM	Rad CPM
Vsig10	0.93	3.71×10^{-2}	35.97	68.61
Adm	0.93	1.77×10^{-2}	23.39	44.43
Ddc	0.91	3.37×10^{-2}	312.35	588.47
Ikzf1	0.91	1.72×10^{-2}	70.44	132.00
Slc18a2	0.90	3.73×10^{-2}	248.80	464.53
Plau	0.89	1.64×10^{-2}	38.13	70.61
Col22a1	0.87	4.76×10^{-3}	159.58	291.52
Traf1	0.86	4.32×10^{-2}	39.14	71.07
Hydin	0.86	4.84×10^{-2}	34.75	63.10
Icam1	0.86	3.60×10^{-2}	41.40	75.07
Arid5a	0.85	1.45×10^{-2}	35.34	63.73
Ntn1	0.84	3.30×10^{-6}	1,403.72	2,521.16
Sema3b	0.81	1.01×10^{-2}	93.79	163.92
Prss35	0.74	3.07×10^{-2}	112.85	188.69
Dysf	0.72	3.00×10^{-2}	84.73	139.63
Ifitm3	0.72	6.99×10^{-4}	96.15	158.44
Sulf1	0.71	1.00×10^{-3}	438.63	717.07
Tll2	0.71	1.74×10^{-2}	35.73	58.39
Ephx1	0.71	1.13×10^{-5}	105.23	171.76
Cldn1	0.70	1.66×10^{-2}	56.44	91.83
Cdca3	0.70	3.14×10^{-3}	69.96	113.76
Sardh	0.70	8.30×10^{-3}	152.84	247.75
Pax5	0.68	5.43×10^{-4}	1,054.66	1,685.89
Etl4	0.68	3.03×10^{-2}	136.38	217.90
Ddx58	0.67	3.45×10^{-2}	58.36	93.12
Plk1	0.64	1.68×10^{-2}	84.98	132.83
Pros1	0.63	1.28×10^{-3}	120.90	187.09
Nkx6-1	0.62	4.94×10^{-2}	97.83	150.86
Dnah6	0.61	2.39×10^{-2}	147.30	225.23
P4ha3	0.61	2.34×10^{-2}	184.78	281.93
Slc25a37	0.61	1.61×10^{-2}	237.51	361.79
Etv1	-0.61	2.00×10^{-3}	366.77	240.33
Epop	-0.62	3.39×10^{-3}	135.33	88.10
Slc14a2	-0.62	4.35×10^{-2}	131.38	85.20
Gm18736	-0.64	4.40×10^{-2}	45.02	28.91
Dmbx1	-0.65	2.12×10^{-10}	450.69	287.76
Pcdh20	-0.67	2.31×10^{-5}	211.66	132.71
Egfem1	-0.70	3.31×10^{-2}	60.65	37.30
Ighm	-1.00	1.30×10^{-2}	34.36	17.20

Gene ID	Log2 Fold Change	p-adj	Control CPM	Rad CPM
Sp5	-1.06	1.86×10^{-14}	529.68	253.47
Mettl11b	-1.33	3.11×10^{-2}	16.83	6.68
P2ry12	-1.37	1.76×10^{-11}	85.89	33.31
Crybb1	-1.46	3.92×10^{-4}	33.54	12.16
Siglech	-2.20	1.28×10^{-3}	24.99	5.45
Ttr	-4.00	2.03×10^{-2}	856.07	53.41

Supplementary Table S7: Differentially expressed genes in the female midbrain following gestational radiation exposure.

All 16 genes differentially expressed between control and gestationally irradiated female midbrain (n = 8 per sex per treatment group, one fetus per sex per dam; sham: 8 dams, irradiated: 9 dams), identified by DESeq2 as log2 fold change ≥ 0.6 or ≤ - 0.6 with a Benjamini-Hochberg false discovery rate (FDR) adjusted p-value (p-adj) <0.05. Genes are ranked by descending log2 fold change, where positive values indicate increased expression relative to control (14 upregulated, 2 downregulated). Control CPM and Rad CPM are mean normalized counts per million for each group.

Gene ID	Log2 Fold Change	p-adj	Control CPM	Rad CPM
Eda2r	2.15	1.32×10^{-7}	11.50	51.02
Agbl2	2.08	2.64×10^{-2}	4.91	20.74
Gm37795	1.99	1.02×10^{-2}	6.57	26.12
Pqlc3	1.96	2.79×10^{-4}	7.01	27.18
Siglec1	1.90	6.58×10^{-3}	10.19	38.11
Lyz2	1.71	1.66×10^{-7}	83.74	273.59
Bst2	1.60	5.75×10^{-4}	36.50	110.62
Cd84	1.48	3.42×10^{-3}	9.15	25.45
Mapkapk3	1.14	3.91×10^{-3}	23.09	50.94
Psrc1	1.13	7.21×10^{-3}	52.04	114.12
Icam1	1.00	6.94×10^{-3}	41.51	82.97
Ephx1	0.92	5.24×10^{-8}	106.52	201.17
Gstt2	0.90	5.75×10^{-4}	41.45	77.54
Dglucy	0.64	3.97×10^{-2}	56.20	87.82
Csf1r	-0.69	1.68×10^{-2}	486.05	300.35
P2ry12	-1.06	5.75×10^{-4}	64.86	31.02

Supplementary Table S8: Differentially expressed genes in the male hindbrain following gestational radiation exposure.

All 53 genes differentially expressed between control and gestationally irradiated male hindbrain (n = 8 per sex per treatment group, one fetus per sex per dam; sham: 8 dams, irradiated: 9 dams), identified by DESeq2 as log2 fold change ≥ 0.6 or ≤ - 0.6 with a Benjamini-Hochberg false discovery rate (FDR) adjusted p-value (p-adj) <0.05. Genes are ranked by descending log2 fold change, where positive values indicate increased expression relative to control (13 upregulated, 40 downregulated). Control CPM and Rad CPM are mean normalized counts per million for each group.

Gene ID	Log2 Fold Change	p-adj	Control CPM	Rad CPM
Flg	5.09	9.60×10^{-4}	2.45	83.44
Krt5	4.45	6.04×10^{-3}	3.39	74.05
Gpnmb	2.11	9.40×10^{-5}	7.32	31.59
Siglec1	1.59	9.40×10^{-5}	14.88	44.72
Gm37795	1.57	1.16×10^{-6}	12.76	37.86
Tnfrsf10b	1.16	5.14×10^{-4}	16.15	36.05
Lyz2	1.06	7.74×10^{-4}	98.91	206.82
Col19a1	0.88	1.40×10^{-3}	29.84	54.81
Eda2r	0.87	1.13×10^{-2}	38.53	70.33
Bst2	0.83	5.48×10^{-3}	52.54	93.30
Mecom	0.66	6.99×10^{-3}	123.83	196.02
Prrxl1	0.62	2.10×10^{-2}	218.46	335.91
Cfap54	0.62	2.01×10^{-2}	127.28	195.53
Cnksr3	-0.60	3.92×10^{-4}	154.23	101.49
En2	-0.61	2.10×10^{-3}	712.58	468.10
Zbtb18	-0.62	1.16×10^{-6}	1,770.77	1,152.58
Bcas1	-0.66	7.68×10^{-6}	310.68	197.17
Pax6	-0.66	7.49×10^{-9}	916.72	581.40
Sp5	-0.67	3.20×10^{-2}	157.75	98.88
Eomes	-0.70	9.30×10^{-3}	272.98	167.73
Ptx3	-0.71	5.05×10^{-3}	83.45	51.17
Ndp	-0.72	4.58×10^{-4}	92.72	56.26
Scrg1	-0.76	9.46×10^{-4}	81.70	48.25
Plxnb3	-0.76	5.50×10^{-4}	83.70	49.38
Csf1r	-0.77	3.32×10^{-6}	539.09	317.00
Cx3cr1	-0.78	7.71×10^{-3}	165.55	96.66
Plp1	-0.78	3.09×10^{-2}	288.14	168.18
Crlf1	-0.80	1.91×10^{-2}	70.77	40.78
Barhl1	-0.80	6.35×10^{-8}	1,107.70	634.41
St18	-0.81	3.00×10^{-3}	263.81	150.36
Gpr17	-0.85	1.98×10^{-15}	906.05	503.09

Gene ID	Log2 Fold Change	p-adj	Control CPM	Rad CPM
Gpr34	-0.88	1.92×10^{-2}	34.01	18.43
Galnt15	-0.90	5.97×10^{-6}	155.04	83.33
Mbp	-0.92	7.49×10^{-9}	1,312.66	693.81
Fgf5	-0.97	3.55×10^{-2}	38.53	19.73
Cd163	-0.99	4.23×10^{-5}	67.73	34.11
Gpr37l1	-1.05	8.14×10^{-3}	123.41	59.67
Lims2	-1.08	5.19×10^{-4}	72.59	34.42
Ucma	-1.15	3.03×10^{-2}	26.08	11.78
Ighm	-1.16	2.36×10^{-3}	34.45	15.43
Tcf7l2	-1.16	1.38×10^{-3}	915.32	408.64
P2ry12	-1.23	3.43×10^{-9}	103.14	44.01
Fat2	-1.47	1.47×10^{-16}	444.30	160.68
Siglech	-1.53	2.93×10^{-2}	18.16	6.30
CT010467.1	-1.67	2.61×10^{-3}	309.30	97.02
S100a9	-1.71	4.95×10^{-3}	31.01	9.50
S100a8	-1.93	8.63×10^{-3}	18.97	4.98
Serpinb8	-2.08	5.27×10^{-3}	36.59	8.65
Lcn2	-2.21	9.30×10^{-3}	17.41	3.76
Crybb1	-2.26	3.36×10^{-8}	39.40	8.24
Ngp	-2.33	1.46×10^{-5}	35.89	7.16
Neu4	-2.34	1.58×10^{-5}	21.69	4.29
Mobp	-5.10	5.18×10^{-11}	34.32	1.00

Supplementary Table S9: Differentially expressed genes in the female hindbrain following gestational radiation exposure.

All 38 genes differentially expressed between control and gestationally irradiated female hindbrain (n = 8 per sex per treatment group, one fetus per sex per dam; sham: 8 dams, irradiated: 9 dams), identified by DESeq2 as log2 fold change ≥ 0.6 or ≤ - 0.6 with a Benjamini-Hochberg false discovery rate (FDR) adjusted p-value (p-adj) <0.05. Genes are ranked by descending log2 fold change, where positive values indicate increased expression relative to control (8 upregulated, 30 downregulated). Control CPM and Rad CPM are mean normalized counts per million for each group.

**Gene ID`**	**Log2 Fold Change**	**p-adj**	**Control CPM**	**Rad CPM**
Gpnmb	2.24	8.17×10^{-4}	4.38	20.74
Oasl2	1.33	3.18×10^{-2}	26.26	65.88
Siglec1	1.25	3.33×10^{-3}	14.96	35.65
Ano3	1.20	5.37×10^{-4}	22.72	52.17
Eda2r	1.17	3.43×10^{-5}	28.58	64.15
Tlr3	0.92	1.43×10^{-2}	25.32	48.06
Tnfrsf10b	0.88	2.42×10^{-2}	19.18	35.37
Lgals3bp	0.77	6.64×10^{-3}	87.00	148.36
Barhl1	-0.61	1.98×10^{-3}	740.13	483.26
Zic5	-0.65	1.06×10^{-2}	549.61	351.21
Zbtb18	-0.65	1.34×10^{-5}	1,336.31	850.73
Neurod2	-0.66	1.98×10^{-7}	739.57	467.86
Mag	-0.70	1.06×10^{-2}	103.37	63.56
Plp1	-0.73	1.06×10^{-2}	284.48	171.60
Ptx3	-0.73	1.06×10^{-2}	61.72	37.09
Pax6	-0.75	1.01×10^{-10}	742.89	442.83
Bcas1	-0.75	5.37×10^{-4}	269.88	160.58
Gpr17	-0.75	6.85×10^{-5}	786.17	467.05
Neurod1	-0.76	3.43×10^{-13}	704.06	416.54
Prdm8	-0.80	4.12×10^{-13}	490.20	280.79
Mbp	-0.84	5.37×10^{-4}	1,173.76	655.65
Cd163	-0.88	1.89×10^{-2}	55.22	30.08
Plxnb3	-0.88	1.84×10^{-3}	78.79	42.79
Sp5	-0.92	2.82×10^{-4}	124.24	65.53
Csf1r	-0.95	1.17×10^{-29}	494.54	256.38
Ighm	-0.96	2.23×10^{-2}	35.58	18.23
Mybpc1	-1.03	3.85×10^{-5}	105.35	51.49
Il16	-1.30	1.27×10^{-2}	27.41	11.16
Myoc	-1.31	1.19×10^{-2}	20.94	8.46
Eomes	-1.36	4.77×10^{-10}	215.64	84.14
Fgf5	-1.39	1.95×10^{-4}	27.99	10.70

**Gene ID`**	**Log2 Fold Change**	**p-adj**	**Control CPM**	**Rad CPM**
P2ry12	-1.40	6.30×10^{-9}	99.02	37.49
Fat2	-1.52	4.34×10^{-18}	326.97	114.30
Crybb1	-1.73	4.85×10^{-7}	45.66	13.79
Samd3	-1.89	5.37×10^{-4}	15.81	4.26
S100a9	-1.99	2.40×10^{-2}	24.57	6.18
Ngp	-2.47	1.25×10^{-2}	21.09	3.81
Mobp	-4.36	1.76×10^{-3}	27.12	1.32

***Supplementary Table S10: Comprehensive list of DEGs identified by RNA-Seq across the telencephalon, diencephalon, midbrain, and hindbrain in BALB/c mice**.*

The table includes region-specific and brain region overlapping DEGs between the sexes (n = 8 per sex per treatment group, one fetus per sex per dam; sham: 8 dams, irradiated: 9 dams). DEGs were defined by a fold change of ≥ 1.5 or ≤ - 1.5 and an adjusted p-value of < 0.05.

Brain Regions	Total # of Genes	Genes
Male Telencephalon, Female Telencephalon, Male Diencephalon, Female Diencephalon, Male Midbrain, Female Midbrain, Male Hindbrain, Female Hindbrain	3	*P2ry12, Siglec1, Eda2r*
Male Telencephalon, Female Telencephalon, Male Diencephalon, Female Diencephalon, Male Midbrain, Male Hindbrain, Female Hindbrain	2	*Crybb1, Gpnmb*
Male Telencephalon, Female Telencephalon, Male Diencephalon, Female Diencephalon, Male Midbrain, Female Midbrain, Male Hindbrain	1	*Lyz2*
Male Telencephalon, Female Telencephalon, Female Diencephalon, Male Midbrain, Female Midbrain, Male Hindbrain	1	*Bst2*
Male Telencephalon, Female Telencephalon, Male Diencephalon, Male Midbrain, Male Hindbrain, Female Hindbrain	1	*Ighm*
Male Telencephalon, Female Telencephalon, Female Diencephalon,	1	*Tnfrsf10b*

Brain Regions	Total # of Genes	Genes
Male Hindbrain, Female Hindbrain		
Male Telencephalon, Female Telencephalon, Female Diencephalon, Male Midbrain, Female Midbrain	1	*Icam1*
Male Telencephalon, Female Telencephalon, Male Diencephalon, Male Midbrain, Female Midbrain	1	*Ephx1*
Male Telencephalon, Female Telencephalon, Male Midbrain, Male Hindbrain, Female Hindbrain	1	*Sp5*
Male Telencephalon, Female Telencephalon, Male Diencephalon, Female Diencephalon	2	*Ms4a6d, Lgals9*
Male Telencephalon, Female Telencephalon, Female Diencephalon, Male Midbrain	2	*Ifit1, Ifitm3*
Male Telencephalon, Male Diencephalon, Female Diencephalon, Female Midbrain	1	*Psrc1*
Male Telencephalon, Female Telencephalon, Male Hindbrain, Female Hindbrain	3	*Fat2, Plxnb3, Zbtb18*
Male Telencephalon, Female Telencephalon, Male Diencephalon, Male Midbrain	2	*Plau, Rsad2*
Male Telencephalon, Female Telencephalon, Male Hindbrain, Female Hindbrain	1	*Crlf1*
Male Telencephalon, Female Telencephalon,	2	*Mapkapk3, Pqlc3*

Brain Regions	Total # of Genes	Genes
Male Midbrain, Female Midbrain		
Male Telencephalon, Female Telencephalon, Female Diencephalon	16	*Gbp7, Ms4a6c, B2m, Msr1, Irgm1, Lyve1, Gbp9, Adamts12, F13a1, Fam114a1, Edn3, Slc38a4, Dnah7b, Stab1, Parp14, Nfe2l2*
Male Telencephalon, Female Telencephalon, Male Diencephalon	4	*Tor3a, Parp12, Phlda3, Ifit3*
Male Telencephalon, Female Telencephalon, Female Hindbrain	3	*Samd3, Oasl2, Lgals3bp*
Male Telencephalon, Female Telencephalon, Female Midbrain	1	*Cd84*
Male Telencephalon, Female Telencephalon, Male Midbrain	7	*Ikzf1, Pcdh20, Ddx58, Sulf1, Slc25a37, Ttr, Pidd1*
Male Telencephalon, Female Diencephalon, Male Midbrain	1	*Gm47761*
Male Telencephalon, Male Hindbrain, Female Hindbrain	1	*Pax6*
Female Midbrain, Male Hindbrain, Female Hindbrain	1	*Csf1r*
Male Diencephalon, Male Midbrain, Male Hindbrain	1	*Siglech*
Male Midbrain, Female Midbrain, Male Hindbrain	1	*Gm37795*
Female Telencephalon, Female Diencephalon	1	*Tmem252*
Male Telencephalon, Female Telencephalon	569	*Scn5a, Islr, Calcr, 1700001L05Rik, Wnk4, Kcns3, Cpz, Irf5, Steap3, Cd274, Frem1, Gm6548, 4930539E08Rik, Pitpnm3, Ebf3, Gm37090, Bmp3, Slc4a5, Kcnj2, Ovgp1, C1qa, Col8a2, Gm8909, Tlr2, Sult1a1, Dlx6os2, Tap2, Gli1, Mst1r, Ifitm1, Nid2, Dock6, Krt222, Ptgdr, Dab2, Cdkn3, Tfap2b, Mrc2, Clec14a, Hapln4, Nts, Fgd6, Rnf182, Mpped1, Col6a1, Cpne7, Sidt1, mt-Nd1, Snai2, Tfpi, Slc2a4rg-*

Brain Regions	Total # of Genes	Genes
		ps, Msx1, Thbd, Tnfrsf1b, Gm17750, A2m, Ms4a7, Slc12a5, Efhc1, Adcy7, Ankle1, C3ar1, Cfap46, Ptpn14, Mertk, Lrrc32, Gm49027, Lepr, Crispld2, Cd38, Phldb2, Sesn2, Dok3, Reln, Ifi207, Ghr, Tmem106a, Chrna4, Hacd4, Prokr2, Myo1f, Bicc1, Clec4n, Gm44250, Atp1a2, Dsp, Col4a5, Col18a1, Tmem72, A4galt, 9330162G02Rik, Ddit4l, Itgax, Syne1, Arhgap9, Gm1818, Cyth4, Mrgprf, Gjb2, Gm14827, S1pr5, Ccno, Fcgrt, Aox3, Epor, Six5, Tmem191c, Apobec1, Tac2, Ptchd4, Akr1b10, Arsg, Gbp2, Gm28151, Xaf1, Folr2, Themis2, Hlf, Kcne2, Hpse, Sfta3-ps, Gm6145, Col24a1, Shfl, Pfkp, Trp73, Id2, Slc22a6, Rtp4, Trabd2b, Bhlhe22, 9430064I24Rik, Lcp1, Col23a1, Piezo1, AC151602.1, Lix1, Slfn2, Trem2, Calhm2, A330023F24Rik, Myof, Svep1, P2ry6, Ryr2, Slc2a12, Spta1, Gm36995, Tap1, Col16a1, BC022960, Ifi44, Acss3, Emilin1, Gdf9, Gm20605, Fn1, Firre, C4b, Foxc2, Lrrc25, AI662270, Whrn, Gm43302, Col6a2, E330034L11Rik, Rhbdl3, Igf2os, Aldh1a1, Alas2, Msx3, Cyp1b1, Gm40841, Tnfrsf23, Gucy1a1, Serpind1, Gm42778, Gm49083, C78859, Lox, Ccr5, Mmp19, Gm10134, Slc22a2, Ntf3, Hspb8, Ppp1r3b, Gm26672, Aifm3, Ssc5d, Gja1, Gypc, Myh9, Ebf2, Lime1, Gm37249, Mrc1, Helz2, Nupr1, Tgfbi, Ildr2, Tns2, Lamc3, Rgs6, Gpr21, Tmem74, Gm20559, Mvp, Cpeb1, Tgm2, 1110002E22Rik, AB124611, Ppfia4, Magel2, Slc7a11, Tnfrsf11a, Arap1, Cspg4, Osr1, Mov10, Gm4081, Creb3l1, Igf2, Oas1a, Mrap2, Syt6, Lama4, Itgal, F5, Mef2c, Nhsl2, Bmp6, Tmem140, H2-K1, Arhgap30, Car10, Alx4, Lhcgr, Msra, Cd300a, Amy1, Snhg11, Cregl, Hmox1, Dock2, Ripor3, Atoh8, Tifab, Muc1, Satb2, Acad10, Ifi202b, Grk3, Car14, Ace, Hif3a, Ccdc74a, Tbc1d2, H2-T24, Rrbp1, Lhx5, Nfkb2, Tpsb2, Fign, Grin2d, Slc37a2, Mfrp, Has2, Slc6a20a, Rbm47, Ranbp3l, Rin3, Tcirg1, Ptgds, Kcnt1, Slc13a4, Sema7a, 2900040C04Rik, Ugt1a7c, Tmsb15l, Ssh3, Svil, Grp, Cldn11, Cacna2d2, Gpc3, Pou3f1, C1ra, Flt3l, Mmp2, Cdk18, Zfp469, Cmbl, Ikbke, Jade2, L3mbtl1, Adamts9, Samd9l, Cd24a, Rnf213, Uba7, Gm11127, Lrp1, Cdkn1a, Cfap126, Stap2, Cma1, Tfcp2l1, Itih2, Tlr13, Col4a2, Lhx1, Nid1, Lpp, Adgra2, Nr4a3, Ssbp2, Gbp4, Hspg2, Slc12a7, Slc16a3, Irgm2,

Brain Regions	Total # of Genes	Genes
		Trank1, Lacc1, Krt8, Axin2, Fcna, Fes, Smad6, Gbx1, Adra1b, Ror1, Prodh, Cldn3, Ncf2, Dsc3, Slc8b1, Mapk15, Sned1, 9130024F11Rik, Aox4, Lef1, Il17rc, Sh3d21, Lin28b, Trpv4, Stat6, Gm38317, Ifi27, Fgfbp1, Ccdc187, Fam20a, Itih5, Gm5089, Parp10, Cybb, Rasa4, Slc15a3, Ugt1a6a, Tcea1-ps1, Cacna1a, Kank2, H2-D1, Mab21l1, Laptm5, Zwilch, Ddr2, Trpm2, Irf1, Sphk1, Bmp5, Oxtr, Aebp1, Gm4316, Minar1, Gm42979, Ndnf, Unc93b1, Ifi44l, H19, Enpp1, Ifi203, Synj2, Greb1, Unc5b, Gm49593, Cfh, Il33, Plec, Baiap3, Colec12, Tradd, Bhlhe41, Fcer1g, Usp43, Dok5, Zfp92, Hdc, Rcsd1, Casq1, Ctsc, Rgs14, Rhpn1, Lgals3, Ms4a6b, Aox1, H2-Q6, Gucy1b1, Mir99ahg, Sntb1, Irf8, Mpeg1, Lama3, Evc, Barhl2, Adrb2, Ptprc, Wnt6, Apoe, Kifc2, Hspa1a, Vwf, 8430422M14Rik, Col4a1, A830009L08Rik, Parp9, Prdm6, Fcgr3, Vnn1, Igsf6, Foxd1, Gm44168, Bmp4, Nhlh2, Col4a3, Onecut2, Adamts15, Tgfbr3, Itga10, Slc6a13, Plekha7, Epha10, Pik3ap1, Lilrb4a, Gjb6, Rxfp1, 5031434O11Rik, Ldb2, Gm17396, H2-Q4, Ly6g6e, Rgs11, Pdlim3, Man2b2, Pear1, Fcgr2b, Dtx3l, Neat1, Frrs1, Susd2, Slc16a11, Wnt4, Gucy2e, Prune2, Cp, Ahnak2, Adam12, AU020206, Fmod, Mirg, Ogn, Klhdc7a, Cox6b2, Htr2c, Nhs, Wdr86, Car12, Col27a1, Isg15, Mroh7, Cdh3, Nlrc3, Angptl2, Fezf1, Rbm20, BC067074, Gm11639, Kcnc2, Itpr3, Rmi2, 9130017K11Rik, Tmem37, A230103L15Rik, Foxc1, Trim66, Hgf, Ncf1, Neurod6, Plekhd1, Fkbp10, Cped1, Usp18, Ccbe1, Adamts19, Ugt1a6b, 9930014A18Rik, Efcc1, Peg3, Rreb1, Gm20045, Cntnap1, Acvr1, Ier3, 2900009J06Rik, Aldh1a2, Insrr, Kndc1, Lpl, Zc3hav1, Bmp7, Ermn, Cxcl12, Aspm, Tpcn2, Kcnj13, Crym, Rac2, A230057D06Rik, Slc6a7, Igtp, Ccdc88b, Proca1, Tonsl, Rassf4, Eif2ak2, Nbeal2, Irak4, Gm50322, Tbx18, 9930111J21Rik2, Clic6, Olfml1, Prr32, Abca9, Cfp, Hspb3, Irf7, P2rx5, Gm43654, Samd11, Arhgap45, Rhbdf2, Cd68, Apod, Vwa5b2, Lipg, Dcxr, Syt4, Il10ra, Ahnak, Rmst, Krt18, Lrrk1, Cep164, Gpr6, Fbxo32, Arhgap19, Obscn, Ecel1
Male Telencephalon, Female Diencephalon	6	*Plekha2, Slfn9, Ehd2, Slc16a12, Kif2c, Cck*
Female Telencephalon, Male Diencephalon	4	*Phospho1, Vsir, Atp13a5, P2rx7*

Brain Regions	Total # of Genes	Genes
Female Telencephalon, Female Hindbrain	2	*Neurod1, Neurod2*
Male Diencephalon, Female Diencephalon	1	*Kcng4*
Male Telencephalon, Female Hindbrain	1	*Zic5*
Female Telencephalon, Male Midbrain	7	*Sardh, Tll2, Slc6a4, Pros1, Sema3b, Slc18a2, Adm*
Male Telencephalon, Female Midbrain	2	*Agbl2, Gstt2*
Male Telencephalon, Male Hindbrain	4	*Gpr34, Col19a1, Mecom, Tcf7l2*
Male Telencephalon, Male Midbrain	6	*Cdca3, Egfem1, Gm42047, Etl4, Ttc21a, Hydin*
Male Hindbrain, Female Hindbrain	12	*Gpr17, Plp1, Bcas1, Fgf5, Barhl1, Mobp, S100a9, Eomes, Cd163, Mbp, Ptx3, Ngp*
Female Telencephalon	604	*Tnfrsf13b, Wdr63, Tmod1, Insm2, Syt2, Zfp853, Tmco4, Myrip, Ifi30, A330084C13Rik, Gm6501, H2-Q5, Slc41a3, Fignl2, Tekt5, Lgi3, Gm12198, Dcn, Rtkn, Gm10145, Slco1a4, Irs4, Mlip, Srl, Immp2l, Necab2, Dnaic1, Trpc6, Rsph10b, Rpl9-ps7, Rspo3, Grid1, Ly6e, Mynn, Neb, Spsb2, Grem2, Skor1, Gm27199, Nmi, Tmem63a, Olfml2b, Kcnh5, Crabp2, Sv2c, Tspan17, Col9a2, Timp1, Klc3, Arc, Gm28050, 4930594M22Rik, Gcnt4, Zeb2, Npbwr1, Cpxm1, Cd33, B130024G19Rik, Trim30a, Pla2g7, Tmc8, Gpr50, Pdlim2, Pf4, Pir, Grik1, C2cd2, Gm43719, Plekha4, Sdk1, Gm37607, Cdc42ep5, Zap70, Adamts16, Erap1, Gm42918, Gm8399, Nxnl2, Fgf2, Fam184b, Nfix, Ltf, Wfs1, Ctss, Mx2, Gm8822, Ehbp1l1, Alx3, Cx3cl1, Plaat1, Filip1, Kctd11, Syt17, Dnm3os, Nckap5, Fastkd2, Gm26801, Tep1, Poc5, Lmntd1, Prlr, Slc5a5, Gm41760, Gm29562, Gabrq, Smad9, Hrh1, Ankrd63, Itgb2, Hlx, Arl13b, Tnip2, Lamc1, Dct, Agtrap, Camk2d, Icam5, Pon3, Aff2, Tmem132e, Dgkk, Cyp26b1, Tmprss7, Psmb8, Vwc2l, Slc16a8, Slc7a10, Brca2, Aste1, Iigp1, Gas6, Dhx58, 2010300C02Rik, Grm1, Smoc2, Abi3bp, Pde8a, Me3, Elfn1, Fut4, Aspn, Sostdc1, Klhdc1, Panx2, Slc6a9, Gm10146, Arhgef16, Gm4724, Parp4, Htr1d, Tgfb1, Vamp5, Calb1, Il1rapl2, Paox, C1qb, Runx1, Gm42894, Capg, Cyb561, H6pd, Fmnl1, Htr7, Sh3bp1, Kcnh6, Renbp, Gprc5c, Retsat, Clmn, Glp1r,*

Brain Regions	Total # of Genes	Genes
		Gbgt1, Odf3b, Myo7a, Gm6158, Slc24a4, Pla2g3, Ppp1r3g, Tnfaip2, Spag17, Col13a1, Cdkn1b, Ccng2, Alk, Gm37494, Mrs2, Enpep, Camk2a, Kif19a, Adgra1, Clec3b, Itprid1, Skap1, Gfra2, Optc, Lrp1b, Mxra8, Tmem151a, 4930447C04Rik, Gpr157, Shh, Stac, Asb2, Plek, Fcgr1, Tacr1, Uhmk1, Igfbp5, C2, Gm30382, Amz1, Deup1, Gpr183, Tnfaip8l2, Gxylt2, Tbx2, Serpinb9, Mc5r, Vtn, Gal3st1, Plscr2, AI606473, Nphs2, Hpcal1, Cgnl1, Serhl, Fam49a, Hck, Spi1, Dkkl1, Gm48161, Ninj1, Ifi35, Exoc3l4, Igsf1, Abracl, E130102H24Rik, Calml4, Cd55, E430018J23Rik, Anpep, Tor4a, Clec10a, Prima1, S100b, Ndst4, AW551984, Matn2, Sema3c, D630023F18Rik, Gpr61, Gm3055, Vax1, A330102I10Rik, Nrip3, Fhit, Sorcs3, Sp110, Nrsn2, Tnfrsf8, Cdh22, Ccdc158, Gm10499, C5ar1, Lhx8, Zfp953, Rasl10a, Hbb-bt, Gusb, Gm29260, Tiam2, Dera, Zfp874a, 2700033N17Rik, Srpx2, Sgsh, Ccr1, Gm44675, Tac1, Slc25a47, Gm44562, Slc18a3, Adra2a, Tex15, Epha4, Smpdl3b, Piezo2, C1ql2, Gabrg1, Dnase1l1, Creg2, Gbp3, Fxyd7, Tmem132d, Kl, Il34, Pde8b, Apobec2, Map2k6, Adh7, Mterf1a, Klhl33, Atp10b, Hmgcs2, Glra3, Dlec1, Faap24, Zmym1, Trim21, Selenbp1, Angptl4, Pycr1, Foxp1, Pkp2, Inpp5j, Npr1, Col26a1, Iqcd, Gadd45b, Fbxo2, Nudcd1, Slc1a6, Cntnap5a, Gucy1a2, Igsf10, Lrp8os2, Plekhf1, Spa17, Sdc4, Gabre, BC025920, Foxd2, Il4ra, Gulp1, Amn, Plcg2, Impg2, Lbhd2, Zim1, Zfp879, Sash3, Lnx1, Kcna2, Fam163b, 9530082P21Rik, Aqp4, Vasn, Atp6v0d2, Lrtm2, Bnc2, Neurl1a, Fezf2, Tril, Thsd7b, Tmem117, Dnase2a, Moxd1, Tuba4a, Hist2h2aa1, Upp2, Gpc5, Arhgap27, Man2a1, Gm5171, Kcnj16, Slc30a3, Spata33, Tacr3, Ecm1, Sowaha, Adam15, Egr4, Slc22a3, Rarres2, Ankrd35, Plcxd1, C1qc, Tmie, Slc9a9, Pcdhgc4, Cyp4v3, Thy1, Nptxr, Dok2, Gdf5, Vat1l, Tmc6, Ache, Gpr101, Ggt5, Tfap2d, Nell1, Mgmt, Gm27021, Plek2, Lrrc3b, Gm45311, Ramp1, Entpd1, A930017M01Rik, Fam43a, Manba, Plxnd1, Nr1h3, Bin2, Nipal3, Spp1, Grin3a, Tgfbr1, Nkx2-3, Sfmbt2, Hap1, Rasgef1a, Pdzd2, Col6a6, Erbb3, Itga8, Arhgap22, Fam129a, Il1rl1, Il1rn, Gja5, Cmklr1, Map7d2, Nkapl, Adamts2, Gm42715, Gbp10, Pcolce, Resp18, Lhx1os, Adamtsl4, Pdgfd, Pamr1, Gm5468,

Brain Regions	Total # of Genes	Genes
		Ifi27l2a, Zan, Gab3, Pdxk, Rab39, Iqca, Cdh18, Piwil2, Bnip3l-ps, Hnmt, Ackr1, Plk5, Hsd3b7, Slc16a7, Smim1, Mycn, Gm38124, Gm10516, Kcnc4, Drc7, Adcy8, Llgl2, Gm46409, Dnm1, Wnt9a, Megf6, Ctxn2, Mkx, Hic1, Gm10419, Gbp2b, Rasgrp1, Tmem268, Tmem91, Lmo1, Rab32, 3110045C21Rik, Galns, Pdgfra, Rassf5, Cdh17, Rabepk, Lbp, Sec14l2, Gm19410, Rspo2, Rasd2, Trpm5, Slc17a7, Cdh23, Lhx2, Masp1, Dact2, Atp6ap1l, Pld2, Pacsin1, Gtdc1, Abca4, Cd14, Hist1h1e, Lin7b, Klhl41, Abcc3, Tmbim1, Slc17a8, Gm14176, Gm17259, Bcan, Slc26a8, Prkcb, Hebp1, Slc4a10, Gm16485, Nbl1, Eps8l2, Uncx, Slc7a3, Parm1, Car5b, Clec7a, Csrp2, Fads6, Gm7332, Cttnbp2, Gm2136, Gda, B3galt5, Tshr, Lynx1, Cd44, Pced1b, Gpld1, Mgll, Myh14, Trim13, Tm4sf5, Mmrn2, Rinl, Egfl7, Npffr1, Zfp719, Card10, Kank1, Lamb3, Ehd4, Thsd4, Eya2, Rcan2, Ptprn, Rasgef1b, B3gnt9, Efna4, Cyba, Smco3, Igfbpl1, 9430053O09Rik, Kcng2, Npy1r, Psmb9, Nqo2, Il6ra, Cfap70, Fastkd3, Sst, Tfr2, Slc24a2, Ccn2, Casq2, Pappa, Spag4, A2ml1, Gpsm3, Kcnk9, Asah2, Thrsp, Fsd2, Ntrk1, Sp100, Ifitm10, Heph, Rasal1, Pld6, Gpx3, Klhl35, Ddn, Bdkrb2, Cntn2, Zfp941, Adgrg6, Scg2, Cpq, Cfap100, Gm43517, Xpnpep2, Gm12063, Gm28661, Emx1, Dpp4, Nckap1l, Gm41662, Naprt, Gm38198, Lrrk2, Olfr1372-ps1, Chrdl1, Enpp2, Fzd10os, Pdzph1, H2-Q7, Cers3
Female Diencephalon	13	*Arhgap29, Tubb6, Synpo2l, Prc1, Pmp22, Rax, Dusp27, Lamb1, Gpr179, Calcrl, Gm10273, Col11a2, Flnc*
Male Telencephalon	833	*Gm47798, Wnt11, Gm2824, Hebp2, Tle2, Scrib, Gm3052, Ccdc173, Slc35g1, Nsun7, Npy, Zfyve26, Abcd4, Atp13a4, Adamts10, Gdpd2, Tspoap1, Tchh, Trim56, Gm4890, Rad54l, Slc25a53, 1600023N17Rik, 3010001F23Rik, Mir124a-1hg, Oip5, Serpinb1b, Gm45441, Chek2, Gm43481, H2-M5, C4a, Pxdn, Jag1, Foxm1, Il3ra, Pabpc1l, 4930570G19Rik, Kcnu1, Adam33, Rrp1b, Vmp1, Nlrp6, Malat1, Rnaset2b, Trmt9b, 4933439C10Rik, Baz1a, Ptprs, Gm28035, Zfp558, Dnah11, Sigirr, Neurod4, Gm20342, Dnah9, Cmtm7, Lgals4, Gm47087, Snx7, Mir17hg, Cops9, Mdm4, 1700109H08Rik, Cct6a, Dot1l, Slc13a3, H3f3aos, Saysd1, Fancd2, Slc16a10,*

Brain Regions	Total # of Genes	Genes
		Gm17102, Csf2rb, BC005561, Arhgef5, Gm42798, Dhrs11, Thpo, Gm43549, Gtse1, Onecut3, B930086L07Rik, Zfhx3, Sfxn2, Arhgap24, Procr, Gm45110, Gm37139, Plekhh1, Stac3, Gm45250, Gm47113, Parp3, Gm13910, Gm1673, Ttf2, C030037D09Rik, Ints6l, Meg3, Vegfa, Ccdc162, Gm9774, Gm49405, Cdc25b, Efemp1, Rab7-ps1, Snx20, Maf1, Pou2f2, C130074G19Rik, Maskbp3, Gm37309, Fam227a, Gm42463, Hbb-bs, Ccnl2, Lrp4, Rps14, 5330431K02Rik, Ppargc1b, Eloc, Gm37018, Cks2, Cdca2, Tpp1, Srrm2, Gm10778, Ubr4, Gm48062, Gm38057, Gm44831, Gm12905, Cgas, Sim1, Eldr, Adcy4, Vwa5b1, Emp3, Pomc, Lama5, Per3, Vamp1, Coro7, Noxo1, Gm45169, Gm49322, Spag1, Lama1, Fanca, Gm37310, Gm33869, Gsdmd, Alpl, Dennd1c, 9530056E24Rik, Paxbp1, Gm37694, Gm10421, Txlnb, Kank4, Dmpk, Arhgef1, Spag5, Map4k2, Tgfbr3l, C130051F05Rik, Gm43858, Pard3b, Sox13, Nlrc5, Adora2a, Tent5c, Nktr, Gm10718, Tmem181c-ps, Rpl10a-ps1, Foxb1, Gm16464, A530041M06Rik, Gm45501, Gm44437, P3h2, Crb1, Ano1, Aspdh, Tpsab1, 4930518I15Rik, Aph1c, Zc3h7a, Esyt1, Uhrf1, Pnpla7, Ubap1l, Ccdc114, Nadsyn1, Appl2, Adra2b, Mink1, Tead3, Dna2, Cdh5, Snapc4, Gm40309, Kif14, Knl1, Tk1, Slc9a5, Rpl10-ps1, 2610035F20Rik, Efcab7, Nat8f4, Arhgap6, Tex9, Dhtkd1, Nip7, Gask1b, 9930017N22Rik, Gm37608, Gm14325, Olfml2a, A930024E05Rik, Vwa3a, Gm49502, Sec61g, Zdhhc1, Gm43759, mt-Co1, Catsperg1, Mrps23, Ulk3, Ybx2, Scd3, Acot9, Mien1, Cgn, Mtmr11, Grk4, Ticrr, Abcg5, C230057M02Rik, Trappc4, Cenpi, Gm48796, Gm11611, Mamdc4, Rpl18-ps2, 6430511E19Rik, Zfp759, Gm2999, Slc25a42, C1qtnf3, Robo3, Slc38a6, Gm12966, Phykpl, Vmn2r1, D930016D06Rik, Cyp4f13, Tram2, Hkdc1, Gm42992, Sema3g, P2rx4, Gm10240, Trpm3, Slitrk6, Kcnj5, Igf2bp1, AI480526, A430110C17Rik, Cfap65, A730011C13Rik, Vps13d, Slc19a2, Rfx2, Six4, Shisa7, Chrna7, AW822252, Smim26, Gm35339, Mbd6, Itga5, Doc2g, 4632427E13Rik, Ttll3, Elf4, Gm9954, Stard9, Tbxas1, Pmfbp1, Gm20390, Gli2, Hk3, Rgl2, Htr3a, Zgrf1, Gm37206, Abcc10, Pkn3, Gm37333, Pla2g6, Miat, Zmynd15, Prkd2, Kif15,

Brain Regions	Total # of Genes	Genes
		P4ha2, Ackr2, Kifc5b, Ccn4, Gm38190, Rpl9-ps6, Glb1l, Irf9, 5830487J09Rik, Plekhg4, Ift172, Mrvi1, Gm49194, Ano5, Chrd, Ankrd23, 2900052L18Rik, Aoc2, 4731419I09Rik, E330013P04Rik, Gm38043, Crocc, Ubxn11, Flna, Atad2, Sgo2a, Dnase1l2, Pla2g4b, Gm48485, Arhgef40, Gm29695, Gm42853, Rad51ap1, Gm50107, Arhgap17, Crip3, Bag3, Thsd1, Tanc1, Cdt1, Chchd1, Gm49883, Pld4, Cacng1, Gm4739, Ikzf4, Ager, Palb2, Gm19409, 2610037D02Rik, Gm29666, Ankrd16, Gm44433, Clasrp, Pyroxd2, Zmym6, Notch1, Gm17455, Cacna1g, Catsperg2, Elmo3, Itga11, Neil3, Mmp23, Hba-a2, Cdh1, Phyhd1, Lcp2, Zfp708, Syndig1, Ptpn18, Arglu1, Gm43389, Reck, Clcf1, Cspp1, Wdfy4, Gm44901, Gm43813, Col6a5, Ece2, Gm12992, Gm37893, Dse, Gm36936, A830073O21Rik, Mki67, Lmx1a, Eml6, Rarb, Tmem86b, Impa2, Cdc14b, Ttc25, Bgn, Nhlh1, Ifih1, Gm13436, Gm47283, Prox2, Cit, Carmil3, Copz2, Neurog2, Hcar1, C130023A14Rik, Cyp27a1, Gm2423, Rora, Gm10719, Rbp4, Gm43162, Fmn1, C130089K02Rik, Cdc25c, Gm45716, Gm43681, Eya1, Supt4a, Tha1, Med12, Cfap69, Tyrobp, Gm5628, Abhd14b, Tmc4, E030022I16Rik, Cdc14a, Rfx5, Snrpe, Cfap43, Stard8, Lrp2, Gpx4-ps2, Slfn5, Gm43099, AC107792.1, Syne3, Zfhx4, Slc16a6, Tomm6os, Tmem88, Onecut1, Plekha6, Gm45652, Sla, Ect2, Tmem200c, Gm17491, Gbp11, Cenpe, Ckap2l, Hba-a1, Gm19817, Fam228a, Tcf7l1, Ntng1, mt-Co3, Gm40847, Dmrta1, Prnd, Catsper2, Sgms2, Pabpc4l, Ermard, Prr11, Fgfr3, Ism2, Mc4r, Espl1, Gm21781, Ccdc3, Gm37584, Myo9b, Gm38253, Gm37254, Syt15, Pla2g4c, Hpgds, Prss53, Hells, Neurog1, Baiap2l2, Gca, Aspg, Gch1, 2600014E21Rik, Gm15328, Prxl2b, Gm44758, Rpl9-ps4, Tmprss5, Gm44769, Zbtb7b, Nrbp2, Abcc5, Rgl3, Lyl1, Dio2, Shroom4, Clspn, Ap1g2, Pdzd7, Mir100hg, Gm43483, Snrnp48, Csf1, Masp2, Gm3531, E2f8, 2700038G22Rik, Nfat5, Ccdc142, Gm43305, Hopx, Cntrl, Iqgap3, Pheta2, Csf2ra, Gli3, Casr, Kif18b, Gm4544, Sfi1, Eml5, AC099699.1, Pdlim1, Mitf, Aff1, Gm47573, Cep250, Pld1, Slc26a11, 5330434G04Rik, Clcn2, mt-Co2, Wdr90, Arhgap4, Mcub, 2900027M19Rik, Lamb2, Gm21982,

Brain Regions	Total # of Genes	Genes
		Pigl, Foxr2, Fbn2, Rtbdn, Gm28512, Col5a1, Igdcc3, Scn10a, Aoc3, Mrpl47, Gm14270, Clca3a1, Cep95, Plch2, A930004J17Rik, Unc80, Unc45b, Gm44286, Gm48700, Mrpl23, 6430590A07Rik, Gm38077, Gm27000, Gm42991, Acvr1c, Gm12940, Gm19040, Tmem35b, Csf2rb2, Csrnp1, Rin1, Hspe1, Gm29514, Podn, Psma6, Mindy4, Car13, P3h3, Cdc26, 4930556M19Rik, Gm26881, Pantr2, Srpx, Stxbp2, Il18bp, Scube2, BC024063, 9630028B13Rik, 45901, Mavs, Lrriq1, Gm28042, Ccdc194, Atp5l, mt-Nd5, AI661453, Kntc1, Ddx11, Gm45222, Alpk1, Gm50253, Il1rl2, 1700020D05Rik, Cnn2, Gmnc, Alox5ap, Gm8206, Gm49493, Ube2c, 2700029L08Rik, Man2c1, Zfp560, Tcaf2, Angpt2, Gm47260, Otof, Atp5e, Lhx9, Slc11a1, Abca8b, Gm3448, Aurkb, Gm44949, Nek10, Scarf2, Dlgap5, A930033H14Rik, Car4, Rock1, Gm47210, mt-Cytb, Acsf2, Anxa1, Snai1, 2610307P16Rik, Tnfrsf25, Sfrp5, Slc39a2, A330076H08Rik, Ppfibp2, Epb41l4b, Gm43951, Cenpf, Zfp366, Gm17383, Gabpb2, Trf, Gm21972, Recql4, Gm7694, Ebf4, D430036J16Rik, 2500002B13Rik, Npas4, Gm4117, Cpa3, Gm43336, Dock5, Dnajb3, Bora, Gm5577, Gm11578, Gm43080, Gm14150, Gm32444, Gm37606, Kmt2d, Tubgcp6, Arhgap25, Gm47033, Coro6, Dpp7, Ncapd2, Dgkq, Nfatc1, Cfap44, Mbnl2, Mis18bp1, Pml, Gm26901, Stc2, Myo19, Gm11266, Gm37019, Adam8, Gm8430, Xylb, Gigyf1, Fbxl12os, Zfp967, Lmo4, Rtel1, Col20a1, Gpr182, Fermt3, Gm3764, Lpo, D330041H03Rik, Wdr62, Hapln3, Fst, Pif1, Spaca6, Gm43300, Gm14391, Serping1, Glt28d2, Gm37226, Crispld1, Gm37084, Id3, Aifm2, Gm13563, Syde1, Card14, 1700014D04Rik, Gm47939, Gm10643, Hcls1, Dusp11, Rhbdf1, Clcn1, Pnisr, Naa80, Celsr1, Map7, Cln6, Gm45074, Gm43843, Gm49396, Ankrd24, Zfhx2, Tbcc, Ube2l6, Bub1b, 2900079G21Rik, Gm37928, Tert, D030055H07Rik, Fhod1, Rtl9, Ttc39a, Ccdc159, Elovl1, Gm6658, Lrrc45, Gm19938, Prox2os, Flt1, Gpt, Scara5, Grb10, Ska1, Exoc3l, Gm49599, Gm49759, Tirap, Itpr2, Plxna3, Cubn, Tfap2c, Gm42748, Itga2, Safb2, Gypa, Cxcl16, Stx3, Ushbp1, Akap8l, Mybl2, Fxyd5, Gm21887, D930036K23Rik, Gm43188, Col7a1, Reep4, Mfsd2b, mt-Nd4, Gm46378, Arhgef39, Incenp,

Brain Regions	Total # of Genes	Genes
		A530058N18Rik, Rflna, Myh11, Leng8, Hmmr, Gm49708, Gm45457, Cecr2, Kcp, Arsk, BC024139, Gm37274, AC154542.1, Cep192, Gm36963, Ucp2, Fgfbp3, Mkln1, Gm42664, Slc25a45, Adgre1, Folr1, BC002059, Plekhg1, Cyb561d1, Polq, Arid3a, E2f2, Syt12, Kcnq1ot1, Gm44597, Nfkbiz, Gm42971, Gm14292, Fyco1, Szt2, Slc22a21, Sox7, Ptch2, Rad54b, Jak3, AC132288.1, Slc4a11, Rnf207
Male Diencephalon	2	*Gm9780, Lncppara*
Female Hindbrain	7	*Mag, Prdm8, Il16, Tlr3, Mybpc1, Ano3, Myoc*
Female Midbrain	1	*Dglucy*
Male Hindbrain	19	*Gpr37l1, En2, Cx3cr1, Serpinb8, Lcn2, Galnt15, Cnksr3, Krt5, Ucma, Flg, Neu4, Prrxl1, Ndp, St18, Lims2, S100a8, CT010467.1, Cfap54, Scrg1*

Supplementary Table S11: Transcriptomic sequencing and alignment metrics for all brain-region samples.

Sequencing alignment metrics for all 128 RNA-seq libraries (tonsil, diencephalon, midbrain, and hindbrain); n = 8 per sex per treatment group, one fetus per sex per dam; sham: 8 dams, irradiated: 9 dams), as reported by the RNA-seq Alignment application (v. 2.0.2) (BaseSpace, Illumina Inc.) following alignment to the mouse reference genome (mm10/GRCm38) using STAR. Read length is shown for Read 1/Read 2. % Abundance is the percentage of reads mapping to abundant species (example., ribosomal and mitochondrial RNA); % Aligned is the percentage of reads aligned to the reference genome; Median CV Coverage Uniformity is the mean median coefficient of variation of transcript coverage (with the lower values indicating more uniform coverage); % Stranded is the percentage of reads with the strand orientation expected for the directional library.

Sample ID	Number of Reads	Read Length (bp)	% Abundant	% Aligned	Median CV Coverage Uniformity	% Stranded
TLab301	14,447,230	139/139	7.10%	74.85%	0.51	99.58%
TLab302	26,455,938	139/139	8.30%	74.56%	0.48	99.59%
TLab303	24,077,945	139/139	8.73%	74.30%	0.47	99.57%
TLab304	30,917,516	139/139	7.66%	74.00%	0.47	99.54%
TLab305	32,229,592	139/139	7.00%	75.41%	0.46	99.55%
TLab306	29,859,452	139/139	10.04%	72.49%	0.47	99.53%
TLab307	34,444,620	139/139	9.95%	73.32%	0.46	99.48%
TLab308	32,119,957	139/139	11.44%	73.39%	0.47	99.55%
TLab309	29,663,496	139/139	7.69%	73.97%	0.47	99.62%
TLab310	24,576,964	139/139	9.57%	71.21%	0.48	99.58%
TLab311	30,670,380	139/139	9.44%	71.35%	0.46	99.54%
TLab312	37,723,010	139/139	7.23%	71.81%	0.46	99.58%
TLab313	27,436,058	139/139	7.36%	71.10%	0.46	99.56%
TLab314	30,665,152	139/139	6.61%	70.90%	0.48	99.59%
TLab315	21,970,255	139/139	8.90%	71.12%	0.48	99.53%
TLab316	24,560,397	139/139	9.01%	74.25%	0.48	99.60%
TLab317	22,439,864	139/139	7.80%	73.00%	0.49	99.52%
TLab318	26,091,522	139/139	6.68%	72.83%	0.48	99.54%
TLab319	29,972,743	139/139	7.19%	73.11%	0.47	99.59%
TLab320	31,075,977	139/139	6.64%	72.02%	0.46	99.58%
TLab321	30,098,381	139/139	7.03%	70.68%	0.47	99.53%
TLab322	28,150,850	139/139	7.10%	69.36%	0.47	99.58%
TLab323	32,726,764	139/139	6.09%	69.65%	0.46	99.48%
TLab324	29,593,104	139/139	7.37%	71.00%	0.48	99.52%
TLab325	23,678,621	139/139	7.25%	73.31%	0.48	99.56%
TLab326	31,854,622	139/139	6.72%	76.28%	0.48	99.59%
TLab327	25,574,723	139/139	7.54%	72.30%	0.46	99.52%
TLab328	22,423,777	139/139	6.97%	72.45%	0.45	99.54%
TLab329	25,632,472	139/139	7.35%	72.88%	0.46	99.55%
TLab330	25,024,941	139/139	6.32%	72.58%	0.45	99.54%
TLab331	88,377,520	139/139	10.56%	71.98%	0.48	99.59%

Sample ID	Number of Reads	Read Length (bp)	% Abundant	% Aligned	Median CV Coverage Uniformity	% Stranded
TLab332	28,588,351	139/139	7.18%	74.70%	0.48	99.53%
TLab333	28,629,154	139/139	7.79%	74.01%	0.49	99.56%
TLab334	24,069,541	139/139	7.87%	71.77%	0.49	99.57%
TLab335	29,800,253	139/139	7.62%	71.22%	0.48	99.54%
TLab336	25,824,500	139/139	7.78%	70.08%	0.48	99.55%
TLab337	22,538,733	139/139	6.07%	69.81%	0.47	99.51%
TLab338	31,303,977	139/139	6.09%	70.94%	0.47	99.52%
TLab339	26,463,654	139/139	8.33%	71.67%	0.48	99.36%
TLab340	16,746,187	139/139	11.07%	66.77%	0.62	99.53%
TLab341	22,789,531	139/139	9.09%	73.96%	0.50	99.57%
TLab342	24,631,833	139/139	8.32%	72.84%	0.48	99.55%
TLab343	28,600,572	139/139	7.75%	70.60%	0.48	99.53%
TLab344	28,105,183	139/139	7.10%	72.24%	0.48	99.57%
TLab345	19,743,041	139/139	8.12%	70.05%	0.47	99.54%
TLab346	22,548,789	139/139	9.47%	66.13%	0.50	99.53%
TLab347	26,842,947	139/139	8.37%	69.78%	0.48	99.52%
TLab348	25,902,493	139/139	11.16%	71.00%	0.50	99.56%
TLab349	21,494,475	139/139	11.54%	75.32%	0.52	99.53%
TLab350	26,690,568	139/139	7.84%	76.54%	0.49	99.39%
TLab351	26,070,675	139/139	8.67%	73.43%	0.48	99.54%
TLab352	24,205,266	139/139	6.61%	72.05%	0.48	99.55%
TLab353	26,765,906	139/139	7.56%	70.19%	0.49	99.52%
TLab354	31,843,582	139/139	7.99%	70.89%	0.48	99.54%
TLab355	30,174,932	139/139	7.63%	71.86%	0.48	99.48%
TLab356	22,530,294	139/139	5.67%	72.16%	0.51	99.42%
TLab357	32,149,592	139/139	7.08%	74.96%	0.49	99.58%
TLab358	23,742,049	139/139	8.34%	73.83%	0.50	99.58%
TLab359	26,002,709	139/139	7.68%	74.91%	0.48	99.52%
TLab360	24,997,966	139/139	6.02%	73.43%	0.48	99.51%
TLab361	20,929,428	139/139	6.34%	73.55%	0.48	99.54%
TLab362	31,812,146	139/139	5.04%	73.66%	0.47	99.54%
TLab363	24,986,982	139/139	8.19%	72.68%	0.49	99.58%
TLab364	29,023,018	139/139	6.92%	71.72%	0.49	99.54%
TLab365	32,973,046	139/139	10.31%	75.94%	0.53	99.58%
TLab366	36,117,543	139/139	12.86%	80.53%	0.49	99.59%
TLab367	24,036,018	139/139	13.01%	76.42%	0.50	99.60%
TLab368	31,913,238	139/139	10.79%	75.42%	0.50	99.62%
TLab369	16,896,466	139/139	15.78%	79.82%	0.55	99.50%
TLab370	12,708,357	139/139	20.87%	76.99%	0.54	99.61%
TLab371	12,708,357	139/139	20.87%	76.99%	0.54	99.61%
TLab372	25,368,945	139/139	11.42%	72.26%	0.52	99.61%
TLab373	44,372,453	139/139	8.22%	78.37%	0.50	99.63%
TLab374	38,369,375	139/139	9.59%	74.95%	0.49	99.60%

Sample ID	Number of Reads	Read Length (bp)	% Abundant	% Aligned	Median CV Coverage Uniformity	% Stranded
TLab375	27,206,387	139/139	13.11%	75.80%	0.48	99.56%
TLab376	27,486,608	139/139	12.34%	73.26%	0.49	99.59%
TLab377	11,396,468	139/139	15.44%	80.00%	0.54	99.50%
TLab378	13,328,459	139/139	16.53%	75.95%	0.53	99.57%
TLab379	35,257,988	139/139	11.34%	74.39%	0.54	99.60%
TLab380	28,785,595	139/139	10.34%	73.49%	0.49	99.60%
TLab381	32,401,380	139/139	23.78%	74.94%	0.50	99.59%
TLab382	27,378,381	139/139	37.38%	70.91%	0.49	99.52%
TLab383	21,842,488	139/139	12.07%	74.02%	0.52	99.62%
TLab384	26,538,432	139/139	6.39%	75.46%	0.50	99.65%
TLab385	22,014,872	139/139	14.76%	74.90%	0.53	99.61%
TLab386	12,073,210	139/139	20.22%	59.11%	0.61	99.60%
TLab387	15,974,458	139/139	18.09%	71.74%	0.53	99.59%
TLab388	29,652,815	139/139	13.11%	73.21%	0.52	99.60%
TLab389	37,350,741	139/139	8.02%	71.89%	0.50	99.55%
TLab390	38,738,761	139/139	10.18%	72.69%	0.49	99.56%
TLab391	36,831,877	139/139	30.10%	66.74%	0.49	99.38%
TLab392	36,434,644	139/139	13.16%	74.01%	0.49	99.61%
TLab393	29,125,909	139/139	18.30%	79.39%	0.54	99.45%
TLab394	33,910,721	139/139	12.37%	78.76%	0.53	99.46%
TLab395	36,478,596	139/139	7.44%	72.61%	0.50	99.62%
TLab396	32,915,600	139/139	6.52%	67.13%	0.52	99.63%
TLab397	34,237,987	139/139	8.91%	74.86%	0.49	99.55%
TLab398	35,175,843	139/139	9.18%	72.48%	0.48	99.54%
TLab399	87,940,398	139/139	8.48%	73.55%	0.48	99.51%
TLab400	37,350,750	139/139	8.83%	74.16%	0.48	99.53%
TLab401	23,296,572	139/139	9.78%	82.01%	0.54	99.35%
TLab402	28,001,767	139/139	8.26%	79.98%	0.52	99.41%
TLab403	22,920,324	139/139	10.35%	68.88%	0.59	99.54%
TLab404	32,714,497	139/139	6.64%	73.55%	0.48	99.54%
TLab405	27,532,984	139/139	10.88%	77.77%	0.58	99.57%
TLab406	38,052,772	139/139	7.41%	76.67%	0.46	99.53%
TLab407	34,441,010	139/139	7.78%	77.92%	0.47	99.55%
TLab408	33,552,795	139/139	9.15%	74.90%	0.49	99.54%
TLab409	21,983,930	139/139	9.80%	83.15%	0.54	99.39%
TLab410	18,971,595	139/139	9.26%	78.90%	0.54	99.36%
TLab411	32,032,582	139/139	6.15%	71.97%	0.51	99.54%
TLab412	36,146,096	139/139	7.83%	75.83%	0.49	99.53%
TLab413	28,058,386	139/139	7.48%	76.16%	0.48	99.50%
TLab414	37,319,356	139/139	7.26%	76.05%	0.47	99.49%
TLab415	33,255,963	139/139	9.07%	75.79%	0.49	99.57%
TLab416	30,941,667	139/139	8.24%	75.00%	0.53	99.59%
TLab417	25,631,889	139/139	7.26%	77.83%	0.52	99.49%

Sample ID	Number of Reads	Read Length (bp)	% Abundant	% Aligned	Median CV Coverage Uniformity	% Stranded
TLab418	32,172,141	139/139	6.75%	76.48%	0.49	99.55%
TLab419	27,393,718	139/139	5.95%	72.37%	0.46	99.48%
TLab420	29,412,589	139/139	5.91%	73.39%	0.48	99.52%
TLab421	38,574,376	139/139	6.97%	74.63%	0.48	99.54%
TLab422	35,068,162	139/139	8.35%	73.42%	0.48	99.53%
TLab423	34,602,296	139/139	7.57%	74.46%	0.47	99.54%
TLab424	35,329,973	139/139	6.90%	72.60%	0.47	99.57%
TLab425	29,047,374	139/139	8.05%	78.78%	0.51	99.47%
TLab426	28,984,798	139/139	6.49%	75.38%	0.49	99.55%
TLab427	30,212,400	139/139	7.93%	72.13%	0.48	99.51%
TLab428	39,448,421	139/139	8.17%	72.00%	0.48	99.53%
